# Prognostic value of circulating plasma cells in patients with multiple myeloma: A meta-analysis

**DOI:** 10.1371/journal.pone.0181447

**Published:** 2017-07-13

**Authors:** Jia Li, Ningning Wang, Nahom Tesfaluul, Xiaojuan Gao, Shuai Liu, Baohong Yue

**Affiliations:** 1 Department of Laboratory Medicine, the First Affiliated Hospital of Zhengzhou University, Zhengzhou, P. R. China; 2 Faculty of Laboratory Medicine, Zhengzhou University, Zhengzhou, P. R. China; 3 Key Laboratory Medicine of Henan Province, Faculty of Laboratory Medicine of Zhengzhou University, Zhengzhou, P. R. China; 4 Open Laboratory, Henan Province Key Subject of Clinical Medicine, Zhengzhou, P. R. China; National Cancer Center, JAPAN

## Abstract

**Purpose:**

The clinical significance and prognostic role of circulating plasma cells (CPCs) in multiple myeloma (MM) are still controversial. We conducted the first meta-analysis to clarify the correlation between CPCs and the clinicopathological features and prognosis of MM patients.

**Methods:**

A comprehensive literary search for relevant studies was performed on PubMed, Embase, Medline, CNKI (Chinese) and Web of Science databases (January 1, 1950 to December 20, 2016). The associations between CPCs and survival rate and clinicopathological parameters, including International staging system (ISS) and Durie-Salm staging system (DS) stage, were evaluated. Then pooled hazard ratios (HRs) for survival with 95% confidence intervals (CIs), subgroup analysis, sensitivity analysis, and publication bias were conducted.

**Results:**

11 studies covering a total of 2943 patients were included. Pooled hazard ratios (HRs) revealed that the presence of CPCs predicted aggressive disease progression (HR = 1.78, 95% CI = 1.57–2.03) and reduced overall survival (OS) (HR = 1.82, 95% CI = 1.59–2.08). Subgroup analyses demonstrated that CPCs positive patients also had poor disease progression and OS in detection methods and sampling time subsets. Moreover, the presence of CPCs was strikingly associated with increased ISS stage (OR = 2.78% CI = 1.69–4.56), but not with DS stage(OR = 1.60; 95% CI = 0.74–3.47).

**Conclusions:**

CPCs status is associated with poorer survival outcome in multiple myeloma. Additionally, increased ISS stage could be significant risk factors for the presence of CPCs.

## Introduction

Multiple myeloma (MM) is a hematological malignancy characterized by the proliferation of clonal plasma cells in the bone marrow [1], predominantly occurring in the elderly with an incidence of 10,000 deaths per year in the United States and Europe [2]. The advent of novel proteasome inhibitors and immunomodulatory drugs has significantly improved response rates and progression-free survival [3]. However, MM can often become refractory to treatment. Therefore, early identification of disease progression and relapse has become increasingly important for prognosis in MM patients.

As it is known, bone marrow plasma cells examination is a golden standard for evaluation the tumor burden and an indicator to assess the prognosis and response for MM patients [4]. Whereas, several studies utilizing various methods suggested that there were small numbers of plasma cells in peripheral blood [5], namely, circulating plasma cells (CPCs), and demonstrated that it had prognostic value among MM, MGUS [6], smoldering MM [7,8] and amyloidosis [9]. However, the prognostic value of CPCs in MM remains controversial. Some studies revealed that CPC status could predict poorer survival outcomes, while other studies failed to support this conclusion [10]. In addition, Peceliunas et al. [11] showed that CPCs status had prognostic relevance for time to tumor progression (TTP) but not for overall survival (OS) in MM patients. Similarly, Vagnoni et al. [12] proposed that CPCs status was associated with time to tumor progression (TTP) but not for overall survival (OS) in multivariate analysis. Interestingly, even in the same trial, CPCs detected at different time points indicated different prognoses of survival for MM participants [13]. These discrepancies may result from the small sample sizes used in these studies as well as differences in the sampling times and detection methods used.

The clinicopathological features such as ISS and DS stages of MM patients with CPCs have been analyzed in several studies, but controversies exist. Some studies [12,13] showed that the level of CPCs appeared to be largely independent of ISS and DS stages. However, in other studies, the presence of CPCs correlated more closely with DS [10] and ISS [10,14,15] stages. Hence, we also evaluated the association between CPCs and ISS, DS stages.

With the development of highly sensitive and specific diagnostic methods, including polymerase chain reaction (PCR), flow cytometry (FCM), slide-based immunofluorescence assay (IF) and conventional morphology (CM), it would be desirable to explore whether CPCs may serve as one biomarker for prognostic prediction and treatment option in MM. Here, we conducted the first meta-analysis to provide a better insight into the prognostic value of CPCs on disease progression and OS in MM patients. Furthermore, subgroup analyses were performed to evaluate whether the detection methods and time points of blood collection influence the prognostic value of CPCs.

## Materials and methods

### Search strategy

A literary search was performed on PubMed, Embase, Medline, CNKI (Chinese) and Web of Science databases (January 1, 1950 to December 20, 2016). Search term combinations were “multiple myeloma,” “myeloma,” “plasma cell myeloma,” “circulating plasma cell,” “circulating myeloma cell,” “peripheral blood plasma cell,” “monoclonal plasma cell,” in title/abstract. We also attempted to identify other potentially relevant articles by searching the reference sections of qualified manuscripts. we contacted authors by email if the data in studies was insufficient. If authors could not be contacted, the studies subsequently excluded.

### Selection criteria and quality assessment

Studies were included only if they following the inclusion criteria: (1) focus on the correlations between CPCs status and clinicopathological features or survival outcomes (either disease progression or OS) in MM patients; (2) sufficient data was provided to extract HR with 95% confidence intervals (CIs); (3) when the same study cohort was published at several reports, only the most complete one was included in our meta-analysis; (4) each study enrolling more than 20 patients; (5) published in English. Studies such as reviews, letters, editorials, conference meeting, case reports abstracts and comments were excluded. We also excluded studies in which the outcomes of interest were not reported or if it was impossible to calculate outcomes from the originally published data. Two investigators independently evaluated the quality of included studies using the Newcastle-Ottawa Scale (NOS) for cohort study [16]. Any discrepancy was resolved by discussion or consultation with a third party if required.

### Data extraction

Two of the authors independently collected the following data from each eligible study: first author’s name, publication year, study design, country, age, number of subjects analyzed, disease stage, median follow-up, sampling times, detection method, and cutoff of CPCs. HRs with 95% CIs for OS and disease progression including progression-free survival (PFS), relapse-free survival (RFS), time to progression (TTP), time to next therapy (TTNT) and event-free survival (EFS). HRs and 95% CIs were extracted from multivariable analyses. If the HR were not provided directly in the original study, we calculated these values from available reported data using software designed by Tierney et al. [17]. When CPCs was detected according to different time points in one study, such as before and after stem cell transplantation, both data were extracted.

### Statistical analysis

The extracted information was analyzed using STATA software version 12.0. The disease progression and OS outcomes were evaluated by HR. When analyzing the association between CPCs and ISS, DS stage, Odd ratio (OR) was calculated. Simultaneously, subgroup analysis was performed on the basis of the detection methods and sample times. The pooled HRs using a fixed- or random-effect model was according to heterogeneity. Heterogeneity was tested using Cochran’s Q test and quantified by the I^2^ index, which is considered significant if P < 0.10 or I^2^ > 50% by convention. To evaluate the robustness of the results of our meta-analysis in the presence of uncertainty, we performed a sensitivity analysis estimating the average HR by omitting one study each time. Publication bias was evaluated with Begg’s and Egger’s test (a p-value < 0.05 was considered statistically significant).

## Results

### Description of included studies

**Fig 1:**
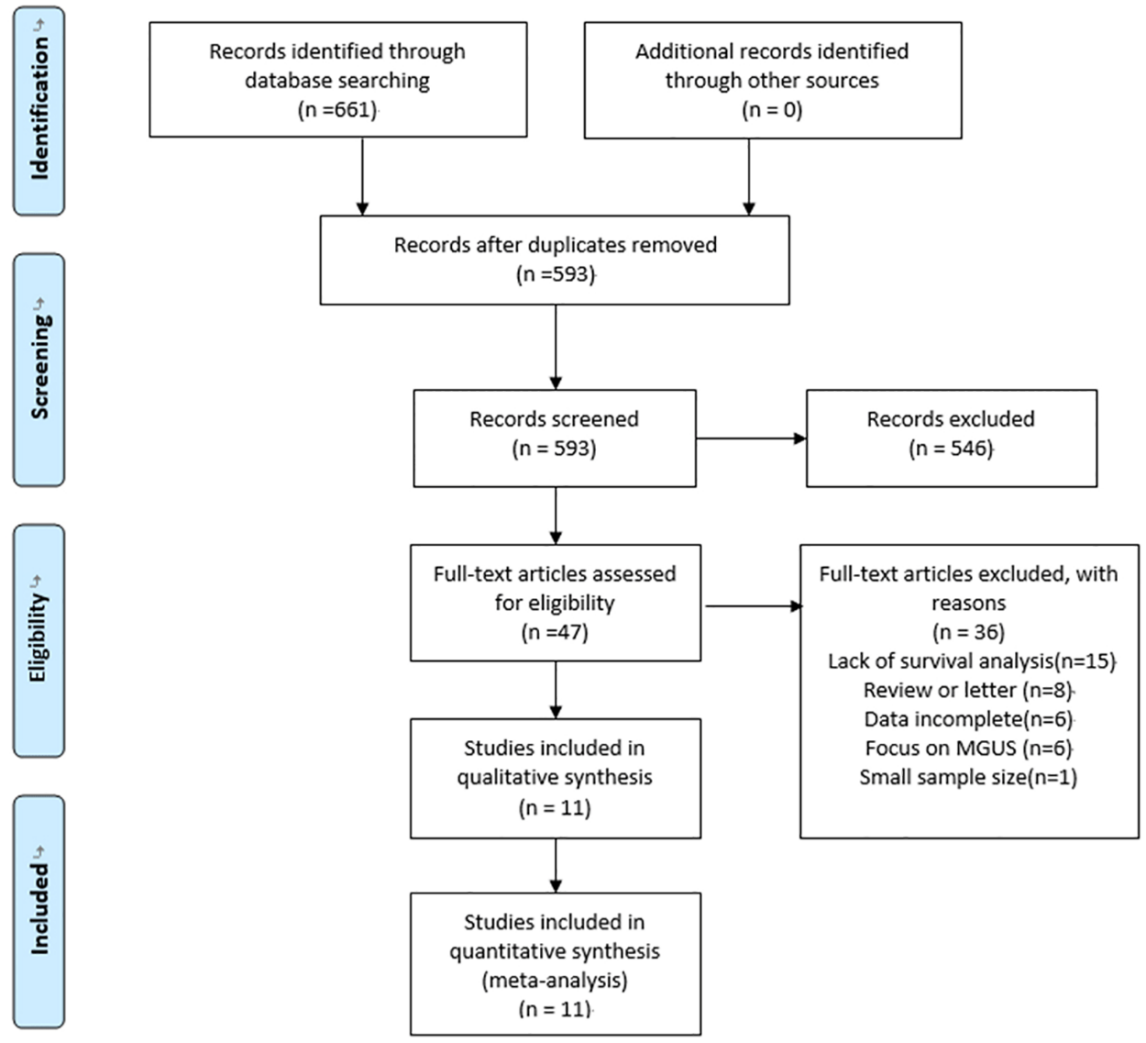
Flow diagram showing the selection process for the including studies.

A total of 661 potentially studies were identified using our search strategy. 612 studies were deemed ineligible after title and abstract screening. 46 potential studies were reviewed via the full-text. Then 36 studies were excluded because they were not meet the inclusion criteria, leaving 11 studies [10–15,18–22] that were eligible included in the meta-analysis (Fig 1).

2943 MM patients were included and were conducted in 5 countries (China, Germany, USA, Lithuania and Italy), published between 1996 and 2016. Peripheral blood were analyzed and methods used to detect CPCs were FCM, PCR, IF and CM. The sampling times were divided into 4 time points (first diagnosis, before stem cell transplantation, after stem cell transplantation, and relapsed/refractory MM). 647 relapsed/refractory (RA) MM patients were included in Gonsalves’s study [22], but only 145 actively relapsing patients were analyzed in the Kaplan-Meier OS distributions. Korthal’s study [13] detected CPCs before and after stem cell transplantation (SCT) and both have the prognostic analysis, so two HRs were extracted. HRs with 95% CIs were directly extracted from original articles in 6 studies [12,14,15,18,20,22]. Five studies[10,11,13,19,21] did not report HRs with 95% CIs directly, so these values were calculated according to the method suggested by Tierney et al. [17]. The characteristics and the quality of the included studies evaluated with the NOS are summarized in Table 1.

**Table 1:** Main characteristics of included studies.

Study	Study design	Country	No. of patients(M/F)	Age (median, range)	Stage	Detectionmethod	Cutoff of CPCs	Sampling times	Follow up	NOS	Outcome	HR	95%CI
Witzig 1996	Pro	USA	254(152/102)	63.6(20.0–92.9)	NR	IF	3×10^6^/L	First diagnosis	NR	6	OS	2.05	1.45–2.91
Gertz 1997	Pro	USA	33(19/14)	52(32–64)	NR	IF	0.2×10^6^/L	B-SCT	NR	5	RFS	1.58	0.52–4.80
											OS	1.26	0.34–4.68
Nowakowski 2005	Pro	USA	302(180/123)	65(29–94)	NR	FCM	10 cells/50 000	First diagnosis	33.5	7	OS	1.42	1.01–1.99
Dingli 2006	Pro	USA	246(155/91)	57.2(30–74)	D-S I-III	FCM	1 cells/50 000	B-SCT	34	8	TTP	1.48	1.09–1.99
											OS	1.64	1.10–2.43
Peceliunas 2012	Pro	Lithuania	42(NR)	57(39–74)	NR	FCM	20 cells/1 000 000	RA	21	7	OS	2.33	1.01–5.36
Korthals 2013	Pro	Germany	21(NR)	55(44–65)	ISS I-III	PCR	0.01%	B-SCT	45	8	EFS	1.53	0.18–13.36
					D-S I-III						OS	1.76	0.12–25.79
Korthals 2013*	Pro	Germany	32(NR)	55(42–62)	ISS I-III	PCR	0.01%	A-SCT	45	8	EFS	4.41	1.56–12.48
					D-S I-III						OS	5.88	1.03–33.59
Gonsalves 2014	Retro	USA	157(93/62)	65(39–95)	ISS I-III	FCM	400 cells/150 000	First diagnosis	NR	5	TTNT	1.85	1.07–3.16
											OS	3.16	1.43–7.08
Gonsalves WI 2014	Retro	USA	145(NR)	63(43–80)	NR	FCM	100 cells/150 000	RA	21	8	OS	2.67	1.37–5.19
An 2015	Retro	China	767(471/296)	59	ISS I-III	CM	2%	First diagnosis	41	8	PFS	1.54	1.22–1.95
					D-S I-III						OS	1.59	1.26–2.00
Vagnoni 2015	Pro	Italy	104(52/52)	72(45–85)	ISS I-III	FCM	41 cells/50 000	First diagnosis	35.9	6	PFS	2.63	1.51–5.92
					D-S I-III								
Chakraborty 2016	Retro	USA	840(500/340)	61.1(24.4–76.1)	ISS I-III	FCM	1 cells/15 0000	B-SCT	44	8	PFS	2.03	1.64–2.50
											OS	2.52	1.78–3.55

* Same literature but different sampling time

NR: Not Reported; HR: hazard ratio; CI: confidence intervals; NOS: Newcastle-Ottawa Scale; CPCs: circulating plasma cells; IF: immunofluorescence; PCR: polymerase chain reaction; FCM: flow cytometry; CM: conventional morphology; B-SCT: before stem cell transplantation; A-SCT: after stem cell transplantation; RA: relapsed or refractory multiple myeloma; ISS: international staging system; DS: Durie-Salm staging system; OS: overall survival; PFS: progression-free survival; RFS: relapse-free survival; TTP: time to progression; TTNT: time to next therapy; EFS: event-free survival; Pro: prospective; Retro: retrospective

### Overall analyses

The HRs for disease progression were available in 8 studies [10–15,19,21]. In Korthal’s study [13], two HRs were extracted according to different sampling times. The estimated pooled HR showed an increased risk of disease progression in patients with CPC positive group (HR = 1.78, 95%CI = 1.57–2.03). The heterogeneity among studies was not noted (*P* = 0.327, I^2^ = 12.9%). Data on OS were available in 9 studies [10,13–15,18–22]. Two HRs were extracted in Korthal’s study for the same reason as mentioned above. The pooled HRs showed a significantly increased risk of mortality in patients with CPC positive group (HR = 1.82, 95%CI = 1.59–2.08). Heterogeneity among studies was not noted (*P* = 0.186 and I^2^ = 28.1%).

### Subgroup analyses

**Fig 2:**
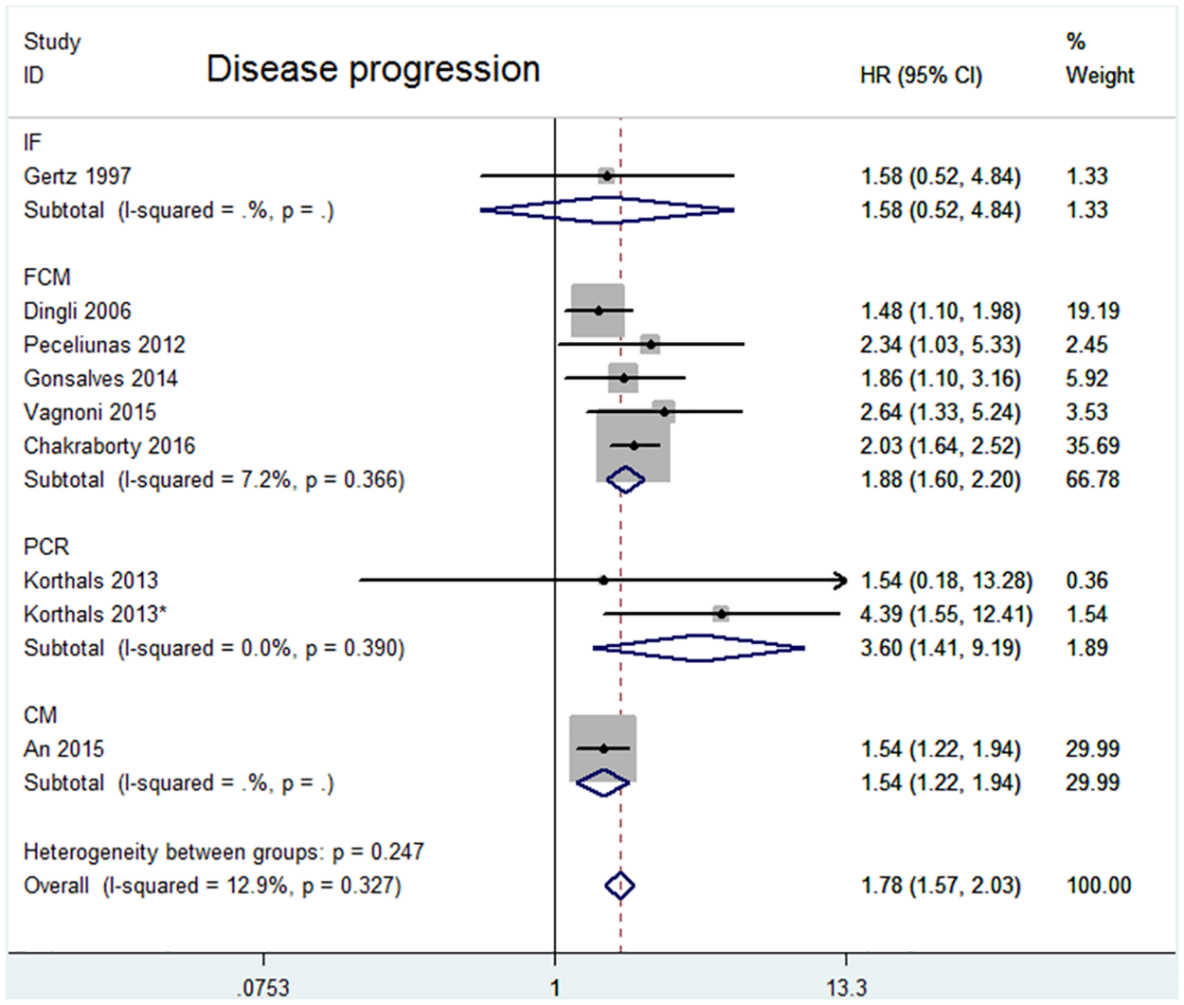
Forest plot of HRs and 95%CIs for disease progression. Subgroup analysis was according to different detection methods. The black boxes’ sizes are proportional to the study weight, with the lines indicating 95% confidence intervals (CIs).

**Fig 3:**
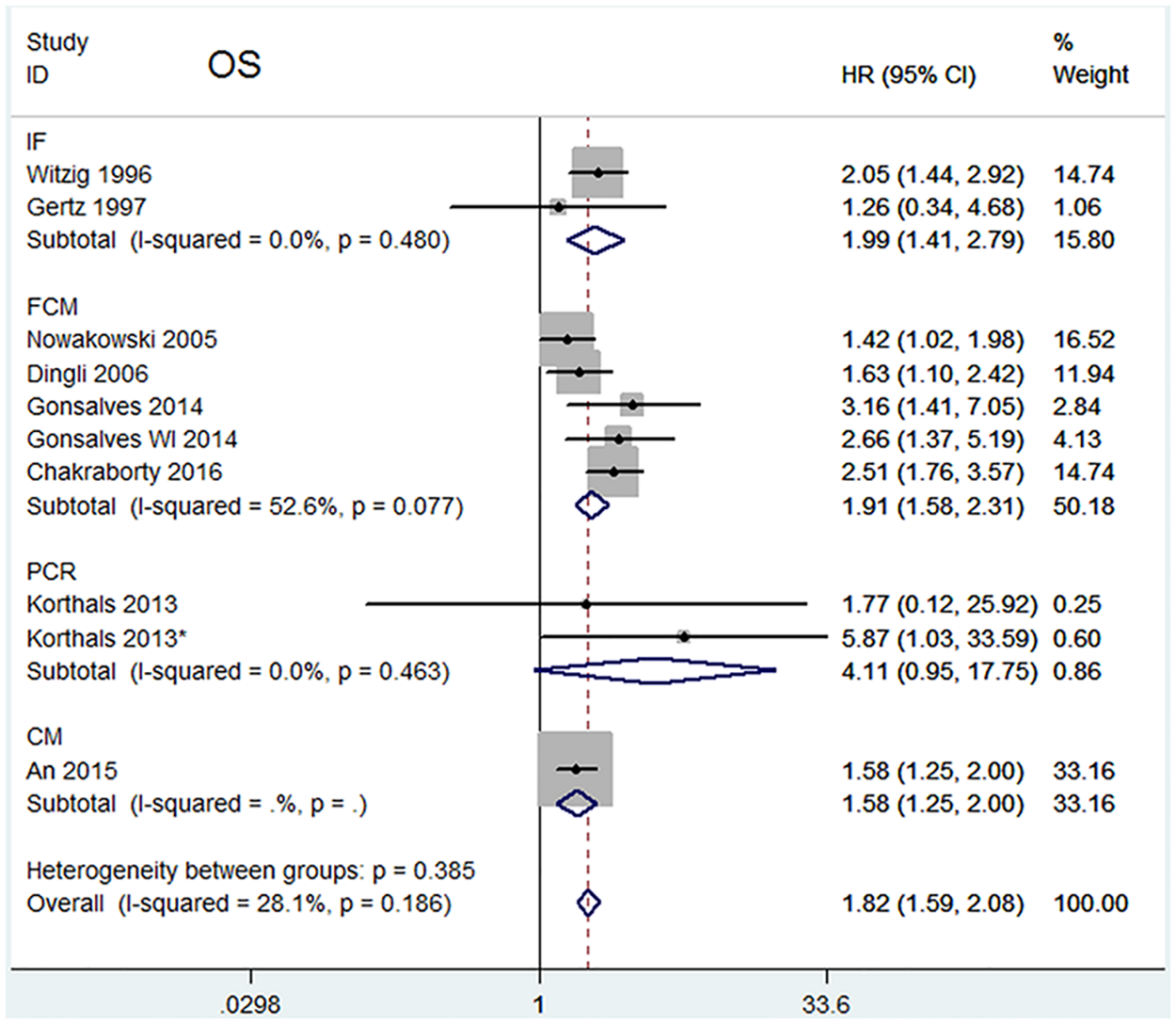
Forest plot of HRs and 95%CIs for OS. Subgroup analysis was according to different detection methods. The black boxes’ sizes are proportional to the study weight, with the lines indicating 95% confidence intervals (CIs). HR higher than 1 indicate that the presence of CPCs is associated with worse prognosis.

When assessing the effects of CPCs status on outcomes for different detection methods, the “FCM” subgroup and “CM” subgroup indicated a worse prognosis for both disease progression (FCM: HR = 1.88, 95%CI = 1.60–2.20; CM: HR = 1.54, 95%CI = 1.22–1.94)(Fig 2) and OS (FCM: HR = 1.91, 95% CI = 1.58–2.31; CM: HR = 1.58, 95%CI = 1.25–2.00)(Fig 3). However, the “PCR” subgroup exhibited prognostic significance for disease progression (HR = 3.60, 95%CI = 1.41–9.19) but not for OS (HR = 4.11, 95% CI = 0.95–17.75). The “IF” subgroup showed prognostic significance for OS (HR = 1.99, 95% CI = 1.41–2.79) but not for disease progression (HR = 1.58, 95%CI = 0.52–4.84). Significant heterogeneity was not found in “FCM” subgroup, “CM” subgroup, “PCR” subgroup and “IF” subgroup.

**Fig 4:**
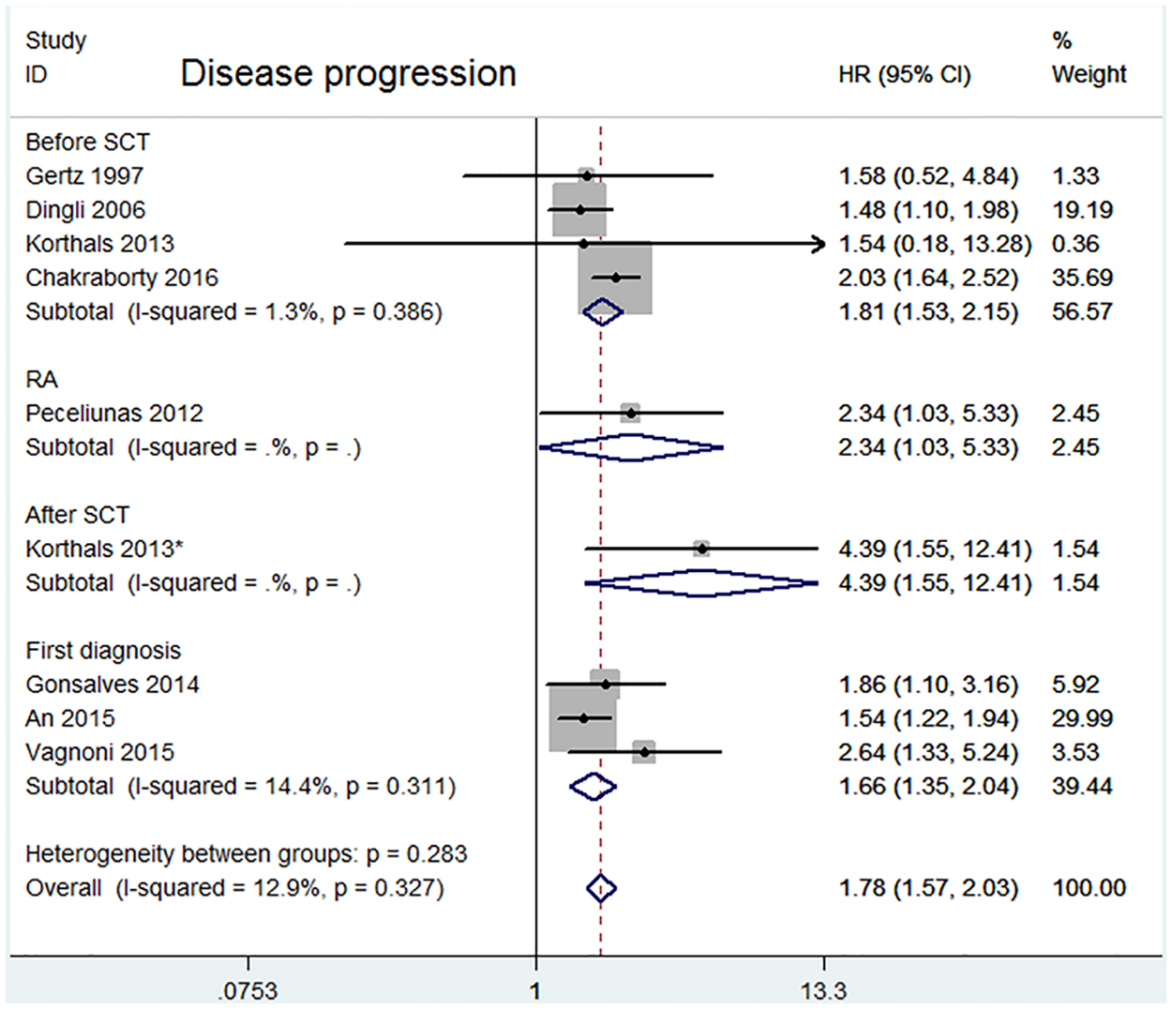
Forest plot of HRs and 95%CIs for disease progression. Subgroup analysis was according to different sampling time: four studies assessed CPCs before SCT; three studies assessed CPCs at first diagnosis. HR higher than 1 indicate that the presence of CPCs is associated with worse prognosis.

**Fig 5:**
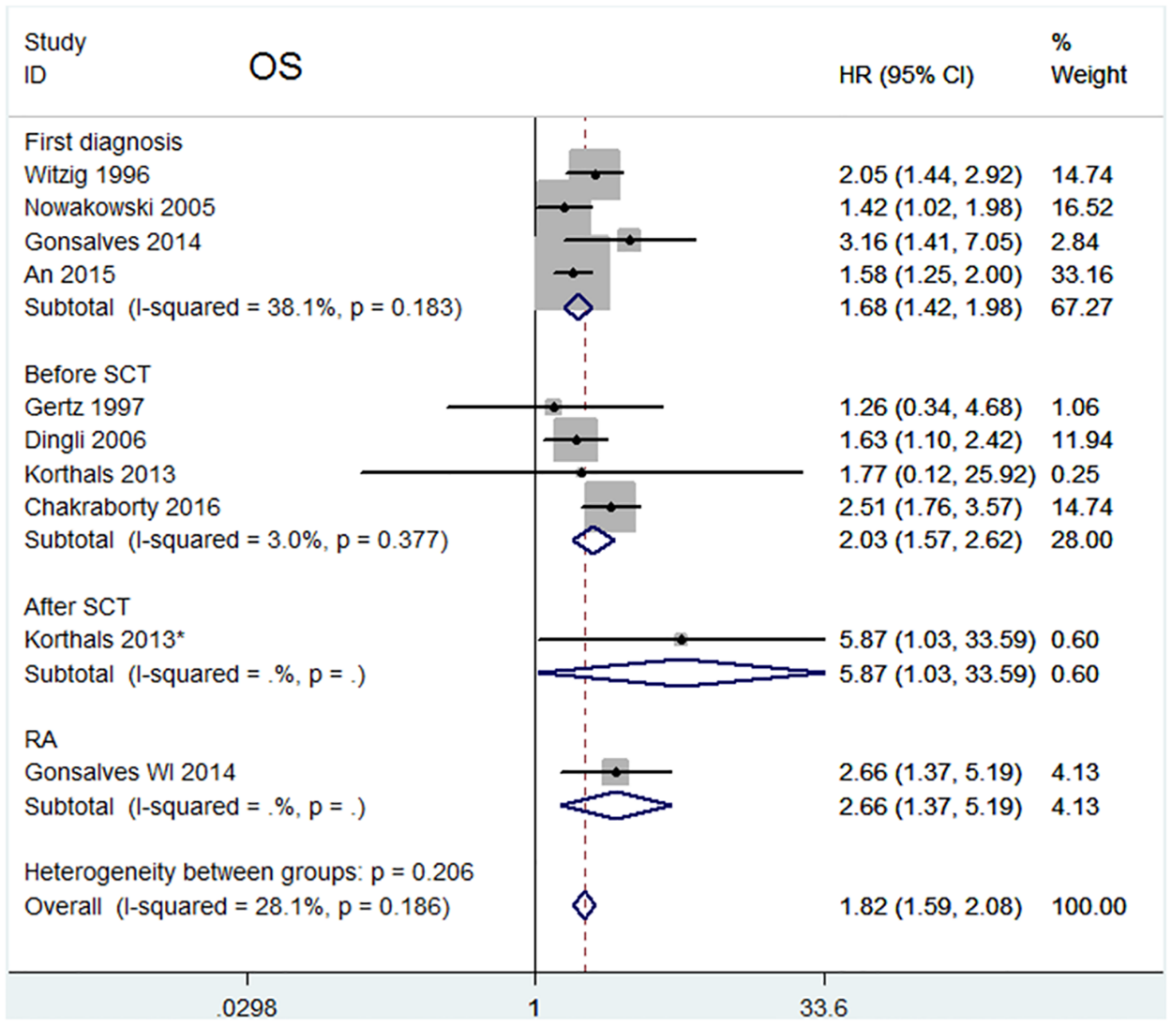
Forest plot of HRs and 95%CIs for OS. Subgroup analysis was according to different sampling time: four studies assessed CPCs at first diagnosis; four studies assessed CPCs before SCT. HR higher than 1 indicate that the presence of CPCs is associated with worse prognosis.

In addition, we explored the effects of CPCs status on outcomes for various sampling times. CPCs detected at first diagnosis and before SCT indicated an increased risk for both disease progression (First diagnosis: HR = 1.66, 95%CI = 1.35–2.04; Before SCT: HR = 1.81, 95% CI = 1.53–2.15) (Fig 4) and OS (First diagnosis: HR = 1.68, 95% CI = 1.42–1.98; Before SCT: HR = 2.03, 95% CI = 1.57–2.62) (Fig 5). CPCs detected at RA and After SCT exhibited prognostic significance for both disease progression (RA: HR = 2.34, 95% CI = 1.03–5.33; after SCT: HR = 4.39, 95%CI = 1.55–12.41) and OS (RA: HR = 2.66, 95% CI = 1.37–5.19; after SCT: HR = 5.87, 95% CI = 1.03–33.59), but both of subgroups only had 1 study.

### CPCs and ISS, DS stage

**Fig 6:**
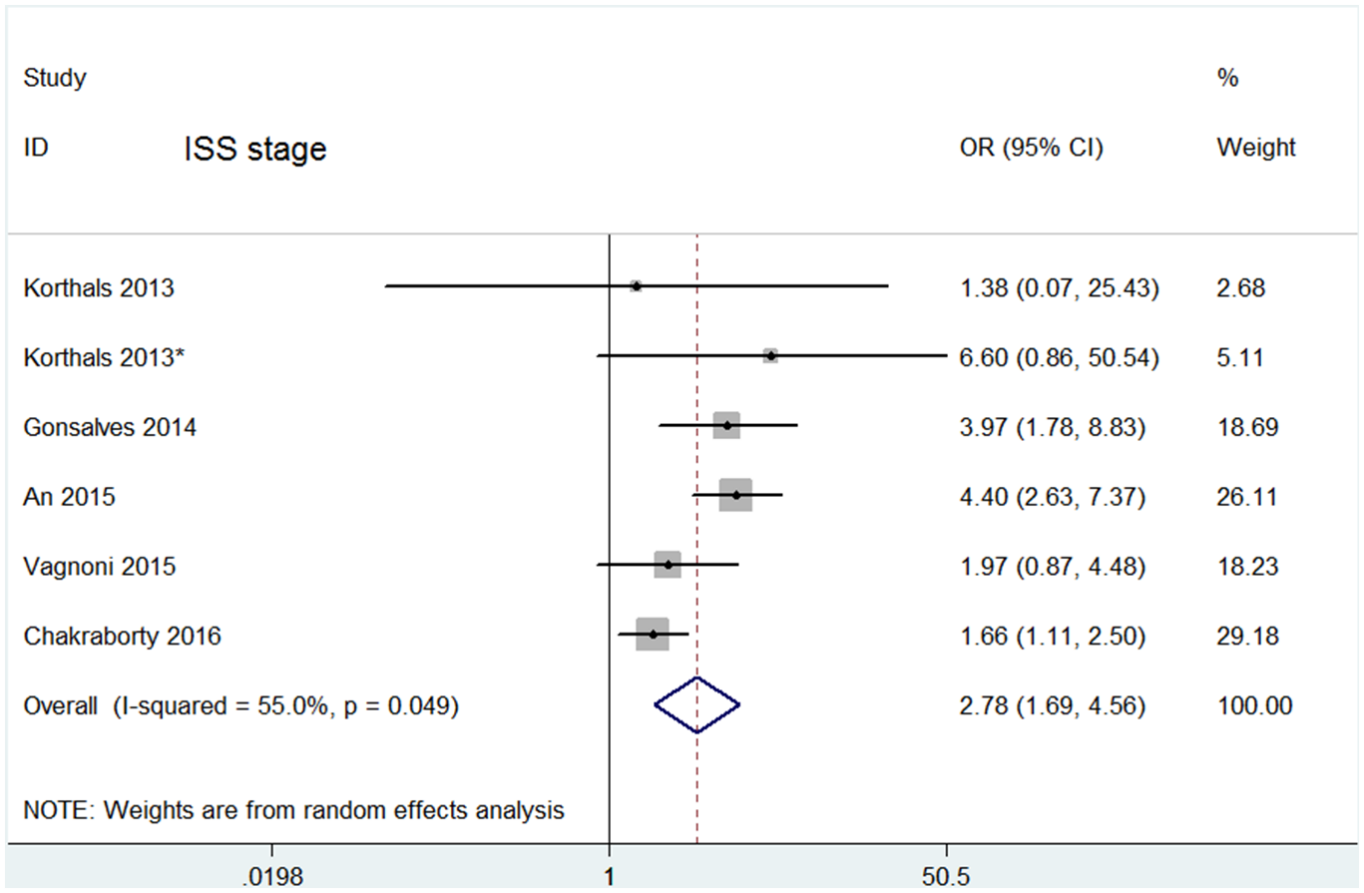
Forest plots of the association between CPCs and ISS stage. Odd ratio (OR) higher than 1 indicate that CPCs were more frequently detected in patients with increased ISS stage.

The ISS and DS stage are two criteria for the diagnosis of multiple myeloma. 5 studies[10,12–15] reported the relationship between CPCs positivity and ISS, while other 4 studies[10,12,13,21] reported DS stage separately. The overall positive rate of CPCs in ISS I~II stage group was 13.88% lower than the 26.61% of ISS III group, whereas in DS I~II stage and DS III stage are 18.34% and 22.97% respectively. Pooled analysis also showed that CPCs positivity in ISS III is greater than that of ISS I~II (OR = 2.78, 95% CI = 1.69–4.56, random effects), the heterogeneity among studies is present (I^2^ = 55.0%, p = 0.049) as shown in Fig 6, but not in the DS stage (OR = 1.60, 95% CI = 0.74–3.47, random effects), with significant heterogeneity between studies (I^2^ = 54.4%, p = 0.067) as shown in S1 Fig.

### Sensitivity analyses and publication bias

A sensitivity analysis was performed by removing one study each time and analyzing the heterogeneity across the remaining studies. Our result showed that no individual studies significantly influenced the HRs of disease progression (S2 Fig) and OS (S3 Fig). Begg’s test showed that no significant publication bias was found in the pooled analysis of disease progression (S4 Fig) and (S5 Fig). Furthermore, we also performed Egger’s test for disease progression and OS. The P value of the Egger’s test was 0.317 and 0.212, which were > 0.05, also demonstrating that there was no publication bias existed.

## Discussion

Many studies have shown that the presence of CPCs was significantly associated with prognosis or other clinicopathologic parameters in MM patients. While, the lack of similar detection methods or consistent cutoff values in different study designs has hampered efforts to effectively treat and monitor disease progression. In an effort to prove the clinical significance of CPCs, we presented the first meta-analysis to evaluate the value of CPCs in MM patients. Overall, our results demonstrated that patients in CPC positive group had a worse OS and more aggressive disease progression compared with CPC negative group. Moreover, the presence of CPCs was associated with elevated ISS, but not with DS stage.

Subgroup analysis for OS and disease progression by detection methods showed that “FCM” subgroups presented significant associations with no heterogeneity, whereas it was not significant in the "PCR" subgroup. Though Paiva et al. [23] have revealed that PCR-based approach is more sensitive (10^−6^) as compared with FCM-based method (10^−4^), it has some limitations. PCR–based technique requires fixed and stable gene mutation or fusion gene fragment for individual follow-up of patients [24–27], so it can only be applied to approximately 75% of patients [25]. Moreover, cells detected by PCR requiring specific primers are difficult to reflect the corresponding amount of tumor cells in vivo, and this may explain why CPCs detected by PCR wasn’t associated with OS. FCM for the detection of CPCs is widely used with objectivity, high efficiency and strong reproducibility, since more than 90% of MM patients express plasma cell aberrant immunophenotype [24]. More importantly, the sensitivity of FCM can be improved through acquiring a large number of cells such as 150 000 events [14,15], 100 0000 events [11]. The “IF” subgroup showed prognostic significance for OS but not for disease progression. A slide-based immunofluorescence (IF) assay can be subjective, low sensitive depending on the ability of the morphologist consuming intense labor and much time to recognize CPCs, thus limiting its clinical application [14]. A rational explanation of An et al. Study [10] is that compared with the above methods, the sensitivity and specificity of conventional morphology (CM) are lowest, and the number of cells analyzed is least. Consequently, FCM used to detect CPCs was more likely to associate with OS according to our subgroup analysis.

One essential question was whether CPCs in peripheral blood at a single time point were of prognostic relevance. So further subgroup analysis based on sampling times including at the time of first diagnosis, RA, before SCT and after SCT were performed. Pooled HRs for OS and disease progression were fairly stable and not influenced by the sampling times. This suggested that the presence of CPCs in peripheral blood indicates poor prognosis in MM patients. Considering that “RA” and “After-SCT” subgroups only included one study, more research was required to assess the effectiveness and stability of these sampling times.

Our meta-analysis also underscored the presence of CPCs being closely associated with elevated ISS stage. This suggests that CPCs were more frequently detected in patients with increased ISS stage. Whereas DS stage failed to shows this association. Prognostic factors involve clinical features, laboratory parameters and imaging examinations. ISS stage based on serum albumin and β2-microglobulin, adopted by WHO as a unified system for MM, is mainly applied to determine the prognosis [28]. It has prognostic value for patients who received conventional treatments as well as autologous hematopoietic stem cell transplantation [29]. However, with the development of newer therapies, the DS system has less prognostic impact on survival [29]. This may be associated with the fact that these treatments can strikingly reduce tumor burden and the DS stage predominantly reflects tumor burden [28], thus weakening its significance. Kumar et al. [30]and Nowakowski et al. [20]have also supported that CPCs correlated with aggressive disease rather than tumor burden. CPCs have higher propensity to circulate, strong ability to migrate and invade, then spread the tumor cells to various parts of the bone marrow, and lead to metastasis or relapse [31]. This proposed model for spread shows intriguing similarities to epithelial–mesenchymal transformation (EMT) in solid tumors [32]. Significant heterogeneity was observed in the ISS and DS data, sample size might have contributed to the heterogeneity. More large-scale studies are warranted to validate the clinical power of CPCs status.

Our study has some limitations. First, our meta-analysis included only 11 eligible studies, of which were small retrospective case-series, which resulted in relatively insufficient statistical power with regard to estimating the prognostic role of CPCs in MM patients. Thus larger, prospective studies should be performed. Second, some HRs were extracted from the survival curves, which inevitably brought about small statistical errors. Third, CPCs detection methods and cutoff values were different among 11 included studies, but FCM was most broadly applied in 7 studies, and the ranges of cut-off values in First diagnosis, RA and B-SCT group were 2~26.67×10^−4^, 0.2~6.67×10^−4^ and 0.067~0.2×10^−4^ respectively, mainly fluctuating at 10^−4^. Some studies[23] [25]also demonstrate that the sensitivity of FCM to detect minimal residue disease is about 10^−4^. Certainly, a unified detection method and cutoff value still need to be established. Finally, we did not acquire all effective clinicopathological data such as β2-microglobulin, cytogenetic abnormality, and salvage treatment options from the included studies. If these data were available, our meta-analysis might have provided sufficient evidence to evaluate the impact of treatment intervention.

In conclusion, our meta-analysis indicates that the presence of CPCs was associated with aggressive disease course and poor OS in MM patients. CPCs status was strongly associated with elevated ISS stage and represents aggressive disease rather than tumor burden. Regardless of whether CPCs are detected in an early stage or in relapse patients, CPCs status may serve as a useful tool to guide the prognosis of MM patients. Considering the limitations of present analysis, further prospective multicentre studies designed with larger sample size and unified detection methods are needed.

## Supporting information

S1 File PRISMA checklist. (DOC)

S2 File Search strategy of this study. (DOCX)

S1 Fig Forest plots of the association between CPCs and DS stage. Odd ratio (OR) higher than 1 indicate that CPCs were more frequently detected in patients with increased DS stage.
(TIF)

S2 Fig Sensitivity analysis for the pooled HRs in disease progression. (TIF)

S3 Fig Sensitivity analysis for the pooled HRs in OS. (TIF)

S4 Fig Begg’s funnel plots of the prognostic role of CPCs in disease progression. (TIF)

S5 Fig Begg’s funnel plots of the prognostic role of CPCs in OS. (TIF)
