# Expression of Concern: Prognostic value of circulating plasma cells in patients with multiple myeloma: A meta-analysis

**DOI:** 10.1371/journal.pone.0282230

**Published:** 2023-02-21

**Authors:** The PLOS ONE Editors

After this article was published, similarities were noted between this article [1] and submissions by other research groups which call into question the validity and provenance of the reported results.

In response to queries about these concerns, the first author provided the underlying data in S1 File. During editorial follow-up, it was noted that the statement in the Results section in article [1] that 46 potential studies were reviewed via the full-text is an error and should state that 47 potential studies were reviewed via the full-text.

The authors commented on aspects of how data were collected and analyzed for this study, but overall their responses did not fully resolve the concerns.

The *PLOS ONE* Editors issue this Expression of Concern to notify readers of the unresolved concerns discussed above, and to provide the data received from the authors.

## Supporting information

S1 File The underlying data used for the analysis in this article [1]. (ZIP)
