# Supplementary material for: Prognostic value of circulating plasma cells in patients with multiple myeloma: A meta-analysis
Source: PLoS One. 2017 Jul 13;12(7):e0181447. doi: 10.1371/journal.pone.0181447 (PMC5509371; doi:10.1371/journal.pone.0181447)
Supplement: S2 File — (DOCX) [file pone.0181447.s002.docx]

**Search strategy pubmed:**

| #3 | #1 AND #2 |
| --- | --- |
| #2 | Search ((((((((circulating plasma cells[Title/Abstract]) OR circulating myeloma cells[Title/Abstract]) OR monoclonal plasma cells[Title/Abstract]) OR peripheral blood plasma cells[Title/Abstract]) OR Circulating Multiple Myeloma Cells[Title/Abstract]) OR peripheral blood circulating plasma cells[Title/Abstract])) OR CPCs[Title/Abstract]) OR CMCs[Title/Abstract] |
| #1 | Search ((Multiple Myeloma[MeSH Terms]) OR myeloma[Title/Abstract]) OR Plasma Cell Myeloma[Title/Abstract] |

**Search strategy web of science:**

| # 4 | #3 AND #2 AND #1  Refined by: LANGUAGES: (ENGLISH)  Indexes=SCI-EXPANDED, SSCI, A&HCI, CPCI-S, CPCI-SSH, ESCI, CCR-EXPANDED, IC Timespan=1950-2016 |
| --- | --- |
| # 3 | #2 AND #1  Indexes=SCI-EXPANDED, SSCI, A&HCI, CPCI-S, CPCI-SSH, ESCI, CCR-EXPANDED, IC Timespan=1950-2016 |
| # 2 | TI=(Multiple Myeloma OR myeloma OR Plasma Cell Myeloma)  Indexes=SCI-EXPANDED, SSCI, A&HCI, CPCI-S, CPCI-SSH, ESCI, CCR-EXPANDED, IC Timespan=All years |
| # 1 | TI=( circulating plasma cells OR circulating myeloma cells OR monoclonal plasma cells OR Circulating Multiple Myeloma Cells OR peripheral blood plasma cells OR CPCs OR CMCs)  Indexes=SCI-EXPANDED, SSCI, A&HCI, CPCI-S, CPCI-SSH, ESCI, CCR-EXPANDED, IC Timespan=All years |
