# Supplementary figures and images for: Prognostic value of circulating plasma cells in patients with multiple myeloma: A meta-analysis
Source: PLoS One. 2017 Jul 13;12(7):e0181447. doi: 10.1371/journal.pone.0181447 (PMC5509371; doi:10.1371/journal.pone.0181447)

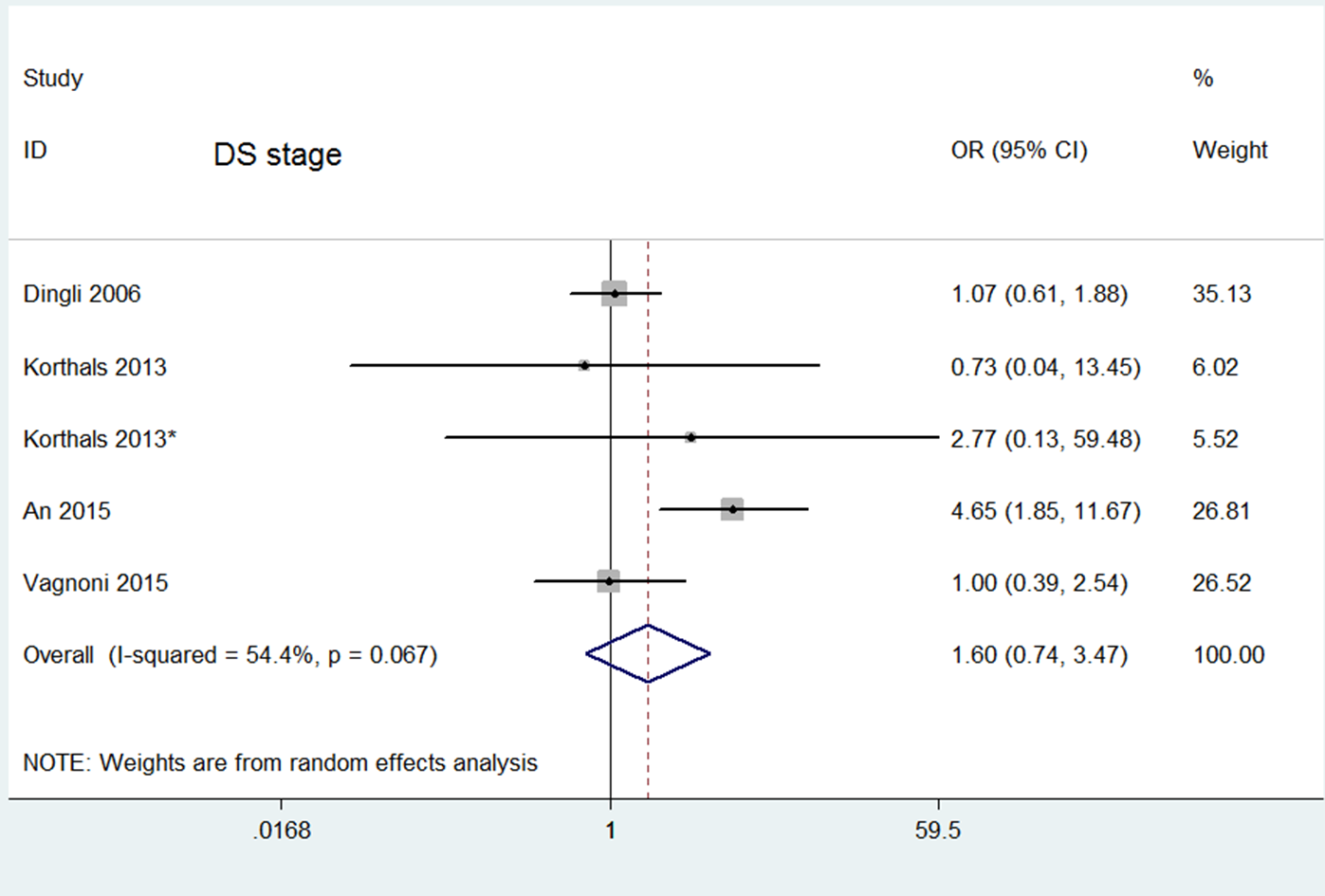

Supplement: S1 Fig — Odd ratio (OR) higher than 1 indicate that CPCs were more frequently detected in patients with increased DS stage. (TIF) [file pone.0181447.s003.tif]

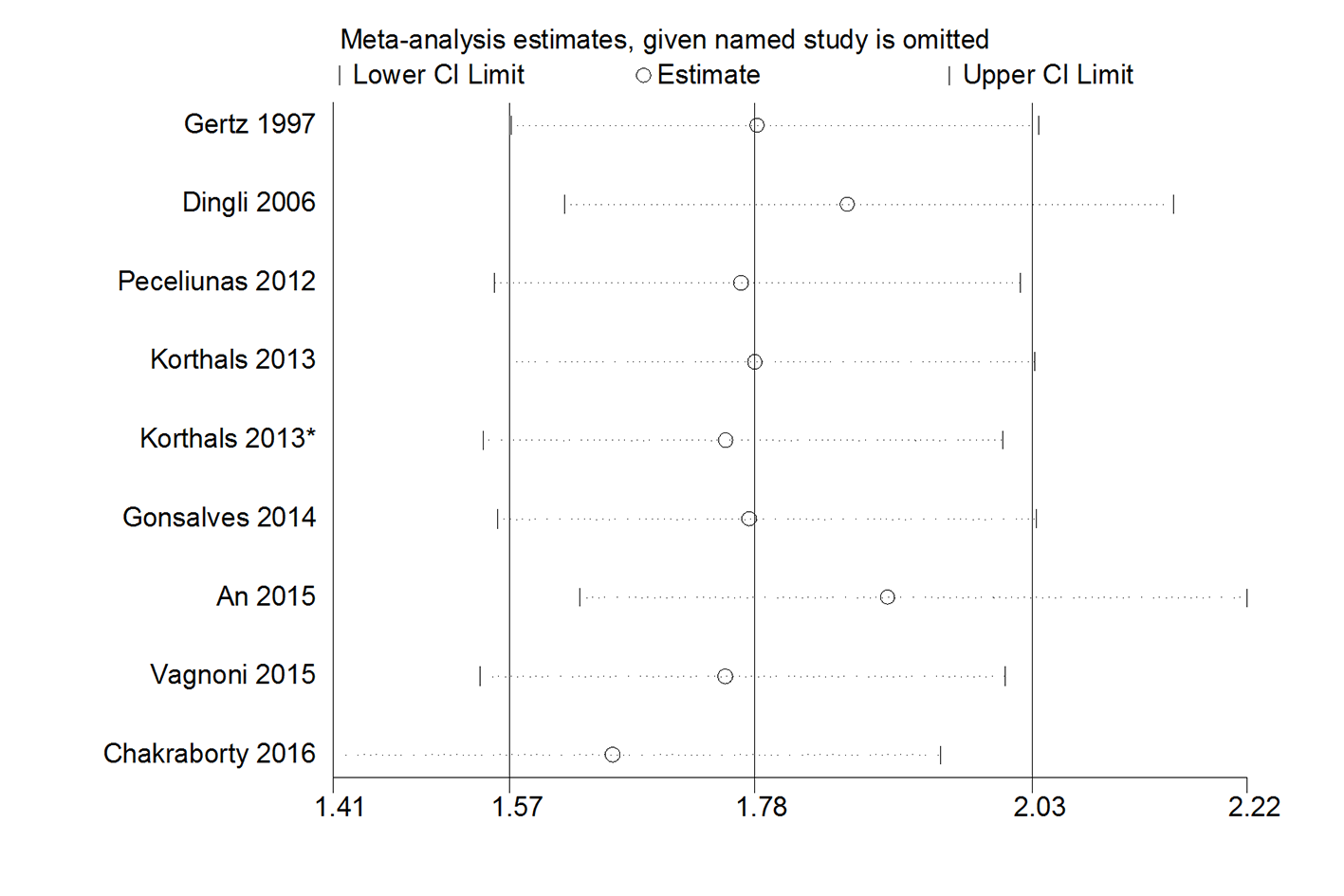

Supplement: S2 Fig — (TIF) [file pone.0181447.s004.tif]

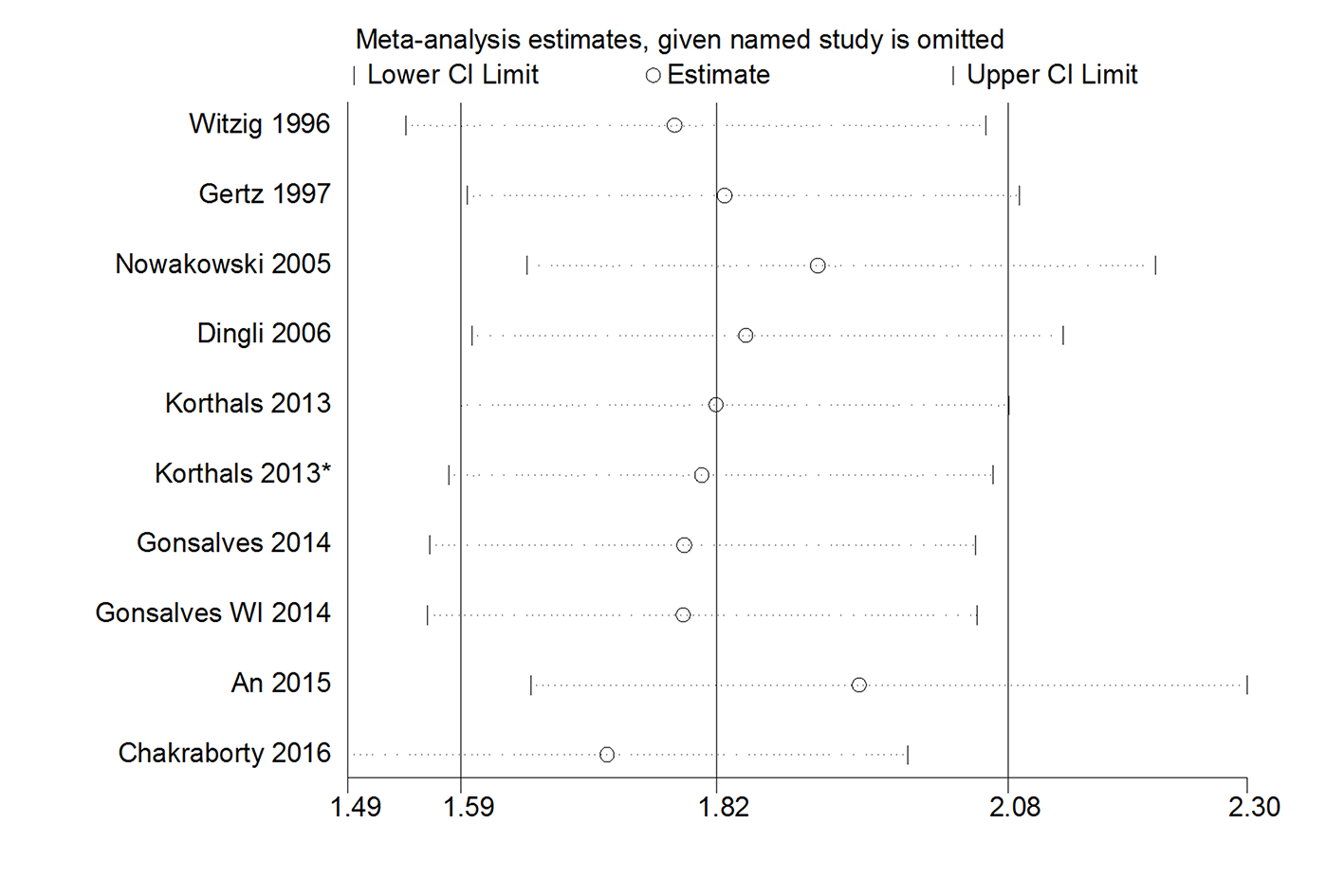

Supplement: S3 Fig — (TIF) [file pone.0181447.s005.tif]

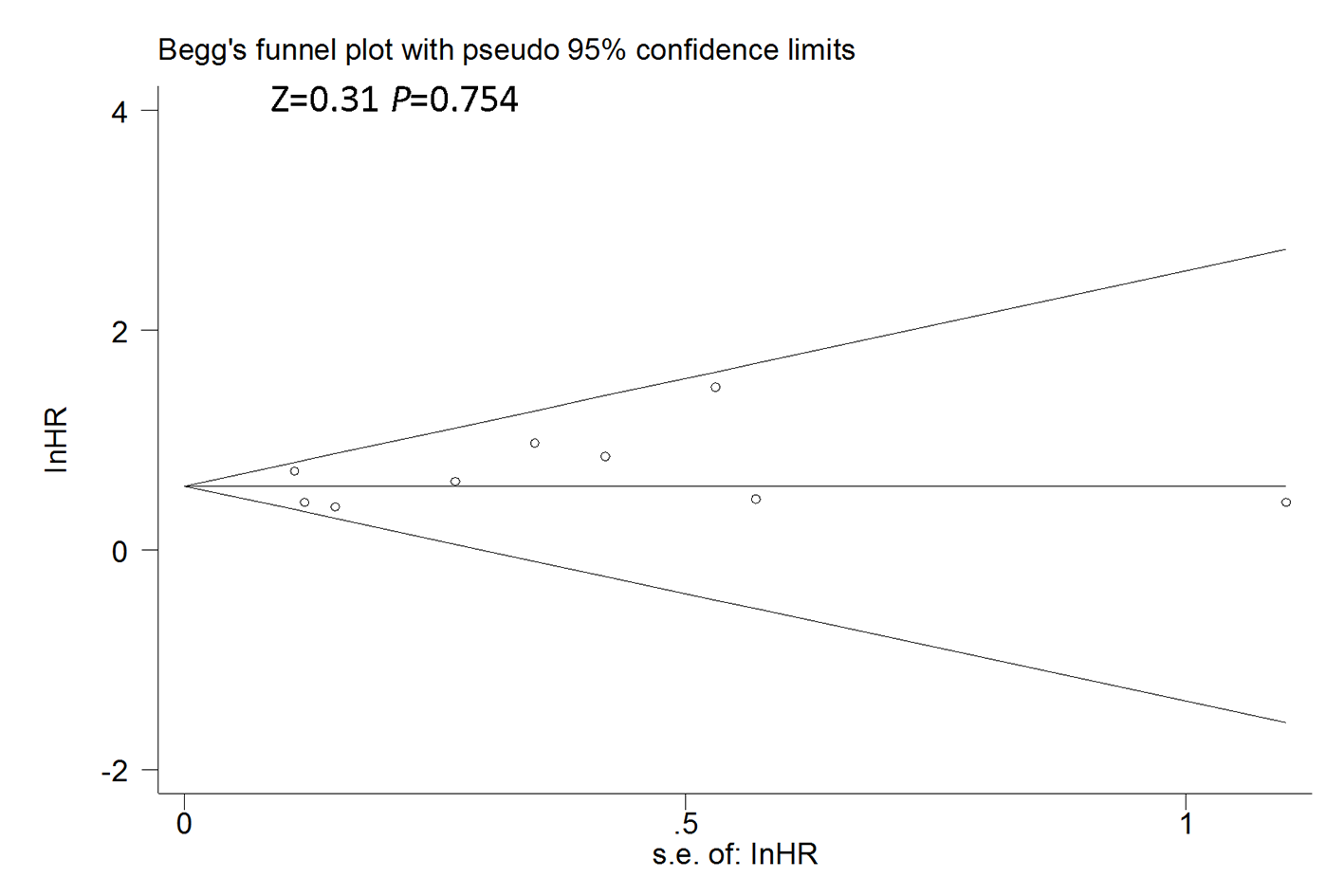

Supplement: S4 Fig — (TIF) [file pone.0181447.s006.tif]

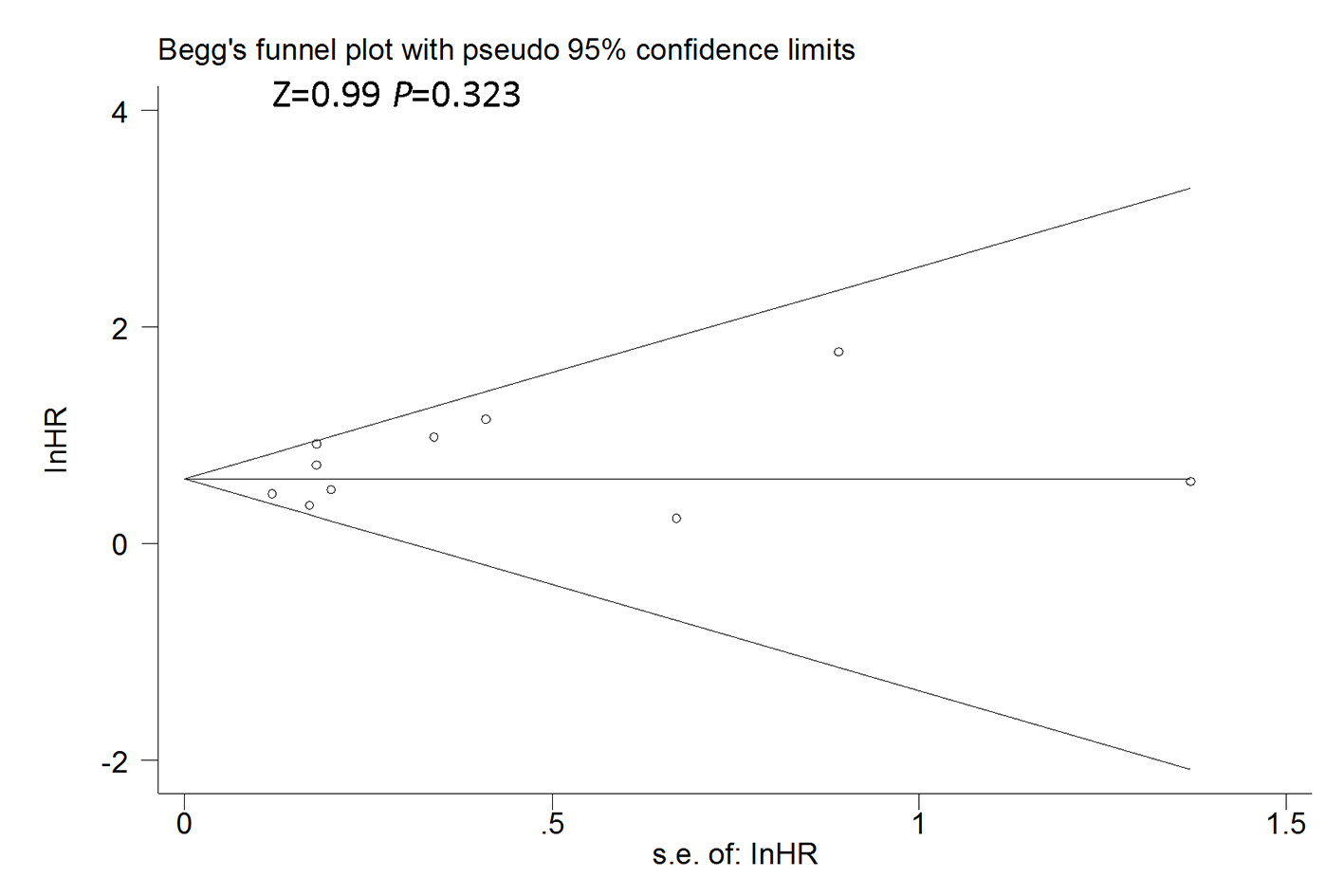

Supplement: S5 Fig — (TIF) [file pone.0181447.s007.tif]
