# Supplementary material for: Expression of Concern: Prognostic value of circulating plasma cells in patients with multiple myeloma: A meta-analysis
Source: PLoS One. 2023 Feb 21;18(2):e0282230. doi: 10.1371/journal.pone.0282230 (PMC9942954; doi:10.1371/journal.pone.0282230)
Supplement: S1 File — (ZIP) [file pone.0282230.s001.zip › primary data/excluded research/1962 Circulating Plasma Cells in Multiple Myeloma.pdf]

BRIEF COMMUNICATION

Circulating Plasma Cells in Multiple Myeloma

A Method for Detection and Review of the Problem

DONALD M. GINSBERG, M.D., *Brooklyn, New York*

SINCE BONE MARROW ASPIRATIONS and the use of electrophoretic patterns of the serum and urine have simplified the diagnosis of multiple myeloma, the search for plasma cells in peripheral blood smears is rarely attempted. It is the purpose of the paper to: [1] describe how these cells may be detected in venous blood with a simple method derived from the work of Moore, Sandberg, and Schubarg (1), and [2] to discuss the relevant literature concerning the presence of the plasma cells in peripheral smears.

MATERIAL

Eighteen patients with diagnoses of multiple myeloma and no evidence of plasma cell leukemia were studied, both with the routine peripheral smear and with the method described below (Tables 1, 2). In addition, the charts of 12 other patients with this diagnosis were reviewed, and the presence or absence of plasma cells in the routine peripheral smear was noted (Table 3).

METHOD

Ten ml of venous blood are drawn and placed in a test tube, which contains 1 mg heparin (0.1 ml of a solution containing 1,000 U.S.P. units of heparin/ml). To this sample, 25 mg of human fibrinogen (dried) are added, and the contents are inverted gently 10 times to insure adequate mixing. This mixture is then incubated at 37 C for 30 minutes. The fibrinogen will cause rapid sedimentation of the red blood cells, so that a precipitate, consisting of these cells, will form; over the precipitate is a cloudy layer of plasma containing the white blood cells (and platelets) with a minimum of red cells. This upper layer is removed with a pipette and placed in a centrifuge tube, which is then spun rapidly for 5 to 10 minutes. The white blood cells (and platelets) will form a small button on the bottom of the tube. The supernate is removed and discarded, and the small button is then removed with a syringe and a long, flat, beveled needle. The few drops thus obtained are smeared on several slides, similar to

TABLE 1. Author's Study of Patients with Previously Treated Multiple Myeloma

No. Cases	Positive for Plasma Cells	
	Routine Smear	Concentration Technique
	No. (%)	*No.* (%)
13	1 (7.7)	4 (31)

TABLE 2. Study of Patients with Multiple Myeloma before and after Treatment

	No. Cases	Positive for Plasma Cells	
		Routine Smear	Concentration Technique
		No. (%)	*No.* (%)
Before treatment	5	3 (60)	Not done
After treatment	5	0 (0)	2 (50)*

* Only 4 patients were available for this study.

Received June 20, 1962; accepted for publication July 3, 1962.

From the Departments of Medicine, Beth-El Hospital, Brooklyn, New York, and the Veterans Administration Hospital, Brooklyn, New York.

Requests for reprints should be addressed to Donald M. Ginsberg, M.D., Beth-El Hospital, Brooklyn 12, New York.

TABLE 3. Hospital Data on Patients with Previously Treated Multiple Myeloma

No. Cases	Positive for Plasma Cells	
	Routine Smear	Concentration Technique
	No. (%)	*No.* (%)
12	1 (8.3)	Not done

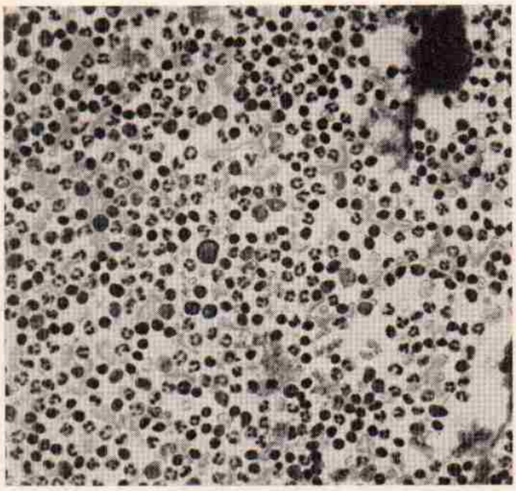

FIGURE 1. Low power view of white cells with concentration technique. There is a plasma cell in the center of the field. Original magnification, × 100.

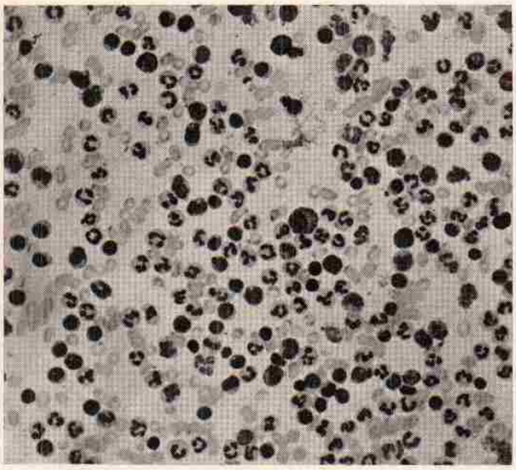

FIGURE 2. Low power view of white cell concentration with the technique described. A typical plasma cell is in the center of the field. Original magnification, × 100.

the way in which a bone marrow aspirate would be prepared. Then the slides are stained with Wright's stain. In examination of the slides, careful attention is given to the peripheral areas, since the greatest number of the plasma cells are found there.

RESULTS

Of 13 patients originally studied by this method and, concomitantly, by routine peripheral smears for comparison, 4 had smears which readily disclosed recognizable plasma cells (Figures 1–4); only one of the routine peripheral smears was positive. Peripheral smears from 5 subsequent cases of multiple myeloma were then studied, although it was known that these patients had received treatment for the disease. None of the routine peripheral smears were

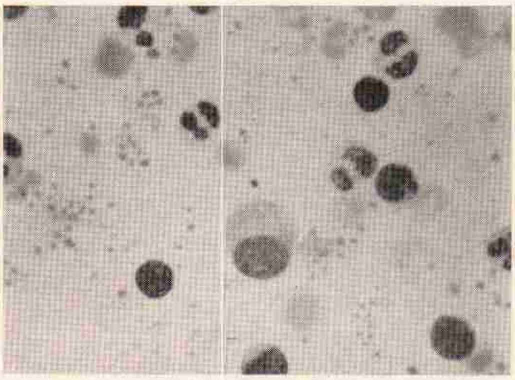

FIGURE 3. Low power view of a plasma cell (concentration technique). Original magnification, × 430.

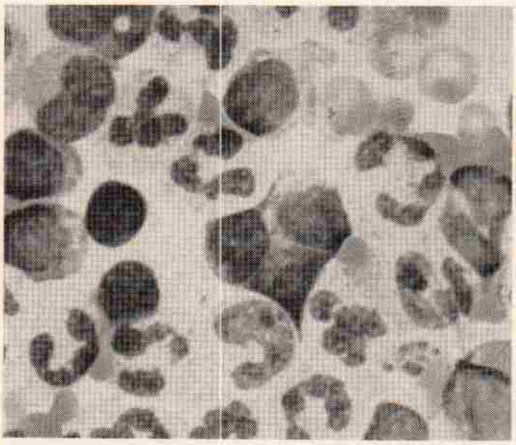

FIGURE 4. High power view with the method described, showing a trinucleated plasma cell (myeloma cell). Original magnification, × 1,000.

positive. Of these 5 patients, 4 were available for further study by the concentration technique; circulating plasma cells were demonstrable in 2 cases.

Discussion

In our studies, plasma cells were easily detected with the concentrated white blood cell layer in only 4 of 13 patients (31 per cent) with myeloma (Table 1). In contrast to these results, plasma cells were found in almost every case of multiple myeloma when smears of the buffy coat were made according to Snapper, Turner, and Moscovitz (2). Of equal interest is that 22 per cent of the 97 patients reported by these authors were found to have plasma cells in the routine peripheral blood smear, and that plasma cells were discovered under low power examination in the peripheral smears of 41 of 56 patients (73.2 per cent) described by Morissette and Watkins (3). Since we found only one positive peripheral smear (7.7 per cent) among those from the original 13 patients studied, the charts of 12 additional cases of multiple myeloma (Table 3) were reviewed; it was apparent that the routine smear demonstrated circulating plasma cells in only one case (8.3 per cent).

Since these results were found to be too difficult to reconcile, 5 other patients (Table 2) were studied, about whom information was available both from before and after the start of anti-myeloma therapy. Three of these 5 patients demonstrated plasma cells in the circulating blood by routine smear before treatment was started; none of the 5 showed circulating plasma cells after therapy. Four of these patients were available for study by the concentration technique only after therapy; plasma cells were demonstrated in 2 of the 4.

Thus, it appears that therapy directed against this disease can alter the status of the circulating plasma cells and that this factor may be responsible for the discrepancies noted. The high percentage of positive smears obtained by Morissette and Watkins (3) probably resulted from the fact that no anti-myeloma treatment was available in 1942. By 1953, when Snapper and his associates reported 22 per cent positive peripheral smears, various types of therapy were being employed in the treatment of multiple myeloma. Whether or not the disease itself was affected, the emergence of plasma cells into the peripheral blood probably was retarded. This explanation seems plausible since, in our study, 3 of 5 patients with myeloma had positive peripheral smears prior to treatment; subsequently, all were negative (Table 3). This theory also would explain the low figures of 7.7 and 8.3 per cent of patients in our study who had positive peripheral smears, as the relationship of study to treatment was difficult to ascertain. By the method outlined above, however, it was possible to discover these circulating cells in a high percentage of patients with multiple myeloma, in spite of the absence of the cells on routine smears. Only by further study with both techniques, both before and after treatment, can this question be answered completely. Until then, the use of the concentration method described is recommended. It is a relatively simple technique and results in a remarkable concentration of the white blood cells, so that various cells, not usually recognized in normal peripheral smears, are seen easily.

Summary

A new method, utilizing a concentrated layer of white blood cells for the detection of plasma cells in patients with multiple myeloma, is described. A brief discussion of the problems of detection is presented also.

Acknowledgment

I would like to thank Dr. I. Snapper, Director of Medicine and Medical Education, Beth-El Hospital, Brooklyn, New York, for his encouragement in this work.

Summario in Interlingua

Es describite un simple methodo pro le detection de plasmazellen in sanguine venose de patientes con myeloma multiple. Le methodo utilisa un concentrate strato de leucocytos. Varie studios in varie typos de patiente indica que frottis peripheric routinari non revela in multe casos le presentia de plasmazellen quando le patiente ha previemente recipite un therapia anti myeloma. Il pare que le intervention therapeutic altera le stato del circulante plasmazellen. Tamen le hic presentate nove methodo concentratori ha succedite in deteger iste cellulas in un alte procentage del patientes

in qui frottis routinari esseva negative. Le uso del nove technica es recommendate.

References

1. Moore, G. E., Sandberg, A. A., Schubarg, J. R.: Clinical and experimental observations of the occurrence and fate of tumor cells in the blood stream. *Ann. Surg.* 146: 580, 1957.
2. Snapper, I., Turner, L. B., Moscovitz, H. L.: *Multiple Myeloma,* Grune & Stratton, Inc., New York, 1953.
3. Morissette, L., Watkins, C. H.: Multiple myeloma: diagnostic value of the blood smear. *Proc. Mayo Clin.* 17: 433, 1946.

Copyright © 2002 EBSCO Publishing
