# Supplementary material for: Expression of Concern: Prognostic value of circulating plasma cells in patients with multiple myeloma: A meta-analysis
Source: PLoS One. 2023 Feb 21;18(2):e0282230. doi: 10.1371/journal.pone.0282230 (PMC9942954; doi:10.1371/journal.pone.0282230)
Supplement: S1 File — (ZIP) [file pone.0282230.s001.zip › primary data/excluded research/1993 Quantitation of circulating peripheral blood plasma cells and their relationship to disease activity in patients with multiple myeloma.pdf]

Quantitation of Circulating Peripheral Blood Plasma Cells and Their Relationship to Disease Activity in Patients with Multiple Myeloma

Thomas E. Witzig, M.D., Madhav V. Dhodapkar, M.D., Robert A. Kyle, M.D., and Philip R. Greipp, M.D.

Background. **The analysis of peripheral blood mononuclear cells for plasma cells and B-cell surface light chain ratios may provide additional insight on the pathogenesis of multiple myeloma and predict disease activity. The goals of this study were to correlate these parameters with clinically determined disease activity and establish a cutoff value of circulating plasma cells that could be used in future clinical trials.**

Methods. **Peripheral blood samples from 84 patients with monoclonal gammopathies were analyzed by an immunofluorescence, slide-based, labeling index (LI) technique for detection of plasma cells, the LI, and light chain ratios. These parameters were compared with disease activity.**

Results. **Of the 84 patients studied, 27% had inactive and 73% had active disease. The mean number of plasma cells for patients with inactive disease was $0.61 \times 10^6/l$, compared with $139.6 \times 10^6/l$ for patients with active disease ($P < 0.001$). The mean ratios of involved to uninvolved light chain percentages for patients with inactive and active disease were 1.6 and 9.2, respectively ($P = 0.002$). The absolute number of plasma cells better predicted disease activity than the light chain ratio. By use of a cutoff value of $3 \times 10^6/l$, 67% of patients with active disease were determined to have $3 \times 10^6/l$ or more plasma cells, and 96% of those with inactive disease had less than $3 \times 10^6/l$ plasma cells.**

Conclusions. **These results suggest that detection of circulating plasma cells is a marker of disease activity in patients with plasma cell disorders and that an appropriate cutoff value is $3 \times 10^6/l$ or more circulating plasma cells. *Cancer* 1993; 72:108–13.**

Key words: multiple myeloma, cell kinetics, plasma cells.

The peripheral blood compartment typically is not examined closely for the presence of plasma cells in the routine evaluation of patients with plasma cell proliferative disorders. Circulating plasma cells usually are not detected on a Wright-stained peripheral blood smear until a time in the disease course when it is not difficult to determine disease activity by other tests; however, malignant cells can be found in the peripheral blood of patients with active disease earlier in the disease course with immunofluorescence techniques,[1] DNA content flow cytometry,[2–4] or detection of B-cell gene rearrangements.[5–11] Several investigators previously have described in patients with inactive disease the suppression of peripheral blood B-lymphocytes bearing the same surface light chain as the patient's serum or urine monoclonal immunoglobulin (Ig) light chain.[12–14] This suppression is referred to as light chain isotype suppression (LCIS), and the loss of LCIS may serve as an indicator of active disease.

Our hypothesis was that patients with active disease would be more likely to have circulating monoclonal plasma cells than those with inactive disease. To study this issue, we examined peripheral blood from 84 patients with monoclonal gammopathies by use of an immunofluorescence technique to detect circulating plasma cells and determine the B-cell light chain isotype ratio. We wanted to compare these parameters with clinically determined disease activity to learn whether there was any correlation with disease activity and to establish a cutoff value of circulating plasma cells that could be used in future clinical trials.

Materials and Methods

Patient Selection

The clinical and laboratory data from 84 patients with monoclonal gammopathies in whom the peripheral blood was analyzed for circulating plasma cells and B-lymphocyte light chain ratios were reviewed. These patients had been examined at our institution between December 1984 and April 1989 and had a minimum

From the Division of Internal Medicine and Hematology, Mayo Clinic and Mayo Foundation, Rochester, Minnesota.

Supported in part by a Career Development Award from the American Cancer Society (T.E.W.).

Address for reprints: Thomas E. Witzig, M.D., 920E Hilton Building, Mayo Clinic, Rochester, MN 55905.

Accepted for publication February 11, 1993.

follow-up of 1 year. All studies on patients with newly diagnosed multiple myeloma (MM) were performed before the initiation of chemotherapy. The definitions of monoclonal gammopathy of undetermined significance (MGUS), smoldering MM (SMM), new MM, and relapsed MM were as previously described.[15] Briefly, MGUS and SMM were considered inactive disease. Patients with MGUS had a serum monoclonal protein (M-protein) level less than 3 g/dl, less than 10% plasma cells in the bone marrow (if examined), and no other evidence of MM (i.e., bone lesions, anemia, renal insufficiency, or hypercalcemia). Patients with SMM had a serum M-protein level greater than 3 g/dl or a serum M-protein and greater than 10% bone marrow plasma cells and no other clinical or laboratory evidence of overt MM. The diagnosis of SMM in this study was retrospective in that all patients had to have stable disease for at least 1 year. This was required so that the peripheral blood analysis for plasma cells and light chain ratios could be tested in patients who had true, proven SMM.

Patients with new MM and relapsed MM were considered to have active disease. Patients with new MM had clinical findings consistent with the diagnosis, a serum or urine M-protein, more than 10% bone marrow plasma cells, and skeletal osteoporosis or lytic lesions. Patients without skeletal lesions were required to have more than 30% plasma cells in the bone marrow to be classified as having new MM. Patients with relapsed MM had recurrence or progression of features of active MM after chemotherapy requiring a change in therapy or resulting in death from progressive disease.

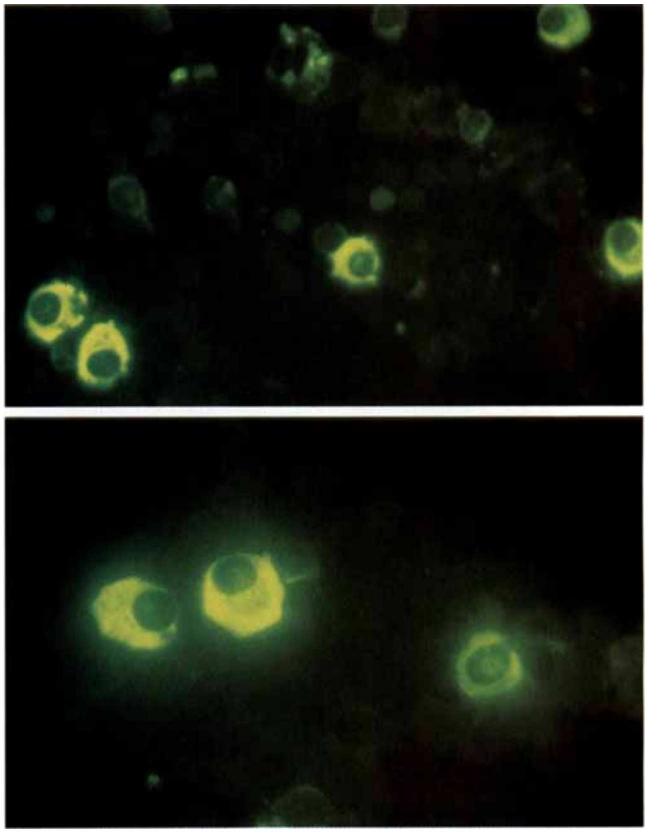

Figure 1. Cytospin slides of peripheral blood monoclonal plasma cells stained with anti–light chain antibody conjugated to FITC, detected in a patient with relapsed multiple myeloma (top, original magnification ×400; bottom, original magnification ×630).

Peripheral Blood Analysis

The number of circulating plasma cells, light chain isotype ratios, and plasma cell labeling index (LI) were obtained by use of a peripheral blood LI technique as previously described.[1] In brief, mononuclear cells were isolated on Ficoll-Hypaque, and 1.0×10^6 cells then were incubated for 1 hour at 37°C in RPMI-1640 containing 10 μmol/l bromodeoxyuridine, 1 μmol/l fluorodeoxyuridine, 10% fetal calf serum, and antibiotics. The cells were washed once with phosphate-buffered saline at pH 7.4, cytocentrifuge slides were made, and the slides were air-dried and then fixed for 10 minutes in 95% ethanol. Twenty micrograms of BU-1 monoclonal antibody (Amersham Corporation, Arlington Heights, IL) was added to the cell spot, incubated for 30 minutes at room temperature, washed in phosphate-buffered saline/0.5% Tween 80 (Difco, Detroit, MI), and air-dried. Eight micrograms of goat anti-mouse IgG labeled with rhodamine isothiocyanate was added to detect BU-1 antibody; simultaneously, lymphocytes and plasma cells were identified by the addition of monospecific anti-kappa and anti-lambda reagent labeled with fluorescein isothiocyanate (Tago, Burlingame, CA). Before the study, the anti-kappa and anti-lambda reagents were tested for specificity and lack of cross-reactivity to cells of the opposite light chain type.

After the fluoresceinated anti–light chain reagents were added, the slides were incubated a second time for 30 minutes, washed in phosphate-buffered saline/Tween, air-dried, and read in an epi-illumination fluorescence microscope (Zeiss, Thornwood, NY) with fluorescein isothiocyanate and rhodamine isothiocyanate filters and an HBO 100-W light source. Abnormal plasma cells had characteristic morphologic features (eccentric nucleus and abundant cytoplasm) and monoclonal cytoplasmic light chain staining (Fig. 1). Whenever plasma cells were detected, we carefully evaluated the slide stained with the opposite light chain to confirm the monoclonality of the plasma cells.

The number of circulating plasma cells $\times 10^6$/l was calculated as follows: no. plasma cells = % plasma cells

$\times$ % Ig+ cells $\times$ % mononuclear cells $\times$ leukocytes, where the % Ig+ cells was the number of B-lymphocytes and plasma cells staining with the fluorescein isothiocyanate anti-light chain reagent corresponding to the known M-protein of the patient per 100 nucleated cells; the % plasma cells was the number of plasma cells/100 Ig+ cells; the % mononuclear cells was the percentage of lymphocytes plus the percentage of monocytes determined on the leukocyte differential performed on the day of the peripheral blood studies; and leukocytes represented the leukocyte count $\times 10^9$/l.

The light chain ratio was calculated as follows: light chain ratio = % Ig+ cells "involved"/% Ig+ cells "uninvolved," where involved refers to the light chain isotype of the known M-protein of the patient.

For the calculation of the LI, at least 500 cells with positive staining for the same light chain isotype as the M-protein of the patient were counted to determine an immunofluorescence LI.

Data Analysis

The chart of each patient was reviewed without the reviewer being aware of the peripheral blood data, and the patient was classified as having either inactive (MGUS, SMM) or active (new MM, relapsed MM) disease. The number of circulating plasma cells $\times 10^6$/l, percentage of plasma cells, LI, and light chain ratios for the patients in the inactive and active disease groups were compared by use of the Mann–Whitney unpaired *t* test. These four parameters also were compared between the groups with new MM and relapsed MM.

Results

Of the 84 patients studied, 27% (23 of 84) had inactive disease and 73% (61 of 84) active disease. These cases could be classified further as 12% (10 of 84) MGUS, 15% (13 of 84) SMM, 42% (35 of 84) new MM, and 31% (26 of 84) relapsed MM.

The median number of circulating plasma cells for inactive cases was 0.0 $\times 10^6$/l (mean, 0.6 $\times 10^6$/l; range, 0–4 $\times 10^6$/l) compared with 6.6 $\times 10^6$/l (mean, 139.6 $\times 10^6$/l; range, 0–3186.7 $\times 10^6$/l) for active cases ($P < 0.001$) (Table 1). Within the group of active cases, the median number of circulating plasma cells for new MM was 5.4 $\times 10^6$/l (mean, 210.5 $\times 10^6$/l; range, 0–3186.7 $\times 10^6$/l) compared with 11.6 (mean, 44.1 $\times 10^6$/l; range, 0–373.2 $\times 10^6$/l) ($P = 0.039$) for relapsed MM. After examination of the data, a value of 3 $\times 10^6$/l or more circulating plasma cells was chosen as a cutoff between active and inactive disease (Fig. 2). By use of this value, 96% (22 of 23) of those with inactive disease had less than 3 $\times 10^6$/l and 67% (41 of 61) of patients with active disease had 3 $\times 10^6$/l or more circulating plasma cells.

There was also a statistically significant difference ($P < 0.001$) between the inactive and active cases for the percentage of plasma cells (as a percentage of the Ig+ cells) (Table 1; Fig. 3). This value can be determined without knowledge of the leukocyte count or leukocyte differential. When a cutoff value of more than 3% plasma cells was used, 96% (22 of 23) of inactive cases had 3% or less and 66% (40 of 61) of active cases had more than 3% plasma cells and therefore were classified correctly.

The LI data are summarized in Table 1. By use of a previously established LI cutoff value of 0.5% or greater,[1] 67% (41 of 61) of patients with active disease were identified; all 22 patients with inactive disease had an LI of less than 0.5%. Six of the 20 patients with active disease but less than 3 $\times 10^6$ circulating plasma cells per liter had either an LI of 0.5% or greater or more than 3% plasma cells. Therefore, if the criterion for active disease was the presence of any one of these three criteria (i.e. $\geq 3 \times 10^6$ plasma cells/l, $>$ 3% plasma cells, or an LI of $\geq$ 0.5%), then 77% (47 of 61) of the active cases and 96% (22 of 23) of the inactive cases were classified correctly.

Among the 61 patients with active disease, a separate analysis of the 35 patients with new MM and 26 patients with relapsed MM was performed (Table 1). We found statistically significant differences between the groups with new MM and relapsed MM for each parameter tested. In the group with new MM, 57% (20 of 35) had 3 $\times 10^6$/l or more circulating plasma cells, 57% (20 of 35) had more than 3% plasma cells, 49% (17 of 35) had an LI of 0.5% or greater, and 63% (22 of 35) had at least one of these parameters indicative of active disease. When the group with relapsed MM was examined, 81% (21 of 26) had 3 $\times 10^6$/l or more circulating plasma cells, 77% (20 of 26) had more than 3% plasma cells, 92% (24 of 26) had an LI of 0.5% or greater, and 96% (25 of 26) had at least one of these parameters indicative of active disease.

The median light chain isotype ratio for inactive cases was 0.8 (mean, 1.6; range, 0.2–5.8) compared with 2.2 (mean, 9.2; range, 0.3–78.6) for active cases ($P = 0.002$). Patients with relapsed MM had a median light chain ratio of 4.6, compared with 1.6 for those with new MM ($P = 0.002$). Figure 4 depicts the light chain ratios in the groups with inactive and active disease. Although the difference between the two groups is significant ($P = 0.002$), it is evident that there is considerable overlap between the two groups, making it difficult to define a cutoff value that separates the two groups.

Table 1. Results of Peripheral Blood Studies by Disease Category

	Inactive disease (N = 23)	Active disease (N = 61)	*P* value	New myeloma (N = 35)	Relapsed myeloma (N = 26)	*P* value
No. plasma cells*						
Mean	0.61	139.6	< 0.001	210.5	44.1	0.039
Median	0	6.6		5.4	11.6	
Range	0–4.0	0–3186.7		0–3186.7	0–373.2	
Percentage of cases with ≥ 3 × 10^6/l	4	67		57	81	
Light chain ratio						
Mean	1.63	9.2	0.002	5.3	14.5	0.002
Median	0.8	2.2		1.6	4.6	
Range	0.2–5.8	0.3–78.6		0.3–78.6	0.3–74	
Percentage of plasma cells†						
Mean	0.67	18.6	< 0.001	15.5	22.9	0.025
Median	0	9.0		4.5	23	
Range	0–5	0–86		0–86	0–76	
Percentage of cases with > 3%	4	66		57	77	
Labeling index						
Mean	0.08	2.4	< 0.001	2.3	2.6	0.006
Median	0	1.0		0.4	1.3	
Range	0–0.4	0–15.9		0–14.6	0.2–15.9	
Percentage of cases with ≥ 0.5%	0	67		49	92	

* Number of circulating plasma cells × 10^6/l.
† Plasma cells as a percentage of cytoplasmic immunoglobulin-positive lymphocytes.

Discussion

In current clinical practice, the distinction between active and inactive MM usually is made with simple blood tests, measurements of serum and urine monoclonal protein, skeletal radiographs, and a bone marrow examination with measurement of plasma cell proliferation.[16–19] It has become apparent that quantitative studies of peripheral blood lymphocytes and circulating plasma cells may add useful information about disease activity.[1,3,12–14,20,21] This study demonstrates that circu-

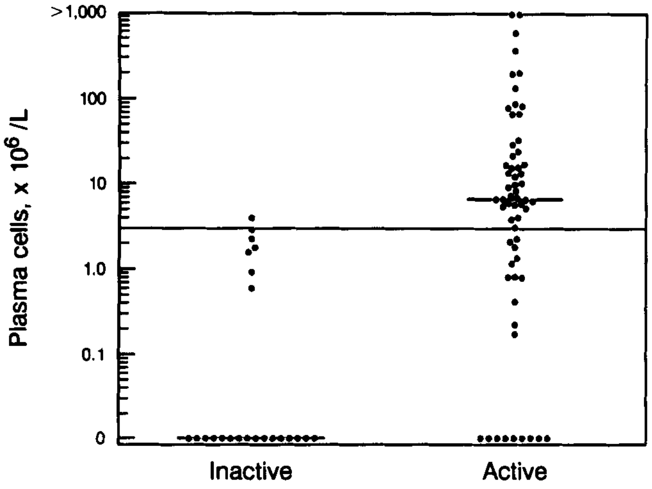

Figure 2. Absolute number of circulating plasma cells × 10^6/l in each disease category. The horizontal line at 3 × 10^6/l is the cutoff value between a low and high number of circulating plasma cells; the short horizontal lines represent median values.

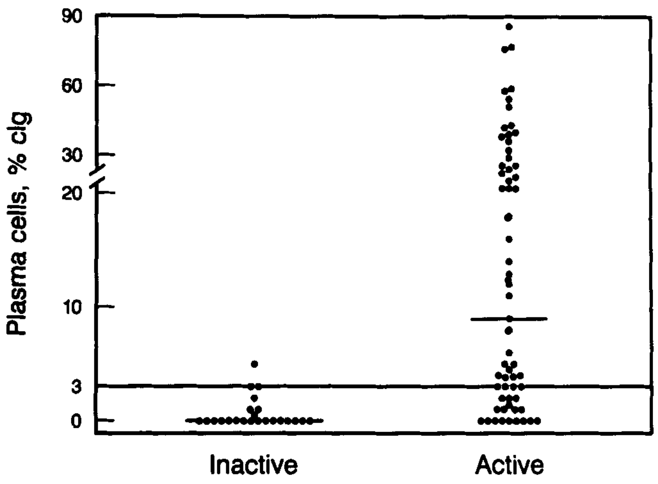

Figure 3. Plasma cells (as a percentage of cytoplasmic Ig-positive lymphocytes) in each disease activity group. The horizontal line at 3% is the cutoff value between a low and high value; the short horizontal lines represent median values.

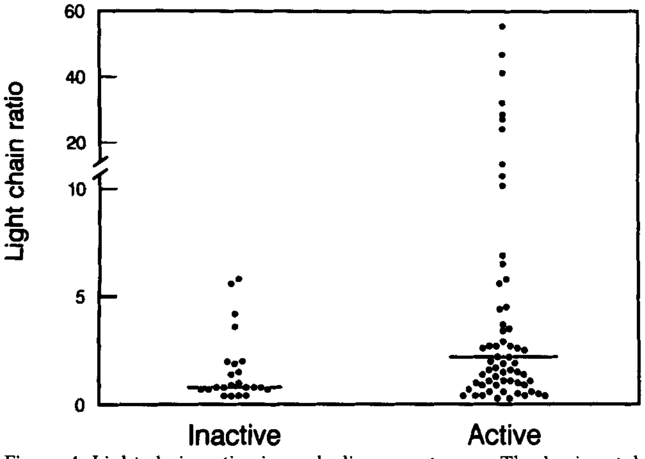

Figure 4. Light chain ratios in each disease category. The horizontal lines represent median values.

lating plasma cells can be quantitated and the presence of 3 × 10^6/l or more plasma cells suggests active disease. The exact mechanism and the factors influencing this important circulatory phase of MM are not clear. The loss of LCIS is also helpful, but in this study the number of circulating plasma cells was a better discriminator of disease activity (Figs. 2 and 3).

Other investigators have shown circulating malignant cells by use of molecular genetic techniques.[5-11] Ig gene rearrangements have been demonstrated from peripheral blood cells in patients with documented circulating plasma cells[9,10] or increased numbers of lymphoid cells bearing the same cytoplasmic Ig light chain as the M-protein.[5] Billadeau et al.[7] recently described the use of allele-specific oligonucleotides in a polymerase chain reaction technique to detect malignant peripheral blood cells in 13 of 14 previously untreated patients with MM. The immunofluorescence method used in our study permits direct identification of the plasma cells by their characteristic morphologic features and monoclonal light chain cytoplasmic Ig staining (Fig. 1). It would appear that our technique will complement the molecular genetic techniques by providing better confirmation of the morphologic features of the cells responsible for the B-cell gene rearrangements. We recently have simplified the detection process by depleting the T-cells from the peripheral blood sample using magnetic beads coated with anti-CD2 monoclonal antibody.[22]

Leonard et al.[23] first provided evidence for LCIS of plasma cells in studies of plasma cells in the lamina propria of the gut in patients with MM. Subsequent studies have described the phenomenon of LCIS as a marker for plateau stage disease.[12-14] Patients with newly diagnosed MM and LCIS have a better prognosis than similar patients without LCIS,[20] and progressive disease is associated with the loss of LCIS. Although the exact mechanism of LCIS is not clear, it is thought to be a manifestation of B-cell immunoregulation and host-tumor interaction. Our studies also show that patients with inactive disease tend to have lower light chain ratios than patients with active disease, although there is considerable overlap between the groups (Fig. 4). It should be mentioned that Wearne et al.[12,13] and Joshua et al.[14,20] used an indirect immunofluorescence assay and flow cytometric analysis to quantitate surface membrane light chain isotypes on peripheral blood B-lymphocytes. Our technique differs in that it is slide based and relies on identification of cells by fluorescence microscopic examination. With this technique, it is sometimes difficult to differentiate surface from cytoplasmic Ig staining on small lymphocytes; therefore, it is likely that many of the cells we are identifying may have primarily cytoplasmic Ig or both surface and cytoplasmic Ig light chain. Some of these cells may not have been counted as positive in a flow cytometric assay.

The detection of circulating cells in the peripheral blood that express the CD38 antigen by use of a flow cytometric technique also has been shown to correlate with active disease.[14,24] Because this antigen is expressed on both terminally differentiated B-lymphocytes and activated T-lymphocytes,[25] it is likely that the CD38+ cells detected in patients with active myeloma are circulating plasma cells and activated T-lymphocytes. In the study by Omede et al.,[24] patients with active disease had more than 450 × 10^6/l circulating CD38+ cells in their peripheral blood, whereas in our study 3 × 10^6/l or more circulating plasma cells indicated active disease. This discrepancy probably results from the presence of the activated T-lymphocytes that also express CD38 and the fact that our method was slide based, whereas they used flow cytometry.

This study suggests that circulating plasma cells and light chain isotype ratios may serve as markers for disease activity in patients with plasma cell proliferative disorders. These assays can be performed easily in the clinical laboratory on a small sample of peripheral blood. By use of the method described in this article, the number of circulating plasma cells, the LI, and the light chain ratio can be determined from one sample. Because some patients will not receive a diagnosis of active disease based on these tests alone, peripheral blood studies should be used in conjunction with other diagnostic tests for optimal care of the patient.

What is the current utility of the detection of circulating plasma cells? These tests may be most useful in patients who appear to have SMM or MGUS, after usual diagnostic testing.[26] If no circulating cells are detected, then this encouraging result may help in the decision to observe the patient without administration

of chemotherapy. Patients who appear clinically to have SMM but have circulating plasma cells will require clinical follow-up to determine whether they have a different clinical outcome. Another current application involves the examination of peripheral stem cell harvests to be used for marrow reconstitution of patients with MM undergoing autologous transplantation. The assay for circulating plasma cells described in this study also has research applications. It should be used along with molecular genetic studies of peripheral blood cells to help resolve the question of the immunophenotype and morphologic features of circulating precursor myeloma cells. It is clear that long-term clinical studies in patients with new MM are needed to determine the independent prognostic value of the presence or absence of circulating plasma cells. These studies will be performed best in the context of cooperative group treatment trials.

References

1. Witzig T, Gonchoroff N, Katzmann J, Therneau T, Kyle R, Greipp P. Peripheral blood B-cell labeling indices are a measure of disease activity in patients with monoclonal gammopathies. *J Clin Oncol* 1988; 6:1041–6.
2. Barlogie B, Latreille J, Alexanian R, Swartzendruber D, Smallwood L, Maddox A-M, et al. Quantitative cytology in myeloma research. *Clin Haematol* 1982; 11:19–46.
3. Morgan R, Witzig T, Gonchoroff N, Kyle R. Circulating peripheral blood cells in multiple myeloma: an important overlooked compartment [abstract]? *Proc Am Assoc Cancer Res* 1987; 28:140.
4. Shimazaki C, Gotoh H, Oku N, Inaba T, Murakami S, Oku N, et al. Detection of minimal residual myeloma cells by dual parameter analysis of DNA and cytoplasmic immunoglobulin. *Acta Haematol (Basel)* 1991; 85:20–5.
5. Baldini L, Cro L, Delia D, Chiorboli O, Neri A, Maiolo A. Analysis of tumor-specific immunoglobulin gene rearrangement in peripheral blood B-cells of multiple myeloma patients. *Am J Hematol* 1991; 37:1–5.
6. Berenson J, Wong R, Kim K, Brown N, Lichtenstein A. Evidence for peripheral blood B lymphocyte but not T lymphocyte involvement in multiple myeloma. *Blood* 1987; 70:1550–3.
7. Billadeau D, Quam L, Thomas W, Kay N, Greipp P, Kyle R, et al. Detection and quantitation of malignant cells in the peripheral blood of multiple myeloma patients. *Blood* 1992; 80:1818–24.
8. Cassel A, Leibovitz N, Hornstein L, Quitt M, Aghai E. Evidence for the existence of circulating monoclonal B-lymphocytes in multiple myeloma patients. *Exp Hematol* 1990; 18:1171–3.
9. Clofent G, Klein B, Commes T, Ghanem N, Lefranc M, Bataille R. No detectable malignant B-cells in the peripheral blood of patients with multiple myeloma. *Br J Haematol* 1989; 71:357–61.
10. Levy Y, Schmitt C, Tsapis A, Brouet J, Fermand J. Phenotype and immunoglobulin gene configuration of blood B cells from patients with multiple myeloma. *Clin Exp Immunol* 1991; 84:435–9.
11. Van Riet I, Heirman C, Lacor P, De Waele M, Thielemans K, Van Camp B. Detection of monoclonal B lymphocytes in bone marrow and peripheral blood of multiple myeloma patients by immunoglobulin gene rearrangement studies. *Br J Haematol* 1989; 73:289–95.
12. Wearne A, Joshua D, Kronenberg H. Light chain isotype associated suppression of surface immunoglobulin expression on peripheral blood lymphocytes in myeloma during plateau phase. *Br J Haematol* 1984; 58:483–9.
13. Wearne A, Joshua D, Young G, Kronenberg H. Multiple myeloma: light chain isotype suppression: a marker of stable disease at presentation. *Eur J Haematol* 1987; 38:43–9.
14. Joshua D, Ioannidis R, Brown R, Francis S, Gibson J, Kronenberg H. Multiple myeloma: relationship between light chain isotype suppression, labeling index of plasma cells and CD38 expression on peripheral blood lymphocytes. *Am J Hematol* 1988; 29:5–11.
15. Greipp P. Monoclonal gammopathies: new approaches to clinical problems in diagnosis and prognosis. *Blood Rev* 1989; 3:222–36.
16. Greipp P, Katzmann J, O'Fallon W, Kyle R. Value of $\beta 2$ microglobulin level and plasma cell labeling indices as prognostic factors in patients with newly diagnosed myeloma. *Blood* 1988; 72:219–23.
17. Greipp P, Witzig T, Gonchoroff N. Immunofluorescence labeling indices in multiple myeloma and related monoclonal gammopathies. *Mayo Clin Proc* 1989; 62:969–77.
18. Kyle R, Greipp P. Smoldering multiple myeloma. *N Engl J Med* 1980; 302:1347–9.
19. Latreille J, Barlogie B, Johnson D. Ploidy and proliferative characteristics in monoclonal gammopathies. *Blood* 1982; 59:43–51.
20. Joshua D, Wearne A, Kronenberg H. Immunoregulation and prognosis in myeloma. *Lancet* 1987; 1:251–3.
21. Pettersson D, Mellstedt H, Holm G. Monoclonal B lymphocytes in multiple myeloma. *Scand J Immunol* 1980; 12:375–82.
22. Witzig T, Gonchoroff N, Ahmann G, Katzmann J, Greipp P. T-cell depletion using anti-CD2 coated magnetic beads simplifies the detection of peripheral blood plasma cells. *J Immunol Methods* 1991; 144:253–6.
23. Leonard R, Maclennan I, Smart Y, Vanhegan R, Cuzik J. Light chain isotype associated suppression of normal plasma cell numbers in patients with multiple myeloma. *Int J Cancer* 1979; 24:385–93.
24. Omede P, Boccadoro M, Gallone G, Frieri R, Battaglio S, Redoglia V, et al. Multiple myeloma: increased circulating lymphocytes carrying plasma cell–associated antigens as an indicator of poor survival. *Blood* 1990; 76:1375–9.
25. Anderson K, Park E, Bates M, Leonard R, Hardy R, Schlossman S, et al. Antigens on human plasma cells identified by monoclonal antibodies. *J Immunol* 1983; 130:1132.
26. Witzig T, Garton J, Gertz M, Kyle R, Lust J, Greipp P. The number of peripheral blood plasma cells distinguishes smoldering from active multiple myeloma [abstract]. *Proc Am Soc Clin Oncol* 1992; 11:360.
