# Supplementary material for: Expression of Concern: Prognostic value of circulating plasma cells in patients with multiple myeloma: A meta-analysis
Source: PLoS One. 2023 Feb 21;18(2):e0282230. doi: 10.1371/journal.pone.0282230 (PMC9942954; doi:10.1371/journal.pone.0282230)
Supplement: S1 File — (ZIP) [file pone.0282230.s001.zip › primary data/excluded research/1994 Detection of monoclonal plasma cells in the peripheral blood stem cell harvests of patients with multiple myeloma-disease phase.pdf]

British Journal of Haematology, 1995, **89**, 640–642

SHORT REPORT

Detection of monoclonal plasma cells in the peripheral blood stem cell harvests of patients with multiple myeloma

THOMAS E. WITZIG, MORIE A. GERTZ, ALVARO A. PINEDA,* ROBERT A. KYLE AND PHILIP R. GREIPP
*Division of Internal Medicine and Hematology, and *Division of Transfusion Medicine, Mayo Clinic and Mayo Foundation, Rochester, Minnesota, U.S.A.*

Received 30 November 1994; accepted for publication 9 December 1994

Summary. We evaluated the harvest product from 47 patients undergoing peripheral blood (BP) stem cell collections for monoclonal plasma cells (PC) using a sensitive immunofluorescence technique. 60% (28/47) had documented tumour cells in the apheresis product. The 32 patients in plateau had a mean of $1 \cdot 62 \times 10^6$ PC/l *v* $74 \cdot 64 \times 10^6$ in 15 relapsed patients ($P < 0 \cdot 01$). 32% (6/19) of patients without any tumour cells in the initial sample had them detected on a subsequent apheresis sample. In four cases (all treated with GM-CSF) small numbers of tumour cells were detected initially but became undetectable on a subsequent sample.

Keywords: multiple myeloma, stem cells, autologous transplant.

High-dose therapy with stem cell rescue is an effective therapy for selected patients with multiple myeloma (MM). A frequent source of stem cells is the peripheral blood (PB). Malignant cells can be documented in small numbers in the PB of some patients with MM by various techniques (Bakkus *et al*, 1994; Berenson *et al*, 1987; Billadeau *et al*, 1992; Fend *et al*, 1993; Witzig *et al*, 1988, 1993).

The aims of this study were to prospectively quantitate the malignant plasma cells in the apheresis products from patients undergoing PB stem cell harvests, to correlate these measurements with the patient's clinical status, and to study serial apheresis samples.

PATIENTS AND METHODS

Patient selection. Patients with MM undergoing PB stem cell harvests between May 1989 and January 1994 were candidates for this Institutional Review Board approved study. After November 1991, patients received GM-CSF $5\,\mu g/kg$ s.q. daily 2 d before harvest and daily until the harvest was complete. All patients had been previously treated; however, none received chemotherapy at the time of harvest to mobilize stem cells.

Sample collection. The PB mononuclear cells examined for monoclonal plasma cells were an aliquot of the apheresis product.

Correspondence: Dr T. E. Witzig, 920E Hilton Bldg, Mayo Clinic, Rochester, MN 55905, U.S.A.

Peripheral blood plasma cell quantitation and labelling index methods. The number of monoclonal plasma cells and the PB plasma cell labelling index (PBLI) were measured as previously described (Witzig *et al*, 1991, 1994). Because the apheresis systems used isolated mononuclear cells, the samples from the harvests were not further manipulated except for depletion of T-cells using magnetic beads.

Data analysis. The patient's clinical record was reviewed to assess disease phase ('plateau' or 'relapse') at the time of apheresis. The number of monoclonal plasma cells and the PBLI values determined in the two groups were compared by the Mann-Whitney test. For patients with serial samples, the highest value was used in the comparisons. $\geqslant 3 \times 10^6$/l plasma cells and a PBLI $\geqslant 0 \cdot 5$% were considered high values (Witzig *et al*, 1993). In the study of serial samples, the number of circulating plasma cells in the initial sample was compared to subsequent samples during the harvest period.

RESULTS

All patients

Eighty-four apheresis samples from 47 patients were analysed. 77% (36/47) of harvests were performed with GM-CSF. 68% (32/47) of patients were in plateau and 32% (15/47) were in relapse. 60% (28/47) of the patients had monoclonal plasma cells in one or more apheresis specimens; 40% (19/47) did not. A comparison of the number of plasma cells and the PBLI between the groups demonstrated

Table I. Peripheral blood plasma cells by disease activity group.

	Disease activity at harvest		
	Plateau ($n = 32$)	Relapse ($n = 15$)	P value
Plasma cells $\times 10^6$/l			0·0001
Mean	1·62	74·64	
Median	0	10·6	
Range	0–18·25	0–396·2	
% patients with any plasma cells	44	93	
% $\geqslant 3 \times 10^6$/l (no. patients)	13 (4)	60 (9)	
PBLI			0·01
Mean	0·01	0·29	
Median	0	0	
Range	0–0·2	0–1·6	
% with PBLI $\geqslant$ 0·5% (no. patients)	0 (0)	20 (3)	

PBLI: peripheral blood labelling index.

Table II. Number of peripheral blood plasma cells detected in serial apheresis samples from 29 patients.

	No. of plasma cells $\times 10^6$/l*		
Patient	First sample	Second sample	Third sample
No GM-CSF ($n = 3$)			
1	0	0	–
2†	31·92 (3)	68·40 (10)	–
3	0 (1)	4·29 (9)	
GM-CSF ($n = 25$)			
Patients 4–15	0	0	–
16†	0 (3)	148·58 (12)	–
17	0 (4)	17·13 (17)	–
18	0 (3)	0·24 (9)	–
19†	4·67 (3)	10·55 (9)	–
20	0 (3)	9·75 (16)	–
21†	2·14 (8)	0 (12)	–
22	0·29 (3)	0 (17)	–
23†	1·06 (3)	0 (24)	–
24†	105·60 (4)	258·80 (10)	396·20 (13)
25†	0 (7)	0·10 (8)	0 (14)
26†	102·82 (9)	287·28 (16)	–
27	0·16 (4)	0 (6)	–
28	1·96 (5)	18·25 (11)	–

*Values in parentheses are day of harvest period (no GM-CSF group) or day of GM-CSF therapy.

† Patients in relapse.

higher values for both parameters in the relapsed group ($P < 0·01$; Table I). In the plateau group, 56% (18/32) had no detectable monoclonal plasma cells compared with only 7% (1/15) in the relapse group.

Patients with serial samples

Nineteen patients had only one sample analysed; 28 patients had serial samples (Table II). 46% (13/28) had no plasma cells at any time during the apheresis period. All 13 patients were in plateau and 12 received GM-CSF therapy. The other 15 had monoclonal plasma cells documented one or more samples.

Nineteen patients had no detectable monoclonal plasma cell on the initial sample. In six of these (five treated with GM-CSF), tumour cells became detectable on subsequent apheresis samples. In four of the six cases the tumour quantitation increased from 0 to a high value ($\geqslant 3 \times 10^6$/l); in the other two it increased from 0 to a low value.

Nine of the 28 patients had tumour cells detected on the first sample. In four of these (each had GM-CSF and a low number of tumour cells), no tumour cells were detected in subsequent samples. In the other five there was a $> 2\times$ increase in the number of tumour cells found in subsequent samples compared with the initial sample.

In two patients the PBLI increased from low ($<$ 0·5%) on the initial samples to 1·2% and 1·4%, respectively, on a subsequent sample during the GM-CSF-stimulated harvests. Both patients were in relapse and had substantial increases ($0–148 \times 10^6$/l and $102·82–287·28 \times 10^6$/l) in the number of plasma cells during the harvest period.

DISCUSSION

This study of patients with MM undergoing PB stem cell harvests demonstrates that these products commonly contain monoclonal plasma cells. This contamination is more likely to occur in patients harvested in relapse. Similar to our results, Mariette *et al* (1994) detected myeloma cells in the leukapheresis samples in 4/10 patients. These findings suggest that patients should be considered for harvest after response to initial treatment or when their PB is documented to be free of tumour cells.

We found that most cases without initial tumour cells had none detected in subsequent samples; however, in six cases contamination of subsequent harvest products was found. In nine cases with initial tumour cells, subsequent apheresis samples showed a substantial increase in five and disappearance in the other four. We also found the appearance of tumour cells or their increase during apheresis can occur in patients not on GM-CSF. Vora *et al* (1994) described a patient with MM who developed monoclonal plasma cells in the PB after treatment with G-CSF; this mobilization was not seen in two additional patients. In the study by Mariette *et al* (1994) the use of G-CSF in three patients did not appear to lead to increased PB tumour cells. Brugger *et al* (1994) reported that 21% of patients with solid tumours without circulating tumour cells at baseline had them mobilized into the PB after chemotherapy and G-CSF. In two patients given GM-CSF we noted an increased plasma cell proliferative rate during the apheresis period. Increased myeloma cell proliferation with cytokines has been previously observed (Klein & Bataille, 1992). These findings indicate that GM-CSF, the apheresis procedure, and the disease state

(plateau or relapse) may all be factors in determining the level of harvest product contamination.

The findings of our study raise several questions. Do patients with contaminated PB stem cell harvests have a shorter time to progression than those that do not? If so, then purging of the harvests should be evaluated. Does chemotherapy used to mobilize stem cells also help to lower the PB tumour burden? This issue has never been formally addressed for MM. These issues are important to resolve so that patients with MM being treated with high-dose therapy and stem cells can achieve more durable responses.

ACKNOWLEDGMENT

This work was supported in part by a Career Development Award from the American Cancer Society (T.E.W.).

REFERENCES

Bakkus, M., Van Riet, I., Van Camp, B. & Thielemans, K. (1994) Evidence that the clonogenic cell in multiple myeloma originates from a pre-switched but somatically mutated B cell. *British Journal of Haematology*, **87**, 68–74.

Berenson, J., Wong, R., Kim, K., Brown, N. & Lichtenstein, A. (1987) Evidence for peripheral blood B lymphocyte but not T lymphocyte involvement in multiple myeloma. *Blood*, **70**, 1550–1553.

Billadeau, D., Quam, L., Thomas, W., Kay, N., Greipp, P., Kyle, R., Oken, M. & Van Ness, B. (1992) Detection and quantitation of malignant cells in the peripheral blood of multiple myeloma patients. *Blood*, **80**, 1818–1824.

Brugger, W., Bross, K., Glatt, M., Weber, F., Mertelsmann, R. & Kanz, L. (1994) Mobilization of tumor cells and hematopoietic progenitor cells into peripheral blood of patients with solid tumors. *Blood*, **83**, 636–640.

Fend, F., Weyrer, K., Drach, J., Schwaiger, A., Umlauft, F. & Grünewald, K. (1993) Immunoglobulin gene rearrangement in plasma cell dyscrasias: detection of small clonal cell populations in peripheral blood and bone marrow. *Leukemia and Lymphoma*, **10**, 223–229.

Klein, B. & Bataille, R. (1992) Cytokine network in human multiple myeloma. *Hematology/Oncology Clinics of North America*, **6**, 273–284.

Mariette, X., Fermand, J.-P. & Brouet, J.-C. (1994) Myeloma cell contamination of peripheral blood stem cell grafts in patients with multiple myeloma treated by high-dose therapy. *Bone Marrow Transplantation*, **14**, 47–50.

Vora, A., Toh, C., Peel, J. & Greaves, M. (1994) Use of granulocyte colony-stimulating factor (G-CSF) for mobilizing peripheral blood stem cells: risk of mobilizing clonal myeloma cells in patients with bone marrow infiltration. *British Journal of Haematology*, **86**, 180–182.

Witzig, T., Dhodapkar, M., Kyle, R. & Greipp, P. (1993) Quantitation of circulating peripheral blood plasma cells and their relationship to disease activity in patients with multiple myeloma. *Cancer*, **72**, 108–113.

Witzig, T., Gonchoroff, N., Ahmann, G., Katzmann, J. & Greipp, P. (1991) T-cell depletion using anti-CD2 coated magnetic beads simplifies the detection of peripheral blood plasma cells. *Journal of Immunological Methods*, **144**, 253–256.

Witzig, T., Gonchoroff, N., Katzmann, J., Therneau, T., Kyle, R. & Greipp, P. (1988) Peripheral blood B-cell labeling indices are a measure of disease activity in patients with monoclonal gammopathies. *Journal of Clinical Oncology*, **6**, 1041–1046.

Witzig, T., Kyle, R., O'Fallon, W. & Greipp, P. (1994) Detection of peripheral blood plasma cells as a predictor of disease course in patients with smouldering multiple myeloma. *British Journal of Haematology*, **87**, 266–272.
