# Supplementary material for: Expression of Concern: Prognostic value of circulating plasma cells in patients with multiple myeloma: A meta-analysis
Source: PLoS One. 2023 Feb 21;18(2):e0282230. doi: 10.1371/journal.pone.0282230 (PMC9942954; doi:10.1371/journal.pone.0282230)
Supplement: S1 File — (ZIP) [file pone.0282230.s001.zip › primary data/excluded research/1994 Significance of Circulating Plasma Cells in Multiple Myeloma_ Leukemia & Lymphoma.pdf]

Taylor & Francis Online
Log in | Register

Journal
Leukemia & Lymphoma
Volume 14, 1994 - Issue 5-6

13 Views
0 CrossRef citations
0 Altmetric

Research article

Significance of Circulating Plasma Cells in Multiple Myeloma

M. Zandecki, T. Facon, C. Preudhomme, F. Canis, V. Izydorczyk, V. Lovi, M. Hammad, F. Bauters & A. Cosson

Pages 491-496 | Received 19 Nov 1993, Published online: 01 Jul 2009
Download citation http://dx.doi.org/10.3109/10428199409049709

Translator disclaimer
References Metrics Reprints & Permissions Get access

Abstract

The number of circulating plasma cells (CPC) was determined on mononuclear cell preparations after Giemsa (morphology) and light chain staining (immunocytochemistry). Both methods gave reproducible and identical results when CPC were 1% or more. Using this limit, no CPC were observed in MGUS (0/11) and primary amyloidosis (0/2), whereas 45/98 (45.9%) multiple myeloma (MM) pts had ⩾ 1% CPC. 3/14 pts (21.4%) in stage I, 5/13 pts (38.5%) in stage II, 20/28 pts (71.4%) in stage III,

4/25 pts (16%) at plateau phase, and 13/18 pts (72.2%) at relapse had ⩾ 1% CPC ($p < 0.001$). Mean beta-2 microglobulin was 3.77 mg/l and 6.08 mg/l for pts without or with ⩾ 1% CPC, respectively ($p = 0.0001$). Presence of CPC was also correlated with an higher percentage of bone marrow PC, but not with the number of Ki-67 positive BM-PC, and not with CRP or LDH levels. K/L and Gamma/Alpha CPC isotype ratio showed these cells as monotypic in nearly all pts. The prognostic value could not really be assessed in this study, as only the initial response to therapy was investigated, and the latter failed to give any difference between pts with and without CPC. So, presence of CPC is not an infrequent finding, but is highly related to tumor mass and active disease; in most if not all patients they are monotypic and certainly belong to the malignant clone. Their prognostic value is unclear but under current investigation; CPC are correlated with B-2M level.

Key Words: Plasma cell, cytoplasmic immunoglobulin, isotype multiple myeloma, blood cell, dissemination,

Log in via your institution

Shibboleth

OpenAthens

Log in to Taylor & Francis Online

Enter your email

Enter your password

Forgot password?

Remember Me

Log in

Or purchase it *

Leukemia & Lymphoma
Volume 14 1994 - Issue 5-6

Article Purchase 24 hours access for USD 54.00 Add to cart

Issue Purchase 30 days access for USD 658.00 Add to cart

* Local tax will be added as applicable

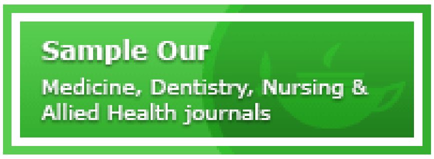
Sample Our
Medicine, Dentistry, Nursing & Allied Health journals

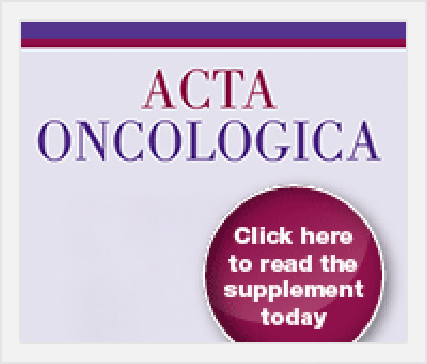
ACTA ONCOLOGICA
Click here to read the supplement today

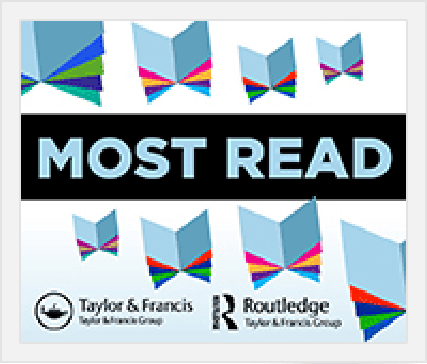
MOST READ
Taylor & Francis
Routledge

Information for

Authors
Editors
Librarians
Societies

Open access

Overview
Open journals
Open Select
Cogent OA

Help and info

Help
FAQs
Press releases
Contact us
Commercial services

Connect with Taylor & Francis

© Informa Group plc Privacy policy & cookies Terms & conditions
Accessibility

Registered in England & Wales No. 3099067
5 Howick Place | London | SW1P 1WG
