# Supplementary material for: Expression of Concern: Prognostic value of circulating plasma cells in patients with multiple myeloma: A meta-analysis
Source: PLoS One. 2023 Feb 21;18(2):e0282230. doi: 10.1371/journal.pone.0282230 (PMC9942954; doi:10.1371/journal.pone.0282230)
Supplement: S1 File — (ZIP) [file pone.0282230.s001.zip › primary data/excluded research/1996 Clonal circulating cells are common in plasma cell proliferative disorders_ a comparison of monoclonal gammopathy of undetermined significance.pdf]

From www.bloodjournal.org by guest on March 3, 2016. For personal use only.

Clonal Circulating Cells Are Common in Plasma Cell Proliferative Disorders: A Comparison of Monoclonal Gammopathy of Undetermined Significance, Smoldering Multiple Myeloma, and Active Myeloma

By Daniel Billadeau, Brian Van Ness, Terry Kimlinger, Robert A. Kyle, Terry M. Therneau, Philip R. Greipp, and Thomas E. Witzig

The blood of most patients with active multiple myeloma (MM) contains cells related to the bone marrow tumor. However, identifying clonal cells in the blood of patients with monoclonal gammopathy of undetermined significance (MGUS) has been difficult. In this study, we analyzed blood mononuclear cells (BMNCs) from 16 patients with MGUS, 2 with amyloidosis, 8 with smoldering MM (SMM), 2 with indolent MM (IMM), and 15 with active MM using three different methods to detect and quantitate clonal cells, ie, immunofluorescence microscopy (IM) for monoclonal plasma cells, three-color flow cytometry (FC) for $CD38^+CD45^-$ $CD45^{dim}$ cells, and the allele-specific oligonucleotide polymerase chain reaction (ASO-PCR). Using ASO-PCR, we were able to detect clonal cells in the blood in 13 of 16 patients with MGUS, 2 of 2 with amyloid, 6 of 8 with SMM, 2 of 2 with IMM, and 13 of 15 with MM. In 9 of the 13 patients with MGUS with blood involvement, the number of clonal cells was very small (<0.04% of the BMNCs). The median percentage of clonal cells as determined by ASO-PCR was 0.02 for MGUS, 0.02 for SMM, and 0.24 for MM. Clonal plasma cells or $CD38^+CD45^-CD45^{dim}$ cells were identified by IM or FC in 6 of 16 MGUS patients, 4 of 8 with SMM, and 11 of 15 with MM. In all cases in which IM or FC detected clonal cells, the ASO-PCR was positive. This study shows that, by using ASO-PCR, clonal cells can be found at very low levels in the blood in most patients with MGUS. However, the number of clonal cells in the blood of MGUS patients is less than those with overt MM ($P = .006$). In contrast to MGUS, patients with active MM are more likely to have identifiable clonal circulating plasma cells ($P = .05$).
© 1996 by The American Society of Hematology.

THE PLASMA CELL proliferative disorders, which include monoclonal gammopathy of undetermined significance (MGUS), smoldering multiple myeloma (SMM), and multiple myeloma (MM), are characterized by the monoclonal expansion of the plasma cell compartment within the bone marrow (BM) as determined by Ig gene rearrangements and the production of a monoclonal M-protein in the serum or urine. MGUS is relatively common and is detected in approximately 1% of adults more than 50 years of age and in 3% of those more than 70 years of age.[1,2] Long-term follow-up studies of MGUS patients have documented that about 20%[3] and 24%[4] of MGUS patients develop MM, amyloidosis (AL type), or a lymphoproliferative disease after 10 and 22 years of follow-up, respectively. Because most patients never develop an overt malignancy, treatment should be avoided and the patient should be observed regularly for symptoms or signs of transformation to active disease.

It has been shown by morphologic, immunofluorescence microscopy (IM), flow cytometric (FC), and molecular techniques that malignant cells circulate in the blood of most patients with active MM.[5-11] However, it has been difficult to show clonal cells in the blood of patients with MGUS using conventional techniques.[5,12-17] The complementarity determining region 3 (CDR3) sequence generated during heavy chain Ig gene rearrangement provides a unique genetic fingerprint for any B-cell clone. The use of the polymerase chain reaction (PCR) along with allele-specific-oligonucleotides (ASO) designed to detect the CDR3 sequence of the tumor has proven to be an invaluable tool in monitoring minimal residual disease[18-21] and in monitoring purging efficacy for autologous blood stem cell transplants in MM.[22-24] We have recently described the use of ASO-PCR to detect malignant cells in the blood of patients with MM.[7] This technique is highly specific and is able to detect 1 malignant cell in a background of 100,000 normal cells.

In this study, the ASO-PCR technique was performed on blood mononuclear cells (BMNCs) from patients with MGUS, SMM, indolent MM (IMM), AL-amyloidosis, and active MM. The goals were to learn if the ASO-PCR technique could detect clonal cells in the blood of patients with MGUS and to compare these results with those obtained from patients with SMM and active MM. To determine if monoclonal plasma cells could be observed in the blood of patients with MGUS, we also analyzed the same blood specimens used for ASO-PCR with IM and FC.

MATERIALS AND METHODS

Patient selection. The patients included in this study were seen and evaluated in the Dysproteinemia Clinic at the Mayo Clinic (Rochester, MN) between August 1993 and March 1995. The study was conducted with the approval of the Institutional Review Board of the Mayo Clinic/Mayo Foundation and the patients gave informed consent to provide additional blood and marrow samples when these tests were ordered for clinical purposes. Patients were diagnosed as MGUS by their treating physician using standard clinical and laboratory criteria, ie, they had a serum monoclonal protein (M-protein) value less than 3 g/dL, ≤10% plasma cells in the marrow, no or a small amount of M-protein in the urine (Bence Jones proteinuria), absence of lytic bone lesions, no anemia due to the plasma cell

From The Department of Laboratory Medicine and Pathology, the Department of Biochemistry, and the Institute of Human Genetics, University of Minnesota, Minneapolis, MN; and the Department of Laboratory Medicine, the Division of Internal Medicine and Hematology, and the Cancer Center Statistics Unit, Mayo Clinic, Rochester, MN.

Submitted October 19, 1995; accepted February 27, 1996.

Supported by National Institutes of Health Grant No. P01 CA-62242. D.B. is a recipient of Immunology Training Grant No. T32 A1-07313.

Address reprint requests to Thomas E. Witzig, MD, 920E Hilton Bldg, Mayo Clinic, Rochester, MN 55905.

The publication costs of this article were defrayed in part by page charge payment. This article must therefore be hereby marked "advertisement" in accordance with 18 U.S.C. section 1734 solely to indicate this fact.

© 1996 by The American Society of Hematology.

0006-4971/96/8801-0136$3.00/0

From www.bloodjournal.org by guest on March 3, 2016. For personal use only.

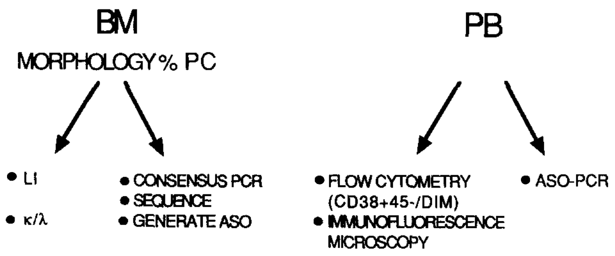

Fig 1. Schema for the analysis of bone marrow (BM) and peripheral blood (PB) specimens.

proliferation, hypercalcemia, or renal insufficiency. Because these patients were diagnosed within the last 2 years, it was not possible to fulfill one important criteria of MGUS, ie, stability of the M-protein during long-term follow-up.[25] Patients with SMM had ≥10% monoclonal marrow plasma cells, a monoclonal protein in the serum or urine, no known lytic or sclerotic bone lesions, a hemoglobin level greater than 10 g/dL, normal serum calcium and creatinine, a clinical diagnosis of SMM by the treating hematologist, no prior chemotherapy, and the recommendation that the patient be observed without chemotherapy. IMM patients met the same criteria as SMM patients, except for the presence of anemia felt to be due to the plasma cell proliferation or asymptomatic lytic bone lesions. Patients with active MM had either newly diagnosed or relapsed MM that required chemotherapy. The patients with AL-amyloid had monoclonal marrow plasma cells, a monoclonal protein in the serum or urine, no known lytic or sclerotic bone lesions, and a tissue biopsy positive for amyloid.

Sample collection and distribution. Patient marrow and blood samples were split and analyzed as outlined in Fig 1. Monoclonal plasma cells and the plasma cell labeling index were determined on the marrow cells as previously described.[26] Mononuclear cells were isolated on Ficoll-Hypaque from heparinized blood and split into two samples. No procedures were performed to specifically remove adherent cells. One sample was used for the IM technique and three-color (CD38/CD45/κ or λ) FC; the other was used for ASO-PCR. The IM, FC, and ASO-PCR tests were performed by separate laboratory personnel blinded to the results of the other tests or the clinical status of the patient. The results from each of the three methods were reported as a percentage of the MNCs for ease of comparison.

FC and IM. The presence and number of circulating monoclonal plasma cells and the blood labelling index (BLI) were obtained by an IM procedure as previously described.[27,28] Monoclonal plasma cells were identified by their morphology (eccentric nucleus with abundant cytoplasm) and by in situ visual documentation of cytoplasmic Ig (cIg) light chain restriction. Three-color FC was performed as previously described.[29,30] Briefly, 2×10^6 MNC were suspended in 100 μL of phosphate-buffered saline (PBS); 10 μL of antihuman CD45-PerCP (Becton Dickinson, Mountain View, CA) and 10 μL of antihuman CD38-phycoerythrin (PE; Becton Dickinson) were added to the cells; and the mixture was incubated in the dark for 15 minutes at room temperature and then washed with PBS. Four milliliters of lysolecithin solution was added to the cell pellet and incubated for 30 minutes in the dark at 4°C. After washing with 3 mL of PBS/3% bovine serum albumin (BSA), the pellet was resuspended in 200 μL PBS/BSA and 100 μL was added to each of two 12- × 75-mm tubes. To these tubes was added either fluorescein isothiocyanate (FITC)-conjugated antihuman κ or λ light chain antibody (Biosource International, Camarillo, CA) and the suspension was incubated for 30 minutes in the dark at 4°C. After incubation, the cells were washed with 2 mL PBS, resuspended in 0.5 mL 1% paraformaldehyde, and analyzed on a FACScan FC. Gates were set around the $CD38^+CD45^-CD45^{dim}$ quadrant and the percentage of cells expressing κ and λ were compared to determine monoclonality. $CD45^{dim}$ cells were those that occupied the gated region between the cells in the sample that did not stain for CD38 or CD45 and the normal lymphocytes with their characteristic strong CD45 expression.[30] Monoclonal κ was defined as κ:λ ratio ≥5. Monoclonal λ was defined as a κ:λ ratio ≤0.5.

DNA and RNA isolation from patient samples. DNA was prepared from the marrow and BMNCs of patients by salt-out extraction of 1×10^7 cells. Briefly, the cell pellet was resuspended in 1 mL of TE (10 mmol/L Tris, pH 8.0, and 1 mmol/L EDTA) and then 2 mL of TNES (10 mmol/L Tris, pH 8.0, 1 mmol/L EDTA, 50 mmol/L NaCl, and 2% sodium dodecyl sulfate [SDS]) was added to lyse the cells. One milliliter of 5 mol/L NH_4Ac was then added, vortexed for 15 seconds, and placed on ice for 30 minutes to facilitate the precipitation of the proteins. Precipitated proteins were pelleted by centrifugation at 3,000 RPM for 15 minutes. The supernatant was saved and the DNA was precipitated by the addition of an equal volume of isopropanol. The DNA precipitate was washed 2× in 70% ethanol, air-dried, and resuspended in 500 mL TE. RNA was prepared from 1×10^7 bone marrow cells using TRIzol reagent (GIBCO-BRL, Gaithersburg, MD) per the manufacturer's protocol.

Consensus PCR of rearranged IgH genes. One microgram of marrow DNA was subjected to VFra3/JH or VH family-specific/JH consensus Ig heavy chain PCR, as previously described.[7,31] In some cases, use of a VFr1c oligonucleotide was used for consensus amplification[32] with the following modifications: the final $MgCl_2$ concentration was 1.2 mmol/L and the cycling conditions were an initial denaturation of 5 minutes at 94°C followed by 40 cycles of denaturation at 92°C for 15 seconds, annealling at 60°C for 15 seconds, and extension at 74°C for 25 seconds. In some cases, 5 μg of RNA was reverse transcribed using isotype-specific constant region primers and then amplified using the VH family-specific primers or VFr1c along with an internal constant region primer, as previously described.[31] PCR products were separated on 7% polyacrylamide gels, isolated, and sequenced as previously described.[7] Along with each patient sample, a normal marrow is run as a control. In the absence of a clonally expanded population, we do not obtain a single PCR product, but rather a nonspecific smear. To further validate the ASO-PCR results, three samples from normal donors at the Mayo Clinic laboratory were sent blinded to the University of Minnesota laboratory and none yielded a consensus product.

Detection and quantitation of clonal circulating cells by ASO-PCR. Allele-specific oligonucleotides were generated using a PCR-Mate oligo synthesizer (ABI, Foster City, CA) to the most specific portion of the CDR3 region based on BLAST (National Center for Biotechnology Information [NCBI]) sequence search comparisons. ASO-PCR was performed as previously described.[7,21] Briefly, patient marrow DNA dilution curves were generated by serially diluting the patient DNA into sterile ddH_2O in 10-fold decrements. An appropriate amount of patient diluted marrow DNA was then mixed with normal marrow DNA to yield a target concentration of 1 μg DNA/50 μL reaction. Along with the diluted marrow samples, 1 μg of patient blood lymphocyte DNA was run in triplicate. The 1× PCR buffer consisted of 20 mmol/L Tris, pH 8.4, 50 mmol/L KCl, 1.5 mmol/L $MgCl_2$, 200 mmol/L of each dNTP, 20 pmol of each oligonucleotide, and 2.5 U of Taq polymerase (Perkin Elmer Cetus, Branchburg, NJ). The ASO in each instance was paired with a JH-specific or JH-consensus oligonucleotide.[7,21] PCR cycling conditions were as follows: an initial 5 minutes of denaturation at 95°C followed by 15 seconds at 92°C, 15 seconds at the appropriate annealling temperature for the ASO, and 25 seconds at 74°C. Annealling temperatures varied between 58°C and 65°C depending on the calculated Tm of the ASO. To visualize the PCR products, the ASO was end-labeled with γ^{32}P-ATP as described,[31] and the PCR product was

From www.bloodjournal.org by guest on March 3, 2016. For personal use only.

separated on an 8% polyacrylamide gel and subjected to autoradiography at room temperature for 1 to 4 hours. Autoradiograms were scanned using the Molecular Dynamics Personal Densitometer SI (Molecular Dynamics, Sunnyvale, CA), and signal intensities of the radiolabeled PCR products were determined using the IP Lab Gel software (Signal Analytics Corp, Vienna, VA). Standard curves generated from serial dilutions showed correlation coefficients greater than .95; Scheffe's 95% confidence intervals were computed as previously described[7] and ranged within 0.5 log of the experimentally determined value. This approach has been shown to be highly specific for detection of cells clonally related to the plasma cell tumor.[7,21] In all cases, negative controls included ASO-PCR of normal bone marrows, as well as cross-checks on unrelated tumor samples.

Statistical methods. The percentage of clonal cells (as a percentage of the blood MNCs) determined by IM, FC, and ASO-PCR was compared by using the Kruskall-Wallis test for three-group comparisons. The two-group comparisons were performed with the Mann-Whitney or rank-sum test. The proportions of the cases that had circulating clonal plasma cells or $CD38^+CD45^-CD45^{dim}$ cells by IM or FC were compared by using either the Fisher's Exact Test or the χ^2 test. For the ASO-PCR results that were below the detectable limits of the assay, we assigned a value of 0. Because of the small number of patients with amyloidosis and IMM, for the three-group comparisons, those patients with amyloidosis were combined with those with MGUS and the patients with IMM were combined with those with active MM.

RESULTS

Patient population. Sixty-four patients with monoclonal gammopathies were accrued to the study and patient marrow and BMNC samples were analyzed as diagrammed in Fig 1. The diagnoses of the 64 patients included 24 MGUS, 7 AL-amyloidosis, 9 SMM, 2 IMM, and 22 active MM.

Results were obtained with IM or FC in all 64 patients, whereas only 43 of 64 yielded PCR products after consensus amplification of the marrow tumor-rearranged IgH gene. These 43 patients became the focus of our comparative analysis of the ASO-PCR, IM, and FC techniques. Of the 43 patients, there were 16 MGUS, 2 AL-amyloidosis, 8 SMM, 2 IMM, and 15 active MM (9 were new and untreated and 6 were relapsed).

MGUS. We have previously shown that the blood of patients with active MM contains circulating cells related to the plasma cell tumor as determined by ASO-PCR.[7] However, the exact morphologic identification of these cells was not established because ASO-PCR detects all B cells related to the malignant clone regardless of morphology. In an attempt to detect clonal circulating cells in MGUS and to determine the morphologic characterization of the circulating clone, we used ASO-PCR, IM, and FC on the same group of patients. The results of the detection and quantitation of clonal cells in the blood are summarized in Table 1. For patients with MGUS, the ASO-PCR technique detected clonal cells in 13 of 16; the remaining 3 were below the detection level of the assay. Of the 13 patients that showed blood involvement, only 4 were ≥0.1% and the remaining 9 ranged from 0.001% to 0.07%, with a median blood involvement of 0.025% (Table 1). DNA samples that were negative by ASO-PCR were also amplified with N-*ras* gene-specific primers to confirm the integrity of the blood DNA sample (data not shown).

Table 1. Detection of Clonal Circulating Cells in Plasma Cell Disorders by Cellular and Molecular Techniques

	Bone Marrow		Peripheral Blood*		
Disease	% PC	LI	IM (% PC)	FC (% $CD38^+45^-45^{dim}$)	ASO-PCR (% clonal cells)
MGUS1	10	0.1	0	0.2	0.07
MGUS2	2	0	0	0	0.008
MGUS3	3	0	0	0	0.031
MGUS4	5	0	0	0	<0.002
MGUS5	5	0	0	0.97	0.02
MGUS6	2	0	ND	0	<0.001
MGUS7	3	0	0.18	0.4	0.3
MGUS8	9	0.2	0	0	0.001
MGUS9	10	0	0	0	0.003
MGUS10	3	0	0	0	0.04
MGUS11	3	0	0.006	0	0.002
MGUS12	2	0	ND	0	0.01
MGUS13	2	0	0	0	0.1
MGUS14	9	0	0	0	<0.0009
MGUS15	8	0	0.01	0.06	0.1
MGUS16	3	0	0.04	0	0.1
SMM1	10	0	0.03	0	0.02
SMM2	14	0	0	0	<0.01
SMM3	19	0.2	0	0	<0.009
SMM4	15	0.2	0	0	0.008
SMM5	30	0.5	0	0	0.001
SMM6	14	0.2	0.02	0.01	10
SMM7	56	0	0.04	0.04	0.03
SMM8	35	0.1	0	0.05	0.06
IMM1	10	0	0	0	0.01
IMM2	28	0	7.6	5.2	2
AMY1	16	0.2	0	0	0.01
AMY2	3	0	0	0	0.07
MM1	38	0.6	1.15	0.04	0.6
MM2†	57	0.9	2.25	0	0.8
MM3	37	0.4	1.54	0.55	6
MM4	14	0	0	0	<0.001
MM5	54	0.5	0.008	0.08	0.1
MM6	29	0	0	0	0.02
MM7	90	1.4	2.01	0.62	0.7
MM8	30	0.4	0.92	0.04	0.2
MM9†	25	0	2.79	ND	0.3
MM10†	11	0	0	ND	<0.005
MM11†	34	0	0.09	0.12	0.2
MM12†	50	0.7	0.99	4.48	7
MM13	67	0.2	0	0	0.001
MM14†	61	0.6	ND	0.05	0.4
MM15	70	0	0.1	0	0.08

Abbreviations: PC, plasma cells; LI, labeling index; AMY, amyloidosis; ND, not done.

* Results for each technique are percentages of mononuclear cells.

† Patients with relapsed myeloma.

The IM technique detected circulating monoclonal plasma cells in 4 of 14 cases, and FC detected clonal $CD38^+CD45^-CD45^{dim}$ cells in 4 of 16 cases (Table 1). The ASO-PCR technique detected clonal cells in each of the 6 patients with MGUS in which either the IM or FC method was positive. In the 4 cases in which FC detected clonal

From www.bloodjournal.org by guest on March 3, 2016. For personal use only.

$CD38^+CD45^-CD45^{dim}$ cells, only 2 of these showed detectable circulating clonal plasma cells using IM. This finding suggests that, in some patients with MGUS, clonal cells lacking typical plasma cell morphology can be found in the blood. Figure 2A through C shows the results from a patient with MGUS who was positive by ASO-PCR, and FC showed monoclonal $CD38^+CD45^-CD45^{dim}$ cells. However, although plasmacytoid cells were seen (Fig 2B), they did not meet the criteria for monoclonal plasma cells by the IM technique. Figure 2D through E is an example of a patient with MGUS who had a positive analysis with ASO-PCR, circulating monoclonal plasma cells by IM, and clonal $CD38^+$ $CD45^-CD45^{dim}$ cells by FC. Because of the limits in sensitivity of the immunologic techniques, the remaining 8 cases were negative by both IM and FC but positive by the more sensitive ASO-PCR.

SMM. Of the 8 SMM analyzed by ASO-PCR, 6 were positive and 2 were below the detection level of the assay (Table 1). One patient had blood involvement that was estimated to be 10% by ASO-PCR. The remaining 5 patients had blood involvement between 0.001% and 0.06%, with a median blood involvement of 0.02% for all 8 patients. IM or FC showed clonal cells in 4 of 8 patients, and the ASO-PCR technique detected clonal cells in each of the 4 patients with SMM in which either the IM or FC method were positive. The percentages of blood involvement as determined by each method were generally in good agreement; however, ASO-PCR analysis of SMM6 found an estimated blood involvement of 10%, which is significantly different from that observed by both IM and FC (0.02% and 0.01%, respectively). The significance of this discrepancy is discussed below. In the remaining cases that were negative by the cellular techniques (SMM4 and SMM5) the percentage of blood involvement as determined by ASO-PCR may be below the detection limits of the other two methods.

IMM and AL-amyloidosis. All 4 cases of IMM and amyloidosis were positive by ASO-PCR (Table 1). Neither the IM nor FC technique detected clonal cells in the patients with amyloidosis, and the level of involvement detected by ASO-PCR was similar to that found in MGUS. In 1 of the IMM patients, large numbers of clonal cells were detected by both IM and FC.

Active MM. ASO-PCR analysis of the 15 MM patients showed 13 patients with clonal cells in the blood; 2 were below the detection limits of the assay (Table 1). IM and FC of the MM samples showed circulating cells in 10 of 14 and 8 of 13 patients, respectively. As found for the patients with MGUS and SMM, the ASO-PCR technique detected clonal cells in each of the 11 patients with MM in which either the IM or FC method were positive.

MGUS versus SMM versus active MM. The percentages of patients with MGUS, SMM, and active MM that had clonal plasma cells or $CD38^+CD45^-CD45^{dim}$ cells detected by IM or FC were 38%, 50%, and 73%, respectively (P = .05). Patients with active MM were more likely to have clonal plasma cells detected by these methods than patients with MGUS (P = .05). When patients with active disease (IMM or MM) were compared with those with inactive disease (MGUS, SMM, or amyloid), they were also more likely to have clonal plasma cells detected by FC or IM (P = .04).

For the comparison of the percentage of clonal cells detected by ASO-PCR for the different plasma cell proliferative diseases, the patients with amyloidosis were grouped with MGUS and those with IMM were grouped with active MM (Table 2). To increase the numbers of patients for this analysis, we also included the ASO-PCR results from 14 patients with MM from our previous report.[7] In contrast to MGUS and SMM, patients with MM had a wide range of blood involvement as determined by ASO-PCR (Fig 3). The difference between the percentage of clonal cells detected by ASO-PCR in the three groups was statistically significant (P = .01). The patients with active MM had a statistically significant increase in the percentage of clonal cells in the blood when compared with those with MGUS (P = .006) and a borderline significant increase compared with those with SMM (P = .06). There was no difference between the patients with SMM and those with MGUS (P = .62).

DISCUSSION

The purpose of this study was to directly compare different techniques that serve to identify and characterize cell populations in the blood that are clonally related to the expanded plasma cell population seen in the marrow. Moreover, this also provided an opportunity to compare several plasma cell disorders to determine if there are differences in blood involvement.

It has been difficult to detect clonal cells in the blood of patients with MGUS using traditional techniques. In this study, we showed that, with ASO-PCR, most patients with MGUS have very small numbers of clonal cells in the blood. Previous analyses of the blood from patients with MGUS using immunohistochemistry[33] or FC with antibodies directed against B-cell and plasma cell-associated antigens[8,12] were not able to detect circulating monoclonal plasma cells. In addition, four studies using Southern blotting of DNA from patients with MGUS were unable to identify B-cell

Fig 2. Identification of clonal circulating cells by both cellular and molecular methods in two patients with MGUS. The photomicrographs (B and E) in each case are a composite of representative cells from 5 separate microscope fields. Each field was photographed on a Zeiss Axiophot fluorescence microscope and the data stored on a Macintosh Power PC 8100/80 as a TIFF image. The images were merged into one file using MacDraw Pro. Patient no. 1 (A through C). (A) FC plot showing a small number of $CD38^+CD45^-$ cells (light green shaded region) that were monoclonal for κ cytoplasmic light chain. (B) IM showing cIg$^+$ B cells with plasmacytoid morphology (original magnification × 630). (C) ASO-PCR analysis of the patient PBMNC sample showing the bone marrow titration curve and peripheral blood sample. NT, no template. Patient no. 2 (D through F). (D) FC plot showing $CD38^+CD45^-$ cells (light green shaded region) that were monoclonal for κ cytoplasmic light chain. (E) IM showing monoclonal κ B cells with definite plasma cell morphology (original magnification × 630). (F) ASO-PCR showing clonal cells in the peripheral blood.

From www.bloodjournal.org by guest on March 3, 2016. For personal use only.

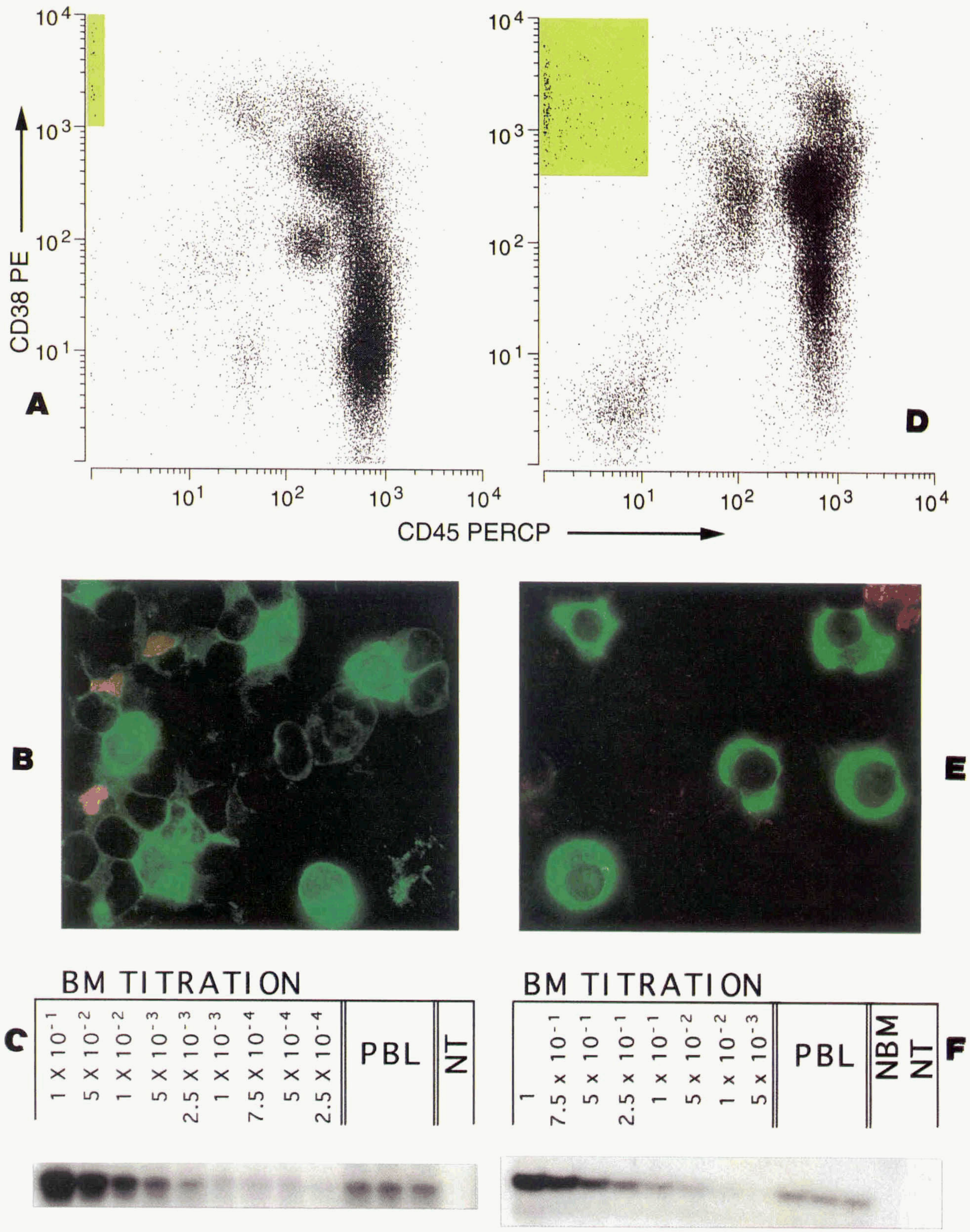

From www.bloodjournal.org by guest on March 3, 2016. For personal use only.

Table 2. Percentage of Clonal Cells in the Blood Detected by ASO-PCR in the Various Plasma Cell Proliferative Groups

Diagnosis (n)	% Clonal Cell by ASO-PCR Mean	Median	Range
MGUS/amyloid (18)	0.06	0.02	0-0.3
SMM (8)	1.23	0.02	0-10
IMM/active MM (31)	1.85	0.21	0-33

gene rearrangements in the blood cells.[14-17] When cytokines were added to blood cells and incubated for several days, plasma cell outgrowths were obtained from the patients with MM but not from those with MGUS.[11,34] Büchi et al[35] studied 23 patients with MGUS and found no alteration in the κ:λ ratio in blood lymphocytes; however, alterations have been found by others.[36,37] A B-cell clone from the blood of a patient with MGUS was isolated and demonstrated to have an idiotypic determinant similar to the serum M-protein and to contain an identical Ig gene rearrangement.[38] Taken together, these data appear contradictory concerning blood involvement in MGUS. However, in light of our data showing the very low percentage of blood involvement in MGUS compared with MM, it is not suprising that in most instances clonal circulating cells were not detected.

The presence of circulating cells clonally related to the marrow tumor has been shown in previous studies by ASO-PCR and IM for patients with active MM.[5,7] In fact, the number and labeling index of the circulating cells is a marker of disease activity.[5,39] The results in this study confirm and extend these results by showing that the percentage of blood involvement is typically higher in MM than in MGUS but varies among patients by as much as 4 logs (Fig 3). Because low levels of clonal circulating cells can be found in MGUS and MM, their presence cannot be used to distinguish these diseases.

In this study, we showed that monoclonal plasma cells and $CD38^+CD45^-CD45^{dim}$ cells can be detected in some cases of MGUS by IM and FC. However, in 10 of the patients with MGUS and amyloid that had clonal cells detected by ASO-PCR, we were unable to detect monoclonal plasma cells or $CD38^+CD45^-CD45^{dim}$ cells in the blood. In addition, there were several cases (SMM6 and MM3) in which substantially more clonal cells were detected by ASO-PCR than by the IM and FC techniques. In the case of the MGUS and amyloid cases, the discrepancy could be due to the fact that plasma cells were indeed present but below the limits of detection by IM and FC. In the cases in which more cells were detected by ASO-PCR, it is likely that the clonal cells detected by ASO-PCR were less differentiated and that all of them did not have plasma cell morphology or a $CD38^+CD45^-CD45^{dim}$ immunophenotype. Further cell sorting studies are needed to examine which B-cell population contains the clonal cells and correlations with overall survival will provide clues to the significance of these different clonal cell populations.

By analyzing the same blood samples with each of the ASO-PCR, IM, and FC techniques, we found that whenever IM and FC detected monoclonal cells in the blood, we always detected clonal cells by ASO-PCR, ie, there were no false-positives with IM or FC. In addition, when ASO-PCR was negative, the IM and FC techniques were also always negative, ie, there were no false-negatives by ASO-PCR. This study indicates that, when molecular techniques detect clonal cells in the blood of patients with monoclonal gammopathies, IM and FC may show cells with plasma cell morphology and immunophenotype. In some instances, FC detected clonal cells at levels that would be expected to have been detected by IM, but the lack of typical plasma cell morphology precluded their detection. Certainly, the FC and the IM methods are not equivalent and provide different measures of clonally related cells. The ASO-PCR technique is much more sensitive and has the advantage of sequence specificity to confirm that the cells are clonally related to

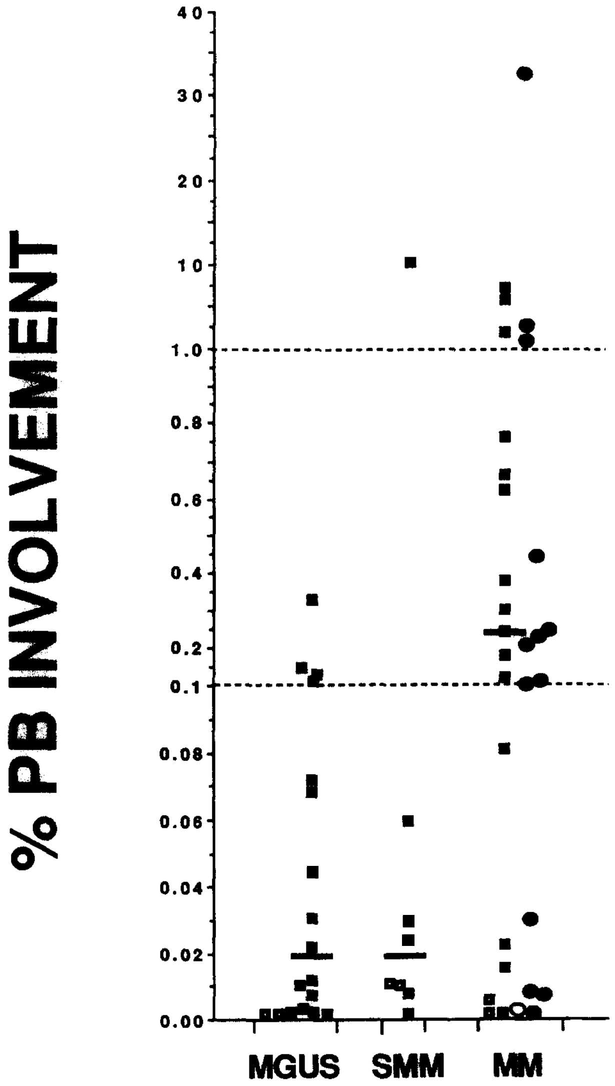

Fig 3. Plot of the percentage of clonal cells detected in the peripheral blood by ASO-PCR versus disease category. The horizontal bars at 0.02 (MGUS), 0.02 (SMM), and 0.21 (MM) represent median values. Open symbols indicate no detection below the limits of that level of sensitivity. (●) Values derived from previous work.[7]

From www.bloodjournal.org by guest on March 3, 2016. For personal use only.

the plasma cell tumor regardless of their morphology; therefore, the population of cells identified by ASO-PCR would include cells with plasma cell morphology (if they were present) and clonally related cells without plasma cell morphology. To learn the immunophenotype of the clonal cells that do not have the morphology of plasma cells, sorting specific cell populations followed by in situ hybridization using the ASO will be required. These experiments will be important to properly identify the cell population that should be targeted with new therapeutic approaches.

The finding of clonal cells in the the blood of patients with MGUS has implications about the way we view the pathogenesis of MM. It is clear that, even at this early, premalignant stage of disease, the clone is present in the blood and has differentiated to the plasma cell stage in the marrow. Because most of these patients will never actually progress to the malignant phase of the disease, there must be additional insults to the plasma cell or precursor cells to cause them to proliferate, secrete cytokines, and produce the signs and symptoms of active myeloma.

Although monoclonal plasma cells were absent or in very low amount in the blood of patients with MGUS, they were easily detected in the marrow of all these patients by IM. Therefore, patients with MGUS and MM are similar in that they both have clonal plasma cells in the marrow, a monoclonal protein in the serum or urine, and clonal cells in the blood. They differ in that patients with MM typically have more M-protein, a greater percentage of plasma cells in the marrow, marrow plasma cells with a higher proliferative rate, and lytic bone lesions. Previous studies have found that, in contrast to active myeloma, marrow plasma cells in patients with MGUS have a low proliferative rate.[39,40] This reduces the probability that blood involvement in patients with MGUS results from spillover of cells proliferating in the marrow compartment. In addition, a recent report by Sahota et al[41] has shown that cells isolated from the marrow of patients with MGUS show clonally related populations with distinct somatic mutations. These results suggest that cells from patients with MGUS retain clonal diversification and raise the possibility that detection of clonal cells in the blood may reflect cells in transit from distal sites that home to the marrow and differentiate into mature plasma cells. Once in the marrow, the proliferative capacity of MGUS cells is significantly reduced compared with the plasma cells typically found in patients with active myeloma.

We have also shown that the blood from patients with SMM, IMM, and amyloidosis can contain clonal circulating cells in the blood. It is now apparent that clonal cells can circulate in the blood of patients with all of the plasma cell proliferative diseases. We have previously shown that patients with SMM who have an increased number of monoclonal plasma cells in the blood have a shorter time to progression to overt MM than those patients who do not.[27] A recent study by Isaksson et al[37] showed that patients with MGUS who had excess blood B lymphocytes expressing the same light chain isotype as the patient's M-protein had an increased risk of transfromation to a B-cell malignancy. It will be interesting to learn with long-term follow-up studies whether the results of the IM, FC, and ASO-PCR studies on the patients with MGUS in this study can predict transformation to overt malignant disease.

This study shows by both cellular and molecular approaches that clonal circulating cells are a common occurrence in plasma cell disorders. Furthermore, the majority of patients with MM have circulating plasma cells as determined by IM and FC that may reflect their active disease state. This is in contrast to what is observed in MGUS, in which relatively few patients show clonal cells as determined by either cellular method, but are clearly positive by ASO-PCR. It is clear that clonal circulating cells are evident in most patients with MGUS; however, the identification of such cells and their impact on possible progression to overt MM will require longitudinal studies. Based on these results, monoclonal gammopathies should be considered hematopoietic disorders involving many lymphoid compartments rather than purely marrow disorders.

REFERENCES

1. Axelsson U, Bachmann R, Hällén J: Frequency of pathological proteins (M-components) in 6,995 sera from an adult population. Acta Med Scand 179:235, 1966

2. Kyle R, Finkelstein S, Elveback L, Kurland L: Incidence of monoclonal proteins in a Minnesota community with a cluster of multiple myeloma. Blood 40:719, 1972

3. Blade J, Lopez-Guillermo A, Rozman C, Cervantes F, Salgado C, Aguilar J-L, Vives-Corrons J-L, Montserrat E: Malignant transformation and life expectancy in monoclonal gammopathy of undetermined significance. Br J Haematol 81:391, 1992

4. Kyle R: "Benign" monoclonal gammopathy after 20-35 years of follow-up. Mayo Clin Proc 68:26, 1993

5. Witzig T, Dhodapkar M, Kyle R, Greipp P: Quantitation of circulating peripheral blood plasma cells and their relationship to disease activity in patients with multiple myeloma. Cancer 72:108, 1993

6. Berenson J, Wong R, Kim K, Brown N, Lichtenstein A: Evidence for peripheral blood B lymphocyte but not T lymphocyte involvement in multiple myeloma. Blood 70:1550, 1987

7. Billadeau D, Quam L, Thomas W, Kay N, Greipp P, Kyle R, Oken MM, Van Ness B: Detection and quantitation of malignant cells in the peripheral blood of multiple myeloma patients. Blood 80:1818, 1992

8. Boccadoro M, Omedé P, Massaia M, Dianzani U, Pioppo P, Battaglio S, Meregalli M, Pileri A: Human myeloma: Several subsers of circulating lymphocytes express plasma cell-associated antigens. Eur J Haematol 40:299, 1988

9. Caligaris-Cappio F, Bergui L, Gaidano G, Schena M, Putto P, Merico F, Riva M: Circulating malignant precursors in monoclonal gammopathies. Eur J Haematol Suppl 43:27, 1989

10. Pilarski L, Belch A: Circulating monoclonal B cells expressing P clycoprotein may be a reservoir of multidrug-resistant disease in multiple myeloma. Blood 83:724, 1994

11. Sawamura M, Murakami H, Tamura J, Matsushima T, Sato S, Naruse T, Tsuchiya J: Tumor necrosis factor-α and interleukin 4 promote the differentiation of myeloma cell precursors in multiple myeloma. Br J Haematol 88:17, 1994

12. Omedé P, Boccadoro M, Gallone G, Frieri R, Battaglio S, Redoglia V, Pileri A: Multiple myeloma: Increased circulating lymphocytes carrying plasma cell-associated antigens as an indicator of poor survival. Blood 76:1375, 1990

13. Shimizu K, Murate T, Kunii A: Circulating immunoglobulin-secreting cells in patients with plasma cell dyscrasia. Blood 55:590, 1980

From www.bloodjournal.org by guest on March 3, 2016. For personal use only.

14. Bagg A, Becker P, Bezwoda W, Van Rensburg L, Mendelow B: Circulating monotypic B-cells in multiple myeloma: Association with lambda paraproteins. Br J Haematol 72:167, 1989

15. Chiu E, Ganeshaguru K, Hoffbrand A, Mehta A: Circulating monoclonal B lymphocytes in multiple myeloma. Br J Haematol 72:28, 1989

16. Cassel A, Leibovitz N, Hornstein L, Quitt M, Aghai E: Evidence for the existence of circulating monoclonal B-lymphocytes in multiple myeloma patients. Exp Hematol 18:1171, 1990

17. Fend F, Weyrer K, Drach J, Schwaiger A, Umlauft F, Grünewald K: Immunoglobulin gene rearrangement in plasma cell dyscrasias: Detection of small clonal cell populations in peripheral blood and bone marrow. Leuk Lymphoma 10:223, 1993

18. Yamada M, Hudson S, Tournay O, Bittenbenders S, Shane SS, Lange B, Tsujimoto Y, Caton AJ, Rovera G: Detection of minimal residual disease in hematopoietic malignancies in B-cell lineage by using third-complementary-determining region (CDRIII)-specific probes. Proc Natl Acad Sci USA 86:5123, 1989

19. Yamada M, Wasserman R, Lange B, Richard BA, Womer RB, Rovera G: Minimal residual disease in childhood B-lineage lymphoblastic leukemia. N Engl J Med 323:448, 1990

20. Jonsson OG, Kitchens RL, Scott FC, Smith RG: Detection of minimal residual disease in acute lymphoblastic leukemia using immunoglobulin hypervariable region specific oligonucleotide probes. Blood 76:2072, 1990

21. Billadeau D, Blackstadt M, Greipp P, Kyle RA, Oken MM, Kay N, Van Ness B: Analysis of B-lymphoid malignancies using allele-specific polymerase chain reaction: A technique for sequential quantitation of residual disease. Blood 78:3021, 1991

22. Vescio RA, Hong CH, Cao J, Kim A, Schiller GJ, Lichtenstein AK, Berenson RJ, Berenson JR: The hematopoietic stem cell antigen, CD34 is not expressed on the malignant cells in multiple myeloma. Blood 84:3283, 1994

23. Schiller G, Vescio R, Freytes C, Spitzer G, Sahebi F, Lee M, Wu CH, Cao J, Lee JC, Hong CH, Lichtenstein A, Lill M, Hall J, Berenson R, Berenson J: Transplantation of $CD34^+$ peripheral blood progenitor cells after high-dose chemotherapy for patients with advanced multiple myeloma. Blood 86:390, 1995

24. Gazitt Y, Reading CC, Hoffman R, Wickrema A, Vesole DH, Jagannath S, Condino J, Lee B, Barlogie B, Tricot G: Purified $CD34^+$ Lin^- Thy^+ stem cells do not contain clonal myeloma cells. Blood 86:381, 1995

25. Kyle R: Monoclonal gammopathy of undetermined significance. Blood Rev 8:135, 1994

26. Greipp P, Witzig T, Gonchoroff N: Immunofluorescence labeling indices in multiple myeloma and related monoclonal gammopathies. Mayo Clin Proc 62:969, 1989

27. Witzig T, Kyle R, O'Fallon W, Greipp P: Detection of peripheral blood plasma cells as a predictor of disease course in patients with smoldering multiple myeloma. Br J Haematol 87:266, 1994

28. Witzig T, Gonchoroff N, Ahmann G, Katzmann J, Greipp P: T-cell depletion using anti-CD2 coated magnetic beads simplifies the detection of peripheral blood plasma cells. J Immunol Methods 144:253, 1991

29. Westendorf J, Ahmann G, Armitage R, Spriggs M, Lust J, Greipp P, Katzmann J, Jelinek D: CD40 expression in malignant plasma cells: Role in stimulation of autocrine IL-6 secretion by a human myeloma cell line. J Immunol 152:117, 1994

30. Witzig T, Kimlinger T, Ahmann G, Katzmann J, Greipp P: Detection of myeloma cells in the peripheral blood by flow cytometry. Cytometry 1996 (in press)

31. Billadeau D, Ahmann G, Greipp P, Van Ness B: The bone marrow of multiple myeloma patients contains B cell populations at different stages of differentiation that are clonally related to the malignant plasma cell. J Exp Med 178:1023, 1993

32. Aubin J, Davi F, Nguyen SF, Leboeuf D, Debert C, Taher M, Valensi F, Canioni D, Brousse N, Varet B, Flandrin G, Macintyre EA: Description of a novel FR1 IgH PCR strategy and its comparison with three other strategies for the detection of clonality in B cell malignancies. Leukemia 9:471, 1995

33. Zandecki M, Facon T, Preudhomme C, Canis F, Izydorczyk V, Lovi V, Hammad M, Bauters F, Cosson A: Significance of circulating plasma cells in multiple myeloma. Leuk Lymphoma 14:491, 1994

34. Goto H, Shimazaki C, Ashihara E, Ohkawa K, Oku N, Inaba T, Murakami S, Fujita N, Nakagawa M: Effects of interleukin-3 and interleukin-6 on peripheral blood cells from multiple myeloma patients and their clinical significance. Acta Haematol 88:129, 1992

35. Büchi G, Giorotto M, Veglio M, Clerico M, Gario S, Termine G, Autino R: Kappa/lambda ratio on peripheral blood lymphocytes and bone marrow plasma cell labeling index in monoclonal gammopathies. Haematologica 75:132, 1990

36. Pettersson D, Mellstedt H, Holm G, Bjøorkholm M: Monoclonal blood lymphocytes in benign monoclonal gammopathy and multiple myeloma in relation to clinical stage. Scand J Haematol 27:287, 1981

37. Isaksson E, Björkholm M, Holm G, Johansson B, Mellstedt H, Österborg A: Blood clonal B-cell excess in patients with monoclonal gammopathy of undetermined significance (MGUS): Association with malignant transformation. Br J Haematol 92:71, 1996

38. Österborg A, Steinitz M, Lewin N, Bergenbrant S, Holm G, Lefvert A-K, Mellstedt H: Establishment of idiotype bearing B-lymphocyte clones from a patient with monoclonal gammopathy. Blood 78:2642, 1991

39. Witzig T, Gonchoroff N, Katzmann J, Therneau T, Kyle R, Greipp P: Peripheral blood B-cell labeling indices are a measure of disease activity in patients with monoclonal gammopathies. J Clin Oncol 6:1041, 1988

40. Greipp P, Kyle R: Clinical, morphological, and cell kinetic differences among multiple myeloma, monoclonal gammopathy of undetermined significance, and smoldering multiple myeloma. Blood 62:166, 1983

41. Sahota S, Leo R, Hamblin T, Stevenson F: Immunoglobulin V (H) gene mutational patterns indicate different tumor cell status in human myeloma and MGUS. Blood 87:746, 1996

From www.bloodjournal.org by guest on March 3, 2016. For personal use only.

blood

1996 88: 289-296

Clonal circulating cells are common in plasma cell proliferative disorders: a comparison of monoclonal gammopathy of undetermined significance, smoldering multiple myeloma, and active myeloma

D Billadeau, B Van Ness, T Kimlinger, RA Kyle, TM Therneau, PR Greipp and TE Witzig

Updated information and services can be found at:
http://www.bloodjournal.org/content/88/1/289.full.html

Articles on similar topics can be found in the following Blood collections

Information about reproducing this article in parts or in its entirety may be found online at:
http://www.bloodjournal.org/site/misc/rights.xhtml#repub_requests

Information about ordering reprints may be found online at:
http://www.bloodjournal.org/site/misc/rights.xhtml#reprints

Information about subscriptions and ASH membership may be found online at:
http://www.bloodjournal.org/site/subscriptions/index.xhtml

Blood (print ISSN 0006-4971, online ISSN 1528-0020), is published weekly by the American Society of Hematology, 2021 L St, NW, Suite 900, Washington DC 20036.
Copyright 2011 by The American Society of Hematology; all rights reserved.
