# Supplementary material for: Expression of Concern: Prognostic value of circulating plasma cells in patients with multiple myeloma: A meta-analysis
Source: PLoS One. 2023 Feb 21;18(2):e0282230. doi: 10.1371/journal.pone.0282230 (PMC9942954; doi:10.1371/journal.pone.0282230)
Supplement: S1 File — (ZIP) [file pone.0282230.s001.zip › primary data/excluded research/1996 Concomitant mobilization of plasma cells and hematopoietic progenitors into peripheral blood of multiple myeloma patients_CD34+CPC.pdf]

From www.bloodjournal.org by guest on March 3, 2016. For personal use only.

Concomitant Mobilization of Plasma Cells and Hematopoietic Progenitors Into Peripheral Blood of Multiple Myeloma Patients: Positive Selection and Transplantation of Enriched CD34+ Cells to Remove Circulating Tumor Cells

By Roberto M. Lemoli, Alessandra Fortuna, Maria Rosa Motta, Simonetta Rizzi, Valeria Giudice, Anna Nannetti, Giovanni Martinelli, Michele Cavo, Marilina Amabile, Serena Mangianti, Miriam Fogli, Roberto Conte, and Sante Tura

One advantage of the use of peripheral blood stem cells (PBSCs) over autologous bone marrow would be a reduced risk of tumor cell contamination. However, the level of neoplastic cells in the PB of multiple myeloma (MM) patients after mobilization protocols is poorly investigated. In this study, we evaluated PB samples from 27 pretreated MM patients after the administration of high dose cyclophosphamide (7 g/m^2 or 4 g/m^2) and granulocyte-colony stimulating factor for the detection of myeloma cells as well as hematopoietic progenitors. Plasma cells containing intracytoplasmic Ig were counted by microscope immunofluorescence after incubation with appropriate antisera directed against light- and heavy-chain Ig. Moreover, flow cytometry studies were performed to determine the presence of malignant B-lineage elements by using monoclonal antibodies against the CD19 antigen and the monotypic light chain. Before initiation of PBSC mobilization, circulating plasma cells were detected in all MM patients in a percentage ranging from 0.1% to 1.8% of the mononuclear cell fraction (mean value, 0.7% ± 0.4% SD). In these patients, a higher absolute number of PB neoplastic cells was detected after chemotherapy and granulocyte colony-stimulating factor. Kinetic analysis showed a pattern of tumor cell mobilization similar to that of normal hematopoietic progenitors with a maximum peak falling within the optimal time period for the collection of PBSCs. The absolute number of plasma cells showed a 10- to 50-fold increase as compared with the baseline value. Apheresis products contained 0.7% ± 0.2% SD of myeloma cells (range, 0.2% to 2.7%). Twenty-three MM patients were submitted to PBSC collection. In 10 patients, circulating hematopoietic CD34$^+$ cells were highly enriched by avidin-biotin immunoabsorption, were cryopreserved, and used to reconstitute bone marrow function after myeloablative therapy. The median purity of the enriched CD34$^+$ cell population was 89.5% (range, 51% to 94%), with a 75-fold increase as compared with the pretreatment samples. The median overall recovery of CD34$^+$ cells and colony-forming unit–granulocyte-macrophage was 58% (range, 33% to 95%) and 45% (range, 7% to 100%), respectively. Positive selection of CD34$^+$ cells resulted in 2.5- to 3-log depletion of plasma cells and CD19$^+$ B-lineage cells as determined by immunofluorescence studies, although DNA analysis of CDR III region of IgH gene showed the persistence of minimal residual disease in 5 of 6 patient samples studied. Myeloma patients were reinfused with enriched CD34$^+$ cells after myeloablative therapy consisting of total body irradiation (1,000 cGy) and high-dose melphalan (140 mg/m^2). They received a median of 4 × 10^6 CD34$^+$ cells/kg and showed a rapid reconstitution of hematopoiesis; the median time to 0.5 × 10^9 neutrophils and to 20 and 50 × 10^9 platelets per liter of PB was 10, 11, and 12 days, respectively. These results, as well as other clinically significant parameters, did not significantly differ from those of patients (n = 13) receiving unmanipulated PBSCs after the same pretransplant conditioning regimen. In summary, our data show the concomitant mobilization of tumor cells and hematopoietic progenitors in the PB of MM patients. Positive selection of CD34$^+$ cells reduces the contamination of myeloma cells from the apheresis products up to 3-log and provides a cell suspension capable of restoring a normal hematopoiesis after a total body irradiation-containing conditioning regimen.
© 1996 by The American Society of Hematology.

MULTIPLE MYELOMA IS a B-cell–derived neoplastic disease that is generally associated with the expansion of mature plasma cells, monoclonal Ig production, and multiple osteolytic lesions.[1] Because of the limitations of conventional therapeutic approaches, several myeloablative radiochemotherapy regimens followed by the reinfusion of autologous stem cells have been proposed.[2-7] Those early trials have shown that the use of autologous stem cells has dramatically reduced the morbidity and mortality of high-dose therapy-related procedures. Moreover, the addition of total body irradiation (TBI) and the increase of the dose of melphalan to 200 mg/m^2 have resulted in over a 50% complete remission rate. Preliminary results from a randomized study have recently shown the superiority of autologous transplantation over conventional treatment for the achievement of complete remission and, perhaps, long-term survival in patients with multiple myeloma (MM).[8] However, these promising results do not clearly indicate the possibility of eradicating the neoplastic clone by using a single course of supralethal radiochemotherapy. In this regard, better results may be obtained by HLA-matched allogeneic bone marrow (BM) transplantation,[9] suggesting both the potential role of graft-versus-myeloma effect of allogeneic immunocompetent cells and the negative impact on relapse rate of neoplastic stem cells contaminating the autograft. In fact, it has been recently shown by gene-marking studies that autologous

From the Institute of Hematology "L. & A. Seràgnoli," University of Bologna and Immunohematology Service, Bologna, Italy.
Submitted January 19, 1995; accepted September 14, 1995.
Supported by Regione Emilia-Romagna, delibera 4243/91, by Progetto Finalizzato C.N.R. ACRO no. 93.02257.PF39, by the Associazione Italiana per la Ricerca contro il Cancro (AIRC) and MURST 60%.
Address reprint requests to Roberto M. Lemoli, MD, Institute of Hematology "L.& A. Seràgnoli", Via Massarenti 9, 40100 Bologna-Italy.
The publication costs of this article were defrayed in part by page charge payment. This article must therefore be hereby marked "advertisement" in accordance with 18 U.S.C. section 1734 solely to indicate this fact.
© 1996 by The American Society of Hematology.
0006-4971/96/8704-0034$3.00/0

From www.bloodjournal.org by guest on March 3, 2016. For personal use only.

grafts contain clonogenic tumor cells that contribute to relapse when reinfused into the patients.[10,11]

More recently, the use of chemotherapy-primed peripheral blood stem cells (PBSCs) and cytokines has shown a more rapid hematopoietic reconstitution than has the reinfusion of BM-derived hematopoietic cells, thus reducing the incidence of serious infections and virtually eliminating mortality.[12] Therefore, many investigators have placed emphasis on blood as an alternative source of autologous hematopoietic stem cells for autografting. However, tumor-related B cells, bearing the same idiotypic determinants of neoplastic plasma cells, have been identified in the blood of myeloma patients,[13,14] and they have been shown to be part of the neoplastic stem cell compartment.[15,16] Moreover, the level of circulating tumor cells in response to mobilization protocols, their relative frequency in relationship to that of normal hematopoietic progenitors, and the potential contamination of the leukaphereses are still poorly investigated. In this report, we used immunofluorescence assays to evaluate circulating neoplastic cells in patients with MM eligible for myeloablative radiochemotherapy and PBSC transplantation. Greater than 300 PB samples and leukapheresis products from 27 individuals treated with high-dose cyclophosphamide (Cy) and granulocyte colony-stimulating factor (G-CSF) were assessed for the detection of tumor cells as well as hematopoietic precursors. Moreover, we evaluated the ''indirect purging'' of myeloma cells provided by positive selection of circulating hematopoietic cells, characterized as $CD34^+$, and their capacity of reconstituting autologous hematopoiesis after a TBI-containing conditioning regimen.

Our results show the mobilization of tumor cells on recruitment of hematopoietic progenitor cells. However, positive selection of $CD34^+$ cells markedly reduces, but does not abrogates, myeloma cell contamination in the apheresis products and provides a cell fraction that can be safely used for restoring normal BM function after a myeloablative therapy.

PATIENTS AND METHODS

Clinical study. Twenty-seven consecutive individuals entered in the study. The diagnosis of MM was made by using standard criteria.[17] The protocol was approved by the University Hospital ethical committee, and each patient gave written informed consent.

Patients were treated with high-dose Cy (7 g/m^2, n = 23; or 4 g/m^2, n = 4) as described,[18] followed by the administration of G-CSF (Filgrastim; Neupogen, Dompè Biotec, Milan, Italy) at the dose of 5 g/kg/d subcutaneously starting on day 2 after chemotherapy and continued until the completion of PBSC collection. Once the $CD34^+$ cell count was greater than 20,000/mL of PB, patients underwent leukaphereses using a Baxter (Rome, Italy) CS 3000 plus blood cell separator using the modified procedure no. 1 program. The small volume collection chamber, with a median volume of 58 mL, was used to reduce extracorporeal volume and platelet (Plt) collection. A median of 9 L of PB was processed through a dual-lumen central venous catheter at a flow rate of 40 to 60 mL/min to obtain a yield greater than 2×10^6 $CD34^+$ cells/kg. The apheresis products (mean number per patient, 2; range, 1 to 3) showed a 95% pure mononuclear cell (MNC) fraction and resulted in a median overall recovery of 70% of MNCs and hematopoietic progenitor cells. PBSCs were cryopreserved, as previously described,[19] immediately after their collection or subsequent to positive selection of $CD34^+$ cells (see below).

Twenty-three MM patients underwent PBSC collection as described, and 10 had their circulating $CD34^+$ cells positively selected. Unmanipulated PBSC collections or $CD34^+$ cells were reinfused on day 0 after a conditioning regimen consisting of 140 mg/m^2 of melphalan (day −3) and 1,000 cGy TBI administered in single fraction (day −1). Five patients (PBSC cohort, 4; $CD34^+$ cells, 1) previously heavily irradiated on the spinal cord, received melphalan (200 mg/m^2; n = 4) or busulfan (16 mg/kg) and Cy (120 mg/kg; n = 1) as myeloablative chemotherapy, respectively. G-CSF at 5 g/kg/d subcutaneously was started at day +1 and administered until the granulocyte count reached greater than 0.5×10^9/L for 3 consecutive days. The primary end point of the clinical study was time to hematopoietic reconstitution, defined as the number of days to achieve a granulocyte count greater than 0.5×10^9/L and an unsupported Plt count of greater than 20 and 50×10^9/L. All patients received a single-donor Plt transfusion when the Plt count was less than 20×10^9/L and a red blood cell transfusion for a hemoglobin level less than 8 g/dL. Patients who achieved a complete or partial remission after transplantation received α-interferon (α-IFN) subcutaneously (3×10^6 IU/m^2 3 times a week) beginning at the time of full hematological recovery and continued until evidence of progressive disease.

Preparation of PB specimens. Serial samples of PB (n = 303) were analyzed. Each patient was assessed before chemotherapy and subsequently at least 3 times a week for the presence of hematopoietic progenitors and tumor cells in PB samples. A minimum of 6 specimens, in addition to the apheresis products, were studied for each patient from day −2 to day +20 after Cy chemotherapy.

Hematopoietic progenitor cell assay. Samples of PB were evaluated in tissue culture assay to determine myeloid progenitor cell growth as previously described.[20] Briefly, 1×10^5 low-density cells were plated in duplicate in culture medium consisting of 1 mL of Iscove's modified Dulbecco's medium (GIBCO-BRL, Paisley, UK), supplemented with 24% fetal calf serum (Sera Lab, Crawley Down, Sussex, UK), 0.8% bovine serum albumin (Sigma Chemical Co, St Louis, MO), 10^{-4} mol/L of 2-mercaptoethanol (Sigma), 2 U of human recombinant erythropoietin (Dompè Biotec, Milan, Italy), bovine hemin 0.2 mmol/L, and 10% (vol/vol) of a selected lot of phytohemagglutinin–lymphocyte-conditioned medium. Methylcellulose final concentration was 1.1%. Granulocyte-macrophage colony-forming unit (CFU-GM), burst-forming unit-erythroid, and colonies derived from pluripotent progenitors were scored after 14 days of incubation at 37°C in a fully humidified 5% CO_2 atmosphere. To determine the number of PB CFU-cells (CFU-C) per unit volume, the number of progenitor cells/10^5 MNCs was multiplied by the MNC count in the same sample.

Cell phenotype analysis. The percentage of $CD34^+$ and $CD19^+$ cells was determined by staining 5×10^5 MNCs with mouse-derived monoclonal antibodies (MoAbs) HPCA-2, IgG2a-fluorescein isothiocyanate (FITC), and Leu-12, IgG1-phycoerythrin (Becton Dickinson, San Jose, CA), respectively. Two additional anti-CD19 MoAbs were previously tested for comparison with Leu-12, B4 (Coulter Clone, Hialeah, FL) and a hybridoma supernatant kindly provided by P.L. Tazzari (Istituto Nazionale dei Tumori, IST, Genoa, Italy). Our results did not show any difference between the three MoAbs as for the percentage of B-cells in PB (data not shown). Double-staining of the CD19/monotypic light chain allowed the assessment of monoclonal B-lineage cells.[21]

Cells were resuspended in phosphate-buffered saline (PBS) containing 1% bovine serum albumin and sodium azide with the MoAbs for 20 minutes at 4°C, and respective normal IgG isotypes were used

From www.bloodjournal.org by guest on March 3, 2016. For personal use only.

as controls. Propidium iodide (2 g/mL; Sigma) was added for the detection of nonviable cells that were excluded from analysis. After 2 washes, flow-cytometric analysis was performed on a gated population set on scatter properties by using a FACScan equipment (Becton Dickinson). A minimum of 10,000 events were collected in list mode on FACScan software.

Intracytoplasmic Ig (cIg) and bromodeoxyuridine (BRDU) staining. Cells containing cIg were counted by immonofluorescence after incubation with appropriate goat antisera directed against light- and heavy-chain Ig coupled with FITC (working dilution, 1:40; Kallestad, Chaska, MN), as previously described.[22] The percentage of myeloma cells in S-phase was determined by the BRDU incorporation assay.[22] Briefly, 10 L of the stock BRDU solution (1 mmol/L) was added to the cell suspension for 30 minutes in 5% CO_2 at 37°C. After 2 washes, slides were obtained by cytocentrifugation, air-dried, fixed in a mixture of methanol and acetic acid (3:1), and denaturated in 0.07 mol/L NaOH for 12 seconds. For nuclear BRDU staining, the slides were first incubated with 5 L of anti-BRDU MoAb (Becton Dickinson) diluted 1:15 with PBS with 0.5% Tween 20 (Sigma) for 30 minutes at room temperature. Finally, they were treated with TRITC-conjugated antimouse Ig (Dako, Glostrup, Denmark) diluted 1:30 and antisera to light- and/or heavy-chain human Ig-FITC. Preparations were then washed overnight with PBS, and positive cells were scored with a fluorescence microscope. Large B cells bearing paranuclear "spots" of monoclonal light and heavy chain were also scored,[21] because the majority of these elements incorporate BRDU and have been reported as part of the proliferative compartment in MM.[23] For the calculation of labeling index, at least 100 cells staining positively for the same light-chain isotype as the patient's monoclonal (M) component were counted. Immunofluorescence staining results were confirmed by two independent observers. The sensitivity of this assays was previously established by mixing different concentrations of RPMI-8226 myeloma cells with a standard number of PB cells and was 1:10^4 cells.

Positive selection of CD34+ cells. One or two leukapheresis products were processed to positively select CD34+ cells,[24] whereas an additional apheresis was cryopreserved as unmanipulated backup. The first apheresis product was stored at room temperature overnight and pooled with the second before further processing. Briefly, PBSCs were incubated for 25 minutes at room temperature with 20 μg/mL of the biotinylated, anti-CD34 MoAb 12.8 in 150 mL of PBS containing 0.1% human serum albumin (HSA). The treated cells were washed with PBS on the Cobe Processor to remove the unbound antibody. This cellular fraction, diluted in 300 mL, was passed over the Ceprate SC Stem Cell Concentrator (Cell Pro, Inc, Bothell, WA), which contained a sterile column of avidin-coated polyacrylamide beads. After washing with 300 mL of PBS, the CD34+ cells were removed from the beads by mechanical agitation and eluted with 90 mL of PBS added with heparin and 4 mL of HSA. Aliquots of the CD34+ target cells and the unbound cells were analyzed to assess the percentage of CD34+ elements and the colony-forming ability in each cell fraction. The restaining was performed using an antibody (HPCA-2) directed toward a different epitope of CD34 antigen than that (12.8) used with the Ceprate Stem Cell Concentrator. After centrifugation, the cells were resuspended in PBS containing 7.5% dimethyl sulfoxide (DMSO) and 4% HSA to a final volume of 4.5 mL. The cells were then cryopreserved in one, or if greater than 100 × 10^6 cells, in two vials, using a controlled-rate freezing method and stored at −196°C.

At time of reinfusion, CD34+ cells were rapidly thawed in a water bath at 37°C and diluted by slowly adding, using a dropper, 4.5 mL of heparinized PBS to provide optimum viability and recovery of frozen cells. The cell suspension was further diluted with PBS to 30 mL of final volume for each vial and reinfused via a central line.

Analysis of minimal residual disease (MRD) by DNA amplification of CDR III region of IgH gene. MRD was analyzed by IgH gene amplification as previously decribed.[25] High molecular weight DNA was extracted from BM samples taken at diagnosis and from leukapheresis collections obtained before and after positive selection of CD34+ cells by proteinase-K digestion followed by phenol chloroform extraction as already reported.[26] Oligonucleotides were synthesized by an Applied Biosystems DNA synthesizer model 394 (Applied Biosystems, Milano, Italy). Sequences of the consensus VH primer 5′-CCGAGGACACGGCCGTGTATTACTG-3′ and JH consensus primer 5′-AACTGCTGAGGAGACGGTGACC -3′ are based on CDR III region VH genes[27,28] and 3′ ends of JH genes. Amplification was performed essentially as described.[25,26] Briefly, 0.1 to 0.4 g of genomic DNA was added to 200 mol/L of each deoxynucleotide and 200 pmol of each amplimer. A total of 2.5 U of Taq polymerase (Perkin Elmer-Cetus, Roche, Italy) was added in a 100 L of 1× polymerase chain reaction (PCR) buffer. The reaction was overloaded with 15 L of mineral oil (Perkin Elmer-Cetus). PCR is a process covered by patent of Hoffman-LaRoche (Basel, Switzerland). Particular conditions were taken to avoid false results or contamination.[29] Normal BM DNA was always analyzed in parallel with test DNAs as a control for contamination of reagents of PCR products. A total of 35 cycles of amplification were performed with a DNA thermal cycler 480 (Perkin Elmer-Cetus). The PCR conditions used were as follow: denaturation at 96°C for 1 minute, primer annealing at 58°C for 1 minute, and then chain elongation at 72°C for 1 minute. A total of 18 L of the PCR product was separated in 1.5% agarose gels (BioRad, Milan, Italy) containing 0.05 g/mL of ethidium bromide and was photographed with Polaroid 667 film (Polaroid Corp, Milan, Italy). The presence of a clonal rearrangement is indicated by a dense band, with or without a background ladder, of differently sized products generated by polyclonally rearranged B cells. The exact size, ranging between 100 to 150 bp, of the clone-specific amplification product in a particular patient can be used to follow MRD. To establish the sensitivity of our method of detection of the IgH gene rearrangement (1:10^4), DNA from a heavily infiltrated MM sample was serially diluted with DNA from normal BM MNCs before PCR amplification. Furthermore, to improve electrophoretic size separation of the bands, 20 L of the amplified product was electrophoresed on an 8% nondenaturing polyacrylamide gel and run to 250 V in a refrigerated (4°C) buffer (1× TBE; Protean II; BioRad). After the run, the gels were soaked with ethidium bromide, photographed at the UV light, and then were subjected to silver-staining (Silver Stain Kit; BioRad) and further photographed. The molecular weight marker (MWM) used in the electrophoretic separation was MWM V and VI from Boehringer Mannheim (Mannheim, Germany).

Statistical analysis. The results are expressed as the mean standard deviation (SD) unless otherwise indicated. Statistical analysis was performed by mean of the nonparametric paired Wilcoxon rank sum test.

RESULTS

Mobilization of tumor cells and hematopoietic progenitor cells. The clinical characteristics of study patients (n = 27) are reported in Table 1. All individuals had received one or more lines of treatment before high dose Cy, and none of them was in complete remission at time of study. Of 23 transplanted patients, 18 (78%) had been treated with a median of 6 cycles of an alkylating agent-containing regimen (mostly melphalan and prednisone); 16 patients (70%) had received a median of 3 cycles of VAD (vincristine, doxorubi-

From www.bloodjournal.org by guest on March 3, 2016. For personal use only.

Table 1. Study Population

Characteristics	No. of Patients
Median age in years (range)	47.5 (29-55)
Sex (M/F)	18/9
Tumor stage*	
I	6
II	5
III	16
M component	
IgG	15
IgA	4
BJ	5
Nonsecretory	3
Light chain	
λ	10
κ	17
Renal insufficiency	
Yes	5
No	22
Previous therapy	
Alkylating agents	22
VAD or VAD-like therapy	18
VAD + alkylating agents	13
α-IFN	5

* According to Durie-Salmon classification.

cin, and dexamethasone) or VAD-like therapy, and 12 individuals (52%) received both. Five patients were enrolled in the study after they had relapsed or progressed during maintenance treatment with α-IFN.

Before initiation of PBSC mobilization, circulating plasma cells were detected in all MM patients, with a mean value of 0.7% ± 0.4% (range, 0.1% to 1.8%) of MNC fraction (Table 2). No plasma cells were actively proliferating as judged by the BRDU-incorporating assay. On Cy chemotherapy and G-CSF–induced mobilization protocol, 0.8% ± 0.6% plasma cells (range, 0.2% to 2.2%) were assessed in over 300 PB samples examined. Moreover, because of the increased leukocyte count, the absolute number of tumor cells showed a 10- to 50-fold increase as compared with the baseline value. Over the same period of time, S-phase plasma cells were detected in 4 patients, with a mean value of 8.2% ± 3% BRDU$^+$ cells (Table 2). Kinetic analysis showed a pattern of tumor cell mobilization similar to that of normal hematopoietic progenitor cells, with a maximum peak falling within the optimal time period for the collection of PBSCs. Figure 1 shows representative examples of concomitant mobilization of plasma cells, CD34$^+$ cells, and CFU-GM in 4 MM patients treated with 7 g/m^2 (Fig 1A, B, and D) or 4 g/m^2 (Fig 1C) of Cy. Apheresis products from 23 patients contained 0.7% ± 0.2% of plasma cells (range, 0.2% to 2.7%; see Table 2). It is noteworthy that mobilized plasma cells were positive for either κ or λ, but not for both, and the light chain expressed was the same as that for the monoclonal serum Ig or urin protein.

In the PB of MM patients, we also analyzed the percentage, the absolute number, and the kinetics of CD19$^+$ B-cell elements bearing the monoclonal light chain. Conversely to mature plasma cells, we did not observe a mobilization pattern of CD19$^+$ cells and their absolute number increased only in 2 patients, whereas in some individuals they tend to decrease to the lower limit of detection of our immunofluorescence analysis (Table 2). The percentage of CD34$^+$/CD19$^+$ pre-B cells was always lower than 0.1% (data not shown).

Ex vivo purging of tumor cells by positive selection of CD34$^+$ cells. Twenty-three MM patients showed a good mobilization of hematopoietic progenitor cells in the PB and were submitted to PBSC collection. Contamination of neoplastic plasma cells and B cells for the whole group is shown in Table 2. Among that cohort of patients, 10 had their circulating CD34$^+$ cells purified by the Ceprate SC concentrator, and the removal of tumor cells is reported in Table 3. Using microscope immunofluorescence and flow cytometry analysis, a reduction of 99.7% ± 0.6% and 99.8% ± 0.3% of plasma cells and CD19$^+$ cells, respectively, was documented after positive selection. Interestingly, the percentage of plasma cells and B cells decreased after positive selection from 0.7% ± 0.4% to 0.1% ± 0.07% and from 1.6% ± 0.5% to 0.4% ± 0.2%, respectively ($P < .03$). This calculation indicated that tumor cells did not merely behave as "innocent bystanders" during the CD34$^+$ cells purification process, and approximately 1-log depletion of myeloma cells was caused by the procedure. An additional 2-log purging resulted from the overall recovery of only 0.8% of MNCs.

However, consistent with immunofluorescence studies, MRD was observed after stem cell purification in 5 of 6 patient samples evaluated by PCR analysis of IgH gene rearrangement (Fig 2). The original clonal bands, identical in size to those observed in diagnostic BM aspirations, were also found in all the leukaphereses (Fig 2).

PBSC processing data and engraftment results. The recovery of hematopoietic progenitor cells is reported in Table 4. The median number of MNCs processed was 6.1 × 10^8/kg with a median of 1.2% CD34$^+$ cells. After positive selection, the median MNCs, CD34$^+$ cells, and CFU-GM recovery was 0.8%, 58%, and 45%, respectively. The median purity of CD34$^+$ cell population was 89.5%, with a 75-fold increase as compared with that of the pretreatment samples. We found an inverse correlation between the degree of previous treatment, especially administration of alkylating agents,

Table 2. Mobilization of Myeloma Cells After Cy and G-CSF

	% PC	% S-Phase PC	% CD19$^+$ Cells
Start	0.7 ± 0.4 (0.1-1.8)	0	2.4 ± 2.2 (0-5.5)
Mobilization time period	0.8 ± 0.6 (0.2-2.2)	8.2 ± 3 (4.5-11.1)*	1 ± 0.5 (0.3-1.4)
Aphereses	0.7 ± 0.2 (0.2-2.7)	10 ± 5 (4-11)*	1.4 ± 0.5 (0.9-2.2)

The results are expressed as mean ± SD (range).

Abbreviation: PC, plasma cells.

* The results refer to the patients (n = 4) who showed circulating BRDU$^+$ plasma cells after Cy and G-CSF.

From www.bloodjournal.org by guest on March 3, 2016. For personal use only.

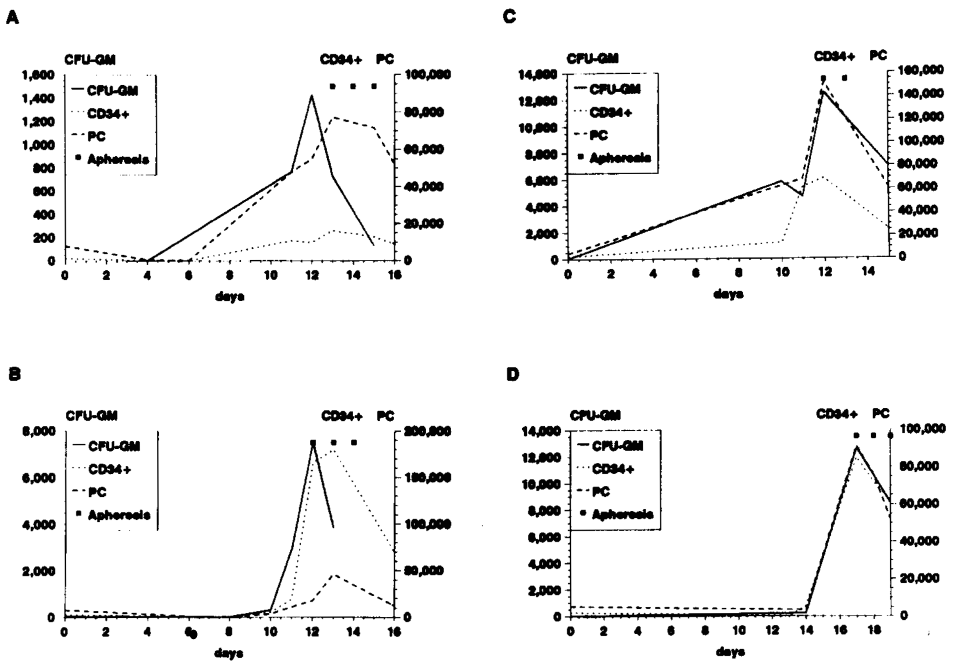

Fig 1. Concomitant mobilization of CFU-GM, CD34⁺ cells and plasma cells after high-dose Cy (7 g/m² for A, B, and D; 4 g/m² for C) and G-CSF in 4 MM patients. The data are presented as the number of hematopoietic progenitors or tumor cells per milliliter of PB.

and the recovery of CD34⁺ cells and CFU-GM. Only patients extensively pretreated required two leukaphereses to obtain greater than 1 × 10⁶ CD34⁺ cells, which was considered the threshold dose to achieve a sustained engraftment.

Myeloma patients were reinfused with a median of 4 × 10⁶ purified CD34⁺ cells/kg and 5.9 × 10⁴ CFU-GM/kg and showed a rapid reconstitution of BM function (Fig 3). The total volume of the enriched cell population ranged between 30 and 60 mL, and no sign of toxicity because of infusion of purified CD34⁺ cells was observed. Engraftment and supportive-care data of the study patients are reported in Table 5 and compared with those of 13 patients who received unmanipulated PBSCs after a TBI-containing conditioning regimen. The two series of patients are comparable as for prior chemotherapy, number of cells collected from the PB, number of leukaphereses, and CFU-GM reinfused ($P > .1$). Both cohort of patients achieved granulocyte engraftment in a median of 10 days and an unsupported Plt count greater than 20 × 10⁹/L in a median of 15 and 11 days, respectively ($P > .1$). The median time to reach greater than 50 × 10⁹ Plt/L was 12 and 18 days for CD34⁺ cells and PBSC patients, respectively. One patient in each group did not reach Plt recovery (both had been reinfused with less than 2 × 10⁶ CD34⁺ cells/kg) and the patient who had received purified

Table 3. Ex Vivo Purging of B-Lineage Cells and Plasma Cells by Positive Selection of CD34⁺ Cells

	PC					CD19⁺ Cells				
Patient No.	Pre (×10⁶)	%	Post (×10⁶)	%	% Purging	Pre (×10⁶)	%	Post (×10⁶)	%	% Purging
1	189	0.7	0.4	0.2	99.8	540	2	1	0.5	99.8
2	591	0.9	0.7	0.3	99.9	985	1.5	0.5	0.2	99.9
3	337	0.9	1	0.3	99.5	487	1.3	1	0.3	99.8
4	162	0.41	0.5	0.1	99.7	356	0.9	1	0.2	99.7
5	544	1.1	0.8	0.2	99.8	300	2.5	1.5	0.6	99.9
6	288	0.5	1.7	0.1	99.4	979	1.7	0.8	0.05	99.9
7	40	0.2	0.2	0.1	99.6	258	1.2	1.4	0.8	99.5
8	172	0.53	0.1	0.1	99.9	162.5	0.5	0.37	0.4	99.7
9	178	0.62	0.7	0.02	99.6	460.8	1.6	1.5	0.1	99.7
10	591	1.5	0.93	0.2	99.8	1,260.8	3.2	3.2	0.7	99.7
Mean ± SD	309.2 ± 200 (0.7 ± 0.4)		0.7 ± 1 (0.1 ± 0.07)		99.7 ± 0.6	579 ± 367.8 (1.6 ± 0.5)		1.3 ± 1 (0.4 ± 0.2)		99.8 ± 0.3

B-lineage elements and terminally differentiated PC were evaluated by immunofluorescence before (Pre) and after (Post) positive selection of CD34⁺ cells.

Abbreviation: PC, plasma cells.

From www.bloodjournal.org by guest on March 3, 2016. For personal use only.

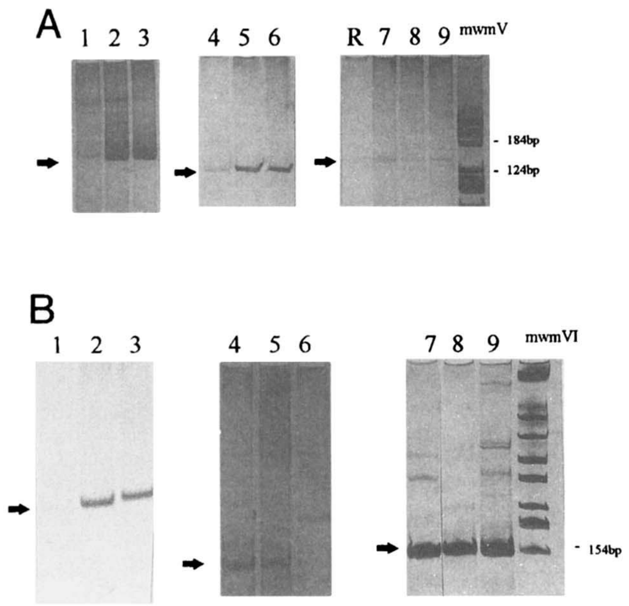

Fig 2. Analysis of amplified DNA samples obtained from 6 MM patients (A and B). Clonal bands of different size corresponding to patient-specific PCR IgH products were detected at diagnosis from BM (full arrows in lanes 3, 6, and 9, respectively). The same bands were observed in the leukapheresis collections (lanes 2, 5, and 8) and in the CD34⁺cell fractions (5 samples; lanes 1, 4, and 7) for each patient, respectively. R, negative control of PCR reagents; mwmV, molecular weight marker V and VI with expected size in base pair signed on the right side of the photograph.

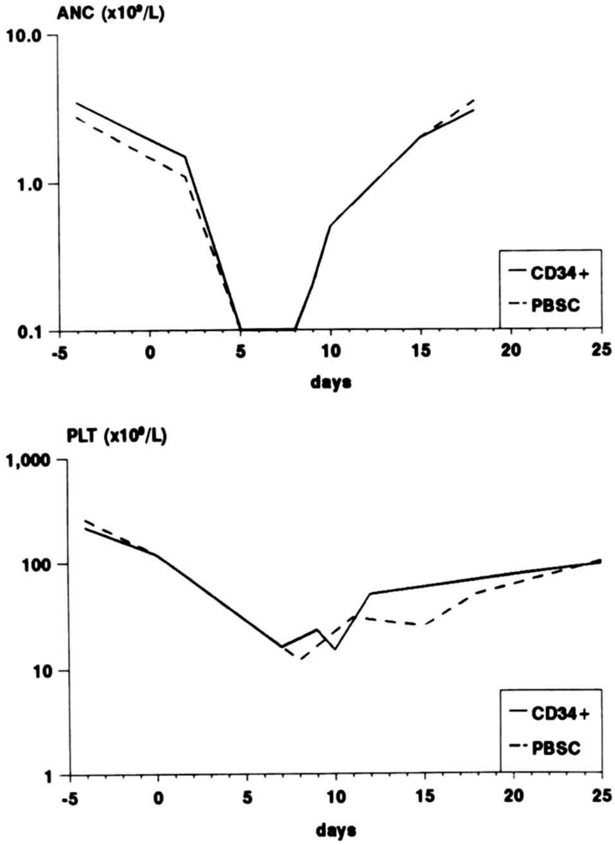

Fig 3. Hematopoietic engraftment rate after TBI and melphalan administration, followed by reinfusion of purified $CD34^+$ cells or unmanipulated PBSCs and G-CSF. The data are presented as the median absolute neutrophil count and the Plt count, respectively.

$CD34^+$ cells died in the peritransplant period because of interstitial pneumonia. Other clinical parameters, such as the length of hospitalization and transfusions requirement (Table 5), number of febrile days, documented infections, use of intravenous (IV) antibiotics were not different between the two groups of patients (data not shown). No individuals in the $CD34^+$ cells cohort received backup PBSCs or required Plt transfusion after discharge from the hospital.

With a median time from reinfusion of 12 months, we have not observed thus far any late graft failure in patients who had received purified $CD34^+$ cells. All but 1 patient reinfused with $CD34^+$ cells are actually being treated with 3 IU/m^2 of α-IFN 3 times a week as maintenance therapy.

DISCUSSION

The use of circulating hematopoietic stem cells offers several advantages over BM stem cells including a faster recovery of hematopoiesis.[12,18] Moreover, PBSC collections are generally believed to have a lower incidence of tumor involvement than BM harvest in cancer patients elegible for autografting. Consequently, PBSC transplantation is being used at many centers after myeloablative therapy for the treatment of MM patients.[5,7] However, recent reports indicate that steady-state circulating myeloma cells may play a crucial role in the pathogenesis of the disease.[13-16] These cells show a heterogeneous phenotype and differentiation stage and may represent a stem cell population in myeloma.[16] Moreover, the increase of circulating solid tumor cells on recruitment of PBSCs has been recently shown.[30,31] Thus, the question arises as to whether the mobilization of PBSCs

Table 4. Apheresis Products Processing Data (n = 10)

Cellular Fraction	MNC/kg	$CD34^+$/kg	% Purity	CFU-GM/kg
Pretreatment	6.1×10^8 (3.1-10.2)	6.7×10^6 (3.5-46.4)	1.2 (0.5-10.8)	27.5×10^4 (6.9-116.7)
Posttreatment	5.6×10^6 (1.9-26.5)	4.8×10^6 (1.3-24.9)	89.5 (51-94)	15.5×10^4 (2-64.7)
Reinfusion	4.6×10^6 (1.6-9.3)	4×10^6 (1.5-8.9)	88.5 (70-96)	5.9×10^4 (0.7-52.5)
% Recovery	0.8 (0.2-5.4)	58 (33-95)		45 (7-100)

The results are expressed as median (range).

From www.bloodjournal.org by guest on March 3, 2016. For personal use only.

results in the concomitant increase of neoplastic cells in the PB of MM patients. To this end, the aims of the present study were threefold: (1) assessment of the kinetic of myeloma cells after chemotherapy and G-CSF–induced mobilization protocol and the potential contamination of the leukaphereses; (2) evaluation of the indirect purging of neoplastic cells provided by positive selection of hematopoietic CD34$^+$ cells; and (3) the ability of the selected cell fraction to restore autologous hematopoiesis after a myeloablative radiochemotherapy.

The results presented here indicate that, in all patients with PB tumor cells under steady state conditions, the concomitant mobilization of plasma cells and hematopoietic progenitors occurs. It is noteworthy that the maximum peak of neoplastic mature plasma cells and B-lineage preplasma cells, as determined by immunofluorescence studies, fell within the optimal time period for collection of circulating CD34$^+$ cells, and leukapheresis products were contaminated, in some cases, by a higher number of myeloma cells than hematopoietic progenitors. Conversely, as determined by intracytoplasmic Ig immunostaining, the number of normal polyclonal plasma cells in the PB did not increase, indicating that the mobilization process may be specific to the abnormal clone. Moreover, actively proliferating neoplastic elements, with an elevated labeling index, were detected in 4 patients indicating the activation of the proliferation process in response to chemotherapy and G-CSF. The relative contribution of Cy and the growth factor in this respect is still unclear. Three additional MM patients receiving G-CSF alone showed a kinetic of tumor cells comparable with that of the patients included in this study (data not shown). Similarly, Gazitt et al[32] and Corradini et al[33] showed the consistent contamination of myeloma cells in PBSC collections (up to 10% of total cells)[32] after Cy and GM-CSF. Possibly, the use of growth factors for stem cell mobilization, with or without chemotherapy, may alter the expression of adhesion molecules associated with myeloma cell membrane. Although the biological and prognostic significance of tumor cells present into PBSC collections is still unknown and circulating plasma cells may primarly reflect advanced stages of the disease and the nature of the relapse may be caused by the regrowth of residual clonogenic cells in vivo, recent studies clearly show that reseeding of reinfused malignant cells contributes to relapse.[10,11] Therefore, we attempted to remove myeloma cells from PBSC autografts by positive selection of hematopoietic CD34$^+$ cells.

Effective ex vivo purging of contaminating myeloma cells with pharmacological and/or immunologic methods may result in the depletion of normal BM progenitor cells, thus delaying engraftment and increasing the risk of myelosuppression-related complications.[6,34] Therefore, enrichment of hematopoietic CD34$^+$ cells may provide an alternative approach for tumor-cell removal with a limited loss of normal stem cells. The CD34 antigen is a 110- to 120-kD glycoprotein that is expressed primarly on the earliest identifiable precursor cells and committed myeloid progenitors.[35-37] In normal individuals, CD34$^+$ cells represent 1% to 4% of the MNCs in the BM, whereas they are barely detectable in the PB. Immunophenotyping studies have been performed to test the reactivity of several anti-CD34 antibodies with malignant cells.[38,39] These studies have shown that the CD34 antigen is not expressed on tumor cells from patients with lymphomas and most solid tumors including breast cancer and neuroblastoma. In addition, the CD34 antigen was not found on the surface of mature plasma cells in MM, although the possibility that this glycoprotein may be present on clonally less differentiated B-lymphocytes is still matter of debate. In fact, the phenotypic and functional characteristics of the earliest precursors of myeloma cell population are still undefined, as is the target cell of malignant transformation. Whereas recent data support the hypothesis that MM originates at the later stages of B-cell differentiation[40,41] when B cells have lost the CD34 antigen,[42] other studies found CD34$^+$ cells to be part of the neoplastic clone.[40] However, it should be pointed out that reverse transcription-PCR, which has been used to detect MRD in some studies,[40,42] is an extremely sensitive technique, and the potential contamination of the CD34 cell fraction by unwanted cells should be carefully avoided. In this regard, Vescio et al[42] did not find IgH gene clonal rearrangement in collections of 99.99% pure CD34$^+$ cells obtained after using the combination of two methods of purification (immunoabsorption and fluorescence-activated cell sorting). Therefore, the presence of the CD34 antigen on hematopoietic progenitors and its absence on mature myeloid cells, lymphocytes, and plasma cells suggests that anti-CD34 antibodies may be useful clinically in isolating hematopoietic precursors for transplantation in myeloma patients.[43] Furthermore, reinfusion of autologous CD34$^+$ cells has been proven to reconstitute normal hematopoiesis in cancer patients treated with high-dose chemotherapy.[24,44-46]

In this report, using rigorously quantitative immunofluorescence assays, we showed that positive selection of CD34$^+$ cells induced the removal of 2.5- to 3-log of both mature

Table 5. Hematologic Reconstitution and Supportive-Care Data

	ANC >0.5 × 10^9/L	Plt >20 × 10^9/L	Plt >50 × 10^9/L	Plt Transfusions	RBC Transfusions	Hospital Discharge
Unmanipulated PBSCs	10 (9-12)	15 (8-NR)	18 (12-NR)	2 (0-2)	2 (0-10)	15 (11-31)
CD34$^+$ cells	10 (9-13)	11 (10-NR)	12 (11-NR)	2.5 (0-7)	0.5 (0-7)	17 (11-22)

The results are expressed as median (range). The conditioning regimen consisted of melphalan at 140 mg/m^2 and 1,000 cGy in single dose for both cohorts of patients. G-CSF treatment (5 μg/kg/d) was started at day +1 after reinfusion of autologous stem cells and was discontinued when the ANC was more than 0.5 × 10^9/L for 3 consecutive days.

Abbreviations: NR, not reached; ANC, absolute neutrophil count; RBC, red blood cell.

From www.bloodjournal.org by guest on March 3, 2016. For personal use only.

plasma cells and B-lineage lymphocytes. The cIg/BRDU double immunofluorescence methodology is highly specific and has been widely used for detecting BM[47] or circulating[48] resting/proliferating monoclonal B elements and plasma cells. CD19$^+$ cells, carrying the monotypic light chain,[21] were evaluated on previous studies, the results of which suggest that lesser differentiated cells of the malignant clone may represent a major reservoire of drug-resistant cells capable of inducing fatal relapse in MM patients.[14,16] The degree of myeloma-cell–purging shown in this report is consistent with earlier studies showing the capacity of avidin-biotin immunoabsorption technique to remove 3-log of normal and neoplastic lymphoid cells (CD34$^-$) from the BM and PB, coupled with a substantial recovery of hematopoietic precursors.[49-51] Recently, selection of hematopoietic CD34$^+$ cells has shown the capacity of purging 2.7- to 4.5-log of circulating myeloma cells by means of highly specific PCR analysis of clonally rearranged Ig gene using patient-specific primers.[43] However, it is very likely that the magnitude of initial tumor cell contamination may influence the purging efficiency. In the present study, the number of myeloma cells before stem cell selection was in the range of 10^8 (Table 3), whereas Schiller et al[43] reported a much lower tumor cell infiltration (range, 1.13×10^4 to 2.14×10^6 cells/kg), and MRD remained only in those specimens (3 of 14) heavily contaminated with myeloma. The persistence of myeloma cells in the CD34$^+$ cell fraction in this report, as shown in some cases by qualitative PCR analysis of IgH gene (sensitivity, 1:10^4 cells; see Fig 2), and in other reports[32,43] indicates that an additional purging step may be necessary to achieve a virtually tumor-free autograft.[50] In this regard, studies to optimize myeloma cell depletion by immunomagnetic beads or cell-sorting are currently underway in our own and other laboratories.[32] As stated above, the clinical impact of purging with respect to relapse of disease remains to be determined in this cohort of patients and in future randomized trials.

This clinical study also provides the evidence that purified CD34$^+$ progenitors are capable of restoring BM function in MM patients treated with a TBI-containing regimen. The median time to granulocyte and platelet engraftment of 10 and 11 days, respectively, is significantly shorter than that of a historical control group of patients receiving purged or unpurged BM cells.[34] Furthermore, the engraftment rate in this study is superimposable to that of patients transplanted with unmanipulated PBSCs (Table 5 and Fig 3). Although this was not a randomized trial, the sequentially treated cohorts of patients were comparable with respect to age, prior therapy, stage of the disease, number of MNCs collected and CFU-GM reinfused, conditioning regimen, and G-CSF administration. To date, there have not been late granulocyte or Plt engraftment failure in these patients, despite maintenance treatment with α-IFN (median follow-up, 12 months). In addition, we have been conducting a parallel pilot trial on positive selection and transplantation of BM-derived CD34$^+$ cells in resistant, relapsed lymphoma patients (Lemoli et al, manuscript submitted). The results of both stem cell enrichment (median purity of reinfused CD34$^+$ cells, 85.5%; enrichment factor, 131-fold) and the hematopoietic recovery (median time to neutrophil and Plt recovery, 14 and 20 days, respectively) confirmed the high degree of stem cell purification reported here and in previous reports[24,44-46] and the capacity of such cell fraction to reconstitute a stable hematopoiesis. Thus, the rapid and sustained engraftment determined by reinfusion of an average of 4×10^6 circulating CD34$^+$ cells/kg and the documented depletion of tumor cells shows that positive selection produces an MM-purging effect without apparent loss of engraftment potential.

In summary, this report shows the mobilization of myeloma cells along with hematopoietic progenitor cells after Cy and G-CSF. Because Cy and cytokines (either G-CSF or GM-CSF) are widely used for PBSC collection, clinical investigators should carefully evaluate the timing of stem cells harvest and/or the use of purging techniques. To this end, positive selection of CD34$^+$ cells is able to remove up to 3-log of contaminating tumor cells from the grafts and provides a cell suspension that can be safely used as supportive therapy for patients undergoing a truly myeloablative conditioning regimen. These results may be also relevant in view of future trials involving ex vivo expansion of hematopoietic progenitor cells, gene-marking experiments, and transplantation of allogeneic, T-cell–depleted, purified stem cells.

REFERENCES

1. Barlogie B, Epstein J, Salvanayagam P, Alexanian R: Plasma cell myeloma: New biological insights and advances in therapy. Blood 73:865, 1989

2. Jagannath S, Barlogie B, Dicke Ka, Alexanian R, Zagars G, Cheson B, LeMaistre FC, Smallwood L, Pruitt K, Dixon DO: Autologous bone marrow transplantation in multiple myeloma: Identification of prognostic factors. Blood 76:1860, 1990

3. Harousseau JM, Milpied N, Laporte JP, Collombat P, Facon T, Tigaud JD, Casassus P, Guilhot F, Ifhra N, Gandhour C: Double intensive therapy in high risk multiple myeloma. Blood 79:2827, 1992

4. Cunningham D, Paz-Ares L, Milan S, Powles R, Nicolson M, Hickish T, Selby P, Treleavan J, Viner C, Malpas J, Slevin M, Findlay M, Raymond J, Gore ME: High-dose melphalan and autologous bone marrow transplantation as consolidation in previously untreated myeloma. J Clin Oncol 12:759, 1994

5. Fermand JP, Chevert S, Ravaud P, Divine M, Leblond V, Dreyfus F, Mariette X, Brouet JC: High dose chemoradiotherapy with autologous blood stem cell transplantation in multiple myeloma. Results of a phase II trial involving 63 patients. Blood 82:2005, 1993

6. Anderson KC, Andersen J, Soeffer R, Freedman AS, Rabinowe SM, Robertson MJ, Spector N, Blake K, Murray C, Freeman A, Coral F, Marcus KC, Mauch P, Nadler LM, Ritz J: Monoclonal antibody-purged bone marrow transplantation therapy for multiple myeloma. Blood 82:2568, 1993

7. Vesole DH, Barlogie B, Jagannath S, Cheson B, Tricot G, Alexanian R, Crowley J: High-dose therapy for refractory multiple myeloma: Improved prognosis with better supportive care and double transplant. Blood 84:950, 1994

8. Attal M, Harousseau JL, Stoppa AM, Sotto JL, Fuzibet G, Rossi JF, Casassus P, Thyss A, Maisonneuve H, Facon T, Ifrah N, Payen C, Bataille R: High-dose therapy in multiple myeloma: A prospective randomized study of the "Intergroupe francais du myelome" (IFM). Blood 84:386a, 1994 (abstr, suppl 1)

From www.bloodjournal.org by guest on March 3, 2016. For personal use only.

9. Cavo M, Benni M, Cirio TM, Gozzetti A, Tura S: Allogeneic bone marrow transplantation for the treatment of multiple myeloma. An overview of published reports. Stem Cells 13:126, 1995 (suppl 2)

10. Brenner MK, Rill DR, Moen RC, Krance RA, Mirro J, French Anderson W, Ihle JN: Gene-marking to trace the origin of relapse agter autologous bone-marrow transplantation. Lancet 341:85, 1993

11. Deisseroth AB, Zu Z, Claxton D, Hanania EG, Fu S, Ellerson D, Goldberg L, Thomas M, Janicek K, French Anderson W, Hester J, Korbling M, Durett A, Moen R, Berenson R, Heimfeld S, Hamer J, Calvert L, Tibbits P, Talpaz M, Kantarjian H, Champlin R, Reading C: Genetic marking shows that Ph^+ cells present in autologous transplants of chronic myelogenous leukemia (CML) contribute to relapse after autologous bone marrow in CML. Blood 83:3068, 1994

12. To LB: Is our current strategy in manipulating hemopoiesis in autologous transplantation correct? Stem Cells 11:283, 1993

13. Berenson J, Wong R, Kim K, Brown N, Lichstentein A: Evidence of peripheral blood B lymphocyte but not T lymphocyte involvement in multiple myeloma. Blood 70:1550, 1987

14. Pilarski LM, Jensen GS: Monoclonal circulating B cells in multiple myeloma: A continuosly differentiating possibly invasive population as defined by expression of CD45 isoforms and adhesion molecules. Hematol Oncol Clin North Am 6:297, 1992

15. Bergui L, Schena M, Gaidano GL, Riva M, Caligaris-Cappio F: Interleukin 3 and interleukin 6 synergistically promote the proliferation and differentiation of malignant plasma cell precursors in multiple myeloma. J Exp Med 170:613, 1989

16. Pilarski LM, Belch AR: Circulating monoclonal B cells expressing P glycoprotein may be a reservoir of multidrug-resistant disease in multiple myeloma. Blood 83:724, 1994

17. Chronic Leukemia-Myeloma Task force, National Cancer Institute: Proposed guidelines for protocol studies-II. Plasma cell myeloma. Cancer Chemother Rep (part 3) 4:145, 1973

18. Gianni AM, Bregni M, Stern A, Siena S, Tarella C, Pileri A, Bonadonna G: Granulocyte-macrophage colony-stimulating factor to harvest circulating haemopoietic stem cells for autotransplantation. Lancet 2:580, 1989

19. Benini C, Bandini G, Motta MR, Belardinelli AR, Calori E, Rizzi S, Martinelli G, Rosti G, Trabetti E, Pignatti P, Tura S: Donor origin of hematopoiesis after a case of allogeneic transplantation with cryopreserved marrow. Haematologica 78:414, 1993

20. Lemoli RM, Gasparetto C, Scheinberg DA, Moore MAS, Clarkson BD, Gulati SC: Autologous bone marrow transplantation in acute myelogenous leukemia: In vitro treatment with myeloid-specific monoclonal antibodies and drugs in combination. Blood 77:829, 1991

21. Bergsagel LP, Masellis-Smith A, Szczepek A, Mant MJ, Belch AR, Pilarski LM: In multiple myeloma, clonotypic B lymphocytes are detectable among $CD19^+$ peripheral blood cells expressing CD38, CD56, and monotypic Ig light chain. Blood 85:436, 1995

22. Lemoli RM, Fortuna A, Grande A, Gamberi B, Bonsi L, Fogli M, Amabile M, Cavo M, Ferrari S, Tura S: Expression and functional role of c-kit ligand (SCF) in human multiple myeloma. Br J Haematol 88:760, 1994

23. Visani G, Lemoli RM, Dinota A, Galieni P, Gobbi M, Cavo M, Tura S: Evidence that long-term bone marrow culture of patients with multiple myeloma favours normal hemopoietic proliferation. Transplantation 48:1026, 1989

24. Shpall EJ, Jones RB, Bearman SI, Franklin WA, Archer PG, Curiel T, Bitter M, Claman HN, Stemmer SM, Purdy M, Myers SE, Hami L, Taffs S, Heimfeld S, Hallagan J, Berenson RJ: Transplantation of enriched CD34-positive autologous marrow into breast cancer patients following high-dose chemotherapy: Influence of CD34-positive peripheral-blood progenitors and growth factors on engraftment. J Clin Oncol 12:28, 1994

25. Brisco MJ, Tan LW, Orsborn AM, Morley AA: Development of a higly sensitive assay, based on the polymerase chain reaction, for rare B-lymphocyte clones in a polyclonal population. Br J Haematol 75:163, 1990

26. Martinelli G, Buzzi M, Zaccaria A, Mantovani V, Farabegoli P, Calori E, Bandini G, Bragliani M, Barboni P, Panzica G, Tura S: New strategies for selection of unrelated bone marrow donors. Bone Marrow Transplant 11:31, 1993

27. Tomlinson IM, Walter G, Marks JD, Llewelyn MB, Winter G: The repertoire of human germline VH sequences reveals about fifty groups of VH segments with different hypervariable loops. J Mol Biol 227:776, 1992

28. Ravetech JV, Siebenlist U, Korsmeyer S, Waldman T, Leder P: Structure of the human immunoglobulin locus: Characterisation of embrionic and rearranged J and D genes. Cell 27:583, 1981

29. Kwok S, Higuchi R: Avoiding false positives with PCR. Nature 339:237, 1989

30. Ross AA, Cooper BW, Lazarus HM, Mackay W, Moss TJ, Ciobanu N, Talmann MS, Kennedy JM, Davidson NE, Sweet D, Winter C, Akard L, Jansen J, Copelan E, Meagher RC, Herzig RH, Klumpp TR, Kahn DG, Warner NE: Detection and viability of tumor cells in peripheral blood stem cell collections from breast cancer patients using immunocytochemical and clonogenic assay technique. Blood 82:2605, 1993

31. Brugger W, Bross KJ, Glatt M, Weber F, Mertelsmann R, Kanz L: Mobilization of tumor cells and hematopoietic progenitor cells into peripheral lood of patients with solid tumors. Blood 83:636, 1994

32. Gazitt Y, Reading C, Hoffman R, Wickrema A, Vesole DH, Jagannath S, Condino J, Lee B, Barlogie B, Tricot G: Purified $CD34^+$ Lin^- Thy^+ stem cells do not contain clonal myeloma cells. Blood 86:381, 1995

33. Corradini P, Voena C, Astolfi M, Ladetto M, Tarella C, Boccadoro M, Pileri A: High-dose sequential chemoradiotherapy in multiple myeloma: Residual tumor cells are detectable in bone marrow and peripheral blood harvests and after autografting. Blood 85:1596, 1995

34. Gobbi M, Cavo M, Tazzari PL, Dinota A, Tassi C, Bontadini A, Albertazzi L, Miggiano MC, Rizzi S, Rosti G, Bolognesi A, Stirpe F, Tura S: Autologous bone marrow transplantation with immunotoxin-purged marrow for advanced multiple myeloma. Eur J Haematol 43:176, 1989 (suppl 51)

35. Strauss LC, Rowley SD, Larussa VF, Sharkis SJ, Stuart RK, Civin CI: Antigenic analysis of hematopoiesis. V. Characterization of MY 10 antigen expression by normal lymphohematopoietic progenitor cells. Exp Hematol 14:878, 1986

36. Andrews RG, Singer JW, Bernstein ID: Monoclonal antibody 12.8 recognizes a 115-KD molecule present on both unipotent and multipotent hematopoietic colony forming cells and their precursors. Blood 67:842, 1986

37. Andrews RG, Singer JW, Bernstein ID: Precursors of colony-forming cells in humans can be distinguished from colony-forming cells by expression of the CD33 and CD34 antigens and light scatter properties. J Exp Med 169:1721, 1989

38. Berenson RJ, Bensinger WI, Andrews RG, Kalamasz D, Hill R, Bernstein ID: Hematopoietic stem cell transplants, in Gale RP, Golde DW (eds): Recent Advances in Leukemia and Lymphoma, UCLA Symposia on Molecular and Cellular Biology, New Series (vol 61). New York, NY, Liss, 1987, p 527

39. Watt SM, Karhi K, Gatter K, Furley AJW, Katz FE, Healy LE, Altass LJ, Bradley NJ, Sutherland DR, Levinsky R, Greaves MF: Distribution and epitope analysis of the cell membrane glycoprotein

From www.bloodjournal.org by guest on March 3, 2016. For personal use only.

(HPCA-1) associated with human hematopoietic progenitor cells. Leukemia 1:417, 1988

40. Takishita M, Kosaka M, Goto T, Saito S: Cellular origin and extent of clonal involvement in multiple myeloma: Genetic and phenotypic studies. Br J Haematol 87:735, 1994

41. Sahota S, Hamblin T, Oscier DG, Stevenson FK: Assessment of the role of clonogenic B lymphocytes in the pathogenesis of multiple myeloma. Leukemia 8:1285, 1994

42. Vescio RA, Hong CH, Cao J, Kim A, Schiller GJ, Lichtenstein AK, Berenson RJ, Berenson JR: The hematopoietic stem cell antigen, CD34, is not expressed on the malignant cells in multiple myeloma. Blood 84:3283, 1994

43. Schiller GJ, Vescio RA, Freytes C, Spitzer G, Sahebi F, Lee M, Hua C, Cao J, Lee JC, Hong CH, Bearman S, Lill M, Berenson R, Berenson J: Transplantation of $CD34^+$ peripheral blood progenitor cells after high dose chemotherapy for patients with advanced multiple myeloma. Blood 86:390, 1995

44. Berenson RJ, Bensinger WI, Hill RS, Andrews RG, Garcia-Lopez J, Kalamasz DF, Still BJ, Spitzer G, Buckner CD, Bernstein ID, Thomas ED: Engraftment after infusion of $CD34^+$ marrow cells in patients with breast cancer or neuroblastoma. Blood 77:1717, 1991

45. Brugger W, Henschler R, Heimfeld S, Berenson RJ, Mertelsmann R, Kanz L: Positively selected autologous blood $CD34^+$ cells and unseparated peripheral blood progenitor cells mediate identical hematopoietic engraftment after high dose VP 16, ifosfamide, carboplatin, and epirubicin. Blood 84:1421, 1994

46. Gorin NC, Lopez M, Laporte JP, Quittet P, Lesage S, Lemoine F, Berenson RJ, Isnard F, Grande M, Stachowiak J, Labopin M, Fouillard L, Morel P, Jouet JP, Noel-Walter MP, Detourmignies L, Aoudjhane M, Bauters F, Najman A, Douay L: Preparation and succesfull engraftment of purified $CD34^+$ bone marrow progenitor cells in patients with non-Hodgkin' s lymphoma. Blood 85:1647, 1995

47. Klein B, Zhang XG, Lu ZY, Bataille R: Interleukin-6 in human multiple myeloma. Blood 85:863, 1995

48. Witzig TE, Kyle RA, O'Fallon WM, Greipp PR: Detection of peripheral blood plasma cells as a predictor of disease course in patients with smouldering multiple myeloma. Br J Haematol 87:266, 1994

49. Lemoli RM, Gobbi M, Tazzari PL, Tassi C, Dinota A, Visani G, Grassi G, Mazza P, Cavo M, Tura S: Bone marrow purging for multiple myeloma by avidin-biotin immunoabsorption. Transplantation 47:385, 1989

50. Lemoli RM, Tazzari PL, Fortuna A, Bolognesi A, Gulati SC, Stirpe F, Tura S: Positive selection of hematopoietic $CD34^+$ stem cells provides ''indirect purging'' of $CD34^-$ lymphoid cells and the purging efficiency is increased by anti-CD2 and anti-CD30 immunotoxins. Bone Marrow Transplant 13:465, 1994

51. Cottler-Fox M, Cipolone K, Yu M, Berenson RJ, O'Shaughnessy J, Dunbar C: Positive selection of $CD34^+$ hematopoietic cells using an immunoaffinity column results in T-cell depletion equivalent to elutriation. Exp Hematol 23:320, 1995

From www.bloodjournal.org by guest on March 3, 2016. For personal use only.

blood

1996 87: 1625-1634

Concomitant mobilization of plasma cells and hematopoietic progenitors into peripheral blood of multiple myeloma patients: positive selection and transplantation of enriched CD34+ cells to remove circulating tumor cells

RM Lemoli, A Fortuna, MR Motta, S Rizzi, V Giudice, A Nannetti, G Martinelli, M Cavo, M Amabile, S Mangianti, M Fogli, R Conte and S Tura

Updated information and services can be found at:
http://www.bloodjournal.org/content/87/4/1625.full.html

Articles on similar topics can be found in the following Blood collections

Information about reproducing this article in parts or in its entirety may be found online at:
http://www.bloodjournal.org/site/misc/rights.xhtml#repub_requests

Information about ordering reprints may be found online at:
http://www.bloodjournal.org/site/misc/rights.xhtml#reprints

Information about subscriptions and ASH membership may be found online at:
http://www.bloodjournal.org/site/subscriptions/index.xhtml

Blood (print ISSN 0006-4971, online ISSN 1528-0020), is published weekly by the American Society of Hematology, 2021 L St, NW, Suite 900, Washington DC 20036.
Copyright 2011 by The American Society of Hematology; all rights reserved.
