# Supplementary material for: Expression of Concern: Prognostic value of circulating plasma cells in patients with multiple myeloma: A meta-analysis
Source: PLoS One. 2023 Feb 21;18(2):e0282230. doi: 10.1371/journal.pone.0282230 (PMC9942954; doi:10.1371/journal.pone.0282230)
Supplement: S1 File — (ZIP) [file pone.0282230.s001.zip › primary data/excluded research/1998 Distribution of myeloma plasma cells in peripheral blood and bone marrow correlates with CD56 expression..pdf]

British Journal of Haematology, 1999, 104, 138–143

Distribution of myeloma plasma cells in peripheral blood and bone marrow correlates with CD56 expression

ANDREW RAWSTRON, SHARON BARRANS, DAVID BLYTHE, FAITH DAVIES, ANNE ENGLISH, GUY PRATT, ANTHONY CHILD, GARETH MORGAN AND ANDREW JACK *Department of Haematology, The General Infirmary at Leeds*

Received 8 June 1998; accepted for publication 14 October 1998

Summary. There is a wide variation in the degree of marrow and blood involvement between patients with multiple myeloma. Both of these parameters are known to be highly significant prognostic factors, and the differences between patients may be due to variable expression of adhesion molecules. To test this we used three-colour flow cytometry to study three adhesion molecules associated with myeloma, namely CD38, CD56 and CD138. The level of expression of these molecules was compared with the distribution of myeloma plasma cells in bone marrow (n=59) and peripheral blood (n=26) in patients at presentation or relapse. The extent of marrow infiltration on the trephine biopsy correlated inversely with CD56 expression (Mann-Whitney U Test, P=0·022); there was no difference in CD38 or in CD138 expression. CD56 expression also correlated inversely with the number of circulating plasma cells (linear regression, R^2=0·4268, slope=−0·58, P=0·0003). Peripheral blood plasma cell numbers correlated weakly with bone marrow plasmacytosis, and inversely with CD38 expression. The level of CD56 expression by neoplastic plasma cells was assessed in 37 patients over a median of 11 months (range 6–25). There was no significant change in expression (Wilcoxon Signed Rank, P=0·6271). We conclude that plasma cell CD56 expression is constant over the course of the disease; unlike CD138 expression, it is significantly linked to the degree of both bone marrow and peripheral blood involvement.

Keywords: multiple myeloma, plasma cell immunophenotype, distribution, peripheral blood involvement.

Both the extent of marrow infiltration (Bartl *et al*, 1982) and of peripheral blood involvement (Witzig *et al*, 1996) are powerful prognostic factors in multiple myeloma. Little is known about the cellular factors that determine these variables, although there is some evidence to suggest that adhesion molecules play a role. In the bone marrow there are distinctive patterns of distribution that differentiate between normal and myeloma plasma cells. Normal plasma cells, but not myeloma plasma cells, are concentrated around the blood vessels (Bain *et al*, 1996), and this may be related to expression of CD38, which mediates interaction with vascular endothelial cells (Funaro *et al*, 1995). Myeloma plasma cells infiltrate between adipocytes, forming small aggregates, which expand to form more diffuse areas of marrow replacement as the disease progresses (Bartl *et al*, 1989). As quantitation of absolute plasma cell numbers in trephine biopsies is technically difficult, the pattern of infiltration can serve as a measure of the extent of marrow infiltration (Child *et al*, 1998). In the peripheral blood we have previously found that circulating plasma cells express significantly lower levels of the adhesion molecules CD56 and CD138 (Syndecan-1, B-B4) than their paired marrow counterparts (Rawstron *et al*, 1997). The CD138 molecule is the ligand for thrombospondin and other components of the marrow extracellular matrix (Mali *et al*, 1990), and it may therefore mediate adherence to the marrow microenvironment. The CD56 molecule is an isoform of the neural cell adhesion molecule, N-CAM, which mediates homotypic adhesion (Lanier & Hemperley, 1995). It is possible that the level of expression of these molecules governs which cells are able to ‘escape’ the marrow environment. Although such a mechanism has been suggested, there is currently no evidence for a direct relationship between the expression of CD38, CD56 or CD138 and the level of circulating plasma cells.

A number of studies have suggested that lack of CD56 expression identifies groups of patients with a poorer prognosis: patients with CD56 disease have been shown to

Correspondence: Dr A. S. Jack, Haematological Malignancy Diagnostic Service, Algernon Firth Building, The General Infirmary at Leeds, Leeds LS1 3EX.

© 1999 Blackwell Science Ltd

have predominantly aggressive disease (Van Camp *et al*, 1990), and patients with extramedullary disease consistently lack CD56 expression (Pellat-Deceunynck *et al*, 1995). In a study of plasma cell ploidy, higher CD56 expression was demonstrated in the group with better prognosis (Garcia-Sanz *et al*, 1995). Furthermore, levels of soluble CD56 have shown correlation with both β2m levels and with disease progression, suggesting that high levels of sCD56 are associated with more aggressive disease (Kaiser *et al*, 1996). However, in a study of 68 patients at presentation, CD56 expression did not appear to be a prognostic factor (Mathew *et al*, 1995).

The aim of this study was to assess the extent to which CD56, CD138 and CD38 correlate with the pattern of marrow infiltration, and the number of circulating plasma cells.

METHODS

Patients. 134 bone marrow aspirate (BM) samples, 59 trephine biopsies, and 31 peripheral blood (PB) samples were assessed from 96 patients, median age 61 (range 42–85), male : female ratio 1·12 : 1, presenting routinely for diagnosis or follow-up to the Haematological Malignancy Diagnostic Service. The proportion of plasma cells and their distribution was assessed on paired marrow aspirate and trephine biopsies from 59 patients (presentation n=51, relapse n=8). Circulating plasma cell level was assessed using paired marrow aspirate and peripheral blood samples from 31 patients (presentation n=28, relapse n=3). Patients included in this study at relapse were off treatment for >3 months prior to assessment.

Sequential studies of CD56 expression by neoplastic plasma cells were assessed in 37 patients (myeloma n=32, MGUS n=5). During the time period, 20 patients showed decreases in plasma cell levels, eight showed increases, and nine were unaltered. Decreasing numbers of plasma cells were found in patients responding to treatment, either from presentation (C-VAMP + autologous transplant n=10; C-VAMP/ABCM n=8), from plateau (autologous transplant n=1) or relapse (n=1). Patients with no alteration in plasma cell level either had stable disease (MGUS n=5, plateau n=2) or were refractory to chemotherapy from presentation (n=2). Patients showing increases in plasma cell levels were either in relapse (post-autologous transplant n=6, post ABCM n=1) or were refractory to chemotherapy from presentation (n=1).

Sample preparation. Leucocytes were prepared by incubation with a 10-fold excess of ammonium chloride (8·6 g/l in distilled H_2O) for 5 min, and washed twice in FACSFlow (Becton Dickinson)/0·3% BSA (Sigma Diagnostics).

Flow cytometry. 10^6 leucocytes were incubated with 10 μl of each pre-titred antibody per test for 20 min at 4°C, washed twice, and acquired using a Becton Dickinson FACSort with CELLQuest v3.1 software. A minimum of 50 000 total cells were analysed in each test. Plasma cells were identified using a sequential gating strategy, assessing CD38, CD45 and CD138 simultaneously, as well as forward and side light-scatter characteristics. Regions were set around the $CD38^+CD45^{lo}$ population, and on their forward and side scatter characteristics. Further plots were gated to include events satisfying both these regions. Regions were checked to ensure that all gated cells expressed CD138, and that all $CD138^{++}$ cells were within this gate, and adjusted if necessary.

Immunohistochemistry. Distribution was assessed on methyl methacrylate 3 μm sections stained as described previously (Blythe *et al*, 1997) with Giemsa, kappa, lambda or Vs38C. Briefly, for immunoperoxidase staining of slides, sections underwent antigen retrieval by microwaving in 400 ml of citrate buffer pH 6·0 (8 min irradiation, 5 min standing, 3 min irradiation then 20 min standing). Sections were then incubated in primary antibody for >1 h, and visualized using a streptavidin–biotin technique (DAKO-Duet K492), with 3,3′-diaminobenzidine (Sigma), and counterstained with Harris' haematoxylin. The degree of marrow infiltration was classified according to the pattern of plasma cell distribution. Samples in which plasma cells were present as single cells or small clusters between adipocytes or haemopoietic cells were classified as 'interstitial', whereas those with large areas of marrow replaced by confluent sheets of plasma cells were classified as 'diffuse'. Cases in which the plasma cells showed mainly an interstitial distribution, but with occasional focal areas of diffuse marrow replacement, were classified as 'interstitial and diffuse'. Cases where the majority of normal haemopoietic tissue was replaced by myeloma were classified as 'replacement' (Fig 1).

Antibodies. Antibodies used for flow cytometry were CD138/Syndecan-1 (B-B4, Serotec) PE, CD56 (MY 31, Becton Dickinson) PE, CD38 (OKT10) Cy5, CD45 (4B2) FITC, CD19 (FMC-63) PE and CD3 (OKT3) PE. Antibody conjugates were prepared in-house, from hybridoma supernatant and titred against known antigen positive cell lines unless from a commercial source. Antibodies used for immunohistochemistry were kappa (1 : 400, Dako), lambda (1 : 300, Dako) or Vs38C (1 : 50, Dako).

RESULTS

Flow cytometric enumeration of myeloma plasma cells correlates with degree of bone marrow infiltration

The degree of marrow infiltration was identified by the distribution of plasma cells on a trephine biopsy. Biopsies from patients at presentation or relapse were classified into one of four categories: interstitial (I, n=10), interstitial and diffuse (ID, n=23), or diffuse (D, n=16) infiltrate, or complete marrow replacement (R, n=10) (Fig 1). There was good correlation between the percentage of plasma cells in the aspirate with the degree of marrow infiltration (Fig 1). The percentage was significantly increased in patients with marrow replacement in comparison to those with only an interstitial infiltrate (Wilcoxon-Mann-Whitney U test, I n=10, R n=10, P=0·0013). In one case with marrow replacement but only 6·5% plasma cells, the aspirate sample was partially clotted, and this sample was excluded from further analysis.

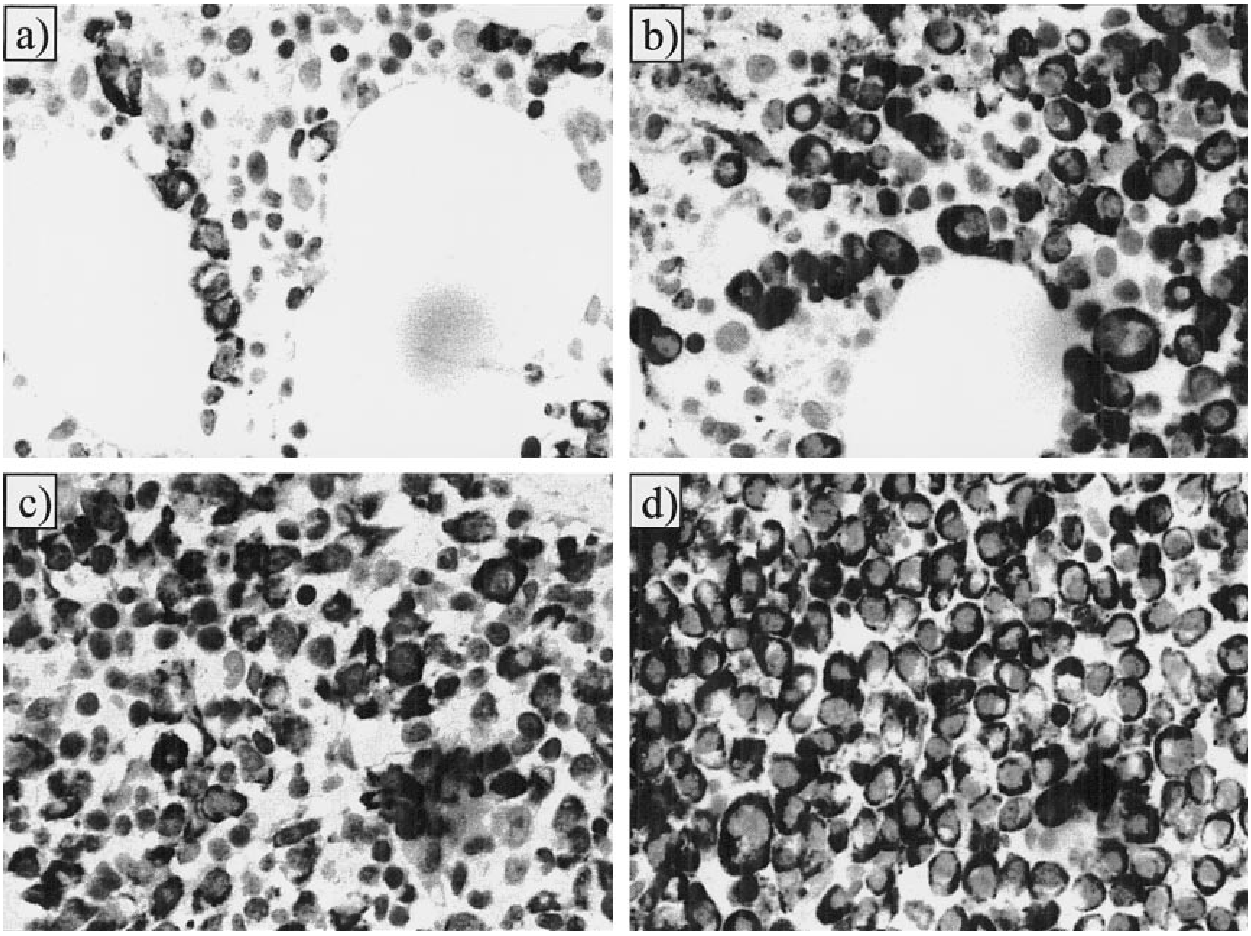

Fig 1. Representative methyl methacrylate 3 μm sections stained with Vs38C, demonstrating the four plasma cell distribution patterns: (a) interstitial, (b) interstitial and diffuse, (c) diffuse, and (d) marrow replacement.

CD56 but not CD138 or CD38 expression is significantly decreased in patients with marrow replacement in comparison to those with interstitial infiltrates

As CD38, CD56 and CD138 have a unimodal distribution on patient cells, the level of expression was measured indirectly using the mean fluorescence intensity of bound antibody. Fig 2 shows the difference in expression of these antigens in comparison to the degree of marrow infiltration. There was no difference in expression of CD38 nor of CD138 by plasma cells in the different groups. However, the expression of CD56 shows an inverse correlation with the degree of marrow infiltration, and median expression is over one log lower in patients with marrow replacement in comparison to those with an interstitial infiltrate (Wilcoxon-Mann-Whitney U test, I n=10, R n=9, P=0·022, Fig 1).

CD56 but not CD138 expression correlates inversely with the absolute numbers of circulating plasma cells

The level of expression of CD38, CD56 and CD138 was assessed in both circulating plasma cells and marrow plasma cells from paired samples of blood and marrow, and compared with the absolute numbers of circulating plasma cells in 31 patients at presentation (n=28) or relapse (n=3). Examination of the data suggested an inverse exponential relationship between the level of CD56 expression and the degree of peripheral blood involvement. The data was therefore log transformed prior to linear regression analysis. The level of CD56 expression, and to a lesser extent CD38 expression, showed an inverse correlation with the absolute numbers of circulating plasma cells (Fig 3). This applied to both blood and marrow plasma cell adhesion molecule expression, although it was slightly less significant for marrow plasma cell expression (PB: CD38 R^2=0·1641, P=0·0219; CD56 R^2=0·4269, P=0·0003; CD138 R^2= 0·0527, P=0·2594; BM: CD38 R^2=0·1698, P=0·0364; CD56 R^2=0·3411, P=0·0017; CD138 R^2=0·0769, P= 0·1701). There was also a weak correlation between the degree of bone marrow infiltration and the numbers of circulating plasma cells (R^2=0·2001, P=0·0219).

CD56 expression does not vary over the course of the disease

In order to assess how the level of CD56 expression by neoplastic plasma cells varies with time, samples from 37 patients with plasma cells that had an atypical phenotype ($CD19^-$ or $CD19^+56^+$) were analysed at two time points which were separated by a median of 11 months (range 6–25).

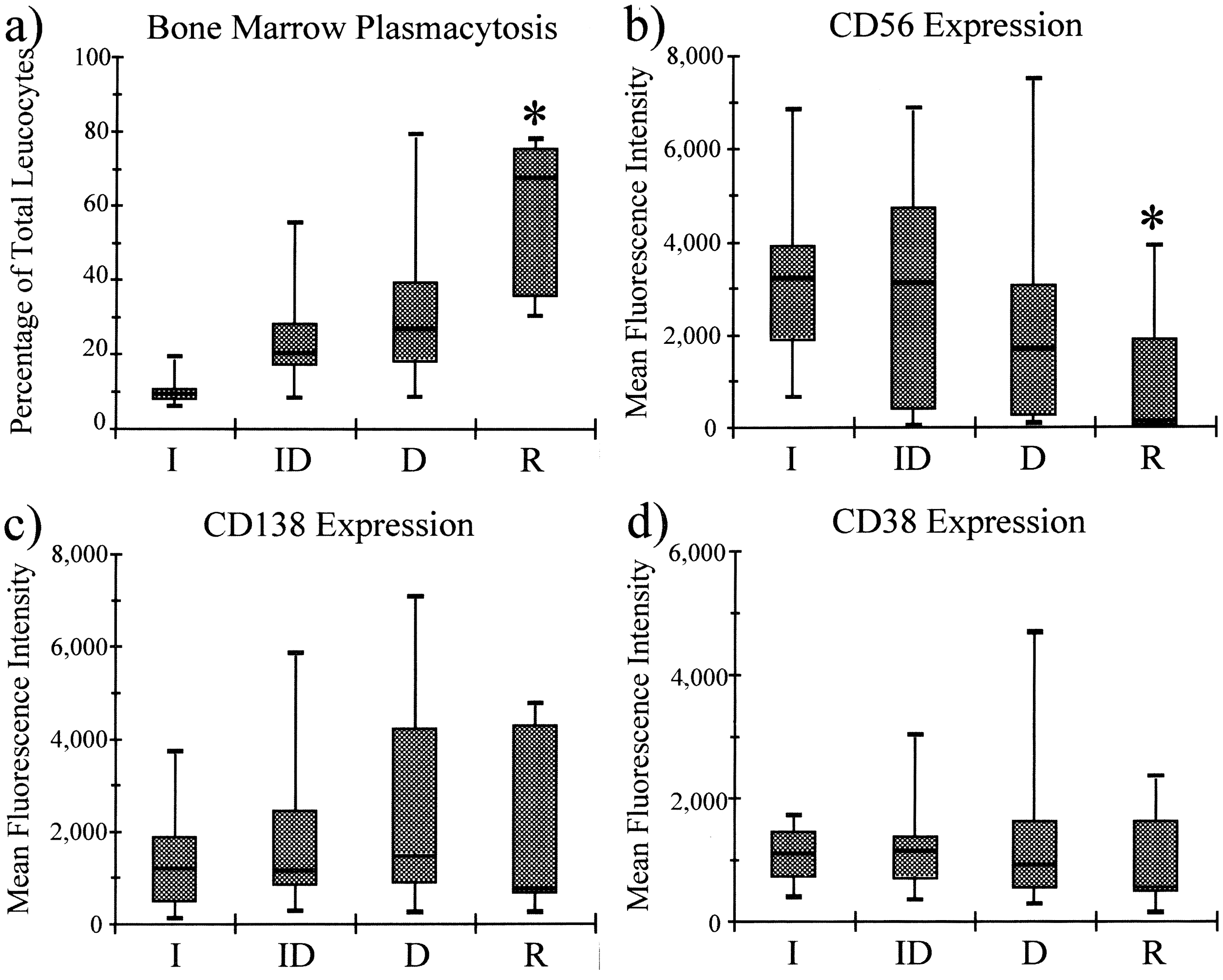

Fig 2. The degree of marrow infiltration correlates with the percentage of plasma cells in the marrow aspirate (a), correlates inversely with the level of plasma cell CD56 expression (b), but shows no correlation with CD138 (c) and CD38 (d) expression. * Indicates significant difference to the interstitial (I) group. ID, interstitial and diffuse group; D, diffuse group; R, complete marrow replacement group.

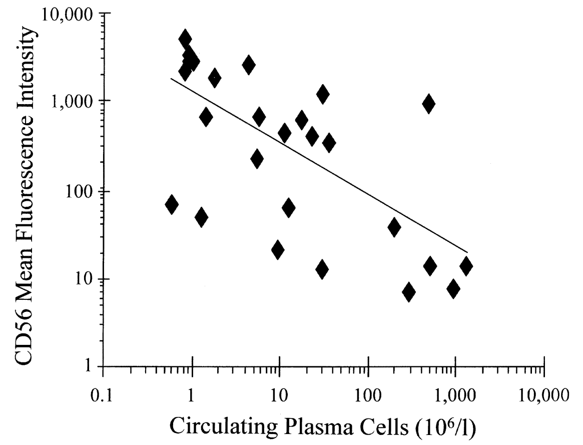

Fig 3. The level of CD56 expression (measured as mean fluorescent intensity) by marrow plasma cells correlates inversely with the absolute numbers of circulating plasma cells.

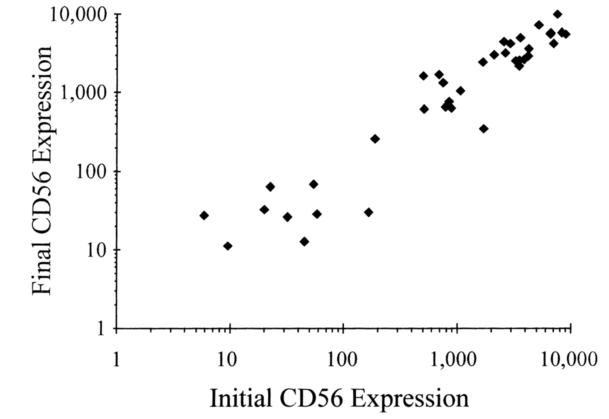

Fig 4. CD56 expression is a constant over the course of the disease. Patients were assessed at serial time points, and the figure shows the initial plasma cell CD56 expression plotted against the final CD56 expression. CD56 expression was measured as mean fluorescence intensity.

Over this period of time, 20 patients showed decreases in the percentage of bone marrow plasma cells, mostly corresponding to response to treatment. Nine patients, mostly with stable disease off treatment, showed no alteration in plasma cell levels. The remaining eight patients showed increases in plasma cell levels consistent with disease progression (see Materials and Methods).

There was no significant alteration of CD56 expression by bone marrow myeloma plasma cells over the course of the disease (P=0·6271, n=3, Wilcoxon matched-pairs signed ranks). The median alteration in expression was a 0·02 log decrease in intensity (range −0·74 to 0·67), with no patients demonstrating >1 log alteration (Fig 4).

DISCUSSION

The results of this study demonstrated that there was a highly significant correlation between the level of CD56 expression by myeloma plasma cells and the degree of peripheral blood and bone marrow involvement. There was a weak but significant correlation between absolute numbers of circulating plasma cells and the level of both CD38 expression and degree of bone marrow replacement. Syndecan-1 (B-B4, CD138) expression did not correlate with the degree of bone marrow or of peripheral blood involvement. There was also a positive correlation between the degree of bone marrow replacement and the absolute numbers of circulating plasma cells, which is in keeping with previous studies demonstrating that peripheral blood involvement correlates with disease stage (Rawstron *et al*, 1997). However, this study demonstrates that, within individual patients, peripheral blood involvement is more closely related to the level of CD56 expression than to the tumour load.

The CD56 molecule is an isoform of the neural cell adhesion molecule (NCAM) which mediates homotypic adhesion (Lanier & Hemperley, 1995). Although its role in neural development is well described (Fields & Itoh, 1996), its activity in myeloma is less clear. However, a number of marrow elements other than neoplastic plasma cells express CD56. These include stromal cell lines that support lymphopoiesis (Thomas *et al*, 1998), osteoblasts (Barille *et al*, 1995) and osteoclasts, which express significantly higher levels of CD56 in myeloma than their normal counterparts (Gregoretti *et al*, 1994). Decreased adherence to these cells may allow disease spread both within the marrow, and between the marrow and other compartments, consistent with the results of this study.

Low CD56 expression predicts high levels of both marrow and blood tumour involvement, both poor prognostic factors (Davies *et al*, 1997). Unlike other prognostic factors that are surrogate markers of disease bulk, such as β_2 microglobulin, the results of this study demonstrate that CD56 is a stable feature of the neoplastic plasma cells. It is therefore of great interest to study how CD56 affects the rate of change of bone marrow disease in sequential samples. The lack of correlation with overall prognosis may be due to the fact that so few patients have been studied, and that CD56 expression has been assessed as positive or negative, rather than as a continuous variable. Further studies are therefore required to determine whether CD56 is an independent prognostic factor.

CD38 expression correlated inversely with the degree of blood involvement. As this molecule mediates adhesion to vascular endothelium, high expression may restrict egress from the marrow. Furthermore, this may account for the differences between normal and myeloma plasma cell distribution in the marrow, as the latter cells have a log lower CD38 fluorescence intensity than their normal counterparts (Rawstron *et al*, 1997). Although circulating plasma cells expressed significantly lower levels of Syndecan-1 than paired marrow counterparts, there was no correlation between Syndecan-1 expression and blood or marrow involvement. This may be because the level of Syndecan-1 showed little variation between patients, with the differences between blood and marrow being due to an increased probability of cells with low Syndecan-1 expression leaving the marrow microenvironment.

In summary, our results demonstrated a clear correlation between the levels of CD56 expression and the degree of bone marrow and peripheral blood involvement. This parameter was more predictive of the level of circulating plasma cells than the degree of marrow replacement. CD138 expression was not linked to plasma cell distribution, although CD38 expression may be important in the differential distribution of normal and myeloma plasma cells within the marrow. The fact that CD56 is a stable feature of the disease, and is closely related to two powerful prognostic factors, suggests that the level of expression should have an effect on patient outcome. Further studies are required to determine whether the level of CD56 expression determines the duration of plateau and progression-free survival.

ACKNOWLEDGMENTS

This work was supported by grants from the Leukaemia Research Fund and the Yorkshire Cancer Research.

REFERENCES

Bain, B.J., Clark, D.M. & Lampert, I.A. (1996) In: *Bone Marrow Pathology*, p. 25. Blackwell Science, Oxford.

Barille, S., Collette, M., Bataille, R. & Amiot, M. (1995) Myeloma cells upregulate interleukin-6 secretion in osteoblastic cells through cell-to-cell contact but downregulate osteocalcin. *Blood*, 86, 3151–3159.

Bartl, R., Frisch, B., Burkhardt, R., Fateh-Moghadam, A., Mahl, G., Gierster, P., Sund, M. & Kettner, G. (1982) Bone marrow histology in myeloma: its importance in diagnosis, prognosis, classification, and staging. *British Journal of Haematology*, 51, 361–375.

Bartl, R., Frisch, B., Diem, H., Mundel, M. & Fateh-Moghadam, A. (1989) Bone marrow histology and serum $\beta 2$ microglobulin in multiple myeloma: a new prognostic strategy. *European Journal of Haematology*, 51, 88–98.

Blythe, D., Hand, N.M., Jackson, P., Barrans, S.L., Bradbury, R.D. & Jack, A.S. (1997) Use of methylmethacrylate resin for embedding bone marrow trephine biopsy specimens. *Journal of Clinical Pathology*, 50, 45–50.

Child, J.A., Jack, A.S. & Morgan, G.J. (1998) In: *The Lymphoproliferative Disorders*, p. 260. Chapman & Hall, London.

Davies, F.E., Jack, A.S. & Morgan, G.J. (1997) The use of biological variables to predict outcome in multiple myeloma. *British Journal of Haematology*, 99, 719–725.

Fields, R.D. & Itoh, K. (1996) Neural cell adhesion molecules in activity-dependent development and synaptic plasticity. *Trends in Neurosciences*, 19, 473–480.

Funaro, A., Roggero, S., Morenstein, A., Calosso, L., Dianzani, U., De Monte, L.B., Zocchi, E., Franco, L., Guida, L., Ausiello, C.M., Drach, J., Mehta, K., Bargeuesi, A. & Malavasi, F. (1995) CD38: a transmembrane glycoprotein with plieotropic ectoenzyme function. In: *Leucocyte Typing V* (ed. by S. F. Schlossman *et al*), pp. 380–383. Oxford University Press.

Garcia-Sanz, R., Orfao, A., Gonzalez, M., Moro, M.J., Hernandez, J.M., Ortega, F., Borrego, D., Carnero, M., Casanova, F., Jimenez, R., Portero, A. & San Miguel, J.F. (1995) Prognostic implications of DNA aneuploidy in 156 untreated multiple myeloma patients. Castelano-Leones (Spain) Cooperative Group for the Study of Monoclonal Gammopathies. *British Journal of Haematology*, 90, 106–112.

Gregoretti, M.G., Gottardi, D., Ghia, P., Bergui, L., Merico, F., Marchisio, P.C. & Caligaris-Cappio, F. (1994) Characterization of bone marrow stromal cells from multiple myeloma. *Leukemia Research*, 18, 675–682.

Kaiser, U., Oldenburg, M., Jaques, G., Auerbach, B. & Havemann, K. (1996) Soluble CD56 (NCAM): a new differential-diagnostic and prognostic marker in multiple myeloma. *Annals of Hematology*, 73, 121–126.

Lanier, L.L. & Hemperley, J.J. (1995) CD56 and CD57 cluster workshop report. *Leucocyte Typing V* (ed. by S. F. Schlossman *et al*), pp. 1398–1400. Oxford University Press.

Mali, M., Jaakkola, P., Arvilommi, A.M. & Jalkanen, M. (1990) Sequence of human syndecan indicates a novel gene family of integral membrane proteoglycans. *Journal of Biological Chemistry*, 265, 6884–6889.

Mathew, P., Ahmann, G.J., Witzig, T.E., Roche, P.C., Kyle, R.A. & Greipp, P.R. (1995) Clinicopathological correlates of CD56 expression in multiple myeloma: a unique entity? *British Journal of Haematology*, 90, 459–461.

Pellat-Deceunynck, C., Barille, S., Puthier, D., Rapp, M.J., Harousseau, J.L., Bataille, R. & Amiot, M. (1995) Adhesion molecules on human myeloma cells: significant changes in expression related to malignancy, tumor spreading, and immortalization. *Cancer Research*, 55, 3647–3653.

Rawstron, A.C., Owen, R.G., Davies, F.E., Johnson, R.J., Jones, R.A., Richards, S.J., Evans, P.A.S., Child, J.A., Smith, G.M., Jack, A.S. & Morgan, G.J. (1997) Circulating plasma cells in multiple myeloma: characterization and correlation with disease stage. *British Journal of Haematology*, 97, 46–55.

Thomas, P.S., Pietrangeli, C.E., Hayashi, S., Schachner, M., Goridis, C., Low, M. & Kincade, P.W. (1998) Demonstration of neural cell adhesion molecules on stromal cells that support lymphopoiesis. *Leukemia*, 2, 171–175.

Van Camp, B., Durie, B.G., Spier, C., De Waele, M., Van Riet, I., Vela, E., Frutiger, Y., Richter, L. & Grogan, T.M. (1990) Plasma cells in multiple myeloma express a natural killer cell-associated antigen: CD56 (NKH-1; Leu-19). *Blood*, 76, 377–382.

Witzig, T.E., Gertz, M.A., Lust, J.A., Kyle, R.A., O'Fallon, W.M. & Greipp, P.R. (1996) Peripheral blood monoclonal plasma cells as a predictor of survival in patients with multiple myeloma. *Blood*, 88, 1780–1787.
