# Supplementary material for: Expression of Concern: Prognostic value of circulating plasma cells in patients with multiple myeloma: A meta-analysis
Source: PLoS One. 2023 Feb 21;18(2):e0282230. doi: 10.1371/journal.pone.0282230 (PMC9942954; doi:10.1371/journal.pone.0282230)
Supplement: S1 File — (ZIP) [file pone.0282230.s001.zip › primary data/excluded research/2003 Bone marrow angiogenesis and circulating plasma cells in multiple myeloma.pdf]

British Journal of Haematology, 2003, **122**, 272–274

SHORT REPORT

Bone marrow angiogenesis and circulating plasma cells in multiple myeloma

Shaji Kumar, Thomas E. Witzig, Philip R. Greipp and S. Vincent Rajkumar *Division of Hematology and Internal Medicine, Mayo Clinic, Rochester, MN, USA*

Received 20 January 2003; accepted for publication 25 March 2003

Summary. Bone marrow (BM) angiogenesis is increased in multiple myeloma (MM) and has prognostic significance. The presence of circulating plasma cells (PCs) in MM is associated with a poorer prognosis. We examined BM biopsies obtained at diagnosis of MM for angiogenesis, and correlated the microvessel density (MVD) with the presence of circulating PCs. There was a positive correlation between the absolute number of circulating PCs and the mean MVD. This relationship was independent of the disease activity and of the PC burden in the marrow. The increased angiogenesis may promote plasma cell proliferation and enable PC migration into the circulation.

Keywords: multiple myeloma, cell proliferation, angiogenesis, plasma cell, circulating tumour cell.

Abnormal, increased new blood vessel formation or angiogenesis in the bone marrow (BM) is seen in many haematological malignancies, similar to that described in solid tumours (Folkman, 1995). In multiple myeloma (MM), the degree of BM angiogenesis is a powerful prognostic factor for survival, which gradually increases with increasing disease activity, and persists after treatment (Rajkumar *et al*, 2000, 2002; Kumar *et al*, 2002). Clonal plasma cells (PCs) and B-lymphocytes can be detected in the peripheral blood of patients with PC proliferative disorders by immunofluorescence microscopy (IM) and flow cytometry (FC), and are a measure of disease activity (Witzig *et al*, 1988). We have previously demonstrated that the number of circulating PCs can predict the disease course in smouldering MM, and is an independent prognostic factor for survival (Witzig *et al*, 1996). Here, we examined the relationship between the degree of bone marrow angiogenesis and the presence of circulating PCs in MM patients.

MATERIALS AND METHODS

MM patients ($n = 110$), for whom a complete clinical follow-up and archived BM biopsy blocks were available and who also had a peripheral blood PC (PBPC) estimation within a month of the biopsy, were studied.

PBPC detection and quantification was performed using a sensitive, slide-based immunofluorescence technique, as previously described (Witzig *et al*, 1996). Plasma cells were identified morphologically and expressed as a percentage of the cytoplasmic immunoglobulin containing cells (cIg^+) cells. A high PBPC count was defined as $> 0{\cdot}5 \times 10^6$ plasma cells/l. Paraffin-embedded BM biopsy blocks were used to prepare slides for microvessel density (MVD) determination. Immunohistochemical staining for CD34 was performed by a labelled streptavidin–biotin peroxidase method, and the degree of angiogenesis was estimated in terms of MVD, as previously described (Rajkumar *et al*, 2000, 2002). Briefly, slides were first scanned under low power (100×) in order to determine three 'hot-spots' or areas with the maximum number of microvessels, which were then evaluated at 400× magnification. The number of microvessels in each field was determined and their average was expressed as MVD per field. This technique has been validated, and has a low interobserver variability and correlates well with other methods, such as computerized image analysis. Patients were divided into two groups based on the average MVD/field: low (MVD < 20) and high (MVD ≥ 20).

Statistical analyses. The non-parametric Mann–Whitney *U*-test or Kruskal–Wallis test was used to compare the MVD and the circulating plasma cells among different groups. Chi-squared test was used to compare categorical variables. Logistic regression was used to determine independence of the relationship between MVD and circulating PC. Approval for the study was obtained from the Institutional Review Board.

RESULTS AND DISCUSSION

The study group consisted of 110 patients with smouldering MM (44 patients), newly diagnosed MM (52 patients) or

Correspondence: Dr S. V. Rajkumar, Mayo Clinic, 200 First Street SW, Rochester, MN 55905, USA. E-mail: rajks@mayo.edu

© 2003 Blackwell Publishing Ltd

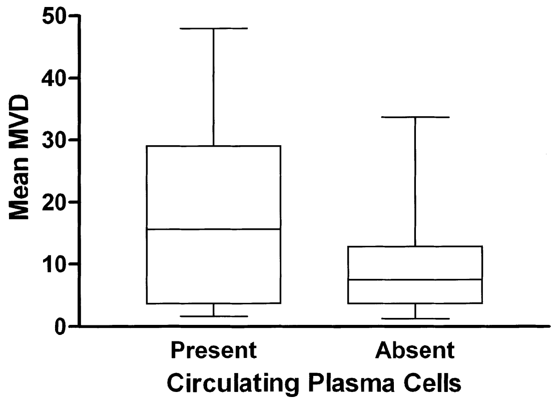

Fig 1. Box plot demonstrating the correlation between bone marrow angiogenesis and circulating plasma cells.

relapsed MM (14 patients). The median MVDs in these three groups were 5, 13 and 23 respectively ($P < 0{\cdot}0001$). Twenty-eight patients (25%) had high-grade angiogenesis (median MVD, 33) and the remaining 82 patients (75%) had low-grade angiogenesis (median MVD, 6). The median circulating plasma cells in the high-grade group was 10×10^6/l (mean 192; range 0–2434) compared with 0×10^6/l (mean 24; range 0–822) for the low-grade group ($P < 0{\cdot}0001$). The median MVD in patients with circulating PC was 16 (mean 18; range 2–48) compared with 8 (mean 9; range 1–34) in those with no circulating PC ($P = 0{\cdot}0002$) (Fig 1). The number of circulating plasma cells correlated with the average MVD (rho = 0·353, $P < 0{\cdot}0001$). In a multivariate model, including the disease stage, bone marrow plasma cell percentage and MVD, only MVD was independently predictive of the presence of circulating plasma cells ($P = 0{\cdot}02$).

The exact role of increased bone marrow angiogenesis in the biology of MM continues to be unraveled (Vacca *et al*, 1994; Rajkumar *et al*, 2002). Although concerns have been raised about marrow angiogenesis being an epiphenomenon, several lines of evidence support an important role for this finding, similar to that in solid tumours. Regression of myeloma in mouse models has been seen with antiangiogenic agents (Fujii *et al*, 2002). The prognostic value of angiogenesis in solitary bone plasmacytomas, the solid tumour equivalent of myeloma, and the increasing degree of angiogenesis seen from monoclonal gammopathy of unknown significance to active myeloma again underscores its importance in the biology of this disease (Rajkumar *et al*, 2002).

The presence of circulating clonal B cells and plasma cells in the peripheral circulation has been known for a long time (Witzig *et al*, 1988). The mechanisms for the movement of these cells into the peripheral circulation are not clear. Loss of syndecan, very late antigen-4 and other adhesion molecules have been incriminated in this migration (Pellat-Deceunynck *et al*, 1995). Circulating PCs are proliferative and may be important in the dissemination of the disease.

The malignant plasma cells interact with the stromal and vascular endothelial cells in the bone marrow microenvironment through various cytokines and adhesion molecules. Adhesion of the PC to the stromal cell results in secretion of vascular endothelial growth factor (VEGF), which plays a role in the initiation and maintenance of abnormal angiogenesis. VEGF is capable of stimulating the proliferation and migration of myeloma cells (Podar *et al*, 2001). VEGF stimulates both endothelial cells and stromal cells to secrete interleukin 6 (IL-6), which is a key cytokine regulator of myeloma cell growth (Kawano *et al*, 1988) and an essential growth factor for myeloma cells. IL-6 in turn has been shown to be capable of stimulating VEGF secretion by myeloma cells, resulting in a feedback loop (Dankbar *et al*, 2000). These close biological interactions between the endothelial cell and the myeloma cells can partly explain the correlation observed in our study. It is also possible that the relatively porous new blood vessels in the marrow allow the plasma cells to enter the peripheral circulation. This relationship between bone marrow angiogenesis and the presence and burden of circulating plasma cells has not been described before and provides new insight into the biology of the disease.

ACKNOWLEDGMENTS

Supported in part by the Goldman Philanthropic Partnerships, IL, USA, and Grants CA 93842, CA 85818, CA62242, CA 10080, National Cancer Institute, Bethesda, MD, USA. Dr Rajkumar is a Leukaemia and Lymphoma Society of America Translational Research Awardee and is also supported by the Multiple Myeloma Research Foundation.

REFERENCES

Dankbar, B., Padro, T., Leo, R., Feldmann, B., Kropff, M., Mesters, R.M., Serve, H., Berdel, W.E. & Kienast, J. (2000) Vascular endothelial growth factor and interleukin-6 in paracrine tumor–stromal cell interactions in multiple myeloma. *Blood*, **95**, 2630–2636.

Folkman, J. (1995) Seminars in Medicine of the Beth Israel Hospital, Boston. Clinical applications of research on angiogenesis. *New England Journal of Medicine*, **333**, 1757–1763.

Fujii, H., Yaccoby, S. & Epstein, J. (2002) Control of myeloma with the anti-angiogenic agent endostatin. *Blood*, **96**, 360a.

Kawano, M., Hirano, T., Matsuda, T., Taga, T., Horii, Y., Iwato, K., Asaoku, H., Tang, B., Tanabe, O., Tanaka, H., Kuramoto, A. & Kishimoto, T. (1988) Autocrine generation and requirement of BSF-2/IL-6 for human multiple myelomas. *Nature*, **332**, 83–85.

Kumar, S., Fonseca, R., Dispenzieri, A., Lacy, M.Q., Lust, J.A., Witzig, T.E., Gertz, M.A., Kyle, R.A., Greipp, P.R. & Rajkumar, S.V. (2002) Bone marrow angiogenesis in multiple myeloma: effect of therapy. *British Journal of Haematology*, **119**, 665–671.

Pellat-Deceunynck, C., Barille, S., Puthier, D., Rapp, M.J., Harousseau, J.L., Bataille, R. & Amiot, M. (1995) Adhesion molecules on human myeloma cells: significant changes in expression related to malignancy, tumor spreading, and immortalization. *Cancer Research*, **55**, 3647–3653.

Podar, K., Tai, Y.T., Davies, F.E., Lentzsch, S., Sattler, M., Hideshima, T., Lin, B.K., Gupta, D., Shima, Y., Chauhan, D., Mitsiades, C., Raje, N., Richardson, P. & Anderson, K.C. (2001) Vascular endothelial growth factor triggers signaling cascades

mediating multiple myeloma cell growth and migration. *Blood*, **98**, 428–435.

Rajkumar, S.V., Leong, T., Roche, P.C., Fonseca, R., Dispenzieri, A., Lacy, M.Q., Lust, J.A., Witzig, T.E., Kyle, R.A., Gertz, M.A. & Greipp, P.R. (2000) Prognostic value of bone marrow angiogenesis in multiple myeloma. *Clinical Cancer Research*, **6**, 3111–3116.

Rajkumar, S.V., Mesa, R.A., Fonseca, R., Schroeder, G., Plevak, M.F., Dispenzieri, A., Lacy, M.Q., Lust, J.A., Witzig, T.E., Gertz, M.A., Kyle, R.A., Russell, S.J. & Greipp, P.R. (2002) Bone marrow angiogenesis in 400 patients with monoclonal gammopathy of undetermined significance, multiple myeloma, and primary amyloidosis. *Clinical Cancer Research*, **8**, 2210–2216.

Vacca, A., Ribatti, D., Roncali, L., Ranieri, G., Serio, G., Silvestris, F. & Dammacco, F. (1994) Bone marrow angiogenesis and progression in multiple myeloma. *British Journal of Haematology*, **87**, 503–508.

Witzig, T.E., Gonchoroff, N.J., Katzmann, J.A., Therneau, T.M., Kyle, R.A. & Greipp, P.R. (1988) Peripheral blood B cell labeling indices are a measure of disease activity in patients with monoclonal gammopathies. *Journal of Clinical Oncology*, **6**, 1041–1046.

Witzig, T.E., Gertz, M.A., Lust, J.A., Kyle, R.A., O'Fallon, W.M. & Greipp, P.R. (1996) Peripheral blood monoclonal plasma cells as a predictor of survival in patients with multiple myeloma. *Blood*, **88**, 1780–1787.
