# Supplementary material for: Expression of Concern: Prognostic value of circulating plasma cells in patients with multiple myeloma: A meta-analysis
Source: PLoS One. 2023 Feb 21;18(2):e0282230. doi: 10.1371/journal.pone.0282230 (PMC9942954; doi:10.1371/journal.pone.0282230)
Supplement: S1 File — (ZIP) [file pone.0282230.s001.zip › primary data/excluded research/2007 Detection and follow-up of fibroblast growth factor receptor 3 expression on bone marrow and....pdf]

Detection and follow-up of fibroblast growth factor receptor 3 expression on bone marrow and circulating plasma cells by flow cytometry in patients with t(4;14) multiple myeloma

M. O. Chandesris,[1] J. Soulier,[2] S. Labaume,[1] A. Crinquette,[2] L. Repellini,[1] K. Chemin,[1] M. Malphettes,[1,3] C. Fieschi,[1] B. Asli,[3] Y. Uzunhan,[3] J. P. Fermand,[1,3] J. C. Bories[1] and B. Arnulf[1,3]

[1]*EA 3963, Paris VII University, Saint-Louis Hospital,* [2]*Central Haematology Laboratory, Assistance Publique-Hôpitaux de Paris, Saint-Louis Hospital, AP-HP, and* [3]*Immuno-Haematology Department, Assistance Publique-Hôpitaux de Paris, Saint-Louis Hospital, AP-HP, Paris, France*

Summary

The t(4;14)(p16;q32) translocation, found in 15% of multiple myeloma (MM) cases, indicates a poor prognosis. Plasma cells (PC) with t(4;14) ectopically express the fibroblast growth factor receptor 3 (FGFR3) tyrosine kinase receptor, which has potential transforming activity and may represent a therapeutic target. To detect FGFR3 protein expression, bone marrow (BM) aspirate from 200 consecutive newly diagnosed ($n = 116$) or relapsing ($n = 74$) MM patients was studied by flow cytometry (FC) using anti-CD138 and anti-FGFR3 antibodies. FC data was compared to real time quantitative-polymerase chain reaction (RQ-PCR) of the *IGH-MMSET* and *FGFR3* transcripts. An *IGH-MMSET* transcript was found in 24/200 patients (12%). In 20 of these, FC detected $CD138^+/FGFR3^+$ cells. No expression of FGFR3 was detected in the 4 $FGFR3^-$ cases by RQ-PCR. FGFR3 was never expressed on PC without t(4;14). Circulating PC (CPC) were detected in patients with (11/11) and patients without (13/41) t(4;14). In 2/8 t(4;14) cases studied longitudinally, coexisting $FGFR3^+$ and $FGFR3^-$ CPC were observed. Fluorescent *in situ* hybridisation (FISH) analysis of the $FGFR3^-$ subclones showed deletion of the der(14) in one patient. In conclusion, as a supplemental method to RQ-PCR or FISH, FC analysis of FGFR3 expression is a reliable and routinely available method for the detection and management of new therapeutic approaches of t(4;14) MM.

Keywords: multiple myeloma, t(4;14) translocation, fibroblast growth factor receptor 3 detection, flow cytometry, circulating plasma cells.

Received 29 September 2006; accepted for publication 30 November 2006

Correspondence: Dr Bertrand Arnulf, Centre Hayem 2eme etage, Saint-Louis Hospital, EA 3963, Paris VII University, 1 avenue Claude Vellefaux, 75475 Paris cedex 10, France.
E-mail: bertrand.arnulf@sls.ap-hop-paris.fr

Advances in the tumour plasma cell biology of multiple myeloma (MM) have pointed to chromosomal translocation involving the immunoglobulin heavy chain locus located at 14q32. These rearrangements occur in about 50–70% of MM cases and involve multiple oncogenic partners, conferring different prognoses (Kuehl & Bergsagel, 2005). The t(14;14)(p16;q32) is found in 15% of MM cases and is associated with resistance to conventional and intensive chemotherapy and a short time to progression when a response occurs, leading to a poor prognosis (Winkler *et al*, 2003; Chang *et al*, 2004; Jaksic *et al*, 2005). This reciprocal translocation leads to the deregulation of two potential oncogenes, the fibroblast growth factor receptor 3 (*FGFR3*), which is ectopically expressed on der(14), and the multiple myeloma SET domain gene (*MMSET*), which is over expressed from a fusion gene on der(4) (Kuehl & Bergsagel, 2005).

Fibroblast growth factor receptor 3 is a tyrosine kinase receptor that belongs to the high affinity FGFR family (FGFR1 to 5). FGF binding induces dimerisation of FGFR3, tyrosine auto-phosphorylation and signal transduction through mitogen-activated protein kinase (MAPK), phospholipase C (PLC) gamma and phosphatidylinositol 3 (PI3) kinase to induce cell proliferation and differentiation (L'Hôte & Knowles, 2005). Ectopic expression of FGFR3 in plasma cells seems to have a transforming activity and the abolition of FGFR3 expression or kinase activity induced apoptosis in t(4;14) plasma cells from patients and in t(4;14) MM cell lines (Plowright *et al*, 2000; Chesi *et al*, 2001; Li *et al*, 2001; Trudel *et al*, 2004a). Thus,

© 2007 The Authors

FGFR3 may represent a potential therapeutic target in this subset of MM (Trudel *et al*, 2004b; Chen *et al*, 2005).

Lack of FGFR3 expression was observed in the tumour plasma cells of 25–30% of patients with t(4;14) MM, mostly as a result of a deletion of the der(14) chromosome (Keats *et al*, 2003; Santra *et al*, 2003). Therefore, t(4;14) is usually searched for by fluorescent *in situ* hybridisation (FISH), or alternatively, by detection of the *IGH/MMSET* fusion transcript using polymerase chain reaction (PCR). However, detection and follow up of FGFR3 expression at the protein level is needed, particularly with the aim of developing tyrosine kinase inhibitors as therapeutic agents in t(4;14) MM. Accordingly, we conducted a study to quantitatively detect FGFR3 by flow cytometry (FC) on the plasma cell membrane in the bone marrow (BM) and peripheral blood (PB) of a series of newly diagnosed or relapsing patients with MM.

Patients and methods

Patients

Bone marrow samples from two hundred consecutive patients (112 men and 88 women, median age 59 years) with newly diagnosed ($n = 116$) or relapsing ($n = 74$) MM were studied. In 52 patients, PB and BM samples were studied in parallel. In eight patients in whom a t(4;14) MM was diagnosed, FGFR3 expression of PB cells was followed at monthly intervals for several months. As a control, BM samples from two healthy donors and from one patient with disseminated carcinoma without BM involvement were studied. All patients gave informed consent.

Cell lines

RPMI 8226 t(4;14) negative and OPM2, LP1, NCI-H929, KMS11 and KMS18 t(4;14) positive MM cell lines were cultured in RPMI medium [Gibco-BRL Life Technologies (GBLT), Cergy Pointoise, France] supplemented with 10% fetal calf serum (FCS, GBLT), except LP1 which was cultured in Dulbecco's modified Eagle's medium (DMEM, GBLT).

Methods

Flow cytometry. Mononuclear cells from BM or PB sample were isolated by ficoll hypaque (lymphocyte separation medium; Eurobio, Les Ulis, France) and washed twice with phosphate-buffered saline (PBS) (GBLT) containing 1% bovine serum albumin (BSA). Then, 2×10^5 cells were stained with each pretitrated antibody in 50 μl of PBS/BSA for 30 min at 4°C, washed twice, and acquired by means of a Becton Dickinson (BD) cell-sorting flow cytometer (FACSort; BD Biosciences, Oxford, UK) with cell Quest v3.1 software (BD Biosciences). Cells were incubated with anti-CD138-fluorescein isothiocyanate (FITC) (clone B-A38; Diaclone, Besancon, France), anti-FGFR3-phycoerythrin (PE) (monoclonal mouse IgG1; clone 136334, R&D systems, Lille, France), and anti-CD19-phycoerythrin cyanin 5 (PC5) (BD Biosciences) antibodies. FGFR3-PE labelling was tested on gated CD138 positive and CD19 negative cells. Between 50 000 and 500 000 cells were used in each test. The gating strategy was optimised to exclude contaminating events and cellular debris.

Real time quantitative-PCR (RQ-PCR) analysis of IGH/MMSET fusion transcripts and FGFR3 transcripts, and FISH. Plasma cells from BM were CD138-selected using a magnetic-activated cell sorting system (Miltenyi Biotec GmbH; Bergisch-Gladbach, Germany; http://www.miltenyibiotec.com). The t(4;14) *IGH-MMSET* rearrangement was searched for in plasma cells from the 200 patients using a RQ-PCR assay that was adapted from Chesi *et al* (1998). Briefly, plasma cell RNAs were extracted using the Rneasy kit (Qiagen, Courtaboeuf, France; http://wwwqiagen.com) and the corresponding cDNA was used for RQ-PCR. This assay efficiently detects the 3 *IGH-MMSET* fusion transcript families using 3 RQ-PCR systems that are normalised to the housekeeping gene *GUS* expression. RQ-PCR primers were: GUS, 5′-CCG AGT GAA GAT CCC CTT TTT A-3′, 5′-GAA AAT ATG TGG TTG GAG AGC TCA TT-3′, and 5′-CCA GCA CTC TCG TCG GTG ACT GTT CA-Tamra; IGH-MMSET: 5′-TTG CAA GGC TCG CAG TGA C-3′ (IMU), and 5′-ACC ACG GTC ACC GTC TCC TCA-3′ (JH), combined with 5-TCT GAA CAG AAA GGG GAC TCT GC-3′ and 5′-AGA ACG GAA GCA TCT GGG CTG GAT-Tamra (MB4-1), 5′-TTC AAC AGG TGG TCT TTG TCT CTT C-3′ and 5′-TCC AGC TAA GAA AGA GTC TTG TCC AAA CAC T-Tamra (MB4-2), and 5′-GGG GCG TCA CCA AAG AAC TG-3′ and 5′-CAG AAA AAG AGT GCA CGC CAG TAT CAC G-Tamra (MB4-3). RQ-PCR positive MM cases were confirmed by FISH using the Vysis LSI IGH/FGFR3 Dual Color Probe (Vysis-Abbott, Voisins-le-Bretonneux, France; http://www.vysis.com) that was hybridised on interphasic purified cells. *FGFR3* transcripts were detected using RQ-PCR, normalised to *GUS* expression. Primers were: 5′-AAG ATC TCC CGC TTC CCG-3′, 5′-CCT GAG GAC AGC CTT GCG-3′, and 5′-AAG CGA CAG GTG TCC CT-Tamra. The 5 RQ-PCR reactions (*IGH-MMSET* MB4-1, MB4-2, and MB4-3 species, *FGFR3*, and *GUS*) were performed independently, each in duplicate (total 10 reactions).

Results

Detection of FGFR3 expression on myeloma cell line and CD138 positive BM plasma cells

t(4;14) positive (OPM2, LP1 and NCI) and t(4;14) negative (RPMI) MM cell lines were first studied for FGFR3 expression. Only the t(4;14) cell lines expressed FGFR3 at variable levels (Fig 1). The median percentage of plasma cells was 12% (range, 0·5–70%) in the 200 BM aspirates that were analysed. In 20 of the 200 cases (10%), FC analysis of mononuclear cells

© 2007 The Authors

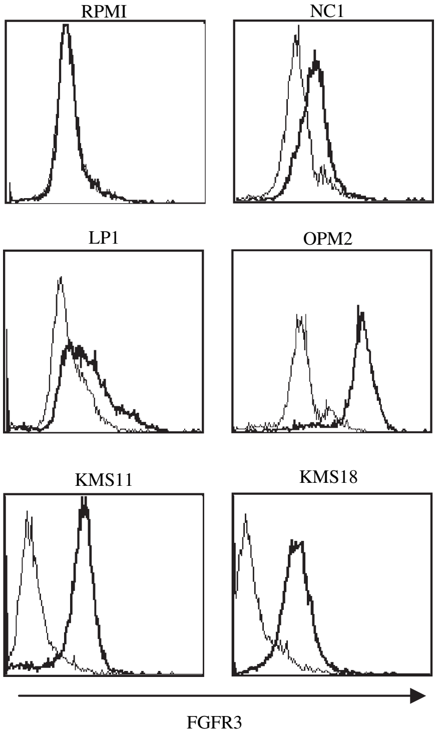

Fig 1. (OPM2, KMS11, KMS18, NCI-H929, LP1) and t(4;14) negative (RPMI) multiple myeloma (MM) cell lines. Thin line indicates control isotype Thick line indicates anti-FGFR3 antibody.

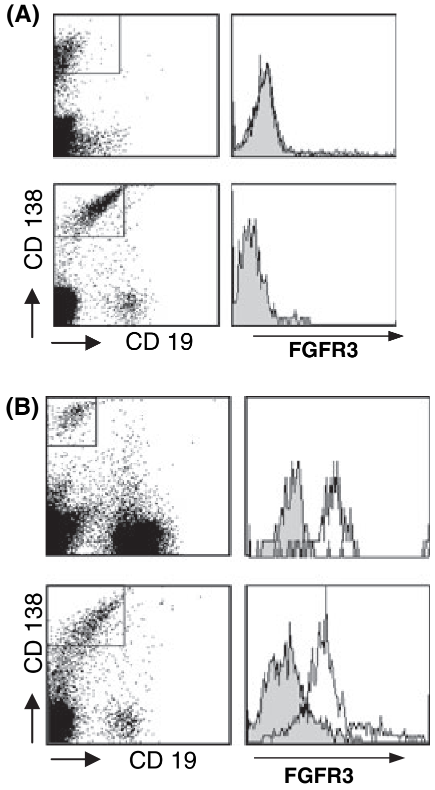

Fig 2. Flow cytometric study of fibroblast growth factor receptor 3 (FGFR3) expression in plasma cells from patients with positive and negative t(4;14) multiple myeloma (MM). Correlation of FGFR3 expression in peripheral blood (PB) and bone marrow (BM) $CD138^+$/$CD19^-$ plasma cells. (A) Flow cytometric study of FGFR3 expression in $CD138^+$/$CD19^-$ PB (upper panels) and BM (lower panels) plasma cells from a patient without t(4;14) MM. FGFR3 expression is analysed on the $CD138^+$/$CD19^-$ gated population. The filled histogram indicates control isotype. Thick line indicates anti-FGFR3 antibody. (B) Flow cytometric study of FGFR3 expression in $CD138^+$/$CD19^-$ PB (upper panels) and BM (lower panels) plasma cells from a patient with t(4;14) MM. The filled histogram indicates control isotype. Thick line indicates anti-FGFR3 antibody.

using FITC-conjugated anti-CD138 and PE-conjugated anti-FGFR3 antibodies detected double-labelled plasma cells. The intensity of PE-related fluorescence was variable, reflecting a moderate to strong expression of FGFR3 (Fig 2B). In contrast, haematopoietic cells from five normal BM and polyclonal plasma cells from a patient with a disseminated carcinoma and hypergammaglobulinaemia were FGFR3 negative (data not shown).

Correlation of FGFR3 expression and t(4,14) translocation

In all patients, t(4;14) was searched for by RQ-PCR analysis of *IGH/MMSET* fusion transcript in purified CD138 positive BM cells. Twenty-four cases (12%) had *IGH/MMSET* fusion transcripts and the presence of t(4;14) was confirmed by FISH. These included the 20 FGFR3 positive cases detected by FC, which were found to express high levels of *FGFR3* mRNA transcript using RQ-PCR. In contrast, in the four remaining IgH/MMSET positive cases, FGFR3 expression was not detected at the protein or transcriptional level. Thus, the results of FGFR3 protein expression by FC and of *FGFR3* transcript by PQ-PCR were correlated in all 24 cases.

The 24 patients with a t(4;14) myeloma were 16 (66%) women and six men. Fourteen (58%) had an IgA MM. They were studied at diagnosis or at relapse in 7 and 17 cases respectively.

FGFR3 expression on BM and circulating peripheral blood plasma cells

In 52 out of the 200 patients, CD138 and FGFR3 expression were analysed by FC concomitantly in BM and in PB mononuclear cells. Circulating CD138 positive plasma cells were detected by FC in 24 patients, of whom 11 had a t(4;14) MM. Of these, FGFR3 expression was detected in both BM and PB cells in nine cases (Fig 2A and B). In the two remaining cases, FGFR3 expression could be detected in PB cells whereas the BM aspirate, which contained only a few plasma cells (<0·1%), had to be considered as negative. The median percentage of circulating plasma cells, as assessed by FC, in patients with and without FGFR3 expression was 1.45% (ranging from 0·4% to 5·8%) and 1·31% (ranging from 0·1% to 10%) respectively. In all the newly diagnosed t(4;14) positive ($n = 7$) and negative ($n = 109$) patients, no correla-

© 2007 The Authors

tion was found between the percentages of BM and circulating plasma cells. PB analysis enabled detection of FGFR3 expression, even in the presence of a very low percentage (0·4%) of circulating CD138 positive plasma cells. In all but two cases (see below), BM or PB CD138 positive cells were uniformly either positive or negative for FGFR3 expression, according to t(4;14) MM status.

Follow up of FGFR3 expression on plasma cells

In eight t(4;14) MM cases, FC analysis of FGFR3 expression on circulating plasma cells was evaluated at monthly intervals for a median time of 6 months (3–10 months), during which patients were on ($n = 7$) or off ($n = 1$) therapy. In four cases, the percentage and intensity of labelling of FGFR3 positive peripheral plasma cells remained stable.

In two patients, the number of circulating FGFR3$^+$/CD138$^+$ plasma cells initially decreased while they were in remission after treatment. When relapse occurred, circulating CD138$^+$ plasma cells were again detected but these cells were FGFR3 negative. Loss of FGFR3 protein expression was corroborated by the absence of detectable *FGFR3* transcript in the purified PB plasma cells of the two patients. In contrast, *IGH/MMSET* fusion transcript was still detectable by quantitative RQ-PCR, and FISH analysis confirmed the loss of the *FGFR3* gene on the der(14) (data not shown).

In the two last patients, a double population (CD138$^+$/FGFR3$^+$ and CD138$^+$/FGFR3$^-$) of plasma cells was detected (Fig 3A). In both cases, FGFR3 positive and negative populations were separated by fluorescent-activated cell sorting (FACS) and analysed by RQ-PCR and FISH. As expected, *FGFR3* and *IGH/MMSET* fusion transcripts were detected in FGFR3 positive populations (Fig 3B). In contrast, in both cases the FGFR3 negative population did not express *FGFR3* transcripts, although they still expressed *IGH/MMSET* fusion transcripts derived from the same breakpoint region. In one patient, FISH analysis was consistent with a loss of the *FGFR3* gene on the der(14) whereas the *FGFR3* gene was still present in the second case (Fig 3C).

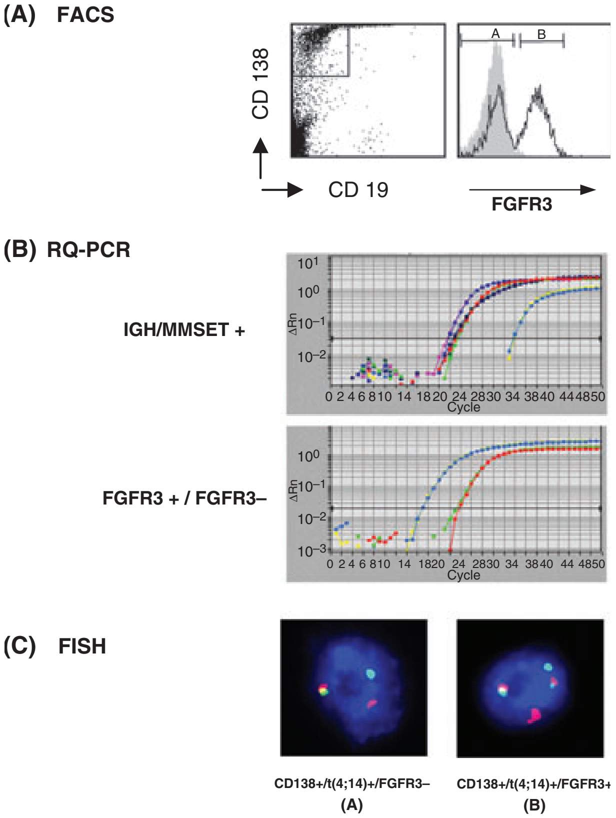

Fig 3. (A) Flow cytometric analysis of FGFR3 expression in BM plasma cells from a patient with t(4;14) MM. FGFR3 expression was analysed on the CD138$^+$/CD19$^-$ gated population. The filled histogram indicates control isotype. Thick line indicates anti-FGFR3 antibody. Two distinct population patterns of CD138$^+$/CD19$^-$ cells were found: one population without FGFR3 expression (A) and one population stained with anti-FGFR3 antibody (B). The FGFR3$^+$ and FGFR3$^-$ cells were separated and both populations were analysed by RQ-PCR (B) and FISH (C). (B) Both fractions were *IGH/MMSET* positive (upper panel); low *FGFR3* transcript expression was found in the FGFR3 protein negative cells. (C) In the FGFR3$^+$ fraction (B), two FISH fusion signals were detected (right), whereas in the FGFR3$^-$ fraction (A) a single fusion signal was detected (left), consistent with a der(14) deletion in these cells.

Discussion

Identification of the t(4;14) translocation and FGFR3 expression is an important step in the management of patients with MM, given its poor prognosis and the potential development of therapy targeted against FGFR3 tyrosine kinase activity. To recognise this subgroup of MM, analysis of CD138-selected BM plasma cells by FISH is the current method of choice (Kuehl & Bergsagel, 2005). As previously suggested (Chesi *et al*, 1998) and herein illustrated, RQ-PCR for detection of *IGH/MMSET* and *FGFR3* transcripts is also useful for the diagnosis and characterisation of t(4;14) MM (Kuehl & Bergsagel, 2005). The detection of the FGFR3 protein, by immuno-histochemistry on decalcified, paraffin-embedded BM biopsies, has also been proposed (Chang *et al*, 2005).

The present data highlighted the usefulness of FC analysis for FGFR3 expression in BM and PB samples of MM patients. This approach is quick to perform and is easily applied to routine laboratory practice in as much as it does not require CD138 purification. In addition, it provides results in 'real time', which is convenient for clinical decision-making. In this series of 200 patients, FC detection of FGFR3 positive cells was consistently associated with the presence of t(4;14) translocation, by FISH and/or by RQ-PCR for *IGH/MMSET* and *FGFR3* transcripts. More importantly, no FGFR3 expression was found when t(4;14) was absent, suggesting that FGFR3 ectopic expression in MM is always related to the t(4;14). Conversely, FGFR3 negative plasma cells were observed in 4/24 (16%) patients who had a t(4;14) MM. In all four cases, *IGH/MMSET* transcripts but not *FGFR3* transcripts were detected by RQ-

© 2007 The Authors

PCR in the myeloma cells. Because of these t(4;14) FGFR3 negative MM, which may represent up to 25–30% of all t(4;14) MM (Keats *et al*, 2003; Santra *et al*, 2003), FISH and/or RQ-PCR remain mandatory to fully identify this subset of MM.

In FGFR3 positive t(4;14) MM, FC study of blood samples regularly detected FGFR3 positive plasma cells. In any patient with MM, the number of circulating plasma cells, as assessed by FC at diagnosis, may be correlated with tumour burden and, in addition, may have prognostic significance (Rawstron *et al*, 1997; Nowakowski *et al*, 2005). Indeed, detection of circulating myeloma cells may identify a group of patients with a high risk of progression and may be used to provide a simple and powerful scoring system when combined with abnormal cytogenetics (Dingli *et al*, 2006). In our series, the percentage of circulating FGFR3 positive plasma cells was not significantly higher in t(4;14) MM when compared with non t(4;14) MM, nor was it significantly higher in newly diagnosed when compared with relapsing patients. The percentage of circulating plasma cells as a predictive value for the outcome of patients with t(4;14) MM is currently under evaluation in our group.

The follow-up of FGFR3 expression on PB plasma cells may be useful to assess the degree of response and the time to progression of patients on therapy, particularly of those who will receive targeted therapy against FGFR3 tyrosine kinase activity. In addition, this may enable the detection of either spontaneous or drug-induced intra-clonal heterogeneity. Indeed, in two patients, we observed the co-existence of $FGFR3^{+}$ and $FGFR3^{-}$ plasma cells circulating subpopulations. Both expressed an identical *IGH/MMSET* transcript, suggesting that they were derived from the same t(4;14) clone. In one case, FISH analysis was consistent with the loss of der(14) in FGFR3 negative t(4;14) cells, as already reported (Santra *et al*, 2003). In the second case, der(14) was detected and the absence of the FGFR3 protein may be due to a partial deletion of the IGH/*FGFR3* fusion region not detected by FISH, or to an as yet unknown silencing mechanism.

In these cases, the co-existing double populations may have been induced by the selective pressure of the chemotherapy. Alternatively, FGFR3 may have an early oncogenic role that is subsequently dispensable in the maintenance of tumour plasma cells. Of note, in one patient, both $FGFR3^{+}$ and $FGFR3^{-}$ subclones coexisted over several months, suggesting the absence of any selective growth advantage of one or other type of cells. The molecular mechanisms and the signification of these subpopulations are currently under investigation in our laboratory.

Taken together, our results show that CD138/FGFR3 immuno-phenotyping using FC is a rapid, easily standardised, sensitive method for detecting the PC compartment expressing FGFR3 in BM and PB of patients with t (4;14) MM. It may be useful for the management of these patients, particularly when developing new therapeutic approaches, such as targeting FGFR3 tyrosine kinase activity, which are required because of the seriousness of this subgroup of MM.

Acknowledgements

We are indebted to Dr Takemi OTSUKI (Okayama, Japan) for kindly providing the KMS11 and KMS18 MM cell lines. We would like to thank Dr S Choquet (Pitié-Salpetrière Hospital, Paris, France), Dr M Divine (Henri Mondor Hospital, Creteil, France), Dr D Ghez (Necker Hospital, Paris, France), Pr D Bouscary (Cochin Hospital, Paris, France) for their help in the management of patient's samples and Pr JC Brouet (Immuno-Hematology Department, Saint-Louis Hospital, Paris, France) for useful discussions. This study was supported by a grant from the Fondation de France. MOC was supported by a fellowship from the Fondation pour la Recherche Medicale (FRM).

References

Chang, H., Sloan, S., Li, D., Zhuang, L., Yi, Q.L., Chen, C.I., Reece, D., Chun, K. & Stewart, A.K. (2004) The t(4;14) is associated with poor prognosis in myeloma patients undergoing autologous stem cell transplant. *British Journal of Haematology*, **125,** 64–68.

Chang, H., Stewart, A.K., Qi, X.Y., Li, Z.H., Yi, Q.L & Trudel, S. (2005) Immunohistochemistry accurately predicts FGFR3 aberrant expression and t(4;14) in multiple myeloma. *Blood*, **106,** 353–355.

Chen, J., Lee, B.H., Williams, I.R., Kutok, J.L., Mitsiades, C.S., Duclos, N., Cohen, S., Adelsperger, J., Okabe, R., Coburn, A., Moore, S., Huntly, B.J., Fabbro, D., Anderson, K.C., Griffin, J.D. & Gilliland, D.G. (2005) FGFR3 as a therapeutic target of the small molecule inhibitor PKC412 in hematopoietic malignancies. *Oncogene*, **24,** 8259–8267.

Chesi, M., Nardini, E., Lim, R.S., Smith, K.D., Kuehl, W.M. & Bergsagel, P.L. (1998) The t(4;14) translocation in myeloma dysregulates both FGFR3 and a novel gene, MMSET, resulting in IGH/MMSET hybrid transcripts. *Blood*, **92,** 3025–3034.

Chesi, M., Brents, L.A., Ely, S.A., Bais, C., Robbiani, D.F., Mesri, E.A., Kuehl, W.M. & Bergsagel, P.L. (2001) Activated fibroblast growth factor receptor 3 is an oncogene that contributes to tumor progression in multiple myeloma. *Blood*, **97,** 729–736.

Dingli, D., Nowakowski, G.S., Dispenzieri, A., Lacy, M.Q., Hayman, S.R., Rajkumar, S.V., Greipp, P.R., Litzow, M.R., Gastineau, D.A., Witzig, T.E. & Gertz, M.A. (2006) Flow cytometric detection of circulating myeloma cells before transplantationin patients with multiple myeloma: a simple risk stratification system. *Blood*, **107,** 3384–3388.

Jaksic, W., Trudel, S., Chang, H., Trieu, Y., Qi, X., Mikhael, J., Reece, D., Chen, C. & Stewart, A.K. (2005) Clinical outcomes in t(4;14) multiple myeloma: a chemotherapy-sensitive disease characterized by rapid relapse and alkylating agent resistance. *Journal of Clinical Oncology*, **28,** 7069–7073.

Keats, J.J., Reiman, T., Maxwell, C.A., Taylor, B.J., Larratt, L.M., Mant, M.J., Belch, A.R. & Pilarski, L.M. (2003) In multiple myeloma, t(4;14)(p16;q32) is an adverse prognostic factor irrespective of FGFR3 expression. *Blood*, **101,** 1520–1529.

Kuehl, W.M. & Bergsagel, P.L. (2005) Early genetic events provide the basis for a clinical classification of multiple myeloma. *American Society of Hematology Education Program Book*, 346–352.

© 2007 The Authors

L'Hôte, C.G.M. & Knowles, M.A. (2005) Cell responses to FGFR3 signalling: growth, differentiation and apoptosis. *Experimental Cell Research*, **304,** 417–431.

Li, Z., Zhu, Y.X., Plowright, E.E., Bergsagel, P.L., Chesi, M., Patterson, B., Hawley, T.S., Hawley, R.G. & Stewart, A.K. (2001) The myeloma-associated oncogene fibroblast growth factor receptor 3 is transforming in hematopoietic cells. *Blood*, **97,** 2413–2419.

Nowakowski, G.S., Witzig, T.E., Dingli, D., Tracz, M.J., Gertz, M.A., Lacy, M.Q., Lust, J.A., Dispenzieri, A., Greipp, P.R., Kyle, R.A. & Rajkumar, S.V. (2005) Circulating plasma cells detected by flow cytometry as a predictor of survival in 302 patients with newly diagnosed multiple myeloma. *Blood*, **106,** 2276–2279.

Plowright, E.E., Li, Z., Bergsagel, P.L., Chesi, M., Barber, D.L., Branch, D.R., Hawley, R.G. & Stewart, A.K. (2000) Ectopic expression of fibroblast growth factor receptor 3 promotes myeloma cell proliferation and prevents apoptosis. *Blood*, **95,** 992–998.

Rawstron, A.C., Owen, R.G., Davies, F.E., Johnson, R.J., Jones, R.A., Richards, S.J., Evans, P.A., Child, J.A., Smith, G.M., Jack, A.S. & Morgan, G.J. (1997) Circulating plasma cells in multiple myeloma: characterization and correlation with disease stage. *British Journal of Haematology*, **97,** 46–55.

Santra, M., Zhan, F., Tian, E., Barlogie, B. & Shaughnessy, Jr, J. (2003) A subset of multiple myeloma harboring the t(4;14)(p16;q32) translocation lacks FGFR3 expression but maintains an IGH/MMSET fusion transcript. *Blood*, **101,** 2374–2376.

Trudel, S., Ely, S., Farooqi, Y., Affer, M., Robbiani, D.F., Chesi, M. & Bergsagel, P.L. (2004 a) Inhibition of fibroblast growth factor receptor 3 induces differentiation and apoptosis in t(4;14) myeloma. *Blood*, **103,** 3521–3528.

Trudel, S., Li, Z.H., Wei, E., Wiesmann, M., Chang, H., Chen, C., Reece, D., Heise, C. & Stewart, K. (2004b) CHIR-258, a novel, multi-targeted tyrosine kinase inhibitor for the potential treatment of t(4;14) multiple myeloma. *Blood*, **105,** 2941–2948.

Winkler, J.M., Greipp, P.R. & Fonseca, R. (2003) t(4;14)(p16;q32) is strongly associated with a shorter survival in myeloma patients. *British Journal of Haematology*, **120,** 170–171.

© 2007 The Authors
