# Supplementary material for: Expression of Concern: Prognostic value of circulating plasma cells in patients with multiple myeloma: A meta-analysis
Source: PLoS One. 2023 Feb 21;18(2):e0282230. doi: 10.1371/journal.pone.0282230 (PMC9942954; doi:10.1371/journal.pone.0282230)
Supplement: S1 File — (ZIP) [file pone.0282230.s001.zip › primary data/excluded research/2007 New criteria to identify risk of progression in monoclonal gammopathy of uncertain significance and smoldering multiple myeloma based on multiparameter.pdf]

From www.bloodjournal.org by guest on November 22, 2016. For personal use only.



# New criteria to identify risk of progression in monoclonal gammopathy of uncertain significance and smoldering multiple myeloma based on multiparameter flow cytometry analysis of bone marrow plasma cells

Ernesto Pérez-Persona,[1] María-Belén Vidriales,[1,2] Gema Mateo,[1] Ramón García-Sanz,[1,2] Maria-Victoria Mateos,[1] Alfonso García de Coca,[3] Josefina Galende,[4] Guillermo Martín-Nuñez,[5] José M. Alonso,[6] Natalia de las Heras,[7] José M. Hernández,[8] Alejandro Martín,[9] Consuelo López-Berges,[1] Alberto Orfao,[2,10] and Jesús F. San Miguel[1,2]

[1]Department of Hematology, University Hospital, Salamanca; [2]Centro de Investigación del Cancer, Universidad de Salamanca/Consejo Superior de Investigaciones Cientificas (CSIC), Salamanca; [3]Department of Hematology, University Hospital, Valladolid; [4]Department of Hematology, Hospital Comarcal del Bierzo, Ponferrada, León; [5]Department of Hematology, Hospital Nuestra Señora del Puerto de Plasencia, Cáceres; [6]Department of Hematology, Hospital Río Carrión, Palencia, Caceres; [7]Department of Hematology, Complejo Hospitalario, León; [8]Department of Hematology, General Hospital, Segovia; [9]Department of Hematology, Hospital Virgen de la Concha, Zamora; and [10]Department of Cytometry, University of Salamanca, Salamanca, Spain

**Monoclonal gammopathy of uncertain significance (MGUS) and smoldering multiple myeloma (SMM) are plasma cell disorders with a risk of progression of approximately 1% and 10% per year, respectively. We have previously shown that the proportion of bone marrow (BM) aberrant plasma cells (aPCs) within the BMPC compartment (aPC/BMPC) as assessed by flow cytometry (FC) contributes to differential diagnosis between MGUS and multiple myloma (MM). The goal of the present study was to investigate this parameter as a marker for risk of progression in MGUS (n = 407) and SMM (n = 93). Patients with a marked predominance of aPCs/BMPC ( ≥ 95%) at diagnosis displayed a significantly higher risk of progression both in MGUS and SMM (*P*< .001). Multivariate analysis for progression-free survival (PFS) selected the percentage aPC/BMPC (≥ 95%) as the most important independent variable, together with DNA aneuploidy and immunoparesis, for MGUS and SMM, respectively. Using these independent variables, we have identified 3 risk categories in MGUS (PFS at 5 years of 2%, 10%, and 46%, respectively; *P*< .001) and SMM patients (PFS at 5 years of 4%, 46%, and 72%, respectively; *P* < .001). Our results show that multiparameter FC evaluation of BMPC at diagnosis is a valuable tool that could help to individualize the follow-up strategy for MGUS and SMM patients. (Blood. 2007;110:2586-2592)**

© 2007 by The American Society of Hematology

## Introduction

Monoclonal gammopathy of uncertain significance (MGUS) and smoldering multiple myeloma (SMM) represent 2 forms of asymptomatic plasma cell (PC) disorders that share in common a variable period of stable disease but which may eventually progress to symptomatic multiple myeloma (MM). MGUS is the most common plasma cell disorder, whose incidence increases with age, affecting approximately 3% of population more than 50 years of age and up to 10% in those more than 70 years of age.[1-3] Diagnosis of MGUS is characterized by the presence of a monoclonal immunoglobulin in serum of no more than 3 g/dL, and less than 10% of plasma cells in bone marrow (BM), in the absence of end-organ damage related to the proliferation of monoclonal plasma cell.[1,2,4] Smoldering multiple myeloma (SMM) is also an asymptomatic plasma cell disorder that fulfills the diagnostic criteria of multiple myeloma (MM), although it is characterized by the absence of hypercalcemia, renal failure, anemia, and bone lytic lesions (CRAB symptoms).[2] Its estimated prevalence ranges from 15% to 44% of newly diagnosed MM.[1,5,6]

When progression occurs, the majority of cases evolve to overt MM. The rate of progression is significantly lower for MGUS cases (approximately 1% per year[7]) than for SMM (10% per year[8]). Therefore, a different follow-up strategy is needed in these 2 groups of monoclonal plasma cell disorders, with a closer follow-up in SMM than in MGUS. However, it would be most valuable to have individualized information on factors associated with the risk of progression at diagnosis to determine patient follow-up strategy according to their individual risk of progression. For both disorders these factors include the amount of monoclonal component, the presence of Bence Jones proteinuria, the isotype of the monoclonal immunoglobulin (IgA), and an abnormal serum free light chain ratio.[1,4,5,8-11] Particularly in the case of SMM, the current standard of care is close follow-up without treatment until MM symptoms develop. Nevertheless, this policy may change with the availability of novel noncytotoxic agents. The obvious cohort of SMM patients in which these new agents should be tested are those at high risk of progression. Therefore, it is most important to identify this patients group in order to define the final value of "preventive treatment approaches."

In recent years, multiparameter flow cytometry (FC) immunophenotyping has been increasingly used in the setting of different haematologic malignancies.[12-17] Concerning plasma cell dyscrasias, it has been shown that, based on the expression of several

Submitted May 7, 2007; accepted June 11, 2007. Prepublished online as *Blood* First Edition paper, June 18, 2007; DOI 10.1182/blood-2007-05-088443.

An Inside *Blood* analysis of this article appears at the front of this issue.

Presented in part at the European Hematology Association 11th annual congress, 2006 (Amsterdam), as an oral presentation.

The publication costs of this article were defrayed in part by page charge payment. Therefore, and solely to indicate this fact, this article is hereby marked "advertisement" in accordance with 18 USC section 1734.

© 2007 by The American Society of Hematology

From www.bloodjournal.org by guest on November 22, 2016. For personal use only.



markers, normal and myelomatous plasma cells (PCs) can be easily differentiated.[12,18,19] We have previously shown that phenotypically aberrant PC (aPC) correspond to clonal PCs, whereas PCs with a normal phenotype are polyclonal.[12,20] Moreover, the proportion of aPCs/BMPC was the most important criteria for differential diagnosis between MGUS and MM patients.[12] Thus, in the majority of MM patients, almost all PCs (> 95%) display an aberrant phenotype, whereas in MGUS patients, normal and malignant plasma cells coexist.[12] Based on this background, we wanted to investigate whether or not this valuable parameter (aPCs/BMPCs) for differential diagnosis between MGUS and MM could also help to predict the risk of transformation of MGUS and SMM into symptomatic disease.

**Table 1. Clinical and biological characteristics of MGUS and SMM patients according to the type of immunoglobulin**

| Characteristics | MGUS (n = 407) | SMM (n = 93) |
|---|---|---|
| Sex, male/female, no. | 205/202 | 46/47 |
| Age, y (range) | 71 (26-95) | 69 (39-88) |
| Serum MC, mg/dL (range) | 1.60 (0.3-3.0) | 2.37 (0.8-6.5) |
| Immunoparesis, no. (%) | 90 (25%) | 47(52%) |
| BM PC by morphology, % (range) | 4 (1-10) | 14 (4-55) |
| BM PC by FC, % (range) | 1.0 (0.1-37) | 2.9 (0. 9-27) |
| Abnormal PC, % (range) | 73 (0-100) | 97 (17-100) |
| Patients with greater than 95% aPC, % (%) | 73 (18) | 56 (60) |
| Presence of BJ proteinuria, no. (%) | 40 (17) | 14 (22) |
| Aneuploidity, no. (%) | 229 (58) | 62 (67) |
| Progression, no. (%) | 33 (8) | 40 (43) |

## Patients, materials, and methods

### Patients

A total of 500 consecutive patients diagnosed between January 1996 and September 2003 who fulfilled the criteria of monoclonal gammopathy of uncertain significance (MGUS) (n = 407) or smoldering multiple myeloma (SMM) were included in this study. Informed written consent was obtained from all the patients included in accordance with the Human Investigations Committee at the University Hospital of Salamanca and the Declaration of Helsinki.

MGUS and SMM were defined according to the International Myeloma Working Group.[2] MGUS was characterized by the evidence of monoclonal component (MC) of less than 3 g/dL, absence or less than 1 g/24 hours Bence Jones proteinuria, and BMPC infiltration of less than 10%. SMM was defined by MC of 3 g/dL or greater, and/or BMPC infiltration of 10% or greater, and/or the presence of Bence Jones proteinuria greater than 1 g/dL; in all cases, absence of end-organ damage (lytic bone lesions, hypercalcemia, renal insufficiency, or anemia) was required. With respect to the criteria used for the definition of SMM,[2] 14 of 93 cases (15%) had both more than 10% of PCs in BM and high MC (≥ 3 g/dL), 64 patients (68%) had 10% or greater PCs in the BM but with lower MC (< 3 g/dL). The remaining 15 patients (16%) had an infiltration of BMPC lower than 10%, and diagnosis of SMM was performed in these cases on the basis of "reconfirmed" MC levels of greater than 3.0. Immunoparesis was defined as a reduction (below the lower normal limit) in the levels of 1 or 2 immunoglobulin (Ig), with respect to the values of the corresponding uninvolved Ig. Progression to multiple myeloma was established according to the criteria defined by the International Working Group.[2]

With a minimum follow-up of 24 months, at the close of this study the median follow-up was 56 months (range, 24-187 months). Thirty-three MGUS cases (8%) and 47 SMM patients (43%) have already progressed. Progression was defined as transformation from MGUS or SMM into symptomatic myeloma, amyloidosis, or chronic lymphoproliferative disease. The median time to progression in MGUS patients was 44 months (range, 4.4-120); the majority of patients progressed to symptomatic MM (n = 26; 79%), 4 cases progressed to chronic lymphoproliferative disease (12%), 2 cases progressed to amyloidosis (6%), and 1 progressed to plasmatic cell leukemia (3%). The median time to progression for SMM patients was 18 months (range, 2-92 months), and all of them evolved into symptomatic MM.

The following variables collected at diagnosis were included in the analysis: Eastern Cooperative Oncology Group (ECOG) status, hemoglobin (Hb), platelet count, erythrocyte sedimentation rate (ESR), serum creatinine, serum liver function tests, $\beta_2$-microblobulin, C reactive protein (CRP), total protein, albumin and MC serum levels, presence of urine MC, serum concentration of polyclonal Ig, percentage of PCs in the BM aspirate evaluated by optical microscopy, and skeleton X-ray (Table 1). The percentage of BMPC was calculated after counting 200 cells. Quantification of the MC was carried out using serum protein electrophoresis on cellulose acetate. Serum polyclonal Ig levels were measured by nephelometry.

### Immunophenotypic studies

Immunophenotypic analyses were performed on erythrocyte-lysed $K_3$-EDTA (ethylenediaminetetraacetic acid) anticoagulated whole BM samples. A total of $2 \times 10^6$ cells per tube were stained, lysed, and then washed using a direct immunofluorescence technique and multicolor staining, which was aimed at the specific identification and immunophenotypic characterization of PCs: CD38-FITC/CD56-PE/CD19-PerCP-Cy5/CD45-APC. In specific cases additional staining for cytoplasmatic Ig light chains were used to clarify the polyclonal vs monoclonal nature of PCs. The cIgκ/cIgλ staining was performed in a 4-color tube including simultaneous surface staining for CD38 plus either CD45, CD56, or CD19, depending on the type of antigenic aberrancy. All monoclonal antibodies used were purchased from Becton/Dickinson Biosciences (BDB; San Jose, CA) except for CD38-FITC (Caltag Laboratories, San Francisco CA). Acquisition was performed in a FACSCalibur flow cytometer (BDB) using double-step procedure using the CellQUEST software (BDB). In the first step, a total of $20 \times 10^3$ events from the total BM cellularity were measured; in the second step, information on a minimum of $3 \times 10^5$ cells was acquired and stored through an electronic "live-gate" drawn on the side scatter (SSC)/CD38$^{++}$ events where PCs are included (Figure 1). In cases with very low numbers of PCs, the acquisition of cells was increased in order to reach the minimum target of PC events (≥ 1000). We first identified PCs based on the expression of CD138/high expression of CD38 with an interm SSC. Once PCs were identified, we focused our analysis on the PC compartment, and within it, we discriminated between PC with a normal and aberrant phenotype (polyclonal and clonal PCs, respectively).[21-23] For this purpose, we used a validated immunophenotypic approach[23,24] where the absence of CD19 and/or CD45, the decreased expression of CD38, and overexpression of CD56 were used for the identification of aberrant phenotypes on PC (Table 2) . For each case analyzed, the following data were recorded: (i) total percentage of PCs from the whole nucleated BM celullarity and (ii) percentage of abnormal plasma cells (aPC) within the BMPC compartment. The latter parameter will be referred to in the text as aPC/BMPC.

DNA index assessed by flow cytometry was performed using a double-staining procedure for nuclear DNA (with propidium iodide) and surface antigens (with anti-CD38 plus anti-CD138 monoclonal antibodies) as previously described,[25] and the analysis was performed specifically on the PC (CD38 plus CD138$^{+}$) cells. Aneuploidy was defined as hypo- and hyperdiploidy bases on flow cytometry assessment.

The analysis by FC and the evaluation of the disease status, including the decision that therapy was needed, were performed by independent observers.

### Statistical analyses

The Chi square and the Mann-Whitney U tests were used to estimate the statistical significance of differences observed between groups. Survival curves were plotted according to the Kaplan and Meier method, using log-rank and Breslow tests for comparison. In the univariate analysis for progression-free survival (PFS), the following variables were tested: age, sex, hemoglobin, heavy chain, light chain, amount of MC, renal function, percentage of BMPC by optical microscope, percentage of BMPC by

From www.bloodjournal.org by guest on November 22, 2016. For personal use only.



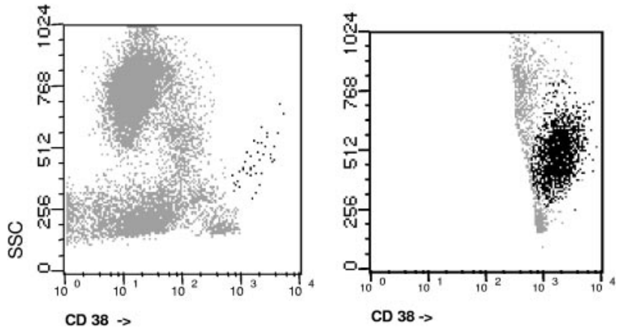


**Figure 1. Gating strategy for PC analysis by multiparametric flow cytometry.**

multiparameter FC, percentage of aPC/BMPC, presence of immunoparesis, DNA ploidy, and presence of Bence Jones proteinuria. Multivariate analysis[15] was performed to explore the independent effect of variables that showed a significant influence on progression-free survival in the univariate analysis. The Cox regression model that was used employed the stepwise methods "forward conditional" as the variable selection model. Quantitative parameters were considered both as continuous as well as dichotomic variables.

All statistical analyses were performed using SPSS software (version 12.5; SPSS, Chicago, IL).

## Results

The clinical and biologic characteristics of MGUS and SMM patients analyzed are summarized in Table 1. Median age (70 years) at diagnosis and sex distribution was similar in both groups. The frequency of IgG paraprotein was slightly higher in MGUS than in SMM (75% vs 64%) whereas the opposite figure was observed for IgA (21% vs 34%). The median value of the serum MC was 1.6 g/dL (range, 0.3 to 3.0 g/dL) and 2.37 g/dL (range, 0.8 to 6.5 g/dL) for MGUS and SMM, respectively. The light chain was κ in 73% (n = 298) of the cases of MGUS patients and in 65% of SMM cases. Immunoparesis was present in 25% of MGUS patients (18% had decreased levels of only one Ig and 7% had low levels of 2 Igs) and in 52% of SMM patients (22% of one Ig and 30% of both chains). Bence Jones proteinuria was detected in 40 (17%) MGUS and 14 (22%) SMM patients. The median percentage of bone marrow plasma cell PC (BMPC) by conventional morphology was 4% and 14% in MGUS and SMM, respectively.

As expected, multiparameter FC showed lower percentages of BMPC than morphology, probably due to a dilutional effect. The median value of PC infiltration by FC was significantly higher in patients with SMM than in MGUS (median 2.9% vs 1.0%, $P < .001$). When the analysis was restricted to the PC compartment and we discriminated between normal and phenotypically aPC (clonal PC), we observed that in SMM, aberrant PC markedly predominate (median 97% aPC from the total BMPC cellularity) whereas in MGUS the distribution of abnormal/normal PC is more balanced, with 73% of PC displaying an aberrant phenotype. Moreover, if we consider the cut-off value of more than 95% aPC/BMPC, which was previously used for differential diagnosis between MGUS and MM (it should be remembered that in symptomatic MM almost all PC [≥ 95%] display an aberrant phenotype), we observed that although 60% of SMM were above this threshold, only 18% of MGUS had 95% or greater aPC ($P < .001$).

Interestingly, the percentage of phenotypically aPC/BMPC at diagnosis allowed the discrimination of 2 groups of patients with significantly different risks of progression to overt MM, both in MGUS and in SMM patients. Thus, in the MGUS group, 24% of patients (18/73) with more than 95% aPC/BMPC progressed with a median time to progression of 107 months, whereas only 4% (15/330) of patients progressed in the group of cases with less than 95% of aPC/BMPC ($P < .001$), with a cumulative probability of progression at 5 years of 25% versus 5%, respectively (Figure 2A). Similarly, within SMM patients the rate of progression to symptomatic MM for cases with more or less than of 95% aPC/BMPC was 63% (36/56) versus 10% (4/37) ($P < .001$) (Figure 2B), with a cumulative probability of progression at 5 years of 64% versus 8%, respectively.

Other factors associated with a significant impact on PFS in both groups of patients included bone marrow plasma cells infiltration by conventional morphology ($P < .002$), overall percentage of PC evaluated by flow cytometry ($P < .01$), and immunoparesis ($P < .001$) (Table 3). In addition, in MGUS patients, a Bence Jones proteinuria higher than 0.2 g/24 hours ($P < .001$), DNA aneuploidy ($P = .01$), and MC levels of 2 g/dL or greater ($P < .001$) also had significant impact on PFS (Table 3). Other variables such as sex, isotype of the MC Ig, $\beta_2$-microglobulin serum levels, ESR, hemoglobin, and C reactive protein were not associated with a different incidence of disease progression.

Interestingly, when we focused on patients with low risk of progression based on conventional criteria, such as low amounts of MC (< 2 g/dL for MGUS and < 3g/dL for SMM) or the absence of immunoparesis, the evaluation of the percentage of aPC/BMPC still allowed the discrimination of 2 groups of patients at different risks of progression. Thus, among cases with low MC, those patients with 95% or greater aPC/BMPC showed lower PFS than those with less than 95% aPC/BMPC, with significant impact on MGUS ($P < .002$) as well as SMM ($P < .001$) (Figure 3). In the same way, upon restricting analysis to patients with no immunoparesis, cases with 95% or greater of aPC/BMPC had poor prognosis in both diagnostic categories (SMM and MGUS) ($P < .02$) (Figure 3). Similarly, the presence of at least or less than 95% aPC/BMPC was able to discriminate 2 prognostic subgroups within patients with high BMPC infiltration or high MC in MGUS as well as SMM patients (data not shown).

In order to explore whether or not the percentage of immunophenotypically aPC/BMPC evaluated by FC was an independent prognostic factor for PFS among MGUS and SMM patients, a multivariate analysis was performed. Interestingly, in both diagnostic groups the sole common variable with independent prognostic value for PFS was the aPC/BMPC ($P < .001$ for MGUS and $P = .003$ for SMM patients). In the MGUS group the DNA index also had independent prognostic value ($P = .001$), with a risk of progression increased by a factor of 8.2 in cases with 95% or greater of aPC/BMPC and by 4.6 in patients with DNA aneuploidy.

**Table 2. Aberrant phenotypic profile in SMM and MGUS patients**

| CD45 | CD19 | CD56 | % |
|---|---|---|---|
| − | − | ++ | 50 |
| − | − | − | 24 |
| −/dim | − | + | 11 |
| − | + | ++ | 8 |
| Dim | − | − | 5 |
| − | + | − | 1 |
| + | Dim | ++ | 1 |

All cases were CD38+.
− indicates not present; +, positive; and ++, strong expression.

From www.bloodjournal.org by guest on November 22, 2016. For personal use only.



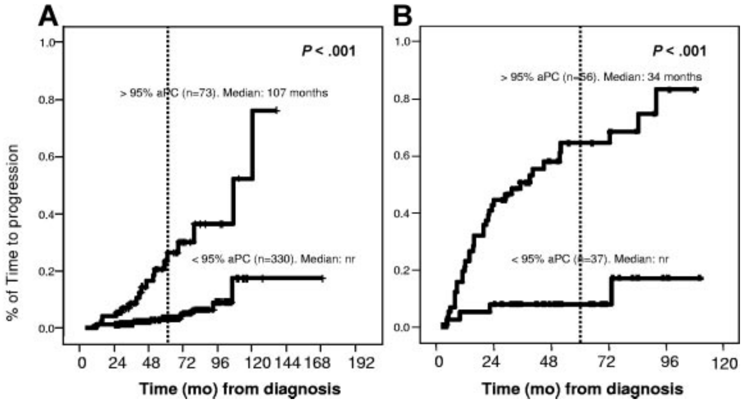


**Figure 2. Time to progression in MGUS and SMM according to the percentage of immunophenotypically aberrant plasma cells.** (A) For MGUS, the median time to progression (TTP) was 107 months versus not reached (nr) for patients with 95% or greater versus less than 95% aberrant PC, respectively ($P < .001$). (B) For SMM, the median TTP was 34 months vs not reached for patients with 95% or greater versus less than 95% aberrant PC, respectively ($P < .001$).

In contrast, for the SMM patients, presence of immunoparesis was selected as also having independent prognostic value for PFS ($P = .02$), with a risk of progression to symptomatic MM increased by a factor of 5.4 in cases with 95% or greater of aPC/BMPC and by 2.5 in patients with low levels of one or more uninvolved immunoglobulin at diagnosis.

Based on variables with independent prognostic value for MGUS transformation (≥ 95% aPC/BMPC and DNA aneuploidy), we established a prognostic index by assigning 1 point for each adverse factor. Accordingly, 3 risk groups of MGUS patients were defined: cases with no risk factors (PFS at 5 years of 2%), cases with 1 risk factor (10% PFS at 5 years), and patients with both risk factors (46% PFS at 5 years) (Figure 4A). The same approach was used for SMM (but now based on ≥ 95% aPC/BMPC and immunoparesis). The PFS at 5 years was 4%, 46%, and 72% for patients with none, 1, or 2 risk factors, respectively ($P < .001$) (Figure 4B).

## Discussion

The term MGUS, introduced by Kyle more than 25 years ago,[26] denotes the presence of a monoclonal protein without evidence of multiple myeloma, amyloidosis, macroblobulinaemia, or other related plasma cell lymphoproliferative disorders.[26] Since SMM was first described by Kyle and Greipp and Alexanian in 1980,[27,28] several series of asymptomatic MM have been reported.[9,29-33] These patients do not display evidence of end-organ damage and are biologically similar to patients with MGUS, although the risk of developing a symptomatic myeloma is much higher.[8] Patients should not be treated unless progression occurs, and the monitoring follow-up has to be closer for SMM than it is for MGUS. Several efforts have been made to identify parameters that will probably predict progression from MGUS or SMM to active MM. For MGUS, the size of serum M

**Table 3. Clinical and biological parameters associated with risk of progression of MGUS and SMM in the univariate analysis**

| | MGUS | | | SMM | | |
|---|---|---|---|---|---|---|
| | Median PFS, mo | Progression no. (%) | P* | Median PFS, mo | Progression no. (%) | P* |
| **Immunoparesis** | | | | | | |
| None | Nr | 12/266 (4.5) | < .001 | Nr | 8/42 (19) | < .001 |
| 1 or 2 Ig | 120 | 17/91 (18) | — | 31 | 31/47 (66) | — |
| **Percentage of BMPC (optical microscope)†** | | | | | | |
| Less than 5% or 15% | Nr | 11/251 (4) | .003 | Nr | 17/57 (30) | < .002 |
| At least 5% or 15% | Nr | 21/144 (15) | — | 34 | 23/36 (64) | — |
| **% of BMPC (flow cytometry)‡** | | | | | | |
| Less than 1% or 5% | Nr | 7/196 (3) | < .006 | Nr | 17/64 (26) | < .001 |
| At least 1% or 5% | Nr | 25/210 (12) | — | 20 | 22/28 (78) | — |
| **MC** | | | | | | |
| Less than 2 g/dL MGUS and less than 3 g/dL SMM | Nr | 11/279 (4) | < .001 | 84 | 23/64 (36) | .078 |
| At least 2 g/dL MGUS and at least 3 g/dL SMM | Nr | 20/111 (18) | — | 31 | 17/29 (58) | — |
| **Light chain** | | | | | | |
| κ | 120 | 23/216 (10) | .02 | 72 | 27/59 (46) | 0.46 |
| λ | 9Nr | 6/165 (3) | — | 92 | 13/33 (39) | — |
| **DNA ploidy** | | | | | | |
| Diploid | 120 | 11/172 (6) | .019 | Nr | 11/31 (43) | 0.31 |
| Aneuploid | Nr | 18/108 (17) | — | 73 | 21/42 (50) | — |
| **Bence-Jones proteinuria** | | | | | | |
| Less than 0.2 g/24 h | 120 | 10/216 (5) | < .001 | 92 | 26/59 (44) | 0.9 |
| At least 0.2 g/24 h | 106 | 7/26 (26) | — | Nr | 2/5 (40) | — |

Nr indicates not reached; and —, not applicable.

* Determined by log-rank test.

† Cut-off point of bone marrow plasma cell infiltration of 5% for MGUS and 15% for SMM.

‡ Cut-off point of bone marrow plasma cell infiltration by flow cytometry of 1% for MGUS and 5% for SMM.

From www.bloodjournal.org by guest on November 22, 2016. For personal use only.



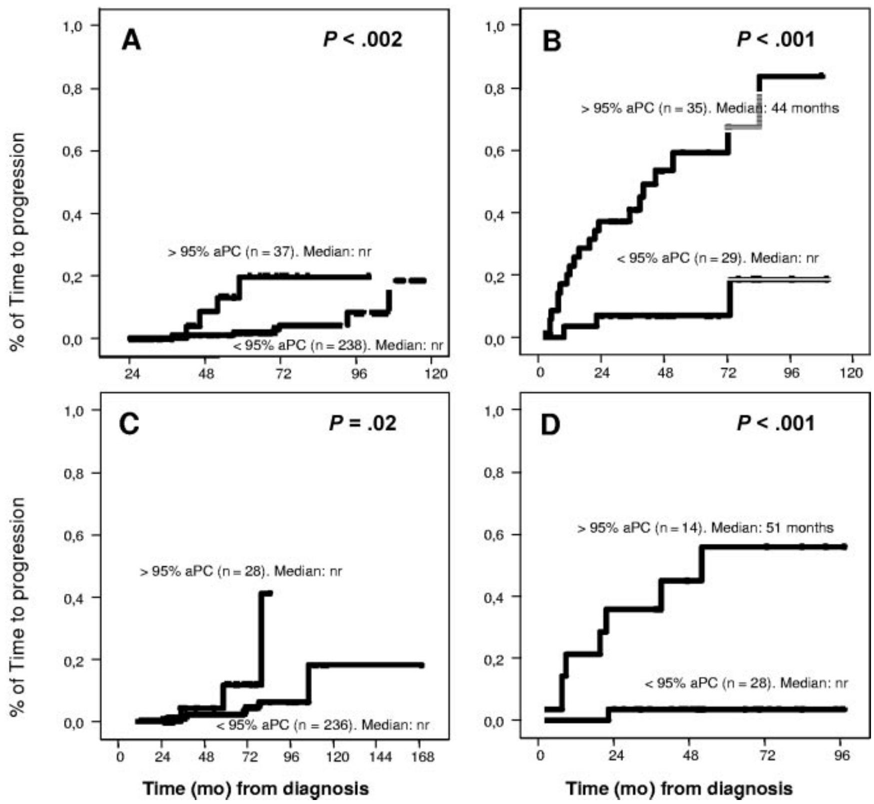


**Figure 3. Impact of percentage of abnormal PC in TTP for patients at low risk.** Patients with low MC (< 2 g/dL for MGUS and < 3 g/dL for SMM): (A) TTP was longer in MGUS patients with less than 95% aberrant PC ($P < .002$). (B) In SMM patients, the median TTP was 44 months versus not reached for patients with greater than or equal to 95% or less than 95% aberrant PC ($P < .001$). Patients without immunoparesis: (C) TTP was longer in MGUS patients with less than 95% aberrant PC ($P = .02$). (D) In SMM patients, the median TTP was 51 months versus not reached for patients with greater than or equal to 95% or less than 95% aberrant PC ($P < .001$).

protein,[4,9] as well as the IgA isotype,[9] an abnormal serum free light chain ratio,[11] detectable BJ protein excretion, more than 5% of PC in BM, and presence of immunoparesis[9] have been identified as predictors of progression. Moreover, Rajkumar et al[11] have recently shown that the combination of a high M component (> 1.5g/dL), a monoclonal protein other than IgG, and an abnormal serum free light chain ratio is associated with a high risk of progression (58% at 20 years compared with 5% when none of these risk factors were present). As far as SMM is concerned, it has been shown that the risk of progression is increased in cases with MC levels of greater than 3 g/dL,[5,8,9,32] IgA isotype,[8,9] BJ protein excretion greater than 50 mg/24 hours,[9] evolving SMM type,[29] greater than 10% of PC in BM,[9] and occult bone lesions on magnetic resonance imaging (MRI).[8,33] It should be noted that the presence of immunoparesis has been associated with a high risk of progression[9] in MGUS but not in SMM.[8]

Multiparameter FC is an increasingly widely used technique that can be applied to the identification of PC among all BM cells, as well as the discrimination of phenotypically abnormal plasma cells from their normal counterpart. The antigens most frequently used for the identification of aberrant PC phenotype include CD19, CD45, and CD56 in combination with CD38/CD138.[12,18,23-25] Thus, the overexpression of CD56 together with the absence of reactivity for CD19 and for CD45 and/or decreased amounts of CD38 have been found to be common characteristics of MM plasma cells.[12,18,34] Such aberrant phenotypes of BM plasma cells have been used for the study of minimal residual disease in MM, and it has been suggested that the persistence of immunophenotypically abnormal PC in the BM after treatment is associated with a worse clinical outcome.[13,21-23,35] In addition, we have shown that the proportion of aPC/BMPC was the most important criteria for differential diagnosis between MGUS and MM patients.[12] Nevertheless, up until now the potential value of PC immunophenotypic investigation for predicting the risk of progression of MGUS and SMM patients has not been explored. Based on this background, we wanted to investigate the potential impact of the detection of

**Figure 4. TTP in MGUS and SMM according to the score system.** (A) The score system for MGUS was built on the basis of the percentage of immunophenotypically aberrant PC within the BMPC compartment (< 95% aberrant PC, score of 0; ≥ 95%, score of 1) and DNA index: aneuploid (score of 1) or diploid (score of 0). In patients with a score of 1, the median TTP has not been reached; in patients with a score of 2, the median TTP is 120 months; and in patients with a score of 3, the median PFS is 61 months ($P < .001$). (B) The score system for SMM was built on the basis of the percentage of immunophenotypically aberrant PC within the BMPC compartment (< 95% aberrant PC, score of 0; ≥ 95%, score of 1) and the presence (score of 1) or absence (score of 0) of immunoparesis. In patients with a score of 1, the median TTP has not been reached; in patients with a score of 2, the median TTP is 73 months; and in patients with a score of 3, the median TTP is 23 months ($P < .001$).

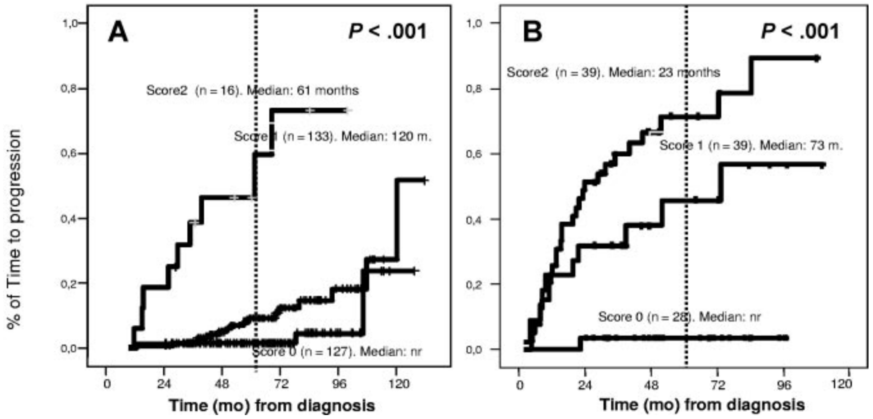

From www.bloodjournal.org by guest on November 22, 2016. For personal use only.



aPC/BMPC on the risk of transformation of MGUS and SMM into symptomatic disease. In our study, at 5 years, the risk of progression was 25% and 64% respectively, for MGUS and SMM cases in which the majority of PC have an aberrant phenotype (≥ 95% aPC/BMPC). These figures are clearly higher than those of patients with less than 95% aPC/BMPC, in which the risk of progression at 5 years is 5% and 8% for the MGUS and SMM group, respectively. It should be emphasized that the immunophenotypic strategy used here is very simple and cost-effective, because it only requires a tube sample with 4 monoclonal antibodies, which results in low cost.

Based on the 2 parameters with independent value in the multivariate analysis (≥ 95% aPC/BMPC, DNA aneuploidy for MGUS, ≥ 95% aPC/BMPC, and presence of immunoparesis for SMM), we propose a simple scoring system, assigning 1 point to each adverse variable, which allows prognostic stratification of MGUS and SMM patients at diagnosis into the 3 risk categories. The cumulative probability of progression from MGUS to MM at 5 years was significantly different for the 3 subgroups: 2%, 10%, and 46%, respectively. A similar pattern was observed for SMM with a risk of progression to symptomatic disease at 5 years of 4%, 46%, and 72%, respectively, for patients with none, 1, or 2 risk factors. As mentioned above, the Mayo Clinic group has recently proposed a new risk classification based on free light chain measurements.[11] Of particular interest would be to prospectively explore whether free light chain and percentage aPC represent independent prognostic factors.

The current standard of care for MGUS and SMM is follow-up without treatment until symptomatic disease develops, but this policy is associated with a significant emotional burden and high cost. Inclusion of multiparameter FC analyses of the BMPC population in the diagnostic evaluation of MGUS and SMM patients could be of a great help for establishing an individualized follow-up strategy according to their risk of progression. Moreover, the availability of novel, noncytotoxic drugs represents an attractive opportunity to investigate their efficacy in SMM (and even MGUS) patients with high risk of progression.

In summary, in the present study we show that multiparameter FC evaluation of BMPC at diagnosis of MGUS and SMM is a valuable tool for predicting the risk of progression to overt MM, because it allows the identification of patients at high risk of early progression (those in which the majority of PC display an aberrant phenotype), which may benefit from early treatment interventions, as opposed to a group of patients (in which both, aberrant and normal PC coexist) who will probably be free of progression for a long period of time and could be safely monitored without treatment over many years.

## Acknowledgments

This work was supported in part by a national grant from the Spanish Instituto de Salud Carlos III (Ref. PI50575 and FIS PI-02/0905) and Ministerio de Educación y Ciencia (Ref. SAF: 2004-06587).

We would like to thank M. Anderson and M. J. Rodrigo for their help with the English language.

## Authorship

Contribution: J.F.S.M. and A.O. conceived the idea and, together with M.-B.V., designed the study protocol; E.P.-P., M.-B.V., G.M., and C.L.-B. analyzed the flow cytometry data; E.P.-P. and M.-B.V were involved in the data analysis and, together with J.F.S.M., cowrote the paper; J.F.S.M. and A.O. reviewed and corrected the paper; R.G.-S. and M.-V.M. were responsible for databases, clinical cases, and follow-up of patients; and A.G.d.C., J.G., G.M.-N., J.M.A., N.d.l.H., J.M.H., and A.M. were responsible for clinical cases and follow-up of patients and contributed to the clinical data entry. E.P.-P. and M.-B.V. contributed equally to this work.

Conflict-of-interest disclosure: The authors declare no competing financial interests.

Correspondence: Jesús F. San Miguel, Department of Hematology, Hospital Universitario de Salamanca, Paseo de San Vicente 58-182, 37007 Salamanca, Spain; e-mail: sanmigiz@gugu.usal.es.

## References

1. Rajkumar SV. MGUS and smoldering multiple myeloma: update on pathogenesis, natural history, and management. Hematology (Am Soc Hematol Educ Program). 2005;340-345.
2. Criteria for the classification of monoclonal gammopathies, multiple myeloma and related disorders: a report of the International Myeloma Working Group. Br J Haematol. 2003;121:749-757.
3. Kyle RA, Rajkumar SV. Monoclonal gammopathy of undetermined significance. Br J Haematol. 2006;134:573-589.
4. Kyle RA, Therneau TM, Rajkumar SV, et al. A long-term study of prognosis in monoclonal gammopathy of undetermined significance. N Engl J Med. 2002;346:564-569.
5. Dimopoulos MA, Moulopoulos A, Smith T, Delasalle KB, Alexanian R. Risk of disease progression in asymptomatic multiple myeloma. Am J Med. 1993;94:57-61.
6. Wisløff F, Andersen P, Andersson TR, et al. Incidence and follow-up of asymptomatic multiple myeloma. The myeloma project of health region I in Norway. II. Eur J Haematol. 1991;47:338-341.
7. Kyle RA, Therneau TM, Rajkumar SV, et al. Prevalence of monoclonal gammopathy of undetermined significance. N Engl J Med 2006;354:1362-1369.
8. Weber DM, Dimopoulos MA, Moulopoulos LA, et al. Prognostic features of asymptomatic multiple myeloma. Br J Haematol. 1997;97:810-814.
9. Cesana C, Klersy C, Barbarano L, et al. Prognostic factors for malignant transformation in monoclonal gammopathy of undetermined significance and smoldering multiple myeloma. J Clin Oncol. 2002;20:1625-1634.
10. Dimopoulos MA, Moulopoulos LA, Maniatis A, Alexanian R. Solitary plasmacytoma of bone and asymptomatic multiple myeloma. Blood. 2000;96:2037-2044.
11. Rajkumar SV, Kyle RA, Therneau TM, et al. Serum free light chain ratio is an independent risk factor for progression in monoclonal gammopathy of undetermined significance. Blood. 2005;106:812-817.
12. Ocqueteau M, Orfao A, Almeida J, et al. Immunophenotypic characterization of plasma cells from monoclonal gammopathy of undetermined significance patients. Implications for the differential diagnosis between MGUS and multiple myeloma. Am J Pathol. 1998;152:1655-1665.
13. Sarasquete ME, Garcia-Sanz R, Gonzalez D, et al. Minimal residual disease monitoring in multiple myeloma: a comparison between allelic-specific oligonucleotide real-time quantitative polymerase chain reaction and flow cytometry. Haematologica. 2005;90:1365-1372.
14. Ocio EM, Mateo G, Vidriales B, et al. Cell cycle analysis of Waldenstrom's macroglobulinemia. Clin Lymphoma. 2005;5:250-252.
15. Orfao A, Ortuno F, de Santiago M, Lopez A, San Miguel J. Immunophenotyping of acute leukemias and myelodysplastic syndromes. Cytometry A. 2004;58:62-71.
16. Vidriales MB, San Miguel JF, Orfao A, Coustan-Smith E, Campana D. Minimal residual disease monitoring by flow cytometry. Best Pract Res Clin Haematol. 2003;16:599-612.
17. San-Miguel JF, Vidriales MB, Orfao A. Immunological evaluation of minimal residual disease (MRD) in acute myeloid leukaemia (AML). Best Pract Res Clin Haematol. 2002;15:105-118.
18. Harada H, Kawano MM, Huang N, et al. Phenotypic difference of normal plasma cells from mature myeloma cells. Blood. 1993;81:2658-2663.
19. Sezer O, Heider U, Zavrski I, Possinger K. Differentiation of monoclonal gammopathy of undetermined significance and multiple myeloma using

From www.bloodjournal.org by guest on November 22, 2016. For personal use only.



flow cytometric characteristics of plasma cells. Haematologica. 2001;86:837-843.

20. Ocqueteau M, Orfao A, Garcia-Sanz R, et al. Expression of the CD117 antigen (c-Kit) on normal and myelomatous plasma cells. Br J Haematol. 1996;95:489-493.
21. Davies FE, Rawstron AC, Owen RG, Morgan GJ. Minimal residual disease monitoring in multiple myeloma. Best Pract Res Clin Haematol. 2002; 15:197-222.
22. Almeida J, Orfao A, Ocqueteau M, et al. High-sensitive immunophenotyping and DNA ploidy studies for the investigation of minimal residual disease in multiple myeloma. Br J Haematol. 1999;107:121-131.
23. San Miguel JF, Almeida J, Mateo G, et al. Immunophenotypic evaluation of the plasma cell compartment in multiple myeloma: a tool for comparing the efficacy of different treatment strategies and predicting outcome. Blood. 2002;99:1853-1856.
24. Mateo Manzanera G, San Miguel Izquierdo JF, Orfao de Matos A. Immunophenotyping of plasma cells in multiple myeloma. Methods Mol Med. 2005;113:5-24.
25. Almeida J, Orfao A, Mateo G, et al. Immunophenotypic and DNA content characteristics of plasma cells in multiple myeloma and monoclonal gammopathy of undetermined significance. Pathol Biol (Paris). 1999;47:119-127.
26. Kyle RA. Monoclonal gammopathy of undetermined significance. Natural history in 241 cases. Am J Med. 1978;64:814-826.
27. Kyle RA, Greipp PR. Smoldering multiple myeloma. N Engl J Med. 1980;302:1347-1349.
28. Alexanian R. Localized and indolent myeloma. Blood. 1980;56:521-525.
29. Rosiñol L, Blade J, Esteve J, et al. Smoldering multiple myeloma: natural history and recognition of an evolving type. Br J Haematol. 2003;123: 631-636.
30. Greipp PR. Smoldering, asymptomatic stage 1, and indolent myeloma. Curr Treat Options Oncol. 2000;1:119-126.
31. Kyle RA. Monoclonal gammopathy of undetermined significance and smoldering multiple myeloma. Eur J Haematol Suppl. 1989;51:70-75.
32. Facon T, Menard JF, Michaux JL, et al. Prognostic factors in low tumour mass asymptomatic multiple myeloma: a report on 91 patients. The Groupe d'Etudes et de Recherche sur le Myelome (GERM). Am J Hematol. 1995;48:71-75.
33. Moulopoulos LA, Dimopoulos MA, Smith TL, et al. Prognostic significance of magnetic resonance imaging in patients with asymptomatic multiple myeloma. J Clin Oncol. 1995;13:251-256.
34. Leo R, Boeker M, Peest D, et al. Multiparameter analyses of normal and malignant human plasma cells: CD38++, CD56+, CD54+, cIg+ is the common phenotype of myeloma cells. Ann Hematol. 1992;64:132-139.
35. Dingli D, Nowakowski GS, Dispenzieri A, et al. Flow cytometric detection of circulating myeloma cells before transplantation in patients with multiple myeloma: a simple risk stratification system. Blood. 2006;107:3384-3388.

From www.bloodjournal.org by guest on November 22, 2016. For personal use only.

2007 110: 2586-2592
doi:10.1182/blood-2007-05-088443 originally published online June 18, 2007

# New criteria to identify risk of progression in monoclonal gammopathy of uncertain significance and smoldering multiple myeloma based on multiparameter flow cytometry analysis of bone marrow plasma cells

Ernesto Pérez-Persona, María-Belén Vidriales, Gema Mateo, Ramón García-Sanz, Maria-Victoria Mateos, Alfonso García de Coca, Josefina Galende, Guillermo Martín-Nuñez, José M. Alonso, Natalia de las Heras, José M. Hernández, Alejandro Martín, Consuelo López-Berges, Alberto Orfao and Jesús F. San Miguel

Updated information and services can be found at:
**http://www.bloodjournal.org/content/110/7/2586.full.html**

Articles on similar topics can be found in the following Blood collections
Clinical Trials and Observations (4425 articles)
Immunobiology (5426 articles)
Neoplasia (4182 articles)

Information about reproducing this article in parts or in its entirety may be found online at:
**http://www.bloodjournal.org/site/misc/rights.xhtml#repub_requests**

Information about ordering reprints may be found online at:
**http://www.bloodjournal.org/site/misc/rights.xhtml#reprints**

Information about subscriptions and ASH membership may be found online at:
**http://www.bloodjournal.org/site/subscriptions/index.xhtml**

Blood (print ISSN 0006-4971, online ISSN 1528-0020), is published weekly by the American Society of Hematology, 2021 L St, NW, Suite 900, Washington DC 20036.
Copyright 2011 by The American Society of Hematology; all rights reserved.
