# Supplementary material for: Expression of Concern: Prognostic value of circulating plasma cells in patients with multiple myeloma: A meta-analysis
Source: PLoS One. 2023 Feb 21;18(2):e0282230. doi: 10.1371/journal.pone.0282230 (PMC9942954; doi:10.1371/journal.pone.0282230)
Supplement: S1 File — (ZIP) [file pone.0282230.s001.zip › primary data/excluded research/2008 Idiotype vaccination in patients with myeloma reduced circulating myeloma cells (CMC)..pdf]

Annals of Oncology 19: 1172–1179, 2008
doi:10.1093/annonc/mdn017
Published online 13 February 2008

Idiotype vaccination in patients with myeloma reduced circulating myeloma cells (CMC)

A. O. Abdalla[1,3], P. Kokhaei[1,3,4], L. Hansson[1,2,3], H. Mellstedt[1,2,3]* & A. Österborg[1,2,3]

[1]Immune and Gene Therapy Laboratory, Cancer Centre Karolinska; [2]Departments of Hematology and Oncology, Karolinska University Hospital, Solna; [3]Department of Oncology/Pathology, Karolinska Institutet, Stockholm, Sweden; [4]Department of Immunology, Semnan Medical University, Semnan, Iran

Received 28 August 2007; revised 4 January 2008; accepted 7 January 2008

Background: Circulating myeloma cells (CMC), exhibiting the same immunoglobulin heavy-chain gene rearrangements as the plasma cells, are part of the myeloma clone. In this study, we evaluated the effect of idiotype (Id) vaccination on CMC.

Patients and methods: Eleven patients were immunized with the autologous Id in combinations with granulocyte–macrophage colony-stimulating factor and interleukin 12, and followed for CMC by quantitative real-time allele-specific PCR. Id-specific T cells were monitored by proliferation assay, enzyme-linked immunospot (interferon-γ) assay, and quantitative real-time PCR for cytokines. Regulatory T (T_{reg}) cells were analyzed by flow cytometry.

Results: CMC were detected in 9 of 11 patients at start of vaccination. In four patients, CMC declined and two had a complete molecular remission. Further two patients had stable levels of CMC during follow-up, while in three patients CMC progressively increased. Six patients had a vaccine-induced Id-specific T-cell response. A significant correlation was observed between reduced/stable levels of CMC and the Id-specific T cells ($P < 0.02$). The frequency of T_{reg} cells was decreased in immune responders, but increased in immune nonresponders ($P < 0.05$). No significant change in the serum M-protein concentration was, however, observed in any patient.

Conclusion: Id vaccination reduced CMC, which correlated with vaccine-induced Id-specific T cells. Further studies are warranted to analyze the clinical significance of CMC and clinical effects of Id vaccination.

Key words: circulating myeloma cells, idiotype vaccination, multiple myeloma

Downloaded from http://annonc.oxfordjournals.org/ by guest on October 19, 2016

introduction

The characteristics of the B cells belonging to the malignant clone in multiple myeloma (MM) are a matter of debate. An early B lymphocyte lineage cell with the ability to replicate and differentiate into the malignant myeloma plasma cell has been postulated as the precursor myeloma cell [1–3]. Precursor myeloma cells might be relatively resistant to chemotherapy and may be responsible for recurrence of the disease [4–6]. Those cells might be detected in the blood [2, 3, 7].

Clonal expansion of B lymphocytes during maturation is accompanied by rearrangement of genes encoding the variable (V) regions of the immunoglobulin (Ig) heavy (H) and light (L) chains forming the idiotypic structure harboring the antigenic determinants. In MM, the tumor cell clone encompasses a differentiating B-cell population which shares identical IgV_H and IgV_L gene rearrangements including terminally differentiated plasma cells expressing the idiotypic structures [2, 7].

The idiotypic structure is a unique target for immunotherapy [8] and has extensively been used in vaccine trials [9]. In MM, idiotype (Id) vaccination induced Id-specific T cells. Clinical effects measured as reduction of the serum M-protein concentration [9] and prolongation of time to progression (TtP) [10] have been noted. Id-reactive T cells may target Id-peptide/major histocompatibility complexes on myeloma cells and lyse the cells [11, 12]. Circulating myeloma cells (CMC) expressing the Id might be a target for the specific T cells.

A previous pilot study from our group showed that CMC might be reduced by Id vaccination [13]. In the present study, those preliminary observations are extended and related to the induction of Id-specific T cells and regulatory T (T_{reg}) cells. Our data show that vaccine-induced reduction of CMC correlated significantly to vaccine-induced Id-specific T cells.

material and methods

patients

Eleven patients with stage I–II IgG MM (Table 1) were included. The median age was 70 years (range 51–87 years). All patients were immunized with the autologous Id protein together with granulocyte–macrophage colony-stimulating factor (GM-CSF) alone, interleukin (IL)-12 alone, or a combination of GM-CSF and IL-12 as adjuvant cytokines [10, 14]. The median time for maintained Id vaccination was 46 weeks (range 4–126 weeks). The studies were approved by the Medical Product Agency and the local ethics committee. Four of the 11 patients were previously analyzed for

*Correspondence to: Prof H. Mellstedt, Department of Oncology, Karolinska University Hospital, Solna, SE-171 76 Stockholm, Sweden. Tel: +46-8-51774308; Fax: +46-8-318327; E-mail: hakan.mellstedt@karolinska.se

© The Author 2008. Published by Oxford University Press on behalf of the European Society for Medical Oncology. All rights reserved. For Permissions, please email: journals.permissions@oxfordjournals.org

Table 1. Clinical characteristics of the myeloma patients vaccinated with the autologous idiotype in combination with adjuvant cytokines

Patient no.	Gender	Age	Clinical stage	Treatment
1	M	51	IA	Untreated
2	F	69	IIA	Treated[a]
3	F	85	IA	Untreated
4	F	69	IA	Untreated
5	M	60	IA	Untreated
6	F	77	IIA	Untreated
7	M	85	IA	Untreated
8	F	88	IIA	Untreated
9	M	67	IA	Untreated
10	M	87	IA	Untreated
11	F	70	IA	Untreated

[a]CIB (cyclophosphamide, interferon-α, betamethasone) ended 3 years before sampling.
F, female; M, male.

CMC [13] but reevaluated by the modified real-time allele-specific oligonucleotide (ASO) PCR technique as described below and followed for a longer time.

immunization protocol and immune testing times

The individual Id vaccines were prepared as described [10, 14]. Five patients (no. 1, 2, 4, 7, and 10) received 0.5 mg of the autologous Id vaccine intracutaneously together with 2 μg IL-12 (Genetics Institute, Inc./Wyeth-Ayerst Research, Cambridge, MA) subcutaneously [10]. Five patients (no. 5, 6, 8, 9, and 11) were given the Id vaccine and IL-12 as described above plus 75 μg of GM-CSF (Schering-Plough, Kenilworth, NJ) daily at the vaccine site, days 1 to 4 [10]. The vaccination procedure was repeated after 2, 4, 6, 8, and 14 weeks (induction phase) [14] and continued at weeks 30, 46, 62, 78, 94, and 110 (maintenance phase). One patient (no. 3) received the Id vaccine plus 75 μg of GM-CSF as above but only repeated after 2, 4, 6, 8, and 14 weeks (induction phase). Patients were tested for cellular immune responses before vaccination and at weeks 4, 8, 10, 14, 16, 30, 32, 46, 48, 62, 64, 78, 80, 94, 96, 110, 112, 126, 142, and 158 or until progressive disease.

isolation of peripheral blood mononuclear cells and bone marrow mononuclear cells

Peripheral blood mononuclear cells (PBMC) and bone marrow mononuclear cells (BMMC) were isolated using Ficoll/Hypaque (Amersham-Bioscience, Uppsala, Sweden) density-gradient centrifugation as described [10].

preparation of monoclonal IgG and F(ab′)$_2$ fragments for *in vitro* tests

The procedure has been described in detail [14]. Briefly, serum was applied to a sterile mabTrapG column (Pharmacia, Uppsala, Sweden). Isoelectric focusing (Pharmacia Phast system) showed that >90% of the IgG was monoclonal. The monoclonal IgG was dialyzed against sterile NaCl and filtered through a Millipore filter (0.20 mm). F(ab′)$_2$ fragments were prepared by pepsin digestion. F(ab′)$_2$ fragments of the monoclonal IgG from other myeloma patients were used as controls.

proliferation assay

The method has been described in detail [14]. Briefly, PBMC were stimulated with purified F(ab′)$_2$ fragments of the idiotypic IgG and allogeneic monoclonal isotype-matched IgG F(ab′)$_2$ fragments as controls. Unstimulated cells and cells stimulated with purified protein derivate (PPD) (Statens Seruminstitut, Copenhagen, Denmark) and phytohemaglutinin (PHA; Sigma-Aldrich, Stockholm, Sweden) were used as controls. Cells were cultured for 6 days and [^{3}H]-thymidine (Amersham, Life Sciences, Amersham, UK) was added during the last 18 h of culture. Tests were run in triplicates. Mean thymidine incorporation was calculated for each triplicate. Stimulation index (SI) was calculated by dividing mean thymidine incorporation of antigen-stimulated cells with that of unstimulated cells. Mean + 3SD of SI induced by isotypic monoclonal IgGs and polyclonal IgG (n = 2183 experiments) was 2.78. An SI cut-off level of ≥3.00 was used to indicate the presence of an Id-specific cellular response [10].

enzyme-linked immunospot assay

The enzyme-linked immunospot (ELISPOT) assay for identification of interferon-γ (IFN-γ)-secreting cells was carried out as described [10]. Briefly, PBMC were incubated with F(ab′)$_2$ fragments of the idiotypic or the isotypic (control) monoclonal IgG for 48 h in humidified air with 5% CO_2 at 37°C. PBMC were also stimulated with medium alone, PPD, and PHA. Spots-forming units (SFU) corresponding to cells secreting IFN-γ were quantified using an automated computer-assisted video imaging analysis system (Axioplan2) (Carl Zeiss Vision, Jena, Germany). The number of SFU was obtained by subtracting the number of spots in cultures incubated with medium alone from that of stimulated cultures. Results are expressed as number of SFU/10^6 PBMC. Mean + 3SD of SFU/10^6 PBMC induced by the isotype-matched IgG F(ab′)$_2$ (control) (n = 513 experiments) was 60. A cut-off level of ≥70 SFU/10^6 PBMC was used to indicate the presence of an Id-specific cellular response [10].

total RNA extraction and first-strand complementary DNA synthesis

Total RNA was extracted from PBMC by the guanidium thiocyanate–phenol–chloroform extraction technique using RNAzol B (AMS Biotechnology Europe, Stockholm, Sweden). First-strand complementary DNA (cDNA) synthesis was carried out according to standard protocols [15] using the First-Strand cDNA Synthesis Kit (Amersham-Pharmacia Biotech, Uppsala, Sweden).

genomic DNA preparation

Genomic DNA was extracted from PBMC using the QRAprep Spin Miniprep Kit (Qiagen®, Helden, Germany).

quantitative real-time PCR for cytokines and cytotoxic proteins

Details of the method have been described [15]. Briefly, PBMC were incubated with F(ab′)$_2$ fragments of the idiotypic- or the isotype-matched (control) monoclonal IgG for 8 and 48 h. Cells were also incubated with medium alone and PHA as controls. Cells incubated for 8 h were analyzed for the expression of the cytokine genes IFN-γ, tumor necrosis factor-α (TNF-α), IL-4, and IL-5, and those stimulated for 48 h were analyzed for the expression of granzyme B and perforin. $MgCl_2$ concentration was optimized for the individual primers to obtain maximum efficiency. The efficiencies of probes and primers used in the assay were in the range of 90%–100% corresponding to slopes between 3.6 and 3.1. An Id-specific cytokine response was considered when the relative fold increase of a gene induced by the autologous Id was higher than the isotypic control, i.e. a fold increase ratio >1.

criteria for a vaccine-induced Id-specific T-cell response

A vaccine-induced Id-specific T-cell response was considered to be present when all the following three criteria were met: (i) an Id-specific SI, SFU, or messenger RNA (mRNA) cytokine gene expression ratio above the

Downloaded from http://annonc.oxfordjournals.org/ by guest on October 19, 2016

corresponding cut-off levels (3, 70, and >1, respectively); (ii) an Id-specific SI, SFU, or mRNA cytokine expression ratio more than twice the respective pre-vaccination baseline value; and (iii) an Id-specific cellular response in at least two of the three analyses detected at at least two different testing times after start of vaccination [10].

The cellular immune response was defined as a Th_1 response when only Th_1 cytokine genes (IFN-γ and/or TNF-α) were expressed and as a Th_2 response when only the Th_2 cytokine genes (IL-4 and/or IL-5) were noted. When both types of cytokine genes were found, the immune response was considered a mixed Th_1/Th_2 with a predominance of Th_1 cells when Th_1 cytokine genes' fold increase ratios were higher or expressed at more testing times than the Th_2 cytokine genes. The immune response was considered a mixed Th_1/Th_2 response with a predominance of Th_2 cells when the Th_2 cytokine genes' fold increase ratios were higher or expressed at more testing times than Th_1 cytokine genes [10, 16].

ASO probes and primers

The gene encoding the variable heavy chain (V_H) of each patient was determined by reverse transcription (RT)–PCR using consensus V_H family primers [17] and a consensus gamma constant Cγ region primer [18] using BMMC. The dominant V_H gene family (tumor clone) was identified as the clone amplified at the lowest dilution. The V_H PCR products were visualized on 1.5% ethidium-stained agarose gel and the V_H amplicons representing the myeloma clone were excised from the gel and purified with a QIAquick Gel Extraction Kit© (Qiagen®). The purified V_H amplicons were ligated into a pGEM-T Easy Vector© (V_H plasmid) and then transformed into competent *E. coli* (JM 109©, Promega®, Madison, WI) and cultivated on Luria-Bertani medium–Ampicillin agar plates (100 g/ml) pretreated with isopropyl-beta-D-thiogalactopyranoside and X-Gal. Five V_H plasmid-positive colonies were recultivated over night and plasmids were extracted with QIAprep Spin Miniprep kit© (Qiagen®), and sequenced using ABI PRISM 310© (Applied Biosystem®, Stockholm, Sweden) with T7 and Sp6 primers and Big Dye© reagent. The sequences were confirmed by aligning to the closest published germline genes using the V-BASE directory (Medical Research Council, Centre for Protein Engineering, UK), and the IgBlast and Blast (National Center for Biotechnology Information, Bethesda, MD) databases [19]. The β-actingene was also cloned and sequenced.

The complementary-determining regions (CDR) II and III sequences were identified for all patients. For the cDNA assay, patient-specific ASO sense primers corresponding to the CDRIII regions were designed for each patient to obtain maximum specificity (Table 2). A Cγ antisense primer (GGAAGTAGTCCTTGACCA) and probe (CCTCCACCAAGGGCCCATCG) were used for all patients. For the genomic DNA assay, a patient-specific sense primer corresponding to the V_H 3 or 1 region, ASO (CDRIII) antisense primer, and ASO (CDRII) probe were used (Table 2). The V_H 3/1, CDRIII, and CDRII primers were tested on a panel of DNA from different patients to ensure that the primer amplified only the V_H gene from the patient of interest.

RT-ASO-PCR and real-time ASO-PCR

Patients were first tested for the presence of the malignant clone by RT-ASO-PCR using patient-specific ASO primers. All positive patients were then subjected to quantitative real-time ASO-PCR analyses as described [20] with minor modifications. Briefly, PCR was carried out in a 25-μl reaction volume containing 1× TaqMan Buffer A (Perkin Elmer), $MgCl_2$ (optimized for each ASO assay), and 3 mM $MgCl_2$ for the β-actin assay (Perkin Elmer), 0.5 mM dNTPs (deoxyribonucleotide triphosphates) (Perkin Elmer, Stockholm, Sweden), 5.0 pmol forward (CDR3) ASO primer (Cybergene AB, Stockholm, Sweden), 5.0 pmol reverse (Cγ) primer (Cybergene AB), 2.5 pmol (Cγ) *Taq*Man probe (Cybergene AB), 0.5 units Ampli-Taq Gold, and 0.1 units uracil-*N*-glycosylase (Perkin Elmer). Two microliters of cDNA were included in each reaction volume except for the negative controls. In two patients, the test was additionally run on genomic DNA for comparison. For generation of standard curves, serial dilutions of known concentrations of the patient-specific V_H and β-actin plasmid DNA were used. For each sample, at least four or six aliquots (when material allowed) were used, two to three aliquots for quantitation of myeloma B cells and three aliquots for β-actin. Aliquots were amplified for an initial period of 2 min at 50°C and 10 min at 95°C followed by 40 concurrent cycles involving denaturation at 95°C for 15 s and annealing/extension at 60°C for 1 min. ABI PRISM 7700 Sequence Detection System (Perkin Elmer) was used for online quantification.

The mean gene copy number of the patient-specific V_H was divided by that of the corresponding β-actin copy number for normalization. The value of each sample before vaccination was set to 100%. The values during

Table 2. Allele-specific oligonucleotide (ASO) primers and probes

Patient no.	ASO CDRIII primer (sense) (cDNA assay)	V_H 3/1 families, ASO CDRIII and CDRII primers and probes (genomic DNA assay)
1	GAGAGCCAAGGACCACAGTAAC	ND
2	ACGAGAGCGGTCGTCATTATG	ND
3	ACAGCCTATAGCATTCGTTGCT	ND
4	CATAGACGTCTGGGGCGA	ND
5	AATAGGACGGAGTGGGGTTG	GGTTACACCTTTACCGACTATGGT (sense)
		CAACCCCACTCCGTCCTATTCTCGC (antisense)
		ATCAGCGGTTACAATGGTAACACAATCTATGCA (probe)
6	CGCCCCTCCCGTATATAACA	GGGGGTCCCTGAGACTCTCCTGT (sense)
		ACTGTTATATACGGGAGGGGCG (antisense)
		CCGATTCACCATCTCCAGAGACAAC (probe)
7	ATAGTAGTGGAGGCTATGGCTCC	ND
8	GTCCTCTCGGGGCACTG	ND
9	GCAAAAGGGCCCTCAGTCTA	ND
10	GGGTTGGGACTACTGGGG	ND
11	GAGTGGGAGTTGCCGACATACT	ND

cDNA, complementary DNA; CDR, complementary-determining regions; ND = not done.

Downloaded from http://annonc.oxfordjournals.org/ by guest on October 19, 2016

follow-up were presented as percent of the pre-vaccination value. When genomic DNA was analyzed, the absolute gene copy number was plotted against testing times and compared with the corresponding gene copy number in the cDNA real-time ASO-PCR assay.

T_{reg} cells

T_{reg} cells were analyzed using the Human Regulatory T cell Staining Kit (eBiosciences, Inc., San Diego, CA). Frozen PBMC were thawed and subjected to Ficoll gradient centrifugation to obtain viable cells. Cell viability was >98%. Cell surface markers were analyzed by fluorochrome-conjugated monoclonal antibodies against CD4 and CD25. FoxP3 was assessed intracellularly by anti-FoxP3 monoclonal antibody. Cells incubated with irrelevant isotype-matched antibodies were used as controls. For each patient, a minimum of 10^5 $CD4^+$ cells were acquired. The $CD4^+$/$CD25^{high}$/$Foxp3^+$ subset was analyzed using FACSCalibur (BD, Mountain View, CA). The data were analyzed by the Flowjo® Software program (Tree Star, OR) and presented as the frequency of triple positive ($CD4^+$/$CD25^{high}$/$Foxp3^+$) cells within the CD4 population.

statistical methods

Fisher's exact test was used to compare the kinetics of CMC in immune responders and immune nonresponders. The chi-square test was amplified to check correlation between CMC and changes in T_{reg} cells. The Mann–Whitney U test was used to compare the changes in T_{reg} cells in immune responders and nonresponders.

results

vaccine-induced Id-specific T cells

The Id-specific T-cell responses are shown in Table 3. Six patients (no. 3, 4, 5, 6, 7, and 8) mounted a vaccine-induced Id-specific T-cell response with a predominance of Th_1 cells. Five of the six immune responders had Id-induced expression of the genes for the cytotoxic proteins granzyme B and/or perforin. The remaining five patients did not meet the criteria for mounting vaccine-induced T cells. No patients had a pre-vaccination Id-specific T-cell immunity (data not shown).

effect of Id vaccination on CMC

A representative experiment showing the patient-specific IgV_H cell clone by RT-ASO-PCR during follow-up is depicted in Figure 1 (patient no. 3). The patient showed complete molecular remission of CMC lasting for >1 year.

All RT-ASO-PCR-positive patients were subjected to quantitative real-time ASO-PCR to determine the kinetics of CMC. Patients who mounted a vaccine-induced Id-specific T-cell response (n = 6) either showed a reduction of CMC over time (no. 3, 4, 5, and 6) (Figure 2A) or maintained a similar level of CMC (no. 7 and 8) (Figure 2B). Two of the patients (no. 3 and 4) achieved a complete molecular

Downloaded from http://annonc.oxfordjournals.org/ by guest on October 19, 2016

Table 3. Vaccine-induced idiotype-specific T-cell responses and relation to circulating myeloma cells (CMC) responses

Patient no.	Adjuvant cytokine(s)	Proliferation assay (SI)	ELISPOT (IFN-γ) (SFU/10^6 cells)	QRT-PCR for multiple cytokines (fold increase ratio)	Overall vaccine-induced T-cell response	Overall response of CMC during follow-up
1	IL-12	—	—	IFN-γ: 32 (30)[a]	[b]	—[c]
2	IL-12	—	—	IL-5: 128 (16)	—	—[c]
3	GM-CSF	3.4 (16), 3.2 (30), 16.3 (48)	90 (30), 320 (62)	TNF-α: 44415 (16), 4 (30); GRZMB: 2 (8), 23 (10)	(Th_1)[d]	Complete remission
4	IL-12	—	3850 (48)	TNF-α: 14972 (14); GRZMB: 4.7 (14)	(Th_1)[d]	Complete remission
5	IL-12/GM-CSF	3.6 (4), 4.4 (14)	—	IFN-γ: 1820 (8); TNF-α: 13308 (8); IL-5: 5 (4) 6580 (8), 3 (16); perforin: 50 (30)	(Mixed with a Th_1 predominance)[d]	Decline
6	IL-12/GM-CSF	—	110 (30)	IL-5: 10 (32); GRZMB: 5 (8), 4 (10); perforin: 3 (10), 5 (14)	(Mixed with a Th_2 predominance)[d]	Decline
7	IL-12	7.5 (4), 179 (8), 24.2 (10), 63.9 (14)	120 (8), 780 (10), 110 (14), 100 (96)	IL-5: 16 (4); IFN-γ: 9 (14); TNF-α: 18 (14); GRZMB: 1.5 (16), 1.5 (30)	(Mixed with a Th_1 predominance)[d]	Stable
8	IL-12/GM-CSF	90.5 (4), 43.5 (8), 40 (10), 35.3 (14)	—	IL-4: 80684 (16)	(Th_2)[d]	Stable
9	IL-12/GM-CSF	—	—	ND	—	Increase
10	IL-12	—	—	TNF-α: 4 (8)	—	Increase
11	IL-12/GM-CSF	—	—	—	—	Increase

[a]Figure within bracket indicates week for the response. Only positive responses are shown.
[b]No T-cell response.
[c]No detectable CMC at any time.
[d]A T-cell response according to criteria (see 'material and methods').
ELISPOT, enzyme-linked immunospot; GM-CSF, granulocyte–macrophage colony-stimulating factor; IFN-γ; interferon-γ; IL, interleukin; GRZMB, granzyme B; SI, stimulation index; SFU, spots-forming units; QRT-PCR, quantitative real-time PCR; TNF-α, tumor necrosis factor-α.

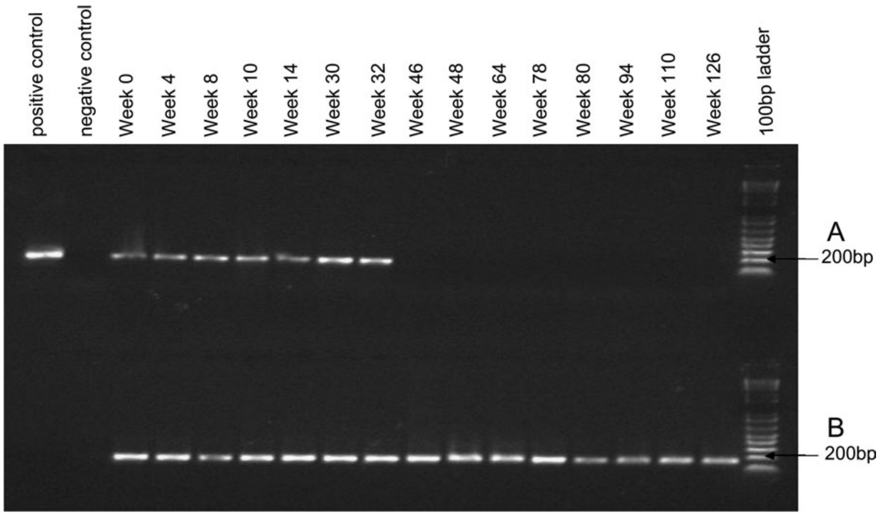

Figure 1. A representative experiment of patient-specific V_H reverse transcription allele-specific oligonucleotide PCR (35 cycles) showing (A) the patient-specific PCR amplicons during vaccination (weeks 0–110) and follow-up (weeks 110–126) and (B) the corresponding β-actin PCR amplicons. Positive control, patient-specific V_H plasmid DNA; negative control, PCR mix containing no V_H DNA.

Downloaded from http://annonc.oxfordjournals.org/ by guest on October 19, 2016

remission in blood and remained negative for CMC during long-term follow-up. Patients who did not mount an Id-specific T-cell response (no. 9, 10, and 11) showed a progressive increase in CMC (Figure 2C). (Patients 1 and 2 had no detectable CMC at any time.) The reduction of CMC in patients with an Id-specific T-cell response was statistically significant compared with the increase in CMC seen in patients who failed to mount a vaccine-induced Id-specific T-cell response ($P < 0.02$). It should, however, be noted that no significant change in the serum M-protein concentration was observed in any patient.

The kinetics of CMC were similar, when CMC were analyzed by genomic DNA or cDNA, as exemplified in Figure 3A and B (patient no. 6). However, the gene copy numbers in the cDNA assay were much higher than the corresponding numbers in the genomic DNA assay, which is to be expected as a single-gene activation may yield multiple mRNA transcripts.

T_{reg} cells

Due to lack of material, T_{reg} cells were only analyzed before and at a median time of 4.5 weeks (range 2–19 weeks) after the last Id immunization in six patients (no. 4, 5, 6, 9, 10, and 11). Three patients (no. 4, 5, and 6) were immune responders and three (no. 9, 10, and 11) immune nonresponders. The frequencies (%) of T_{reg} cells before and after immunization in immune responders were 4.6, 6.1, and 6.2 and 2.4, 3.7, and 3.8, respectively, while in immune nonresponders the corresponding figures were 8.2, 0.75, and 4.5 and 7.3, 2.7, and 5.7, respectively. The change in the frequency of T_{reg} cells comparing immune responders and immune nonresponders was statistically significant ($P < 0.05$) (Figure 4). Three of the four patients with a reduction of T_{reg} cells were immune responders and had a reduction of CMC, while the two patients with an increase in T_{reg} cells were immune nonresponders and had an increase in CMC.

discussion

Circulating tumor cells have been described in several tumor types. The primary tumor lesion might shed tumor cells into the circulation even at an early stage [21–23]. The number of blood tumor cells correlated with progression-free and overall survival in patients with metastatic breast cancer [24, 25]. In patients with high-risk uveal melanoma, the presence of circulating tumor cells was an independent prognostic factor for development of distant metastases [26].

In myeloma, the presence of CMC at the time of diagnosis was associated with aggressive disease and a poor prognosis [27]. CMC in the stem-cell harvest for autologous stem-cell transplantation was an independent prognostic factor predicting early relapse regardless of the initial response to treatment [22].

In spite of the initial high response rates achieved by current anti-myeloma therapies, myeloma remains an incurable disease. Thus, there is a need for complementary therapies to overcome the inevitable disease recurrence. Id-induced immunity was found to be associated with a significant increase in disease-free survival of patients with follicular lymphoma [28] and molecular remission in blood by eliminating circulating lymphoma cells [29]. Id vaccination in myeloma patients has been shown to induce a tumor-specific T response and prolonged TtP [9, 10].

Myeloma patients with early-stage disease or minimal residual disease after treatment most likely have preserved T-cell functions as compared with patients with advanced disease [30]. Such patients may be suitable candidates for immunotherapy. We have previously reported, in a small pilot study [13], that in early-stage myeloma, CMC were reduced following Id vaccination. In the present study, where all patients had stage I or asymptomatic stage II disease, we substantiated those findings. Seven of the patients (64%) had Id-specific T cells before vaccination (data not shown), which are known to be more frequently found in myeloma patients

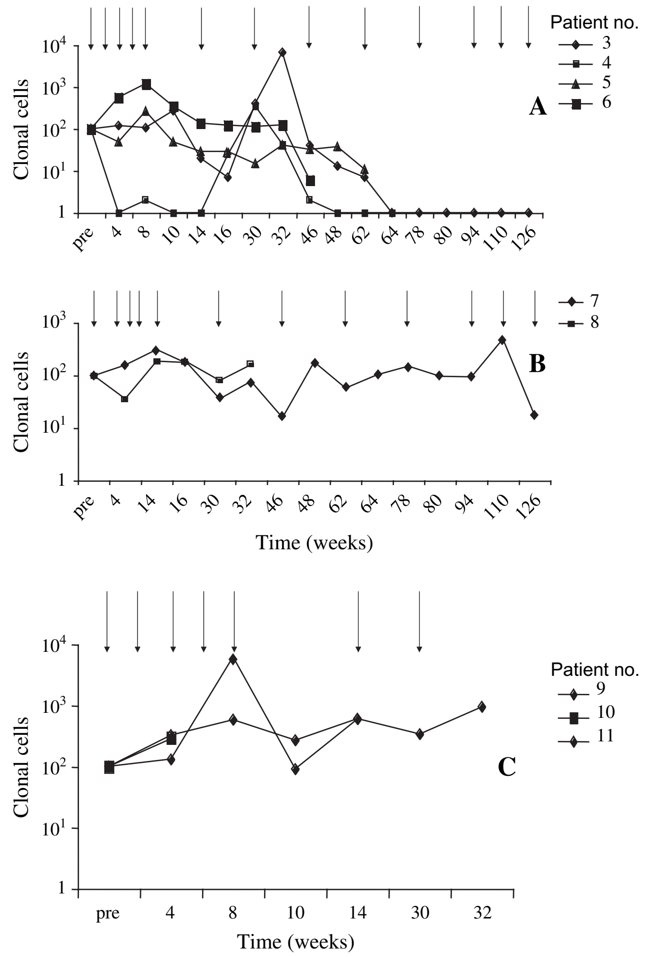

Figure 2. Kinetics of circulating myeloma cells (real-time allele-specific oligonucleotide PCR) in (A) patients (no. 3, 4, 5, and 6) who mounted an idiotype-specific T-cell response during vaccination or (B) patients (no. 7 and 8) with stable levels and (C) in patients (no. 9, 10, and 11) who did not show a vaccine-induced idiotype T-cell response. Baseline for each patient was set to 100%. The values during follow-up are given as percent of the initial value. Arrows indicate time of idiotype vaccination.

with a low tumor burden [31]. The presence of spontaneously induced tumor-specific T cells is considered a favorable sign for response to vaccine treatment as it might be easier to boost than to induce an immune response against an auto-antigen [9]. Six of the 11 patients (55%) mounted or boosted an Id-specific T-cell response during vaccination and all of them showed a reduction and/or stable level of CMC. A blood complete molecular remission was observed in two patients, which lasted for 4 and 14 months, respectively. The decline in CMC was noted quite late and after multiple vaccinations indicating the importance of repeated immunizations. Since no treatment other than the Id vaccine was given, the observed clinical effect is likely to be vaccine induced. No significant change in the serum myeloma protein was, however, noted. The serum M-component reflects the myeloma tumor mass and is secreted mainly by the myeloma plasma cell population. CMC is regarded a spill over of myeloma bone marrow cells including the precursor B lymphoid myeloma cells, which are relatively resistant to chemotherapy and may be responsible for recurrence of the disease [4–6]. The presence of CMC in peripheral blood is an unfavorable prognostic sign [27]. Elimination of CMC might be of therapeutic benefit. The lack of correlation between the levels of CMC and M-component concentration might be explained by that CMC is not the major Ig-producing cell fraction and these cells are a minor fraction of the total myeloma population in patients with a serum M-component. Elimination of CMC might thus not induce a detectable reduction of the M-protein in patients with overt disease. No plasma cells evaluated by morphology were found in peripheral blood (data not shown). Although real-time ASO-PCR analysis using genomic DNA reveals the actual number of CMC, the number of CMC might be too few to be detected [13]. A real-time ASO-PCR assay on the basis of cDNA is more likely to detect V_H gene copy numbers of a very small CMC population. Therefore, we carried out real-time ASO-PCR analysis on cDNA to reduce the risk of false-negative results that might occur using genomic DNA. This sensitive technique may also explain why we noticed a reduction in CMC but not in the

Downloaded from http://annonc.oxfordjournals.org/ by guest on October 19, 2016

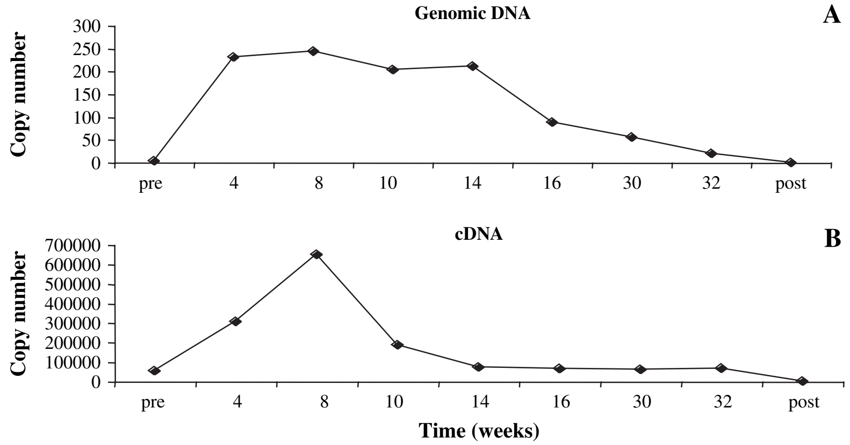

Figure 3. Representative experiment comparing the kinetics of circulating myeloma cells by (A) genomic DNA and (B) complementary DNA (patient no. 6).

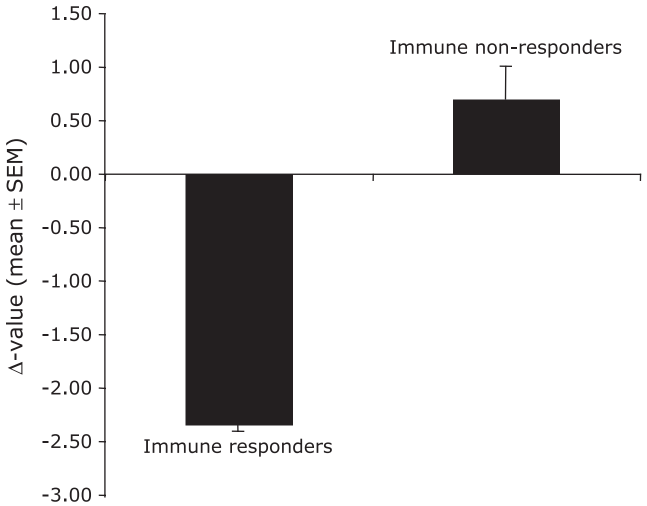

Figure 4. Changes in absolute percent values (delta Δ) of regulatory T (T_{reg}) cells at a median time of 4.5 weeks after the last idiotype immunization compared with baseline in immune responders ($n = 3$) and immune nonresponders ($n = 3$). Negative values indicate a decrease in T_{reg} cells and positive values an increase. The difference comparing immune responders and immune nonresponders was statistically significant ($P < 0.05$).

M-component concentration, i.e. vaccine therapy might only be able to eliminate a small tumor cell population but not a large to bring about reduction of the M-component. A Cγ antisense primer and probe were used to avoid the amplification of any contaminating genomic DNA. The two assay systems were shown to yield similar results with regard to the kinetics of CMC.

Another interesting preliminary observation was the reduction of T_{reg} cells in immune responders and increase in immune nonresponders. There are ample indications in both animals and cancer patients that an increase in both natural and induced T_{reg} cells is associated with decreased tumor-specific immunity and disease progression. Reduced T_{reg} levels seem to be a requirement for an effective tumor immunity and disease regression [32].

The results of the present study confirm and extend our previous observation, indicating that long-term Id vaccination in MM patients might reduce/eliminate CMC. Similar to the results in follicular lymphoma, vaccine-induced anti-Id immunity may be associated with a long-lasting molecular remission in blood. Further studies are warranted to establish the role of CMC in the biology of MM and in particular if analyzing such cells might be a valuable surrogate readout system for the evaluation of novel therapeutic approaches.

funding

The Cancer and Allergy Foundation; the Swedish Cancer Society; the Cancer Society in Stockholm; King Gustav V Jubilee Fund; the Torsten and Ragnar Söderberg's Foundation; the Stockholm County Council and Karolinska Institutet Foundations; the International Myeloma Foundation; the Multiple Myeloma Research Foundation.

acknowledgements

We are grateful to Ms Leila Relander and Ms Monica Liss for excellent secretarial help.

references

1. Corradini P, Boccadoro M, Voena C et al. Evidence for a bone marrow B cell transcribing malignant plasma cell VDJ joined to C mu sequence in immunoglobulin (IgG)- and IgA-secreting multiple myelomas. J Exp Med 1993; 178: 1091–1096.
2. Cremer FW, Goldschmidt H, Moos M. Clonotypic B cells in the peripheral blood of patients with multiple myeloma. Blood 2001; 97: 2913–2914.
3. Mellstedt H, Holm G, Björkholm M. Multiple myeloma, Waldenström's macroglobulinemia and benign monoclonal gammopathy. Characteristics of the B cell clone, immunoregulatory cell populations and clinical implications. Adv Cancer Res 1984; 41: 257–289.
4. Pilarski LM, Belch AR. Circulating monoclonal B cells expressing P glycoprotein may be a reservoir of multidrug-resistant disease in multiple myeloma. Blood 1994; 83: 724–736.
5. Pilarski LM, Cass CE, Tsuro T et al. Multidrug resistance of a continuously differentiating monoclonal B lineage in the blood and bone marrow of patients with multiple myeloma. Curr Top Microbiol Immunol 1992; 182: 177–185.
6. Rasmussen T, Jensen L, Johnsen HE. The CD19 compartment in myeloma includes a population of clonal cells persistent after high-dose treatment. Leuk Lymphoma 2002; 43: 1075–1077.
7. Bergsagel PL, Masellis Smith A, Belch AR et al. The blood B-cells and bone marrow plasma cells in patients with multiple myeloma share identical IgH rearrangements. Curr Top Microbiol Immunol 1995; 194: 17–24.
8. Bogen B, Ruffini PA, Corthay A et al. Idiotype-specific immunotherapy in multiple myeloma: suggestions for future directions of research. Haematologica 2006; 91: 941–948.
9. Choudhury A, Mosolits S, Kokhaei P et al. Clinical results of vaccine therapy for cancer: learning from history for improving the future. Adv Cancer Res 2006; 95: 147–202.
10. Hansson L, Abdalla AO, Moshfegh A et al. Long-term idiotype vaccination combined with interleukin-12 (IL-12), or IL-12 and granulocyte macrophage colony-stimulating factor, in early-stage multiple myeloma patients. Clin Cancer Res 2007; 13: 1503–1510.
11. Li Y, Bendandi M, Deng Y et al. Tumor-specific recognition of human myeloma cells by idiotype-induced CD8(+) T cells. Blood 2000; 96: 2828–2833.
12. Wen YJ, Barlogie B, Yi Q. Idiotype-specific cytotoxic T lymphocytes in multiple myeloma: evidence for their capacity to lyse autologous primary tumor cells. Blood 2001; 97: 1750–1755.
13. Rasmussen T, Hansson L, Osterborg A et al. Idiotype vaccination in multiple myeloma induced a reduction of circulating clonal tumor B cells. Blood 2003; 101: 4607–4610.
14. Osterborg A, Yi Q, Henriksson L et al. Idiotype immunization combined with granulocyte-macrophage colony-stimulating factor in myeloma patients induced type I, major histocompatibility complex-restricted, CD8- and CD4-specific T-cell responses. Blood 1998; 91: 2459–2466.
15. Abdalla AO, Kiaii S, Hansson L et al. Kinetics of cytokine gene expression in human CD4+ and CD8+ T-lymphocyte subsets using quantitative real-time PCR. Scand J Immunol 2003; 58: 601–606.
16. Abdalla AO, Hansson L, Eriksson I et al. Idiotype protein vaccination in combination with adjuvant cytokines in patients with multiple myeloma—evaluation of T-cell responses by different read-out systems. Haematologica 2007; 92: 110–114.
17. Rasmussen T, Kastrup J, Knudsen LM et al. High numbers of clonal CD19+ cells in the peripheral blood of a patient with multiple myeloma. Br J Haematol 1999; 105: 265–267.
18. Bakkus MH, Van Riet I, Van Camp B et al. Evidence that the clonogenic cell in multiple myeloma originates from a pre-switched but somatically mutated B cell. Br J Haematol 1994; 87: 68–74.
19. Lefranc MP, Giudicelli V, Ginestoux C et al. IMGT, the international ImMunoGeneTics database. Nucleic Acids Res 1999; 27: 209–212.

Downloaded from http://annonc.oxfordjournals.org/ by guest on October 19, 2016

20. Rasmussen T, Poulsen TS, Honore L et al. Quantitation of minimal residual disease in multiple myeloma using an allele-specific real-time PCR assay. Exp Hematol 2000; 28: 1039–1045.
21. Cohen SJ, Alpaugh RK, Gross S et al. Isolation and characterization of circulating tumor cells in patients with metastatic colorectal cancer. Clin Colorectal Cancer 2006; 6: 125–132.
22. Dingli D, Nowakowski GS, Dispenzieri A et al. Flow cytometric detection of circulating myeloma cells before transplantation in patients with multiple myeloma: a simple risk stratification system. Blood 2006; 107: 3384–3388.
23. Ikoma D, Ichikawa D, Ueda Y et al. Circulating tumor cells and aberrant methylation as tumor markers in patients with esophageal cancer. Anticancer Res 2007; 27: 535–539.
24. Budd GT, Cristofanilli M, Ellis MJ et al. Circulating tumor cells versus imaging—predicting overall survival in metastatic breast cancer. Clin Cancer Res 2006; 12: 6403–6409.
25. Hayes DF, Cristofanilli M, Budd GT et al. Circulating tumor cells at each follow-up time point during therapy of metastatic breast cancer patients predict progression-free and overall survival. Clin Cancer Res 2006; 12: 4218–4224.
26. Schuster R, Bechrakis NE, Stroux A et al. Circulating tumor cells as prognostic factor for distant metastases and survival in patients with primary uveal melanoma. Clin Cancer Res 2007; 13: 1171–1178.
27. Osterborg A, Nilsson B, Bjorkholm M et al. Blood clonal B cell excess at diagnosis in multiple myeloma: relation to prognosis. Eur J Haematol 1987; 38: 173–178.
28. Inoges S, Rodriguez-Calvillo M, Zabalegui N et al. Clinical benefit associated with idiotypic vaccination in patients with follicular lymphoma. J Natl Cancer Inst 2006; 98: 1292–1301.
29. Bendandi M, Gocke CD, Kobrin CB et al. Complete molecular remissions induced by patient-specific vaccination plus granulocyte-monocyte colony-stimulating factor against lymphoma. Nat Med 1999; 5: 1171–1177.
30. Mozaffari F, Hansson L, Kiaii S et al. Signalling molecules and cytokine production in T cells of multiple myeloma-increased abnormalities with advancing stage. Br J Haematol 2004; 124: 315–324.
31. Yi Q, Osterborg A, Bergenbrant S et al. Idiotype-reactive T-cell subsets and tumor load in monoclonal gammopathies. Blood 1995; 86: 3043–3049.
32. Beyer M, Schultze JL. Regulatory T cells in cancer. Blood 2006; 108: 804–811.

Downloaded from http://annonc.oxfordjournals.org/ by guest on October 19, 2016
