# Supplementary material for: Expression of Concern: Prognostic value of circulating plasma cells in patients with multiple myeloma: A meta-analysis
Source: PLoS One. 2023 Feb 21;18(2):e0282230. doi: 10.1371/journal.pone.0282230 (PMC9942954; doi:10.1371/journal.pone.0282230)
Supplement: S1 File — (ZIP) [file pone.0282230.s001.zip › primary data/excluded research/2013 Detailed characterization of multiple myeloma circulating tumor cells shows unique phenotypic, cytogenetic, functional, and circadian distribution profile.pdf]

From www.bloodjournal.org by guest on November 15, 2015. For personal use only.

Regular Article

LYMPHOID NEOPLASIA

# Detailed characterization of multiple myeloma circulating tumor cells shows unique phenotypic, cytogenetic, functional, and circadian distribution profile

Bruno Paiva,[1] Teresa Paino,[2] Jose-Maria Sayagues,[2,3] Mercedes Garayoa,[2] Laura San-Segundo,[2] Montserrat Martín,[2] Ines Mota,[3] María-Luz Sanchez,[2,3] Paloma Bárcena,[2,3] Irene Aires-Mejia,[2] Luis Corchete,[2] Cristina Jimenez,[2] Ramon Garcia-Sanz,[2] Norma C. Gutierrez,[2] Enrique M. Ocio,[2] Maria-Victoria Mateos,[2] Maria-Belen Vidriales,[2] Alberto Orfao,[2,3] and Jesús F. San Miguel[1]

[1]Clinica Universidad de Navarra, Centro de Investigacion Medica Aplicada, Pamplona, Spain; [2]Hospital Universitario de Salamanca, Instituto de Investigaion Biomedica de Salamanca, Instituto de Biologia Molecular y Celular del Cancer (Univesidad de Salamanca - Consejo Superior de Investigaciones Cientificas), Salamanca, Spain; and [3]Servicio General de Citometría and Departamento de Medicina, Universidad de Salamanca, Salamanca, Spain

## Key Points

- Detailed characterization of myeloma circulating tumor cells shows that these represent a unique subpopulation of BM clonal PCs.
- Myeloma CTCs are clonogenic, quiescent, and may represent an ancestral clone potentially driven by circadian rhythms.

**Circulating myeloma tumor cells (CTCs) as defined by the presence of peripheral blood (PB) clonal plasma cells (PCs) are a powerful prognostic marker in multiple myeloma (MM). However, the biological features of CTCs and their pathophysiological role in MM remains unexplored. Here, we investigate the phenotypic, cytogenetic, and functional characteristics as well as the circadian distribution of CTCs vs paired bone marrow (BM) clonal PCs from MM patients. Our results show that CTCs typically represent a unique subpopulation of all BM clonal PCs, characterized by downregulation (*P* < .05) of integrins (CD11a/CD11c/CD29/CD49d/CD49e), adhesion (CD33/CD56/CD117/CD138), and activation molecules (CD28/CD38/CD81). Fluorescence in situ hybridization analysis of fluorescence-activated cell sorter–sorted CTCs also unraveled different cytogenetic profiles vs paired BM clonal PCs. Moreover, CTCs were mostly quiescent and associated with higher clonogenic potential when cocultured with BM stromal cells. Most interestingly, CTCs showed a circadian distribution which fluctuates in a similar pattern to that of CD34$^+$ cells, and opposite to stromal cell–derived factor 1 plasma levels and corresponding surface expression of CXC chemokine receptor 4 on clonal PCs, suggesting that in MM, CTCs may egress to PB to colonize/metastasize other sites in the BM during the patients' resting period. (*Blood*. 2013;122(22):3591-3598)**

## Introduction

In the late 2000s, 2 pivotal studies elegantly showed that monoclonal gammopathy of undetermined significance (MGUS) precedes multiple myeloma (MM) in most, if not all myeloma patients.[1,2] End-organ damage is the most important criterion to classify therapy-requiring MM patients, and the most common CRAB (hyperCalcemia, Renal failure, Anemia, Bone lesions) symptom is the presence of bone lesions detectable on a skeletal survey.[3] Extramedullary disease is present in ~10% of newly diagnosed symptomatic patients, but it increases up to 20% in the relapse/refractory setting,[4] and typically anticipates a dismal outcome. Plasma cell (PC) leukemia is one of the most aggressive forms of the disease and even with novel drugs, the outcome is very poor with both short remission and survival rates.[5] This landscape does not greatly differ from that of most solid tumors, in which the presence of metastasis is a key prognostic factor.[6] In fact, recent observations suggest that tumor cell dissemination is often an early event,[7] and the clinical sequel of circulating tumor cells (CTCs) has been the focus of extensive research.[8]

Interestingly, peripheral blood (PB) MM CTCs (morphologic and phenotypically defined as mature PCs) are also a common event throughout the spectrum of MM.[9-13] CTCs can only be detected in a small fraction of newly diagnosed MM patients (<15%) by conventional morphology.[14] However, this frequency increases up to 50% to 70% once more sensitive techniques (eg, immunohistochemistry[15] or flow cytometry[11]) are used. Interestingly, the presence of CTCs has been associated with an increased risk of malignant transformation to symptomatic MM in both MGUS[9] and smoldering MM,[12] as well as with an inferior survival among symptomatic newly diagnosed[10] and relapse/refractory MM.[13] Despite all the above, the biological characteristics of MM CTCs remain largely unexplored[11,16] with many unanswered questions, for example: (1) Are all bone marrow (BM) clonal PCs capable to

Submitted June 20, 2013; accepted September 22, 2013. Prepublished online as *Blood* First Edition paper, September 26, 2013; DOI 10.1182/blood-2013-06-510453.

The online version of this article contains a data supplement.

The publication costs of this article were defrayed in part by page charge payment. Therefore, and solely to indicate this fact, this article is hereby marked "advertisement" in accordance with 18 USC section 1734.

© 2013 by The American Society of Hematology

From www.bloodjournal.org by guest on November 15, 2015. For personal use only.



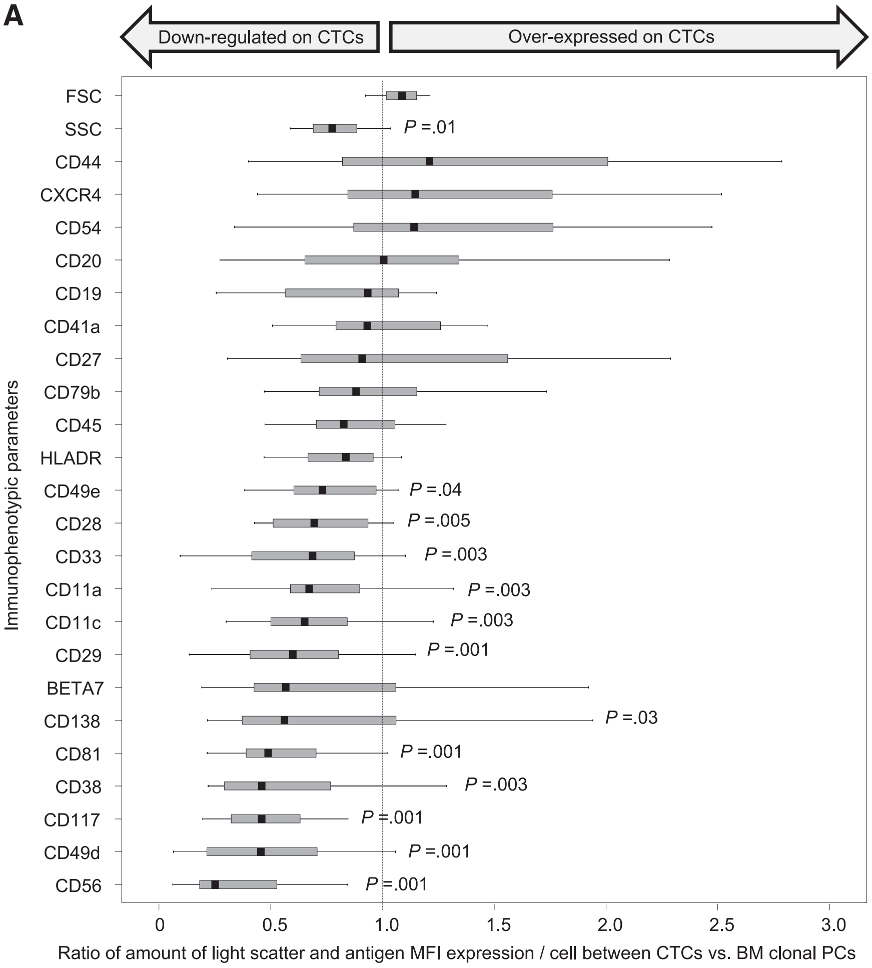


**Figure 1. Detailed immunophenotypic features of paired BM clonal PCs vs CTCs from 15 MM patients.** (A) Notched boxes represent the 25th and 75th percentile values of the ratio between the amount of FSC, SSC, or antigen MFI expression per paired CTCs/BM clonal PCs; the line in the middle and vertical lines correspond to the median value and both the 10th and 90th percentiles, respectively. (B) The corresponding iPEPs for each patient are shown. The iPEP of BM clonal PCs is represented by 1 and 2 SD lines, whereas their paired individual CTCs are represented by black dots. Each patient-specific iPEP is represented using the automated population separator (APS1) plot based on a graphical representation of principal component 1 (x-axis) vs principal component 2 (y-axis) for a total of 25 parameters studied (23 phenotypic markers plus FSC and SSC). In 12 of 15 patients (cases 1, 3, 4, 6, 8, 9, 10, 11, 12, 13, 14, 15) CTCs clustered in a unique area of the whole space occupied by their paired BM clonal PCs, whereas in the remaining cases (patients 2, 5, 7) CTCs were spread throughout the whole BM clonal PC compartment.

egress into PB, or only a specific subpopulation? (2) Do CTCs have stem cell–like features and are they enriched on clonogenic cells? or (3) Do circadian rhythms also affect CTCs (similarly to $CD34^+$ hematopoietic stem cells [HSCs])?

Here, we investigated the phenotypic, cytogenetic, and functional characteristics of CTCs from MM patients, by comparing them to patient-paired BM clonal PCs. Our results show that CTCs represent a unique subset of patient-paired BM clonal PCs with clonogenic potential and a quiescent phenotype, which may potentially be driven to circulate by circadian rhythms.

## Materials and methods

### Patients and samples

Overall, 46 patients with symptomatic MM (32 newly diagnosed cases and 24 patients with relapse/refractory disease) were prospectively studied, and staged according to the International Myeloma Working Group criteria.[3] In all patients, BM and PB samples were simultaneously collected after informed consent was given by each individual, according to the local ethical committees and the Helsinki Declaration protocol. Due to the limitations in the number of CTCs in each individual sample, a prioritization was made for the investigations to be performed. This explains the differences in the number of samples investigated/tested by each technique.

### MFC immunophenotyping

Approximately 100 μL and 1 mL of EDTA-anticoagulated BM and PB paired samples from 15 patients were immunophenotyped using a direct 8-color immunofluorescence stain-and-then-lyse technique, with 4 different combination of monoclonal antibodies (MoAbs) (Pacific Blue [PB], Pacific Orange [PacO], fluorescein isothiocyanate [FITC], phycoerythrin [PE], peridinin chlorophyll protein–cyanin 5.5 [PerCP-Cy5.5], PE–cyanin 7 [PE-Cy7], allophycocyanin [APC], Alexa Fluor 700 [AF700]): (1) CD29, CD45/CD11a, β7, CD79b, CD49d, CD19, CD38; (2) CD11c, CD45, CD41a, CD49e, CD33, CD117, CD19, CD38; (3) CD20, CD45, CD81, CD54, CD138, CD56, CD19, CD38; and (4) HLA-DR, CD45, CD44, CXC chemokine receptor 4 (CXCR4), CD27, CD28, CD19, CD38. Data acquisition was performed for ≥$10^6$ leukocytes per tube in a FACSCanto II flow cytometer (BD Biosciences) using the FACSDiva 6.1 software.

### Generation of iPEPs

In all BM and PB samples analyzed, the PC compartment was defined by the 5 parameters commonly investigated in each aliquot (CD38, CD45, CD19, forward light scatter [FSC], and sideward light scatter [SSC]). Then, the immunophenotypic protein expression profile (iPEP) of BM clonal PCs and CTCs for all 23 phenotypic markers analyzed plus FSC and SSC was generated for every single clonal PC, after merging of flow cytometry data files and calculation of data.[17] Briefly, the merge function of the Infinicyt software (Cytognos SL) was used to fuse the different data files corresponding to the 4 different 8-color MoAb combinations studied per sample, into a single data file containing all information measured for that sample. For any single cell in each 8-color MoAb combination, this included those antigens that were measured directly on it and antigens which were not evaluated directly ("missing values") for that cell in the corresponding tube that it was contained in. Then, the calculation function of the Infinicyt software was used to fill in those "missing values," based on the "nearest neighbor" statistical principle, in a space defined by its unique position in the multidimensional space created by the 5 common (backbone) parameters

From www.bloodjournal.org by guest on November 15, 2015. For personal use only.



**Figure 1. (Continued).**

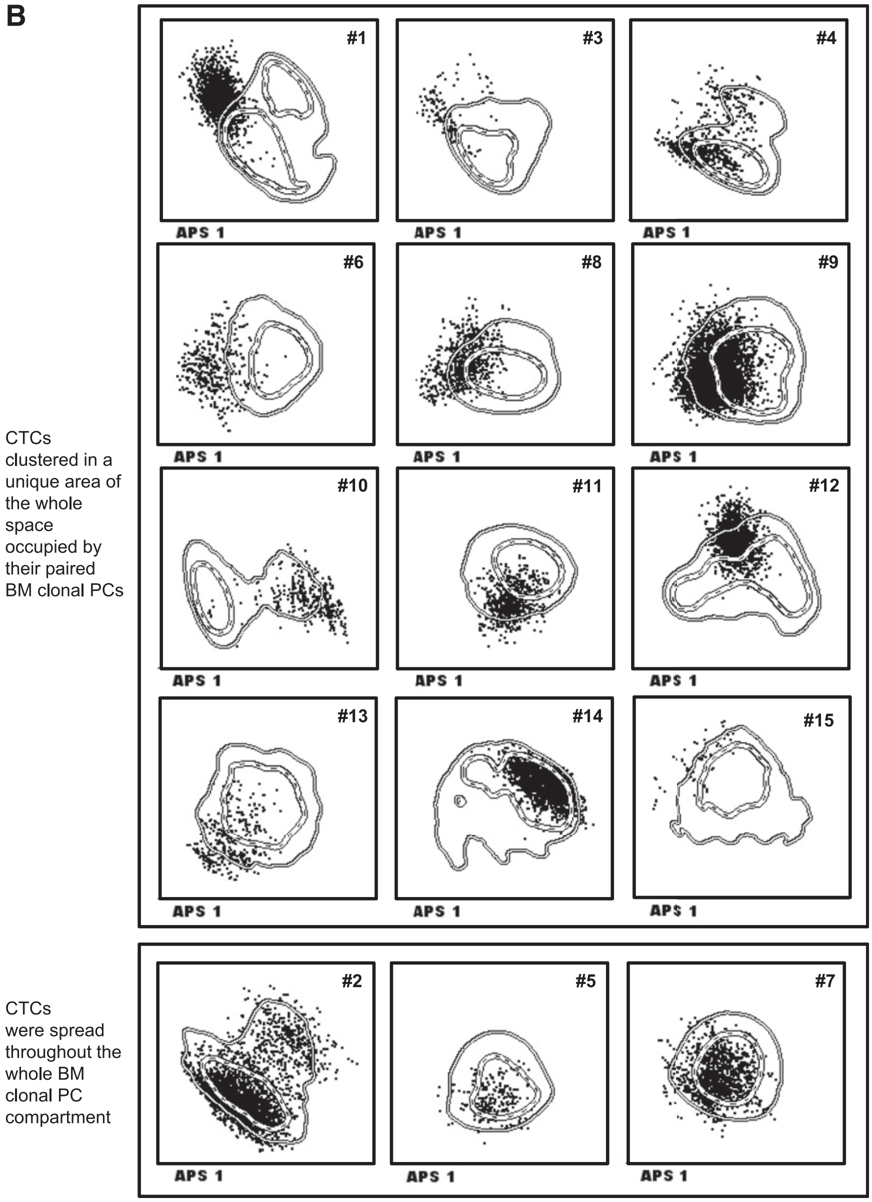


(FSC, SSC, CD38, CD45, and CD19). Finally, BM clonal PCs and CTCs were discriminated from normal PCs by aberrant phenotypic expressions (extensively described elsewhere[18]), and they were compared for the 25 parameters evaluated by principal component analysis and represented using the automated population separator (APS1; principal component 1 vs principal component 2) graphical representation of the Infinicyt software.[17]

## Cell-cycle analyses

The proliferation index of BM clonal PCs and CTCs was analyzed in paired PB and BM samples from 10 MM patients using 5-color staining for nuclear DNA and 4 cell-surface antigens. Briefly, 100 μL and 300 μL of EDTA-anticoagulated BM and PB samples, respectively, were incubated for 15 minutes in the dark (room temperature) with the following combination of MoAb (PacB, PacO, FITC, PE): CD19, CD45, CD38, CD56. After lysing nonnucleated red cells, the nucleated cells were washed and stained with 3 μL per tube of DRAQ5 (Vitro SA). After another 10-minute incubation, samples were immediately acquired in a FACSCanto II flow cytometer using the FACSDiva software program, and information on $\geqslant 10^6$ cells corresponding to the whole BM and PB cellularity was measured and stored. Data were analyzed with the Infinicyt software and the overall percentage of proliferating cells (including those cells in the S-phase of the cell cycle) was calculated, as described elsewhere.[19]

## Colony assays

Patient-specific aberrant phenotypes were used to sort BM clonal PCs and CTCs (purity >95%) from paired BM and PB heparinized samples from 10 MM patients, using a FACSAria III flow cytometer (BD Biosciences). For colony assays, the same number of BM clonal PCs and CTCs were plated into Methocult media (StemCell Technologies) supplemented with 10% lymphocyte-conditioned media (IL-6, 20 ng/mL plus insulinlike growth factor-1 (IGF-1), 20 ng/mL) and cocultured with the hMSC-TERT human mesenchymal stem cell (MSC) line immortalized by expression of the telomerase reverse transcriptase gene (a generous gift from Dr D. Campana; Department of Paediatrics, Yong Loo Lin School of Medicine, National University of Singapore, Republic of Singapore), at a ratio of 10:1. Cells were cultured at 37°C in a 5% $CO_2$ humidified atmosphere; at day +14 of culture, colonies ($\geqslant$40 cells) and clusters ($\geqslant$10 cells) were scored. A negative control with the hMSC-TERT cell line incubated under identical conditions was used along with all patient samples, such control systematically showing no colonies or clusters at day +14 in the 10 experiments performed.

From www.bloodjournal.org by guest on November 15, 2015. For personal use only.



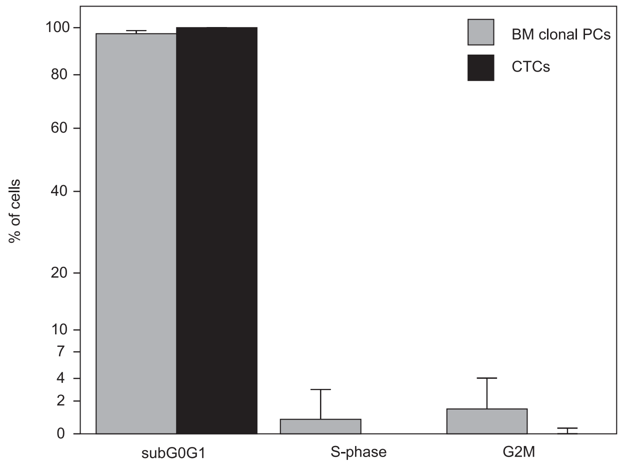


**Figure 2. Distribution of BM clonal PCs and their corresponding CTCs from 10 MM patients among the different stages of the cell cycle: sub-G0G1, S-phase, and G2M.** Results are expressed as median percentage of BM clonal PCs and CTCs in each of the 3 stages and the upper bound of the 95% confidence intervals (vertical lines).

### Assessment of circadian distribution of CTCs, $CD34^+$ HSCs, CXCR4, and SDF1

The absolute number of PB clonal PCs as well as $CD34^+$ HSCs was measured in PB samples from 6 MM patients, using the following MoAb combination (PacB, OC515, FITC, PE, PerCP-Cy5.5, PE-Cy7, APC): CD45, CD138, CD38, CXCR4, CD34, CD19, CD56. PB samples were collected using a central line after discarding the first 2 mL. Measurements started at 4:00 PM and they were repeated every 4 hours up to 12:00 PM the next day, when patients initiated anti-myeloma treatment. Plasma samples were collected in parallel and the stromal cell–derived factor 1 (SDF1) plasma levels were measured using a quantitative enzyme-linked immunosorbent assay (Quantikine; R&D Systems) following the manufacturer's recommendations.

### FISH studies

Fluorescence-activated cell sorter (FACS)-sorted BM and PB (paired) clonal PCs from 5 MM patients were initially screened (as part of the clinical workout) by interphase fluorescence in situ hybridization (FISH), for the presence of immunoglobulin heavy locus (IGH) translocations, del(13q14) and del(17p13). Then, due to the expected low number of sorted CTCs, selected FISH probes targeting chromosomal abnormalities commonly found in MM[20,21] (1q21, 6q21, 9q34, 13q14, 14q,32, Chr15, or 17p13) were used for further cytogenetic characterization of BM clonal PCs and CTCs; a minimum of 100 interphase nuclei were analyzed per probe/probe combination, as described elsewhere.[22]

### Statistical analyses

The Wilcoxon signed rank test was used to evaluate the statistical significance of differences observed between BM and PB clonal PCs. For all statistical analyses, SPSS software (version 15.0; SPSS Inc.) was used.

## Results

### Myeloma CTCs show unique iPEP

Myeloma CTCs were measured in the 46 patients; the median absolute number of CTCs per microliter was 0.93 (0.07-905). Paired BM and PB samples were analyzed in 15 patients. Compared with BM clonal PCs, MM CTCs showed downregulation ($P < .05$) of the CD11a, CD11c, CD29, CD49,d and CD49e integrins, as well as of the CD33 and CD56 adhesion molecules, and the stem cell factor receptor CD117, as reflected by a decreased mean fluorescence intensity (MFI) per PC of each of these markers (Figure 1A). CD28, CD38, and CD81, a tetraspanin involved in B-cell activation and proliferation, were also downregulated ($P < .05$) in CTCs. CD138 was typically underexpressed on CTCs (median MFI of 2141 vs 3665 arbitrary units in BM clonal PCs; $P = .03$). In addition, cellular complexity, as defined by

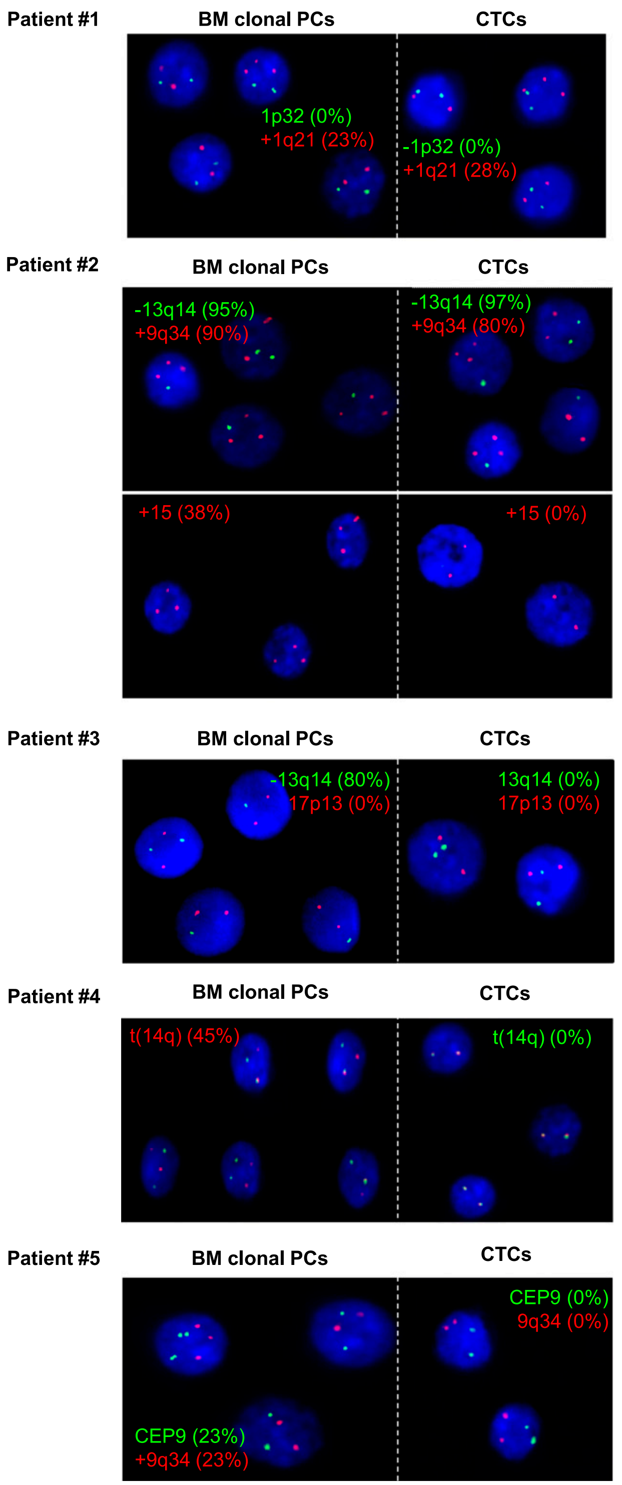


**Figure 3. Representative FISH microscopic photographs of FACS-purified PC nuclei from BM clonal PCs and their paired CTCs from 5 MM patients.** For each patient, selected probes with the corresponding percentage of altered PC nuclei are shown for BM clonal PCs (left panels) and their paired CTCs (right panels).

From www.bloodjournal.org by guest on November 15, 2015. For personal use only.



**Table 1. Frequency of colonies and clusters**

| Patient (no. of cells) | No. of colonies | | No. of clusters | |
|---|---|---|---|---|
| | BM clonal PCs | CTCs | BM clonal PCs | CTCs |
| 1 (1200) | 0 | 0 | 0 | 0 |
| 2 (5300) | 0 | 1 | 0 | 0 |
| 3 (6500) | 2 | 5 | 0 | 2 |
| 4 (10 000) | 0 | 0 | 0 | 0 |
| 5 (10 000) | 0 | 0 | 0 | 0 |
| 6 (40 000) | 0 | 0 | 0 | 3 |
| 7 (72 000) | 0 | 0 | 0 | 0 |
| 8 (80 000) | 0 | 0 | 1 | 14 |
| 9 (100 000) | 0 | 0 | 0 | 0 |
| 10 (349 000) | 0 | 0 | 0 | 0 |

Frequency of colonies and clusters scored at day +14 after coculture of the same number of paired BM clonal PCs and CTCs with the human mesenchymal stem cell line immortalized by expression of the telomerase reverse transcriptase gene, hMSC-TERT.

SSC was also significantly decreased in CTCs vs BM clonal PCs. In turn, no differences were noted ($P > .05$) for other maturation-related markers such as CD19, CD20, CD27, CD45, and CD79b, as well as for the SDF1 chemokine receptor 4 (CXCR4). Patient-based comparisons between the iPEP of CTCs (black dots in Figure 1B) and BM clonal PCs (defined by 1 and 2 SD curves in Figure 1B) revealed that CTCs consistently displayed overlapping phenotypic features with BM clonal PCs; however, in 12 of 15 patients (cases 1, 3, 4, 6, 8, 9, 10, 11, 12, 13, 14, 15) CTCs clustered in a uniquely restricted area of that occupied by BM clonal PCs, whereas in the remaining cases (patients 2, 5, 7) CTCs were spread throughout the whole BM clonal PC compartment (Figure 1B).

### Myeloma CTCs are mostly quiescent

Overall, the proliferation index (percentage of cells in S phase) of BM clonal PCs was significantly higher than that of their paired CTCs (median, 0.8% vs 0%; range, 0.5%-5% vs 0%-0.8%; $P = .005$). Accordingly, virtually all CTCs (median, 100%; range, 97%-100%) were in the sub-G0G1 phase of the cell cycle (Figure 2), this number being significantly higher ($P = .005$) than that found in BM clonal PCs (median, 97%; range, 85%-99%).

### Myeloma CTCs consist of unique cytogenetic subclones of BM clonal PCs

Comparison between the iPEP of BM clonal PCs and CTCs suggested that, at least in a large subset of patients, CTCs would represent a unique subset of the whole BM clonal PC compartment. To build up on these observations, we investigated a selected number of chromosomal abnormalities in highly purified FACS-sorted BM clonal PCs and CTCs from 5 patients (Figure 3). Patient 1 was negative for t(IGH), del(13q), and del(17p), and both BM clonal PCs and CTCs were diploid for 6q21 and 9q34. In turn, gains at 1q21 were observed in 23% and 28% of BM clonal PCs and CTCs, respectively. Patient 2 had del(13q14) and gain of 9q34 in clonal BMPCs (95% and 90%, respectively), with similar percentages being found in CTCs (97% and 80%, respectively); however, trisomy 15 was observed in 38% of BM clonal PCs while undetectable in the corresponding CTCs. Patient 3 had del(13q14) in 80% of BM clonal PCs and del(17p13) was also present in 8% of these cells; in turn, no del(13q) and del(17p) was observed in the patient's CTCs. Similarly, an IGH translocation was detected in 45% of clonal BMPCs from patient 4, but the corresponding CTCs lacked this specific cytogenetic abnormality. In

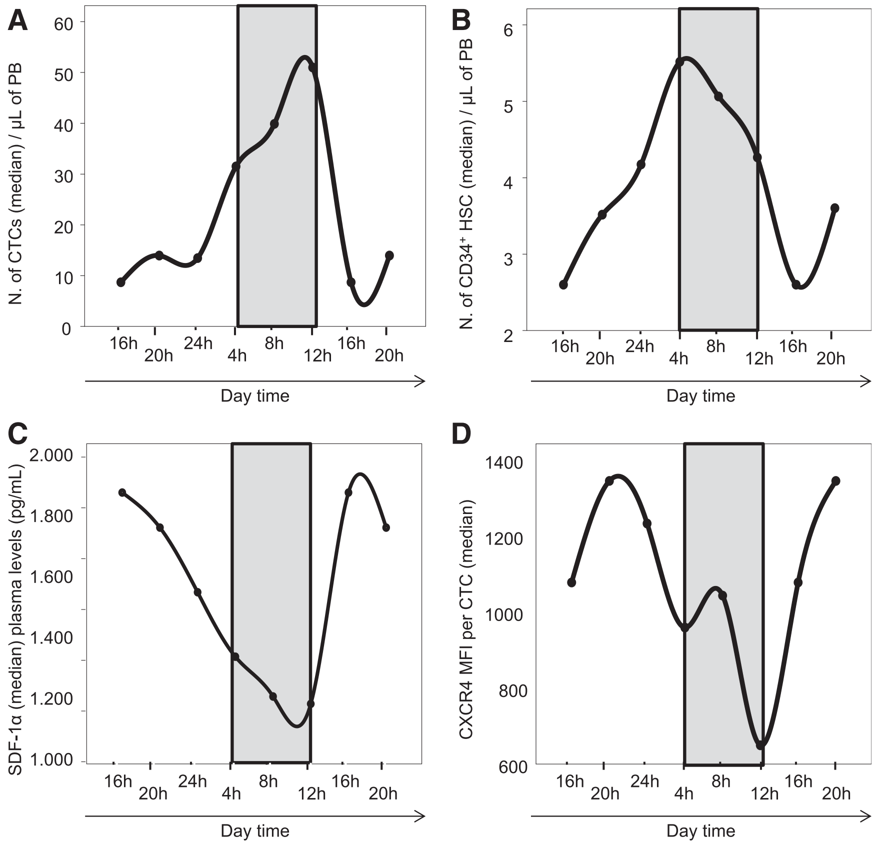


**Figure 4. Circadian rythms in myeloma.** Circadian distribution of CTCs (A), CD34$^{+}$ HSCs and progenitor cells (B), SDF1α plasma levels (C), and the levels of CXCR4 surface expression per CTCs (D) in MM patients (N = 6). All measurements started at 4:00 PM and they were repeated every 4 hours up to 12:00 PM, the next day (when the patients initiated antimyeloma therapy). The 4:00 PM and 8:00 PM time points are repeated at both sides of all 4 graphics to facilitate viewing of the circadian variations.

From www.bloodjournal.org by guest on November 15, 2015. For personal use only.



patient 5, gain of chromosome 9q34 was found in 23% of BM clonal PCs, while all CTCs were diploid for 9q34. To confirm the clonal nature of CTCs from this latter patient, we determined the CDR3 sequence of the rearranged IGH gene from BM clonal PCs and evaluated it by allele specific oligonecleotide–polymerase chain reaction (ASO-PCR) on sorted CTCs. As expected, CTCs showed a clonotypic IGH VDJ gene rearrangement identical to that of their corresponding BM clonal PCs (data not shown).

### Clonogenic potential of myeloma CTCs

Though CTCs were mostly quiescent, they should display some potential to expand if they are involved in the myeloma "metastatic"/dissemination process. In such a case, CTCs should have clonogenic potential when, after circulating in PB, they home again into the BM at a different location. To mimic this process, we cocultured identical numbers (≤349 000) of BM clonal PC and their corresponding CTCs with the -hMSC-TERT cell line. In 3 of 10 patients, clonal PC colonies (N = 2) and/or clusters (N = 3) were observed at day +14 of culture (Table 1). BM clonal PCs and paired CTCs originated colonies simultaneously in only 1 patient (2 and 5 colonies, respectively), whereas in a second patient only CTCs showed clonogenic potential (1 colony). In the first patient, 2 clusters were also observed in the CTC fraction but not in the corresponding BM clonal PC compartment. In the third patient, CTCs formed 14 PC clusters whereas BM clonal PCs only gave rise to 1 cluster. Overall, CTCs showed a threefold colony- and a 16-fold cluster-formation clonogenic potential vs paired BM clonal PCs.

### Circadian rhythm of myeloma CTCs

The number of myeloma CTCs showed a marked fluctuation during the day in each of the 6 patients analyzed (supplemental Figure 1, available on the *Blood* website). Overall, median myeloma CTCs numbers peaked between 4:00 AM (32 cells/μL) and 12:00 PM (51 cells/μL) and they reached the lowest levels between 4:00 PM (9 cells/μL) and 12:00 AM (13 cells/μL) (Figure 4A). Of note, this pattern was observed in 5 of 6 cases (supplemental Figure 1). Interestingly, the number of PB $CD34^+$ HSCs showed a similar pattern of circadian variation (Figure 4B), the median number of $CD34^+$ HSCs peaking at 4:00 AM (5.0 cells/μL) and reaching a nadir at 4:00 PM (2.4 cells/μL). Furthermore, the median SDF1 plasma levels showed a mirror fluctuation compared with CTCs and HSCs (Figure 4C), peaking at 4:00 PM (1892 pg/mL) with nadir at 12:00 PM (1198 pg/mL). Interestingly, CXCR4 expression levels (as defined by its MFI per cell) on the surface of myeloma CTCs followed a similar pattern to its ligand (SDF1), peaking at 8:00 PM (1344 MFI arbitrary units) and reaching the lowest levels at 12:00 PM (650 MFI arbitrary units) (Figure 4C-D).

## Discussion

Presence of circulating clonal PCs in the PB of MM patients has long been recognized and it has been associated with more advanced stages and aggressive disease, particularly when clonal PCs decrease their BM microenvironment dependency.[10] However, the specific mechanisms and significance of circulating PB PCs remains poorly understood. In recent years, it has been speculated that the presence of CTCs emulate the physiological trafficking of normal BM PCs[23-25] and $CD34^+$ HSCs through PB.[26] Malignant PCs would leave the BM, recirculate into PB, and home again into the BM at a different location, in a kind of "metastatic"/dissemination process that spreads the disease over the whole BM.[11,27] If this hypothesis holds true, CTCs should display clonogenic potential so that upon homing into the BM, they could recapitulate the disease.[11,27] To mimic this process, we cocultured CTCs from 10 different patients with BM stromal cells; after 14 days, colonies and/or clusters derived from CTCs were observed in a significant proportion of cases. In turn, identical numbers of paired BM clonal PCs showed lower numbers of colonies and clusters restricted to fewer patients when cultured in identical conditions. Overall, the here reported frequency of clonogenic PCs after culturing in vitro rather low numbers of (PB or BM) clonal PCs (≤349 000) are in line with the efficacy recently described using ~$10^5$ primary BM PCs (3 of 13 samples).[28] These results suggest that the fraction of clonal PCs that exit the BM into the PB (CTCs) could be enriched on clonogenic cells, but also that clonogenic cells are relatively rare among all myeloma tumor cells, particularly in the BM.

Recent reports from our and other groups have shown that circulating PB myeloma PCs have mild phenotypic differences when compared with BM clonal PCs, particularly with regard to CD138 expression.[11,16] Here, we confirm that the level of expression of CD138 on the surface membrane of CTCs is inferior to that found among paired BM clonal PCs, but in the absence of a clear cutoff. This implies that CD138 on its own is not a suitable marker for the specific identification of the CTC fraction among the whole population of clonal PCs in the BM,[16] indicating that the utility of other proteins should be explored in this regard. Here, we compared the overall iPEP of CTCs to their BM counterpart for a large panel of 23 markers using the new analytic tools developed by the EuroFlow Consortium.[17] Our results show that CTCs have an iPEP which systematically overlaps with that of their paired BM clonal PCs. Despite this, in the great majority of the patients, CTCs clustered in a unique position which overlaps with only a fraction of the whole BM tumor bulk, pointing out the potential existence of both down- and upregulation of specific antigens when compared with clonal PCs from the BM of the same patient. In line with this hypothesis, detailed analysis of the different proteins evaluated showed that CTCs display lower levels of several integrins (eg, CD11a, CD11c, CD29 CD49d, CD49e), CD33 and the N-CAM (CD56) adhesion molecules, and the stem cell factor receptor (CD117). These observations confirm previous findings at the single patient level, which suggested that absence of CD56 expression in BM clonal PCs would reflect an increased extramedullary spread potential and more aggressive disease,[29] whereas CD117 expression in MM is associated with a favorable outcome[18] potentially related to an enhanced anchor of clonal PCs to BM niches through kit ligand–expressing stromal cells.[30] Our results support this later hypothesis, suggesting that CTCs represent a phenotypic subset of BM myeloma PCs with lower expression of integrin and adhesion molecules and consequently a lower dependence on BM stromal cell niches would have an enhanced capacity to egress into PB. Of note, CD28, CD38, and CD81 were also found to be downregulated in MM CTCs vs paired BM clonal PCs. CD38 has been adscribed a dual adhesion and signaling function,[31] whereas CD28 has recently been identified as an important molecule in the interaction between PCs and BM stromal dendritic cells.[32] CD81 is an upstream molecule of the B-cell coreceptor which is also involved in B-cell signaling and activation[33]; interestingly, we have recently described that CD81 expression in MM BM PCs is associated with an increased proliferation index.[34] In line with these findings, CTCs were associated with a quiescent state, and a markedly reduced number of proliferating cells as compared with paired BM clonal PCs was also found.

From www.bloodjournal.org by guest on November 15, 2015. For personal use only.



Quiescence, potential to self-renew, and chemoresistance are classical hallmarks of both normal and cancer stem cells.[35,36] In MM, there is a persistent and ongoing debate about the potential cell of origin of the disease; while the work of some groups supports a post–germinal center memory B-cell detectable in PB as the putative myeloma stem cell,[37-39] others have shown for years[40,41] that the clonogenic potential of MM cells could be restricted to the mature CD138$^+$ PC compartment.[16,28,42-47] In the present study, we found PB CTCs to be associated with a great in vitro clonogenic capacity and a quiescent state. These results would also support recent observations showing the existence of a more immature fraction of drug-resistant, relatively quiescent, and clonogenic clonal PCs lacking CD138,[16,28,48] which would be preferentially located in extramedullary sites.[16]

Another frequent feature of cancer stem cells, relates to the fact that they frequently share signaling, functional, and phenotypic features with normal stem cells, representing the more immature fraction of all cancer cells within a tumor.[40] From a phenotypic point of view, MM CTCs shared a phenotype which reflects accurately that of cells at the same maturation stage as their paired BM clonal PCs, both tumor subpopulations showing similar levels of expression of maturation-related B-cell markers such as CD19, CD20, CD27, CD45, or CD79b. However, FISH analysis on highly purified FACS-sorted CTCs and their paired BM clonal PCs, unraveled a different cytogenetic profile between the 2 tumor subpopulations in most MM patients analyzed. Most interestingly, despite a common cytogenetic background, BM clonal PCs showed systematically $\geqslant$1 cytogenetic alteration which was not detected in CTCs. This illustrates that CTCs may have a different cytogenetic profile as compared with BM clonal PCs. Whether or not this could also be associated with a different phenotypic profile remains to be answered; nevertheless, we and others have previously shown that some antigenic profiles (eg, CD20, CD28, or CD117) are associated with the presence of specific cytogenetic abnormalities. Further investigations in larger series of patients and using high-throughput technology on FACS-sorted paired PB and BM clonal PCs are warranted to confirm whether CTCs do in fact represent an ancestral clone from a cytogenetic point of view. Independent of such differences between CTCs and BM clonal PCs, our results would fit into the recent proposed model of tumor evolution, genetic diversification, and clonal selection, within the adaptive landscapes of tissue ecosystems (eg, BM vs PB).[49,50] However, from a functional point of view, it was surprising to see the marked fluctuation of the daily absolute number of CTCs in the PB of MM patients, peaking between 4:00 AM and 12:00 PM, with a nadir between 4:00 PM and 12:00 AM. These results, although preliminary due to low patient number, suggest that similarly to CD34$^+$ HSCs, MM CTCs may egress to PB during the patients' resting period to colonize/metastasize other sites in the BM or at extramedullary tissues.[26] This concept has been elegantly proposed by Mendez-Ferrer et al[26] after pivotal observations that in mice, the cyclical release of HSCs and expression of SDF1 are both regulated by core genes of the molecular clock through circadian noradrenaline secretion by the sympathetic nervous system. Here, we show for the first time that CTCs from MM patients follow a circadian rhythm that resembles that of CD34$^+$ HSCs; moreover, the traffic of CTCs also seems to be modulated by SDF1 levels, which together with CXCR4 expression on the PC surface, fluctuated in a mirror basis with respect to the absolute number of MM CTCs. Subsequent studies are needed to fully elucidate the mechanisms modulating the traffic of CTCs, and to clarify whether this is specific MM biological behavior or instead, a secondary manifestation of normal circadian rhythms typically observed in normal myeloid stem cells.

In summary, in the present study, we show for the first time that MM CTCs represent a unique subpopulation of clonal BM PCs, which seems to be enriched in mostly quiescent clonogenic cells, an ancestral cytogenetic background, and a circadian spread into PB that mimics that of CD34$^+$ HSCs. Further investigations in larger series of patients are warranted to confirm and extent on these findings, focusing on the specific transcriptome and epigenetic profile of CTCs, so that specific therapies targeting this subpopulation can be developed.

## Acknowledgments

This work was supported by the Cooperative Research Thematic Network grants RD12/0036/0071, RD12/0036/0058, RD12/0036/0069, and RD12/0036/0048, of the Red de Cancer (Cancer Network of Excellence), Instituto de Salud Carlos III, Spain, the Subdirección General de Investigación Sanitaria (FIS: PI060339, 06/1354, 02/0905, 01/0089/01-02, PS09/01897/01370, PI112/02311), Asociación Española Contra el Cáncer (GCB120981SAN), Madrid, Spain, Fundación Memoria de D. Samuel Solórzano Barruso, Salamanca, Spain, and the Multiple Myeloma Research Foundation 2012 Research Fellow Award.

## Authorship

Contribution: J.F.S.M., A.O., and B.P. conceived the idea and designed the study protocol; B.P., M.-L.S., P.B., I.A.-M., and M.-B.V. performed immunophenotypic analysis and cell sorting; T.P., M.G., L.S.-S., and M.M. performed cell-culture and CFU experiments; J.-M.S., I.M., and N.C.G. performed FISH studies; C.J. and R.G.-S. performed ASO-PCR assessments; R.G.-S., N.C.G., E.M.O., M.-V.M., and J.F.S.M. provided study material or patients; B.P., T.P., and L.C. performed statistical analysis; B.P., T.P., A.O., and J.F.S.M. analyzed and interpreted data; B.P., A.O., and J.F.S.M. wrote the manuscript; and all authors reviewed and approved the manuscript.

Conflict-of-interest disclosure: The authors declare no competing financial interests.

Correspondence: Jesús F. San Miguel, Clinica Universidad de Navarra; Centro de Investigacion Medica Aplicada (CIMA), Av. Pio XII 36, 31008 Pamplona, Spain; e-mail: sanmiguel@unav.es.

## References

1. Weiss BM, Abadie J, Verma P, Howard RS, Kuehl WM. A monoclonal gammopathy precedes multiple myeloma in most patients. *Blood.* 2009; 113(22):5418-5422.
2. Landgren O, Kyle RA, Pfeiffer RM, et al. Monoclonal gammopathy of undetermined significance (MGUS) consistently precedes multiple myeloma: a prospective study. *Blood.* 2009;113(22):5412-5417.
3. Dimopoulos M, Kyle R, Fermand JP, et al; International Myeloma Workshop Consensus Panel 3. Consensus recommendations for standard investigative workup: report of the International Myeloma Workshop Consensus Panel 3. *Blood.* 2011;117(18):4701-4705.
4. Bladé J, Fernández de Larrea C, Rosiñol L, Cibeira MT, Jiménez R, Powles R. Soft-tissue plasmacytomas in multiple myeloma: incidence,

From www.bloodjournal.org by guest on November 15, 2015. For personal use only.



mechanisms of extramedullary spread, and treatment approach. *J Clin Oncol.* 2011;29(28):3805-3812.

5. Fernández de Larrea C, Kyle RA, Durie BG, et al; International Myeloma Working Group. Plasma cell leukemia: consensus statement on diagnostic requirements, response criteria and treatment recommendations by the International Myeloma Working Group. *Leukemia.* 2013;27(4):780-791.
6. Rice J. Metastasis: The rude awakening. *Nature.* 2012;485(7400):S55-S57.
7. Polzer B, Klein CA. Metastasis awakening: the challenges of targeting minimal residual cancer. *Nat Med.* 2013;19(3):274-275.
8. Yu M, Ting DT, Stott SL, et al. RNA sequencing of pancreatic circulating tumour cells implicates WNT signalling in metastasis. *Nature.* 2012; 487(7408):510-513.
9. Kumar S, Rajkumar SV, Kyle RA, et al. Prognostic value of circulating plasma cells in monoclonal gammopathy of undetermined significance. *J Clin Oncol.* 2005;23(24):5668-5674.
10. Nowakowski GS, Witzig TE, Dingli D, et al. Circulating plasma cells detected by flow cytometry as a predictor of survival in 302 patients with newly diagnosed multiple myeloma. *Blood.* 2005;106(7):2276-2279.
11. Paiva B, Pérez-Andrés M, Vídriales MB, et al; GEM (Grupo Español de MM)/PETHEMA (Programa para el Estudio de la Terapéutica en Hemopatías Malignas); Myeloma Stem Cell Network (MSCNET). Competition between clonal plasma cells and normal cells for potentially overlapping bone marrow niches is associated with a progressively altered cellular distribution in MGUS vs myeloma. *Leukemia.* 2011; 25(4):697-706.
12. Bianchi G, Kyle RA, Larson DR, et al. High levels of peripheral blood circulating plasma cells as a specific risk factor for progression of smoldering multiple myeloma. *Leukemia.* 2013;27(3):680-685.
13. Peceliunas V, Janiulioniene A, Matuzeviciene R, Zvirblis T, Griskevicius L. Circulating plasma cells predict the outcome of relapsed or refractory multiple myeloma. *Leuk Lymphoma.* 2012;53(4):641-647.
14. San Miguel JF, Gutiérrez NC, Mateo G, Orfao A. Conventional diagnostics in multiple myeloma. *Eur J Cancer.* 2006;42(11):1510-1519.
15. Billadeau D, Van Ness B, Kimlinger T, et al. Clonal circulating cells are common in plasma cell proliferative disorders: a comparison of monoclonal gammopathy of undetermined significance, smoldering multiple myeloma, and active myeloma. *Blood.* 1996;88(1):289-296.
16. Chaidos A, Barnes CP, Cowan G, et al. Clinical drug resistance linked to interconvertible phenotypic and functional states of tumor-propagating cells in multiple myeloma. *Blood.* 2013;121(2):318-328.
17. Kalina T, Flores-Montero J, van der Velden VH, et al; EuroFlow Consortium (EU-FP6, LSHB-CT-2006-018708). EuroFlow standardization of flow cytometer instrument settings and immunophenotyping protocols. *Leukemia.* 2012;26(9):1986-2010.
18. Mateo G, Montalbán MA, Vidriales MB, et al; PETHEMA Study Group; GEM Study Group. Prognostic value of immunophenotyping in multiple myeloma: a study by the PETHEMA/GEM cooperative study groups on patients uniformly treated with high-dose therapy. *J Clin Oncol.* 2008;26(16):2737-2744.
19. Matarraz S, Teodosio C, Fernandez C, et al. The proliferation index of specific bone marrow cell compartments from myelodysplastic syndromes is associated with the diagnostic and patient outcome. *PLoS ONE.* 2012;7(8):e44321.
20. Avet-Loiseau H, Li C, Magrangeas F, et al. Prognostic significance of copy-number alterations in multiple myeloma. *J Clin Oncol.* 2009;27(27):4585-4590.
21. López-Corral L, Sarasquete ME, Beà S, et al. SNP-based mapping arrays reveal high genomic complexity in monoclonal gammopathies, from MGUS to myeloma status. *Leukemia.* 2012; 26(12):2521-2529.
22. Fernandez C, Santos-Silva MC, López A, et al. Newly diagnosed adult AML and MPAL patients frequently show clonal residual hematopoiesis [published online ahead of print April 12, 2013]. *Leukemia.*
23. Mei HE, Yoshida T, Sime W, et al. Blood-borne human plasma cells in steady state are derived from mucosal immune responses. *Blood.* 2009; 113(11):2461-2469.
24. Radbruch A, Muehlinghaus G, Luger EO, et al. Competence and competition: the challenge of becoming a long-lived plasma cell. *Nat Rev Immunol.* 2006;6(10):741-750.
25. Caraux A, Klein B, Paiva B, et al; Myeloma Stem Cell Network. Circulating human B and plasma cells. Age-associated changes in counts and detailed characterization of circulating normal CD138- and CD138+ plasma cells. *Haematologica.* 2010;95(6):1016-1020.
26. Méndez-Ferrer S, Lucas D, Battista M, Frenette PS. Haematopoietic stem cell release is regulated by circadian oscillations. *Nature.* 2008;452(7186): 442-447.
27. Ghobrial IM. Myeloma as a model for the process of metastasis: implications for therapy. *Blood.* 2012;120(1):20-30.
28. Hosen N, Matsuoka Y, Kishida S, et al. CD138-negative clonogenic cells are plasma cells but not B cells in some multiple myeloma patients. *Leukemia.* 2012;26(9):2135-2141.
29. Sahara N, Takeshita A. Prognostic significance of surface markers expressed in multiple myeloma: CD56 and other antigens. *Leuk Lymphoma.* 2004; 45(1):61-65.
30. Schmidt-Hieber M, Pérez-Andrés M, Paiva B, et al. CD117 expression in gammopathies is associated with an altered maturation of the myeloid and lymphoid hematopoietic cell compartments and favorable disease features. *Haematologica.* 2011;96(2):328-332.
31. de Weers M, Tai YT, van der Veer MS, et al. Daratumumab, a novel therapeutic human CD38 monoclonal antibody, induces killing of multiple myeloma and other hematological tumors. *J Immunol.* 2011;186(3):1840-1848.
32. Nair JR, Carlson LM, Koorella C, et al. CD28 expressed on malignant plasma cells induces a prosurvival and immunosuppressive microenvironment. *J Immunol.* 2011;187(3): 1243-1253.
33. van Zelm MC, Smet J, Adams B, et al. CD81 gene defect in humans disrupts CD19 complex formation and leads to antibody deficiency. *J Clin Invest.* 2010;120(4):1265-1274.
34. Paiva B, Gutiérrez NC, Chen X, et al; GEM (Grupo Español de Mieloma)/PETHEMA (Programa para el Estudio de la Terapéutica en Hemopatías Malignas) cooperative. Clinical significance of CD81 expression by clonal plasma cells in high-risk smoldering and symptomatic multiple myeloma patients. *Leukemia.* 2012;26(8):1862-1869.
35. Reya T, Morrison SJ, Clarke MF, Weissman IL. Stem cells, cancer, and cancer stem cells. *Nature.* 2001;414(6859):105-111.
36. Nguyen LV, Vanner R, Dirks P, Eaves CJ. Cancer stem cells: an evolving concept. *Nat Rev Cancer.* 2012;12(2):133-143.
37. Matsui W, Huff CA, Wang Q, et al. Characterization of clonogenic multiple myeloma cells. *Blood.* 2004;103(6):2332-2336.
38. Pilarski LM, Hipperson G, Seeberger K, Pruski E, Coupland RW, Belch AR. Myeloma progenitors in the blood of patients with aggressive or minimal disease: engraftment and self-renewal of primary human myeloma in the bone marrow of NOD SCID mice. *Blood.* 2000; 95(3):1056-1065.
39. Matsui W, Wang Q, Barber JP, et al. Clonogenic multiple myeloma progenitors, stem cell properties, and drug resistance. *Cancer Res.* 2008;68(1):190-197.
40. Yaccoby S, Epstein J. The proliferative potential of myeloma plasma cells manifest in the SCID-hu host. *Blood.* 1999;94(10):3576-3582.
41. Yaccoby S, Barlogie B, Epstein J. Primary myeloma cells growing in SCID-hu mice: a model for studying the biology and treatment of myeloma and its manifestations. *Blood.* 1998;92(8):2908-2913.
42. Jakubikova J, Adamia S, Kost-Alimova M, et al. Lenalidomide targets clonogenic side population in multiple myeloma: pathophysiologic and clinical implications. *Blood.* 2011;117(17):4409-4419.
43. Pfeifer S, Perez-Andres M, Ludwig H, Sahota SS, Zojer N. Evaluating the clonal hierarchy in light-chain multiple myeloma: implications against the myeloma stem cell hypothesis. *Leukemia.* 2011;25(7):1213-1216.
44. Chiron D, Surget S, Maïga S, et al. The peripheral CD138+ population but not the CD138-population contains myeloma clonogenic cells in plasma cell leukaemia patients. *Br J Haematol.* 2012;156(5):679-683.
45. Paíno T, Ocio EM, Paiva B, et al. CD20 positive cells are undetectable in the majority of multiple myeloma cell lines and are not associated with a cancer stem cell phenotype. *Haematologica.* 2012;97(7):1110-1114.
46. Christensen JH, Jensen PV, Kristensen IB, Abildgaard N, Lodahl M, Rasmussen T. Characterization of potential CD138 negative myeloma "stem cells". *Haematologica.* 2012;97(6):e18-e20.
47. Kim D, Park CY, Medeiros BC, Weissman IL. CD19-CD45 low/- CD38 high/CD138+ plasma cells enrich for human tumorigenic myeloma cells. *Leukemia.* 2012;26(12):2530-2537.
48. Kawano Y, Fujiwara S, Wada N, et al. Multiple myeloma cells expressing low levels of CD138 have an immature phenotype and reduced sensitivity to lenalidomide. *Int J Oncol.* 2012;41(3):876-884.
49. Morgan GJ, Walker BA, Davies FE. The genetic architecture of multiple myeloma. *Nat Rev Cancer.* 2012;12(5):335-348.
50. Greaves M, Maley CC. Clonal evolution in cancer. *Nature.* 2012;481(7381):306-313.

From www.bloodjournal.org by guest on November 15, 2015. For personal use only.

blood

2013 122: 3591-3598
doi:10.1182/blood-2013-06-510453 originally published online September 26, 2013

# Detailed characterization of multiple myeloma circulating tumor cells shows unique phenotypic, cytogenetic, functional, and circadian distribution profile

Bruno Paiva, Teresa Paino, Jose-Maria Sayagues, Mercedes Garayoa, Laura San-Segundo, Montserrat Martín, Ines Mota, María-Luz Sanchez, Paloma Bárcena, Irene Aires-Mejia, Luis Corchete, Cristina Jimenez, Ramon Garcia-Sanz, Norma C. Gutierrez, Enrique M. Ocio, Maria-Victoria Mateos, Maria-Belen Vidriales, Alberto Orfao and Jesús F. San Miguel

Updated information and services can be found at:
**http://www.bloodjournal.org/content/122/22/3591.full.html**

Articles on similar topics can be found in the following Blood collections
Lymphoid Neoplasia (2157 articles)
Multiple Myeloma (292 articles)

Information about reproducing this article in parts or in its entirety may be found online at:
**http://www.bloodjournal.org/site/misc/rights.xhtml#repub_requests**

Information about ordering reprints may be found online at:
**http://www.bloodjournal.org/site/misc/rights.xhtml#reprints**

Information about subscriptions and ASH membership may be found online at:
**http://www.bloodjournal.org/site/subscriptions/index.xhtml**

Blood (print ISSN 0006-4971, online ISSN 1528-0020), is published weekly by the American Society of Hematology, 2021 L St, NW, Suite 900, Washington DC 20036.
Copyright 2011 by The American Society of Hematology; all rights reserved.
