# Supplementary material for: Expression of Concern: Prognostic value of circulating plasma cells in patients with multiple myeloma: A meta-analysis
Source: PLoS One. 2023 Feb 21;18(2):e0282230. doi: 10.1371/journal.pone.0282230 (PMC9942954; doi:10.1371/journal.pone.0282230)
Supplement: S1 File — (ZIP) [file pone.0282230.s001.zip › primary data/excluded research/2014 Next-generation sequencing of peripheral B-lineage cells pinpoints the circulating clonotypic cell pool in multiple myeloma.full-B.pdf]

From www.bloodjournal.org by guest on October 21, 2016. For personal use only.

LYMPHOID NEOPLASIA

Next-generation sequencing of peripheral B-lineage cells pinpoints the circulating clonotypic cell pool in multiple myeloma

Benjamin Thiele,[1] Marie Kloster,[1] Malik Alawi,[2] Daniela Indenbirken,[3] Martin Trepel,[1] Adam Grundhoff,[3] and Mascha Binder[1]

[1]University Medical Center Hamburg-Eppendorf, Hubertus Wald Tumorzentrum, University Cancer Center Hamburg, Department of Oncology, Hematology, Bone Marrow Transplantation with Section Pneumology, Hamburg, Germany; [2]University Medical Center Hamburg-Eppendorf, Bioinformatics Service Facility, Hamburg, Germany; and [3]Heinrich-Pette-Institute, Leibniz-Institute for Experimental Virology, Hamburg, Germany

Key Points

- Clonotypic B cells, long suspected to represent circulating stem-like cells, are consistently absent in the blood of myeloma patients.
- Malignant plasma cells frequently circulate in the peripheral blood, show evidence for clonal evolution, and may spread the disease.

The identity of the proliferative compartment of myeloma progenitor cells remains a matter of debate. Polymerase chain reaction-based studies suggested pre-switch "clonotypic" B cells sharing the immunoglobulin (Ig) rearrangement of the malignant plasma cell (M-PC), to circulate in the blood and possess stem cell-like properties. Here, we disprove this hypothesis. We screened peripheral blood IgM, IgG, and IgA repertoires of myeloma patients for the clonotypic rearrangement by next-generation sequencing. None of 12 cases showed pre-switch clonotypic transcripts. In the post-switch IgG/IgA repertoires, however, the clonotypic rearrangement was detected at high frequency in 6 of 8 patients with active disease, whereas it was undetectable after treatment, correlating with flow cytometric presence or absence of circulating M-PCs. Minor subclones with alternative post-switch isotypes suggested ongoing switch events and clonal evolution at the M-PC level. Our findings consistently show an absence of pre-switch clonotypic B cells, while M-PCs circulate in the peripheral blood and may contribute to spreading of the disease. (*Blood*. 2014;123(23):3618-3621)

Introduction

Improvement of long-term outcomes in multiple myeloma critically relies on a better understanding of the tumor-initiating cell in this disease. As in other malignancies, the cancer stem cell concept incriminates drug-resistant myeloma stem cells with tumor-initiating, self-renewing properties to feed the malignant plasma cell (M-PC) compartment in disease relapse and progression.[1,2] However, it remains a matter of controversy if the clonogenic population resides within the pool of terminally differentiated post-switch M-PCs or within a less differentiated (surface) immunoglobulin (Ig)-positive pre-switch B-cell compartment. The latter hypothesis has been fueled by the description of so-called clonotypic pre-switch (IgM^+) B cells postulated to express the same patient-individual variable region Ig rearrangement as the M-PC.[3-7] This finding even provided the rationale for therapeutic targeting of this postulated $CD20^+$ population with the monoclonal antibody rituximab.[8,9] However, inconsistent results of xenotransplantation experiments,[10-15] data on intraclonal evolution at the PC level,[16-22] the analysis of class switch junctions,[23] and the lack of benefit from CD20-directed targeted therapy[8,9] challenged the concept of such feeder cells in myeloma. Moreover, a recent study entirely failed to detect clonotypic rearrangements in highly purified B-cell populations devoid of contaminating M-PCs,[24] and our own data also pointed in this direction, because we could not phenotypically detect clonotypic B cells in the majority of patients with patient-individual Ig ligands as tracers.[25]

Here we used next-generation sequencing to definitively confirm or disprove the existence of this highly controversial cell population.

Materials and methods

Patients and samples

Blood and bone marrow samples of 12 myeloma patients (Table 1) visiting the Freiburg and Hamburg University Medical Centers were obtained after written informed consent as approved by the institutional review boards. This study was conducted in accordance with the Declaration of Helsinki.

Detection and Sanger sequencing of clonotypic Ig rearrangements

M-PC heavy-chain Ig rearrangements were determined as previously described from bone marrow and are shown in supplemental Table 1.[25] For detection of circulating clonotypic cells, peripheral blood mononuclear cells were used. Polymerase chain reaction (PCR) approaches for the qualitative detection of clonotypic rearrangements of all isotypes, of IgM isotype only, and for isotype subclass determination by Sanger sequencing are shown in supplemental Figure 1A-B,D and supplemental Table 2, available on the *Blood* Web site.

NGS of Ig repertoires

Ig transcripts were amplified for next-generation sequencing (NGS) from peripheral blood as described in the supplemental Detailed Methods section.

Submitted February 18, 2014; accepted April 10, 2014. Prepublished online as *Blood* First Edition paper, April 21, 2014; DOI 10.1182/blood-2014-02-556746.

B.T. and M.K. contributed equally to this study.

The online version of this article contains a data supplement.

The publication costs of this article were defrayed in part by page charge payment. Therefore, and solely to indicate this fact, this article is hereby marked "advertisement" in accordance with 18 USC section 1734.

© 2014 by The American Society of Hematology

From www.bloodjournal.org by guest on October 21, 2016. For personal use only.

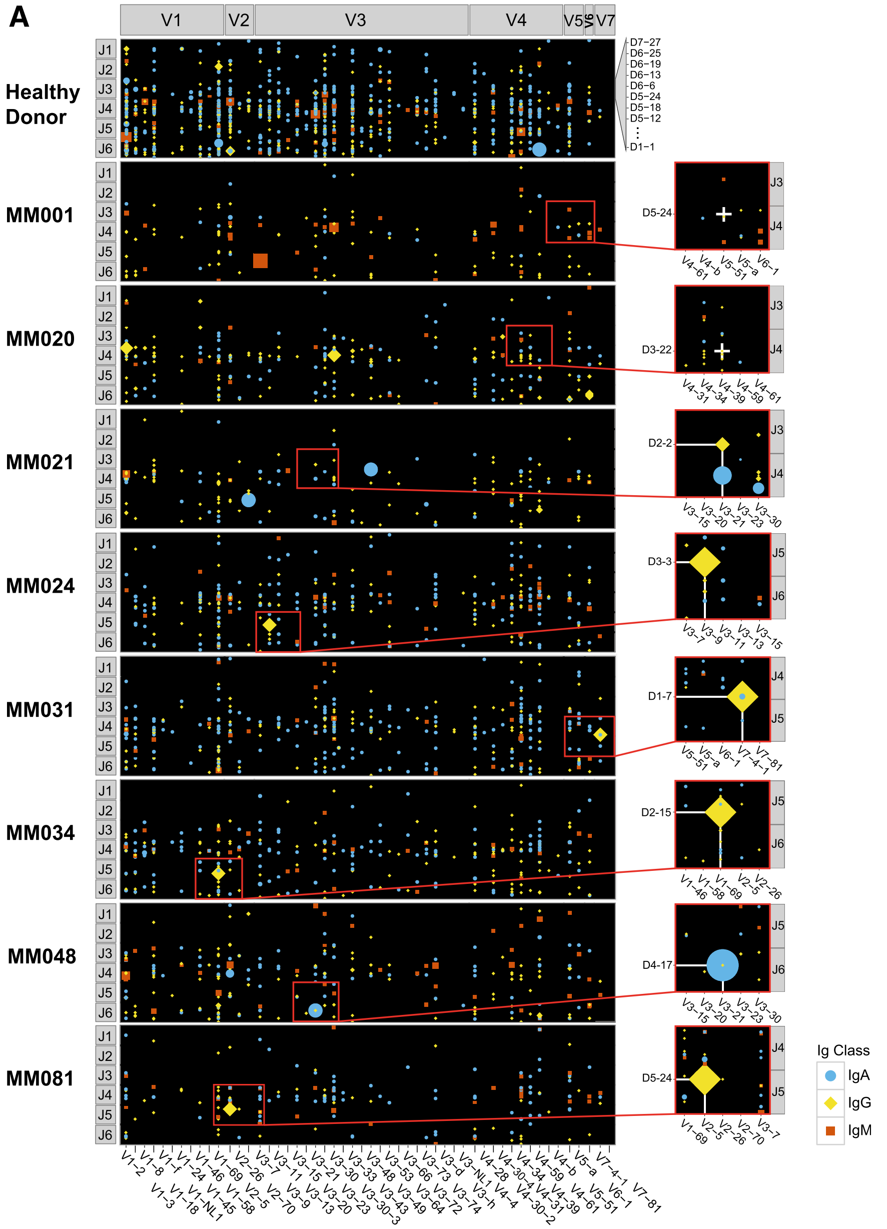

Figure 1. Targeted NGS of heavy-chain Ig repertoires from peripheral B-lineage cells of myeloma patients. (A) Clonotypic rearrangements in the peripheral blood of the myeloma patient cohort. Panel A shows patients MM001 and MM020, who were negative for the clonotypic rearrangement by a qualitative PCR approach. In patients MM021, MM024, MM031, MM034, MM048, and MM081, the clonotypic rearrangement was qualitatively detectable by PCR. For NGS, Ig transcripts were amplified with isotype-specific primers and multiplex sequenced on a MiSeq Illumina sequencer. Data were plotted using ggplot2 for R statistical software assigning a position to each potential V-D-J rearrangement. In patient MM020, a small clone was plotted at the expected site of the clonotypic rearrangement. However, this rearrangement was different from the clonotypic rearrangement (HCDR3 sequence and somatic hypermutation pattern). (B) Absence of clonotypic rearrangements in posttreatment follow-up samples of patients MM024 and MM031. Only IgG repertoires are shown. allo-HSCT, allogeneic hematopoietic stem cell transplant; auto-HSCT, melphalane high-dose chemotherapy followed by autologous stem cell transplant.

The amplification strategy and primer sequences are shown in supplemental Figure 1C and supplemental Table 2. Amplicons were multiplex-sequenced on a MiSeq Illumina sequencer. Data were plotted using ggplot2 for R statistical programming by display on a matrix providing a specific position for each potential V-D-J rearrangement.

Multiparametric flow cytometry

Peripheral blood cells were stained with fluorescently labeled antibodies (CD45-ECD, CD138-PC5, CD38-FITC, CD19-PC7) to discriminate B-lineage cell populations.

Results and discussion

PCR-based detection of clonotypic peripheral blood cells

Peripheral blood clonotypic rearrangements were qualitatively detectable with HCDR3-specific primers in 6 of 8 patients with active disease, suggesting the presence of circulating clonotypic B-lineage cells (data not shown). None of the cases in remission after treatment was PCR-positive. All HCDR3 PCR-positive cases were also positive in an established semi-nested PCR approach using IgM-specific primers. Surprisingly, however, Sanger sequencing of PCR products revealed post-switch IgG/IgA transcripts despite the use of IgM-specific primers, indicating that the PCR may yield false-positive results in the presence of a dominant clonotypic rearrangement of alternative isotype (data not shown).

Targeted NGS of peripheral blood IgM, IgG, and IgA repertoires

This observation prompted us to comprehensively screen Ig repertoires of circulating cells expressing pre- and post-switch isotypes for the presence of the clonotypic rearrangement by NGS. Sequencing data were displayed on a matrix providing a specific position for each potential V-D-J rearrangement (Figure 1). We found highly skewed peripheral Ig repertoires in myeloma patients compared with the polyclonal repertoires of a healthy donor (Figure 1A), reflecting the

From www.bloodjournal.org by guest on October 21, 2016. For personal use only.

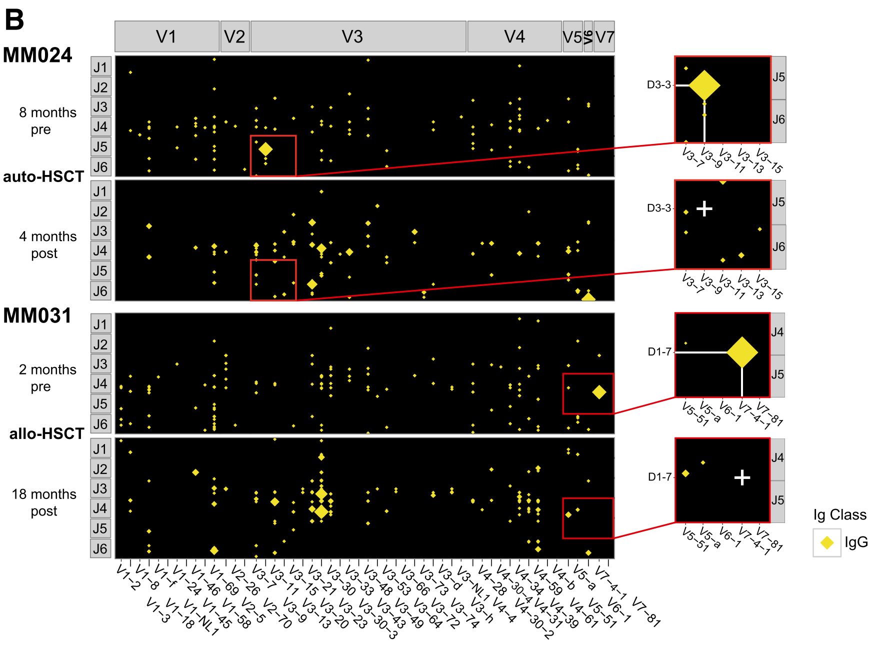

Figure 1. (Continued)

suppression of healthy polyclonal B-lineage cells by the M-PC clone in the bone marrow. None of the patients showed evidence for IgM^+ clonotypic rearrangements, suggesting absence of pre-switch clonotypic B cells in the peripheral blood. The clonotypic rearrangement could, however, be detected within the post-switch repertoire of the M-PC clone (IgG or IgA), indicating that M-PC circulate in the peripheral blood of these patients. In the majority of cases, the clonotypic rearrangement was the most abundant rearrangement detectable within the repertoire with a median of 48% of reads of the respective isotype (supplemental Table 3). PCR-negative control cases MM001 and MM020 (Figure 1A) as well as blood samples of patients MM024 and MM031 in complete remission after treatment (Figure 1B) were negative for the clonotypic rearrangement. In patients MM031 and MM048, we found, apart from the dominant clonotypic rearrangement of the M-PC, an identical rearrangement of alternative post-switch isotype (nonclinical rearrangement). Sequencing of the exact isotype subclass revealed a dominant IgG3 clone with a small IgA1 nonclinical isotype in patient MM031 and a dominant IgA1 clone with a small IgG1 nonclinical isotype in patient MM048. Considering the architecture of the Ig gene locus, this suggested that the MM031 IgG3 clone emerged from the preexisting IgA1 clone, while the small MM048 IgG1 clone had to be considered as precursor of the dominant IgA1 clone. This finding suggested ongoing sequential switch events in the progeny of post-switch myeloma cells and therefore clonal evolution at the PC level.

Flow cytometry-based detection of circulating PCs

To confirm our assumption that the clonotypic post-switch rearrangements may derive from circulating M-PCs, we analyzed peripheral blood cells by multiparametric flow cytometry. We found evidence of circulating M-PCs with the classical immunophenotype in all but one patient with a clonotypic rearrangement in ~1% of leukocytes (supplemental Figure 2A). In the control cases MM001 and MM020 and posttreatment samples of patients MM024 and MM031 (supplemental Figure 2B), no circulating M-PCs could be detected. Patient

Table 1. Clinical features and PCR detection of clonotypic rearrangement in the study cohort of 12 myeloma patients

Patient code	Treatment	Remission status at date(s) of sample acquisition	PCR-positivity for clonotypic rearrangement in peripheral blood
**Patients in remission after treatment**			
MM001	auto-HSCT, Rd	PR	—
MM020	auto-SCT	PR	—
MM023	Vel/Dex	PR	—
MM036	Vel/Dex	PR	—
**Patients with active disease**			
MM032	—	First diagnosis	—
MM050	Rd, Vel/Dex	Relapse	—
MM021	auto-HSCT, Rd, Vel/Dex	Relapse	+
MM024	auto-HSCT, Vel/Dex	Relapse (+ FU sample in CR)	+ (CR sample: −)
MM031	allo-HSCT	Relapse (+ FU samples in CR)	+ (CR sample: −)
MM034	—	First diagnosis	+
MM048	—	First diagnosis	+
MM081	—	First diagnosis	+

allo-HSCT, allogeneic hematopoietic stem cell transplant; auto-HSCT, melphalane high-dose chemotherapy followed by autologous stem cell transplant; CR, complete remission; FU, follow-up; MM, multiple myeloma; PR, partial remission; Rd, Revlimid (Lenalidomide) + Dexamethasone; Vel/Dex, Velcade (Bortezomib) + Dexamethasone.

From www.bloodjournal.org by guest on October 21, 2016. For personal use only.

MM021, who had a low burden of clonotypic rearrangements (only 2% of all IgG reads), was the only NGS-positive case with flow cytometric negativity for circulating M-PCs, most likely due to the different sensitivity levels of these assays.

Discussion of data in the context of previously published work

The hypothesis that pre-switch clonotypic B cells act as tumor-initiating and -propagating cells in myeloma has caused considerable debate for over decades. The results presented here should terminate this controversy. Using highly sensitive and highly specific state-of-the-art technology, we disprove the existence of such cells in the blood. This is in accordance with a recently growing body of evidence by other studies.[8-23]

Two methodological aspects most likely account for the discrepancy between prior reports[3-7] and our study. First, PCR with HCDR3- and isotype-specific primers may lack specificity in the presence of abundant clonotypic transcripts of alternative isotype. Without confirmation by direct sequencing of such transcripts, this finding may have falsely suggested the existence of IgM-positive clonotypic B cells, although the transcripts derived from IgG- or IgA-positive M-PCs. Second, many of the previous studies were primarily based on B-cell subpopulations isolated by fluorescence-activated cell sorting. Because M-PCs can circulate in the peripheral blood, such B-cell populations may easily have been contaminated by circulating M-PCs, which may then have provided the clonotypic sequences falsely attributed to B cells. Consequently, when highly purified B-cell populations devoid of contaminating M-PCs were investigated, no clonotypic transcripts were found.[24]

Taken together, our data show that pre-switch clonotypic B cells are inexistent in the blood of myeloma patients, while circulating M-PCs frequently occur in active disease and may contribute to spreading the disease.

Acknowledgments

This work was supported by the Wilhelm Sander Foundation (grant 2009.035.02 to M.B.).

Authorship

Contribution: B.T., M.K., M.A., and D.I. performed experiments; M.B., B.T., M.K., M.T., and A.G. interpreted data; and M.B., B.T., and M.K. wrote the manuscript.

Conflict-of-interest disclosure: The authors declare no competing financial interests.

Correspondence: Mascha Binder, University Medical Center Hamburg-Eppendorf, Department of Oncology, Hematology, BMT with section Pneumology, Martinistrasse 52, D-20246 Hamburg, Germany; e-mail: m.binder@uke.de.

References

1. Huff CA, Matsui W. Multiple myeloma cancer stem cells. *J Clin Oncol.* 2008;26(17):2895-2900.
2. Hosen N. Multiple myeloma-initiating cells. *Int J Hematol.* 2013;97(3):306-312.
3. Pilarski LM, Masellis-Smith A, Szczepek A, Mant MJ, Belch AR. Circulating clonotypic B cells in the biology of multiple myeloma: speculations on the origin of myeloma. *Leuk Lymphoma.* 1996; 22(5-6):375-383.
4. Billadeau D, Van Ness B, Kimlinger T, et al. Clonal circulating cells are common in plasma cell proliferative disorders: a comparison of monoclonal gammopathy of undetermined significance, smoldering multiple myeloma, and active myeloma. *Blood.* 1996;88(1):289-296.
5. Taylor BJ, Pittman JA, Seeberger K, et al. Intraclonal homogeneity of clonotypic immunoglobulin M and diversity of nonclinical post-switch isotypes in multiple myeloma: insights into the evolution of the myeloma clone. *Clin Cancer Res.* 2002;8(2):502-513.
6. Chen BJ, Epstein J. Circulating clonal lymphocytes in myeloma constitute a minor subpopulation of B cells. *Blood.* 1996;87(5): 1972-1976.
7. Szczepek AJ, Seeberger K, Wizniak J, Mant MJ, Belch AR, Pilarski LM. A high frequency of circulating B cells share clonotypic Ig heavy-chain VDJ rearrangements with autologous bone marrow plasma cells in multiple myeloma, as measured by single-cell and in situ reverse transcriptase-polymerase chain reaction. *Blood.* 1998;92(8):2844-2855.
8. Musto P, Carella AM Jr, Greco MM, et al. Short progression-free survival in myeloma patients receiving rituximab as maintenance therapy after autologous transplantation. *Br J Haematol.* 2003; 123(4):746-747.
9. Zojer N, Kirchbacher K, Vesely M, Hübl W, Ludwig H. Rituximab treatment provides no clinical benefit in patients with pretreated advanced multiple myeloma. *Leuk Lymphoma.* 2006;47(6): 1103-1109.
10. Pilarski LM, Hipperson G, Seeberger K, Pruski E, Coupland RW, Belch AR. Myeloma progenitors in the blood of patients with aggressive or minimal disease: engraftment and self-renewal of primary human myeloma in the bone marrow of NOD SCID mice. *Blood.* 2000;95(3):1056-1065.
11. Pilarski LM, Seeberger K, Coupland RW, et al. Leukemic B cells clonally identical to myeloma plasma cells are myelomagenic in NOD/SCID mice. *Exp Hematol.* 2002;30(3):221-228.
12. Matsui W, Huff CA, Wang Q, et al. Characterization of clonogenic multiple myeloma cells. *Blood.* 2004;103(6):2332-2336.
13. Matsui W, Wang Q, Barber JP, et al. Clonogenic multiple myeloma progenitors, stem cell properties, and drug resistance. *Cancer Res.* 2008;68(1):190-197.
14. Yata K, Yaccoby S. The SCID-rab model: a novel in vivo system for primary human myeloma demonstrating growth of CD138-expressing malignant cells. *Leukemia.* 2004;18(11): 1891-1897.
15. Yaccoby S, Epstein J. The proliferative potential of myeloma plasma cells manifest in the SCID-hu host. *Blood.* 1999;94(10):3576-3582.
16. McSweeney PA, Wells DA, Shults KE, et al. Tumor-specific aneuploidy not detected in CD19+ B-lymphoid cells from myeloma patients in a multidimensional flow cytometric analysis. *Blood.* 1996;88(2):622-632.
17. Pilarski LM, Giannakopoulos NV, Szczepek AJ, Masellis AM, Mant MJ, Belch AR. In multiple myeloma, circulating hyperdiploid B cells have clonotypic immunoglobulin heavy chain rearrangements and may mediate spread of disease. *Clin Cancer Res.* 2000;6(2):585-596.
18. Zojer N, Schuster-Kolbe J, Assmann I, et al. Chromosomal aberrations are shared by malignant plasma cells and a small fraction of circulating CD19+ cells in patients with myeloma and monoclonal gammopathy of undetermined significance. *Br J Haematol.* 2002;117(4):852-859.
19. Santonocito AM, Consoli U, Bagnato S, et al. Flow cytometric detection of aneuploid CD38(++) plasmacells and CD19(+) B-lymphocytes in bone marrow, peripheral blood and PBSC harvest in multiple myeloma patients. *Leuk Res.* 2004;28(5): 469-477.
20. Rasmussen T, Kuehl M, Lodahl M, Johnsen HE, Dahl IM. Possible roles for activating RAS mutations in the MGUS to MM transition and in the intramedullary to extramedullary transition in some plasma cell tumors. *Blood.* 2005;105(1): 317-323.
21. Pfeifer S, Perez-Andres M, Ludwig H, Sahota SS, Zojer N. Evaluating the clonal hierarchy in light-chain multiple myeloma: implications against the myeloma stem cell hypothesis. *Leukemia.* 2011;25(7):1213-1216.
22. Rasmussen T, Haaber J, Dahl IM, et al. Identification of translocation products but not K-RAS mutations in memory B cells from patients with multiple myeloma. *Haematologica.* 2010; 95(10):1730-1737.
23. Taylor BJ, Kriangkum J, Pittman JA, et al. Analysis of clonotypic switch junctions reveals multiple myeloma originates from a single class switch event with ongoing mutation in the isotype-switched progeny. *Blood.* 2008;112(5): 1894-1903.
24. Thiago LS, Perez-Andres M, Balanzategui A, et al. Circulating clonotypic B-cells in multiple myeloma and monoclonal gammopathy of undetermined significance. *Haematologica.* 2014; 99(1):155-162.
25. Trepel M, Martens V, Doll C, et al. Phenotypic detection of clonotypic B cells in multiple myeloma by specific immunoglobulin ligands reveals their rarity in multiple myeloma. *PLoS ONE.* 2012;7(2): e31998.

From www.bloodjournal.org by guest on October 21, 2016. For personal use only.

2014 123: 3618-3621
doi:10.1182/blood-2014-02-556746 originally published online April 21, 2014

Next-generation sequencing of peripheral B-lineage cells pinpoints the circulating clonotypic cell pool in multiple myeloma

Benjamin Thiele, Marie Kloster, Malik Alawi, Daniela Indenbirken, Martin Trepel, Adam Grundhoff and Mascha Binder

Updated information and services can be found at:
http://www.bloodjournal.org/content/123/23/3618.full.html

Articles on similar topics can be found in the following Blood collections
Brief Reports (1905 articles)
Lymphoid Neoplasia (2392 articles)
Multiple Myeloma (347 articles)

Information about reproducing this article in parts or in its entirety may be found online at:
http://www.bloodjournal.org/site/misc/rights.xhtml#repub_requests

Information about ordering reprints may be found online at:
http://www.bloodjournal.org/site/misc/rights.xhtml#reprints

Information about subscriptions and ASH membership may be found online at:
http://www.bloodjournal.org/site/subscriptions/index.xhtml

Blood (print ISSN 0006-4971, online ISSN 1528-0020), is published weekly by the American Society of Hematology, 2021 L St, NW, Suite 900, Washington DC 20036.
Copyright 2011 by The American Society of Hematology; all rights reserved.
