# Supplementary material for: Expression of Concern: Prognostic value of circulating plasma cells in patients with multiple myeloma: A meta-analysis
Source: PLoS One. 2023 Feb 21;18(2):e0282230. doi: 10.1371/journal.pone.0282230 (PMC9942954; doi:10.1371/journal.pone.0282230)
Supplement: S1 File — (ZIP) [file pone.0282230.s001.zip › primary data/excluded research/2015 Clinical Significance of Circulating Plasma Cells in Myeloma Multiparameter Flow Cytometric Analysis, PC.pdf]

HomeDecember 3, 2015; Blood: 126 (23)

Clinical Significance of Circulating Plasma Cells in Myeloma: Multiparameter Flow Cytometric Analysis

Manabu Fujisawa, MD*,[1], Yasuhito Suehara, MD*,[1], Kota Fukumoto, MD*,[1], Kentaro Narita, MD*,[2], Yoshiaki Usui, MD*,[1], Masami Takeuchi, MD*,[1], Kosei Matsue, MDPhD[3], and Hiroyuki Takamatsu, MD PhD[4]

Author Affiliations

Article Figures & Data Info & Metrics E-Letters PDF

Abstract

[Introduction] It has been reported that clonal circulating plasma cells (cPC) were detected in patients with multiple myeloma (MM) and other plasma cell dyscrasias (PCD), and their detection has been shown to be a risk in newly diagnosed MM and shortened progression free survival in patients smoldering MM (sMM). It is unclear that the detection of cPCs in peripheral blood may be a simple reflection of tumor burden of bone marrow plasma cells or may represent different. This study was performed to evaluate the clinical utility of quantifying clonal cPC using 6-color FCM in patients with MGUS, SMM, and symptomatic MM (symMM) at diagnosis (Dx). They were compared with bone marrow samples (BM PCs) simulatneously analyzed in the same way. Besides we evaluated the transition of quantification of clonal cPC of PB samples at various stages after treatment.

[Materials and Methods] We analyzed 280 peripheral blood (PB) samples from 194 patients with PCD (MGUS, SMM, and symMM; *n* = 34/39/121, respectively) diagnosed at the Department of Hematology/Oncology, Kameda Medical Center and Kanazawa University Hospital from January 2012 to July 2015. Similarly, we analyzed 30 PB samples from 30 healthy controls. Mononuclear cells (MNC) were isolated from PB by density gradient centrifugation and stained with antibodies to CD45, CD38, CD19, CD138, CD56, and cytoplasmic kappa and lambda immunoglobulin (Ig) light chains. The cells were collected using a Beckman-Coulter NAVIOS and analyzed using Kaluza software. The gating strategy employed was expression of CD38, CD45, CD138, and cytoplasmic Ig light chains. Clonal cPC was defined as the population of $CD138^{+}$, $CD38^{+}CD45^{-\sim\pm}$, and $CD19^{-}$. To confirm the clonality of these cells, light chain restriction of cytoplasmic Ig was used (κ/λ>4 or <0.5) using the 2nd tube. Quantification was performed by acquiring > 2×10^{6} total events. At least ≥ 20 clonal cPC were required for the analysis.

[Results] We analyzed clonal cPC of 194 PB samples with MGUS, SMM, and symMM (*n* = 34/39/121, respectively) at diagnosis (Dx). The mean values of clonal cPC were: MGUS, 2.67×10^{-5}/MNC (range, 0 - 3.0×10^{-4}); SMM, 2.43×10^{-4}/MNC (range, 0 - 3.6×10^{-3}); symMM, 6.99×10^{-3}/MNC (range, 0 - 1.5×10^{-1}). The number of clonal cPC was significantly higher in SMM than MGUS, and in symMM than SMM (P < 0.001). In healthy controls, the mean value of cPC without CD19 expression was 3.84×10^{-6}/MNC (range, 0 - 1.3×10^{-5}). Although these cPC lacked the expression of CD19, none of the samples did not show the light chain restriction of cytoplasmic Ig. Next, we analyzed clonal cPC of 185 PB samples (Dx, *n* = 85; partial response [PR], *n* = 31; very good partial response [VGPR], *n* = 25; complete response [CR], *n* = 22; progressive disease [PD], *n* = 23) in various treatment responses of symMM. The mean values of clonal cPC were: at Dx, 6.99×10^{-3}/MNC (range, 0 - 1.5×10^{-1}); at PR, 5.19×10^{-5}/MNC (range, 0 - 3.5×10^{-4}); at VGPR, 9.79×10^{-5}/MNC (range, 0 - 9.7×10^{-4}); at CR, 1.90×10^{-5}/MNC (range, 0 - 1.2×10^{-4}); at PD, 1.94×10^{-3}/MNC (range, 0 - 3.9×10^{-2}). The number of clonal cPC was significantly higher at Dx than from PR to CR, and significantly lower in CR than from PR to VGPR (P < 0.01, P = 0.23,

respectively). However, the number of clonal cPC was not significantly different between PR and VGPR (*P* = 0.61). When patients with PCD at Dx were divided into high and low clonal cPC groups (high cPC ≥ $1.0x10^{-4}$/MNC and low cPC < 1.0x10-3/MNC), the number of the high cPC group was as follows: (MGUS 2/35 (5.7%), SMM 9/38 (23.7%), symMM 35/85 (41.2%)) (P<0.01). In symMM patients, clinical characteristics were comparable between the two groups except for bone marrow PCs. A total of 133 bone marrow samples (BM PCs) were measured simultaneously with PB samples at Dx. The number of clonal cPC tended to be related to the percentage of clonal plasma cells/total plasma cells in BM in symMM (R = 0.579), but no such relation was observed in MGUS and SMM (R = 0.112, -0.03).

[Conclusions] In conclusion, quantification of clonal cPC by 6-color FCM is feasible and appears to reflect the tumor burden in patients with newly diagnosed MM and sMM, but not in patients after treatment. Further studies using quantification of clonal cPC combined with other hematological parameters will identify patients at high risk of early progression and poor prognosis.

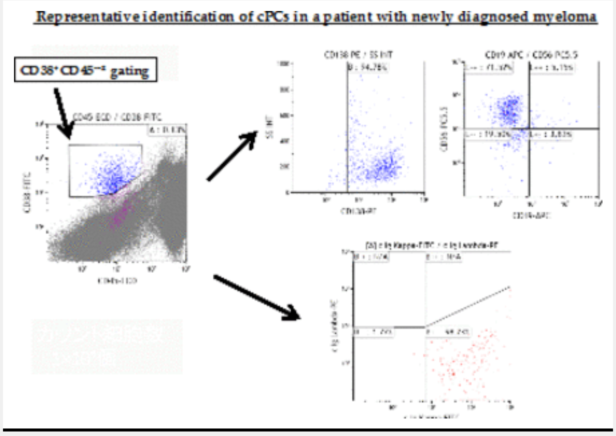

Figure 1. Download figureOpen in new tabDownload powerpoint

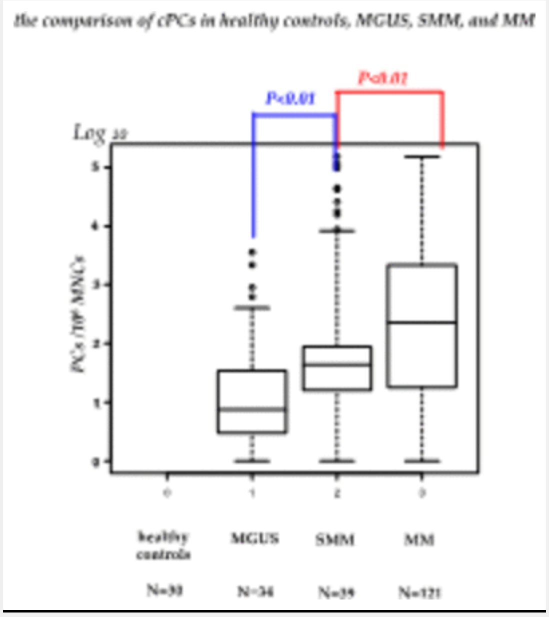

Figure 2. Download figureOpen in new tabDownload powerpoint

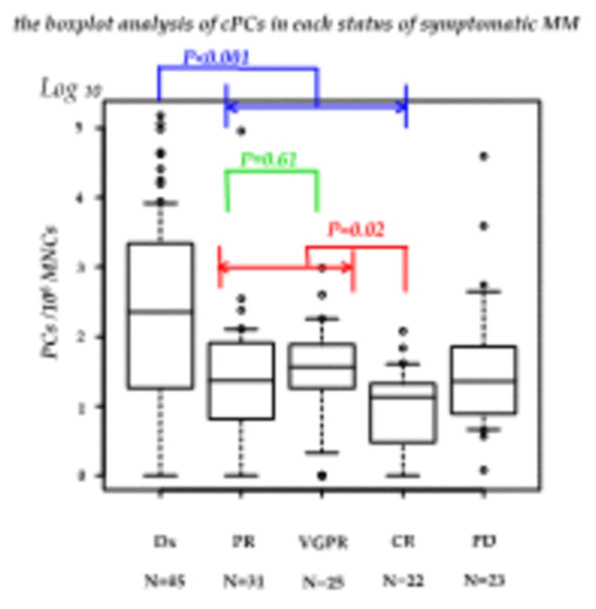

Figure 3. Download figureOpen in new tabDownload powerpoint

Disclosures No relevant conflicts of interest to declare.

- ↵* Asterisk with author names denotes non-ASH members.
