# Supplementary material for: Expression of Concern: Prognostic value of circulating plasma cells in patients with multiple myeloma: A meta-analysis
Source: PLoS One. 2023 Feb 21;18(2):e0282230. doi: 10.1371/journal.pone.0282230 (PMC9942954; doi:10.1371/journal.pone.0282230)
Supplement: S1 File — (ZIP) [file pone.0282230.s001.zip › primary data/excluded research/2016 A CD138-independent strategy to detect minimal residual disease and circulating tumour cells in multiple myeloma-cpc.pdf]

A CD138-independent strategy to detect minimal residual disease and circulating tumour cells in multiple myeloma

Barbara Muz,[1] Pilar de la Puente,[1] Feda Azab,[1] Micah John Luderer,[1] Justin King,[2] Ravi Vij [2] and Abdel Kareem Azab[1]

[1] Department of Radiation Oncology, Cancer Biology Division, Washington University School of Medicine, and [2]Section of Stem Cell Transplant and Leukemia, Division of Medical Oncology, Washington University School of Medicine, St. Louis, MO, USA

Received 10 August 2015; accepted for publication 18 November 2015

Correspondence: Abdel Kareem Azab, Ph.D., B. Pharm., Department of Radiation Oncology, Cancer Biology Division, Washington University in Saint Louis School of Medicine, 4511 Forest Park Ave., Room 3103, St. Louis, MO 63108, USA.
E-mail: aazab@radonc.wustl.edu

Summary

CD138 (also termed SDC1) has been the gold-standard surface marker to detect multiple myeloma (MM) cells for decades; however, drug-resistant residual and circulating MM cells were shown to have lower expression of this marker. In this study, we have shown that residual MM cells following bortezomib treatment are hypoxic. This combination of drug exposure and hypoxia down-regulates their CD138 expression, thereby making this marker unsuitable for detecting residual or other hypoxic MM cells, such as circulating tumour cells, in MM. Hence, we developed an alternative biomarker set which detects myeloma cells independent of their hypoxic and CD138 expression status *in vitro*, *in vivo* and in primary MM patients. The new markers were able to identify a clonal CD138-negative population as minimal residual disease in the bone marrow and peripheral blood of MM patients. Further investigation to characterize the role of this population as a prognostic marker in MM is warranted.

Keywords: multiple myeloma, cell surface antigens, diagnostic haematology, minimal residual disease, flow cytometry, haematological malignancy.

Multiple myeloma (MM) is the second most common haematological malignancy and represents approximately 20% of deaths from haematological malignancies (Kyle *et al*, 2003; Ludwig, 2005). Elucidating the molecular mechanisms of cell signalling pathways in MM cells and their interaction with the bone marrow (BM) microenvironment has led to the development of novel therapies (Nair *et al*, 2012; de la Puente & Azab, 2013). The survival of MM patients has improved significantly in the past decade due to the introduction of these novel therapies, including immunomodulatory drugs and proteasome inhibitors (Mitsiades *et al*, 2007; Kumar *et al*, 2008).

Flow cytometry is the leading technology for the detection of MM cells (Paiva *et al*, 2008; Rawstron *et al*, 2013) and the acceptable method is based on CD138+CD38+ for the primary definition of plasma cells, enhanced by various combinations of markers such as CD19-/CD45-/CD56+ or CD27-/CD81-/CD20+/CD28+/CD117+/CD200+ (San Miguel *et al*, 2006; Rawstron *et al*, 2008). In addition to flow cytometry, polymerase chain reaction (PCR)-based approaches are used to detect clonal cells as minimal residual disease (MRD) in MM, and demonstrate equivalent ability to detect MRD as compared to flow cytometry (Puig *et al*, 2014). However, two critical issues remain unresolved for the PCR approach: the inability to obtain successful primers in up to a third of patients, and the absolute requirement for a baseline sample (Martinez-Sanchez *et al*, 2008; Ladetto *et al*, 2014). Flow cytometry remains the method of choice due to its widespread accessibility in most haematology laboratories and its increasing ability to interrogate millions of cells in a short time. However, there is still a lack of uniformity in the specific biomarker set chosen to detect MRD, which has hampered the success of this technique (Flanders *et al*, 2013). Therefore, a new set of biomarkers for the detection of MM cells by flow cytometry is warranted.

In MM, circulating tumour cells (CTCs) are considered an unfavourable prognostic factor and indicate an aggressive form of the disease (Paiva *et al*, 2013; An *et al*, 2015), and therefore detecting CTCs can be used as a powerful prognostic tool in this disorder (Peceliunas *et al*, 2012). In the clinical setting, CTCs in MM patients are detected using flow cytometry with CD138 (also termed SDC1) as the principle marker (Peceliunas *et al*, 2012; Paiva *et al*, 2013; An *et al*, 2015); however, recent studies suggest the presence of circulating clonal B-cells in MM that do not express CD138 (Thiago *et al*, 2014). In agreement with these findings, CTCs in

First published online 5 January 2016
doi: 10.1111/bjh.13927

© 2016 John Wiley & Sons Ltd

MM have different biological characteristics compared to MM cells in the BM, in which the expression of several adhesion molecules, including CD138 was downregulated on CTCs (Paiva *et al*, 2013). Previous studies have also shown that shedding of CD138 is mostly prevalent in circulating MM cells (Seidel *et al*, 2000). These findings suggest that CD138+/- status is not a reliable method for the detection of CTCs in MM and an alternative biomarker strategy is needed.

We have previously shown that hypoxia is a general feature of haematological malignancies, including MM (Azab *et al*, 2012a, 2013). Due to rapid tumour development, the oxygenation level in the BM is decreased, driving MM cells to egress from the primary tumour site and metastasize to a new BM niche. In addition, all CTCs exhibited a hypoxic phenotype (Azab *et al*, 2012a). Moreover, it was demonstrated that hypoxia induces overexpression of heparanase, which is responsible for CD138 shedding in cancer cells (Almeida *et al*, 1999; He *et al*, 2004; Wu *et al*, 2010; Peceliunas *et al*, 2012).

In this study, we developed a new set of biomarkers to detect MM cells, both in the BM and in the circulation of MM patients independent of their CD138 expression using a two-colour flow cytometry. We found that most MRD and CTCs did not express CD138, but were detectable with the new method. In addition, we assessed this new method for the detection of MRD in the BM as well as CTCs in order to predict time-to-progression in MM patients and compared it to other CD138-based methods and histology.

Materials and methods

Cell culture

The MM cell lines (MM1s, MM1r, OPM1, OPM2, H929, RPMI8226, U266 and MM1s-GFP-Luc; mycoplasma-negative) were a kind gift from Dr. Irene Ghobrial (Dana-Farber Cancer Institute, Boston, MA, USA). Cells were cultured in RPMI-1640 medium (Corning CellGro, Mediatech, Manassas, VA, USA), enriched with 10% fetal bovine serum (Gibco, Life Technologies, Grand Island, NY, USA), 2 mmol/l of L-glutamine, 100 u/ml Penicillin and 100 μg/ml Streptomycin (CellGro, Mediatech). Cells were incubated at 37°C under normoxic (21% O_2, NuAire water jacket incubator, Plymouth, MN, USA) or hypoxic conditions (1% O_2; Coy, Grass Lake, MI) for 24 or 48 h.

Patient samples

Bone marrow (BM) aspirates and peripheral blood (PB) samples from MM or healthy patients were obtained from the Siteman Cancer Center, Washington University in Saint Louis, MO. PB mononuclear cells (PBMCs) and BM mononuclear cells (BMMCs) from MM patients were isolated using 1x red blood cell lysis buffer according to the manufacturer's protocol (BioLegend, San Diego, CA, USA). Informed consent was obtained from all patients with an approval from the Washington University in St. Louis School of Medicine Institutional Review Board committee and in accordance with the Declaration of Helsinki.

Creating a MRD model to test the hypoxic status of MM cells and CD138 and CD38 expression in vivo *and* in vitro

MM1s-GFP-Luc cells were injected into six female, 7-week-old severe combined immunodeficient (SCID) mice (Taconic Farms, Hudson, NY, USA) intravenously (IV) at the concentration of 2×10^6 cells per mouse and tumour progression was evaluated using bioluminescent imaging (BLI). Approval for these studies was obtained from the Ethical Committee for Animal Experiments at Washington University in St. Louis School of Medicine. We developed a MRD model by treating half of the MM-bearing mice ($n = 3$ per group chosen randomly; no blinding of the investigator was necessary) with no or high-dose bortezomib (1·5 mg/kg twice a week intraperitoneally (IP); Selleck Chem, Houston, TX, USA) until no (or minimal) BLI signal was detected. Next, pimonidazole (PIM; Hypoxyprobe Store, Burlington, MA, USA) at the concentration of 100 mg/kg was injected IP, and after 4 h, mice were sacrificed and the femurs were harvested. BMMCs were isolated from the BM, fixed, permeabilized and stained with anti-PIM-allophycocyanin (APC) (Hypoxyprobe Store), anti-CD138-V450 and anti-CD38-fluorescein isothiocyanate (FITC) antibodies (BD Biosciences, San Jose, CA, USA) on ice for 1 h, washed and analysed by flow cytometry using MACSQuant Flow Cytometry (Miltenyi Biotech, San Diego, CA, USA). MM cells were detected by flow cytometry by gating green fluorescent protein (GFP)-positive cells, and hypoxia in these cells was measured as mean-fluorescence intensity (MFI) of PIM-APC signal. APC^{high} (hypoxic) and APC^{low} (normoxic) cells were analysed for CD138 and CD38 expression, normalized to respective isotype controls and the result was averaged from six mice.

For the *in vitro* study, five MM cell lines (MM1s, OPM1, H929, RPMI8226 and U266) were exposed to normoxia or hypoxia for 24 and 48 h, 1×10^6 cells were stained with anti-CD38-APC and anti-CD138-V450 antibodies. Change of expression was calculated as a ratio between MFI values in hypoxia and normoxia, and averaged from 5 cell lines. MM cells lines (RPMI8226, H929 and OPM2) were also treated with 5 nmol/l bortezomib for 24 h, and the CD38 and CD138 expression study was performed as above.

Development and validation of the new strategy to detect MM cells

PBMCs and MM cells lines (MM1s, U266, OPM1, H929) were stained with antibodies detecting CD38-APC,

© 2016 John Wiley & Sons Ltd

CD3-FITC, CD14-FITC, CD16-FITC, CD19-FITC and CD123-FITC (BD Biosciences), and the percentage of cell sub-populations was identified by these specific markers. Next, MM cell lines (MM1s, OPM1, H929, RPMI8226 and U266) were exposed to normoxia and hypoxia for 24 and 48 h and the expression of CD3, CD14, CD16, CD19 and CD123 was evaluated and depicted as the average from 5 cell lines. To corroborate the detection ability of the new method, OPM1 cells were first labelled with calcein red-orange (Invitrogen, Carlsbad, CA, USA), cultured in normoxia or hypoxia for 24 h, then 10% were spiked into healthy BMMCs and stained with the antibody-cocktail: anti-CD38-APC and anti-(CD3, CD14, CD16, CD19 and CD123)-FITC, as well as anti-CD38-APC alone and anti-CD138-V450 alone; and the percentage of OPM1 cells was detected by flow cytometry.

To validate this method *in vivo*, we tested the ability of the new method to detect MM cells in mice with increasing tumour burden and thus increasing hypoxic state. 7 SCID mice were injected with different concentrations of MM1s-GFP-Luc cells, which were allowed to grow for 5 weeks. BMMCs were isolated from femurs and stained with anti-CD138-Peridinin Chlorophyll Protein-Cyanin 5·5 (PerCP-Cy5·5) or with the antibody-cocktail: anti-CD38-APC and anti-(CD3, CD14, CD16, CD19 and CD123)-Brilliant Violet 421 (BV421; BD Biosciences). MM cells were detected using the two methods and compared to the amount of MM cells detected by the intrinsic marker GFP.

Detection of MM cells in patients' BM using the new strategy and confirming MM cell clonality

Bone marrow from 20 MM patients (10 kappa and 10 lambda) were depleted from CD138 using magnetic bead selection (Miltenyi Biotech) and stained with the antibody-cocktail: anti-CD38-APC and anti-(CD3, CD14, CD16, CD19 and CD123)-FITC to detect MM cells. To assess clonality, the cells were first fixed with formalin, stained with extracellular markers (the antibody-cocktail), washed and permeabilized with 0·5% Tween/1x phosphate-buffered saline, followed by staining with intracellular markers: light chains kappa-PerCP-Cy5·5 and lambda-PE antibodies (BD Biosciences), and analysed by flow cytometry. We also tested the correlation between tumour burden (percentage of MM cells detected by the new method) and the clonality ratio, as well as the percentage of non-clonal (NC), double negative MM cells.

Detection of MM cells in peripheral blood using the new strategy

To corroborate the detection ability of the new method *in vitro*, H929 cells were first labelled with calcein red-orange, cultured in normoxia or hypoxia for 24 h, and then 1% of the H929 cells were spiked into healthy PBMCs. The cells were stained with the antibody-cocktail: anti-CD38-APC and anti-(CD3, CD14, CD16, CD19 and CD123)-FITC or with anti-CD138-V450 alone and were detected by flow cytometry.

In vivo, blood was harvested from MM-bearing mice ($n = 5$), PBMCs were isolated and stained with the antibody-cocktail: anti-CD38-APC and anti-(CD3, CD14, CD16, CD19 and CD123)-BV421, as well as anti-CD138-PerCP-Cy5·5 antibody. MM cells were detected using the two methods and compared to the amounts of cells detected by the intrinsic marker GFP.

Moreover, peripheral blood from 20 MM patients was collected; PBMCs were isolated and stained with either CD138 alone or the antibody-cocktail, and the number of MM cells was detected by flow cytometry.

Statistical analysis

Experiments were performed in quintuplicates and repeated at least three times. Results were presented as mean ± standard deviation, and statistical significance was analysed using Student *t*-test for independence for statistical significance, with the data meeting the assumption of the tests such as normal distribution. Values were considered significantly different for *P* value less than 0·05.

Results

MRD cells are hypoxic with decreased expression of CD138 but unchanged expression of CD38

To test the role of hypoxia in the development of residual drug resistant MM cells, we developed a MRD model by treating MM-bearing mice with bortezomib which resulted in a tumour burden of about 1% of MM cells in the BM (Fig 1A). Bortezomib-treated MRD drug resistant cells were 3·5-fold more hypoxic than the MM cells isolated from vehicle-treated mice as shown by PIM signal (Fig 1B). We further found that CD138 expression was significantly decreased in hypoxic cells (Fig 1C), but CD38 expression was unchanged (Fig 1D). Next, we determined the *in vitro* expression of CD38 and CD138 in five MM cells lines in hypoxia, and found that the average CD138 expression was significantly decreased by half in hypoxia, whereas CD38 expression was unchanged (Fig 1E). Moreover, bortezomib treatment did not alter CD38 expression, but significantly decreased CD138 expression in MM cell lines (Fig 1F).

Development of the CD138-independent strategy to detect normoxic and hypoxic MM cells

In contrast to CD138, CD38 expression was unchanged under hypoxia or following bortezomib treatment; therefore we developed a new method to detect MM cells utilizing the stably expressed CD38. The new method, using an antibody cocktail, defines the MM cell population, as all CD38+ cells

© 2016 John Wiley & Sons Ltd

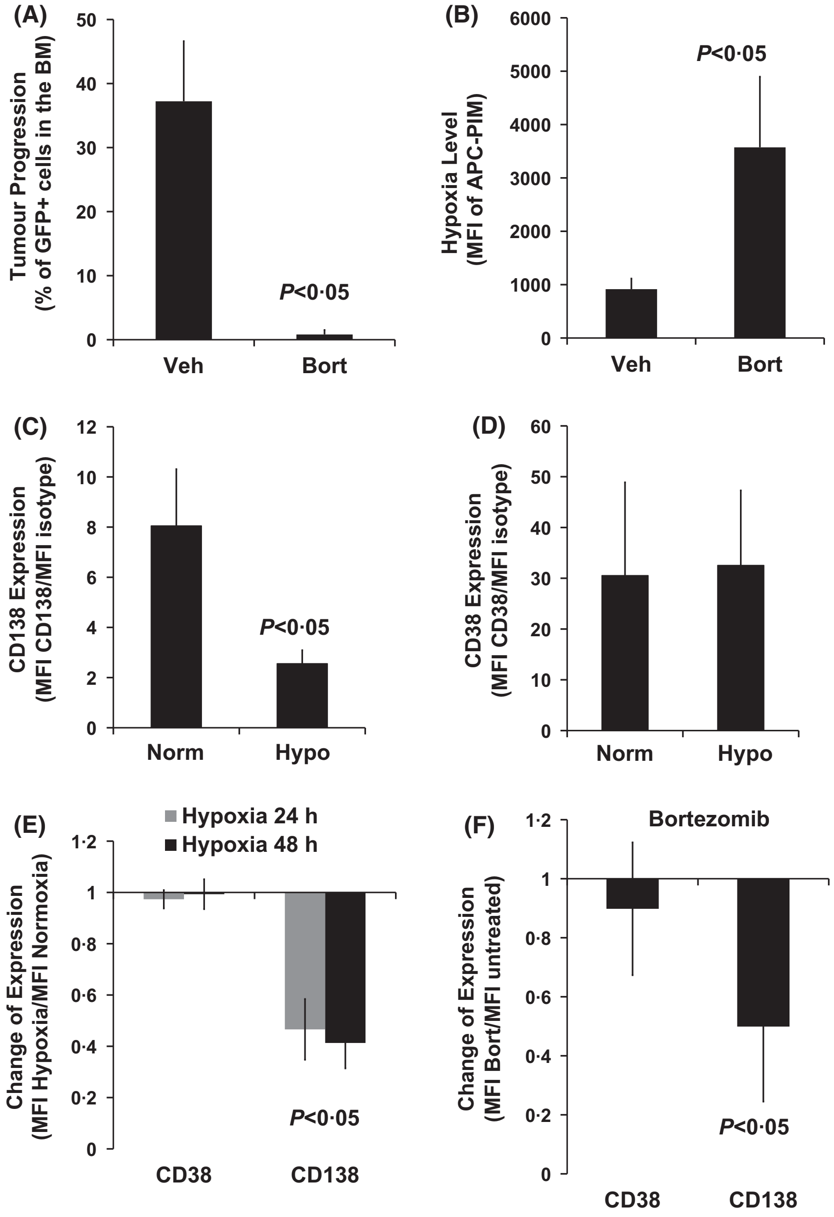

Fig 1. Creating a minimal residual disease model to test hypoxic status of multiple myeloma cells and the expression of CD138 and CD38. (A) Tumour progression based on detecting MM1s-GFP+ cells in the bone marrow from mice treated with vehicle (veh) and 1·5 mg/kg bortezomib (Bort). (B) Hypoxia level shown as mean fluorescence intensity (MFI) of anti-Pimonidazole (PIM) antibody conjugated with allophycocyanin (APC) detected in GFP+ cells from mice treated with vehicle and bortezomib. (C, D) CD138 and CD38 expression in GFP+ cells with PIM^{low} (normoxic, Norm) and PIM^{high} (hypoxic, Hypo) signal depicted as a ratio between MFI of a CD marker and MFI of a respective isotype control shown as mean ± standard deviation (SD) from three mice. (E) Expression of CD38 and CD138 in MM cells exposed to hypoxia (1% O_2) for 24 and 48 h, and normalized to normoxia (21% O_2), shown as a mean ± SD from 5 MM cell lines. (F) Expression of CD38 and CD138 in MM cells exposed to 5 nmol/l bortezomib for 24 h, normalized to untreated cells, shown as a mean ± SD from 3 MM cell lines (performed in triplicates). Student two-sided *t*-test was used to calculate the statistical significance.

(Fig 2A, red box) after the exclusion of all non-myeloma-CD38+ cells by utilizing CD3+ antibody to eliminate T cells, CD14+ to eliminate monocytes and macrophages, CD16+ to eliminate NK cells, eosinophils and neutrophils, CD19+ to eliminate B cells and CD123+ to eliminate dendritic cells and basophils (Fig 2A, purple box).

To evaluate our method for defining the MM cell population, PBMCs from healthy donors were used as control samples. Healthy donors were found to contain approximately 20% of CD38+ cells, 60% of CD3+ cells, 10% each of CD14+, CD16+ and CD19+ cells, and 5% of CD123+ cells. In comparison, all of the MM cell lines were CD38+ but did not express CD3, CD14, CD16, CD19 or CD123 (Fig 2B); and the expression of these markers did not change in hypoxia (Fig 2C).

Next, the sensitivity of traditional method based on CD138 expression was compared to the new method in order to detect OPM1 cells spiked at a ratio of 10% into BMMCs. Using only the CD138 marker, 8% of the normoxic and 5% of the hypoxic MM cells were detected (Fig 2D). Clearly this indicates that gating only for CD138+ cells fails to detect 20% and 50% of the spiked MM cell population under normoxic and hypoxic conditions, respectively. In comparison, using only CD38 as a marker detected about 25% of cells when spiking OPM1 cells under normoxia or hypoxia. However, applying the exclusion criteria indicated that the 25% of detected CD38+ cells consists of 15% CD38+ but non-MM cells (contamination) in addition to the 10% spiked OPM1 cells. Therefore, the new method accurately excluded 15% of CD38+ non-MM cells and correctly detected the

© 2016 John Wiley & Sons Ltd

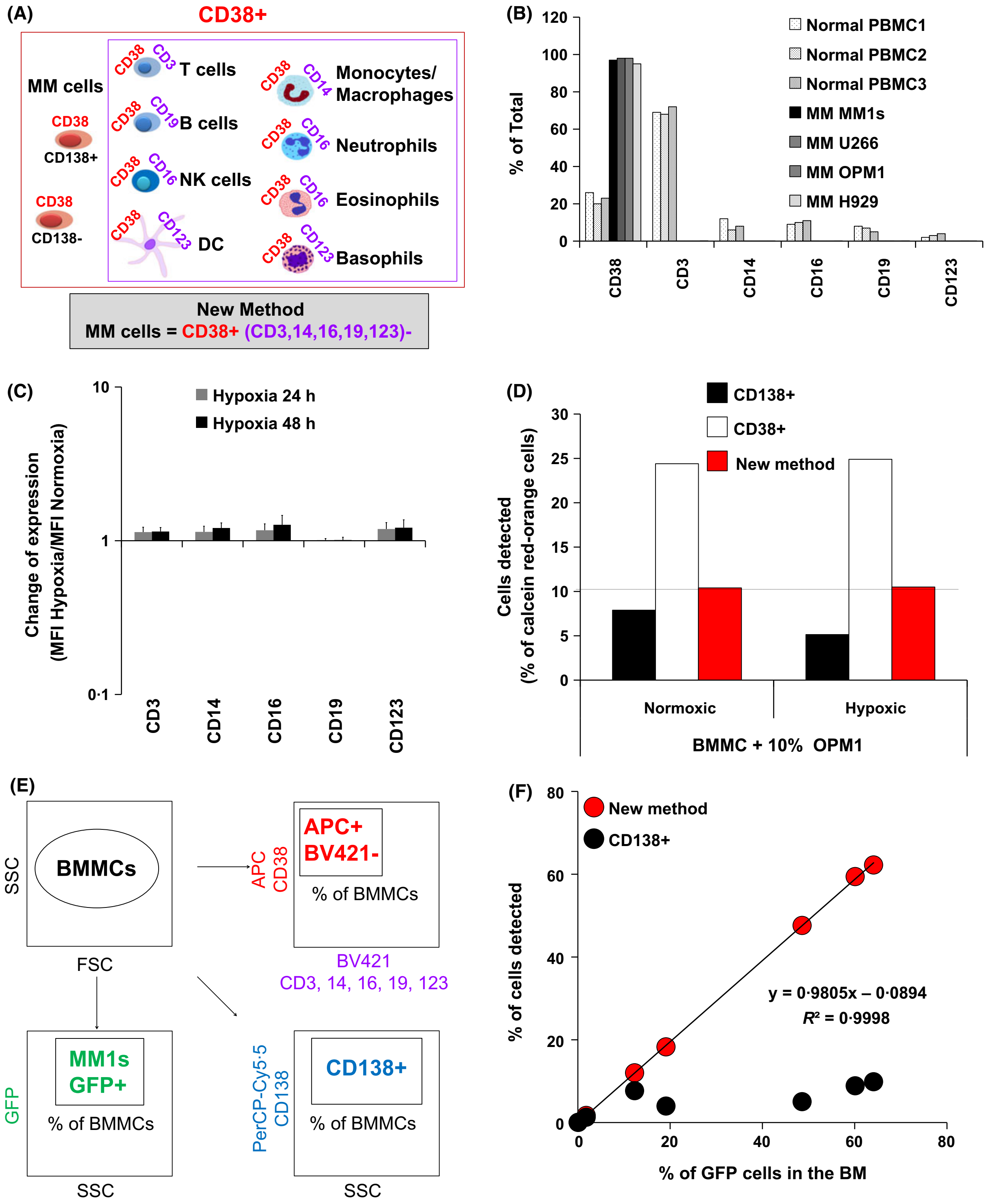

Fig 2. Development and validation of the new strategy to detect MM cells. (A) The methodology behind the new strategy to detect multiple myeloma (MM) cells. (B) The percentage of cells positive for CD38, CD3, CD14, CD16, CD19 and CD123 in the normal PBMCs from three healthy donors and 4 MM cell lines detected by flow cytometry. (C) Expression of CD3, CD14, CD16, CD19 and CD123 in MM cells exposed to hypoxia (1% O_2) for 24 and 48 h, and normalized to normoxia (21% O_2), shown as a mean ± SD from 5 MM cell lines. (D) Detection of normoxic and hypoxic calcein red-orange+ OPM1 cells spiked into BMMCs detected by flow cytometry using CD138, CD38 and the new method. (E) The experimental approach of detecting MM cells in the BMMCs from MM-bearing mice using three strategies: GFP+, CD138 + and the new method. (F) Percentage of MM cells identified by CD138 + or the new method and plotted against the percentage of GFP+ cells in the BM from mice with different tumour size. GFP, green fluorescent protein; DC, dendritic cells; BMMCs, bone marrow mononuclear cells; PBMCs, peripheral blood mononuclear cells; SSC, side scatter; FSC, forward scatter.

© 2016 John Wiley & Sons Ltd

exact 10% of spiked MM cells independent of their normoxic or hypoxic status.

We then tested the ability of CD138 marker or the new method to detect MM cells in SCID mice with different tumour burden (% of MM1s-GFP+ cells) and compared the results achieved by the intrinsic GFP marker (Fig 2E). MM cell detection using GFP marker showed different tumour involvement of 0%, 2%, 12%, 18%, 48%, 60% and 62% of GFP+ cells in the BM. The CD138+ gating detected only a fraction of the MM1s-GFP+ population, less than 10%, even in mice with the highest BM tumour burden (Fig 2F). Strikingly, the new method detected a near identical percentage of MM cells in the BM consistent with the percentage of GFP+ cells with a linear correlation coefficient of $R^2 = 0{\cdot}9998$ and the slope of the correlation close to 1 (0·9805).

The new method identifies clonal CD138-negative MM cells in patients

We then tested the ability of the new method to detect MRD cells in the CD138-depleted BM samples and confirmed the clonality of the APC+FITC- population according to the schematic [Fig 3 A(i)], which was first tested in kappa-positive [Fig 3A (ii)] and lambda-positive patients [Fig 3 A(iii)]. Using the new method, we detected MM cells in all 20 patient samples, with BM involvement of MM cells varying between 0·1% (Patient 9) to 37·9% (Patient 16) (Fig 3B). Moreover, to ensure the clonal nature of these populations, the clonality of these patients was confirmed by calculating the kappa/lambda ratio of APC+FITC- cells. The physiological (polyclonal) ratio in healthy subjects ranges between 0·76 and 1·5, whereas values above or below this ratio indicate clonal proliferation of plasma cells (Nakayama *et al*, 2012). We found that 16 of 20 patients' myeloma cells were clonal and matched the clonality of the original disease (CD138+ cells). The additional four patients (Patient 9, 12, 13 and 17) who had a low percentile of MM cells detected by the new method (0·1%, 0·2%, 0·52% and 0·32%, respectively) had polyclonal plasma cells with the normal clonality ratio (Fig 3C). In addition, the kappa/lambda clonality ratio was in indirect [for lambda patients; Fig 3D (i)] and in direct [for kappa patients; Fig 3D (ii)] correlation with the detected tumour burden. Furthermore, the level of non-clonal contamination (kappa-negative and lambda-negative) in the population detected by the new method was less than 0·69% in all the samples (Fig 3E).

The new method detects CTCs in peripheral blood

To compare the sensitivity of traditional CD138-based detection strategy to the new method in detecting circulating cells, we first tested *in vitro* detection of calcein red-orange-positive H929 cells cultured in normoxia and hypoxia spiked at a ratio of 1% into healthy PBMCs. We showed that the CD138 marker detected 0·95% of normoxic MM cells and only 0·45% of hypoxic cells. On the other hand, the new method detected close to 1% of the spiked population of either normoxic or hypoxic MM cells (Fig 4A). This is consistent with the new method detecting 10% of normoxic and hypoxic OPM1 cells spiked into BMMCs (Fig 2D).

In vivo, we tested the ability of CD138 or the new method to detect circulating MM1s-GFP+ cells in MM-bearing mice (Fig 4B). The CD138 marker failed to detect more than 98% of the circulating MM1s-GFP+ cells, while the new method detected close to 80% ± 24% of the circulating MM1s-GFP+ cells ($P = 0{\cdot}0001$) (Fig 4C).

Testing the prevalence of circulating MM cells in 20 MM patients with progressive disease using CD138-based or the new method, demonstrated that CD138 detected minimal amounts of MM cells in all patients (less than 0·2%), while the new method detected a range between 0·03–3·59% of MM cells in PBMCs (Fig 4D).

Discussion

CD138 (SDC1) is the gold-standard marker for detecting MM cells using immunohistochemistry and multi-parametric flow cytometry analysis of BM biopsies (Wijdenes *et al*, 1996; Lin *et al*, 2004; Rawstron *et al*, 2008; Flanders *et al*, 2013). A major concern regarding the use of CD138 to detect MM cells is that patients harbour a sub-population of cells that is CD138-negative. This sub-population is clonotypic, drug resistant, exhibits stem cell-like properties (Agarwal & Matsui, 2010; Ghiaur *et al*, 2012), expresses drug efflux pumps (Matsui *et al*, 2008; Yang *et al*, 2013), and displays higher clonogenic potential than the CD138+ sub-population *in vivo* (Reghunathan *et al*, 2013). In this study, we developed a new method to detect MM cells in both the BM and the circulation, using flow cytometry independent of the CD138 expression on these cells.

We have previously shown that the tumour microenvironment plays a significant role in progression and drug resistance in MM (Azab *et al*, 2009a,b, 2012b, 2014a,b; de la Puente & Azab, 2013; de la Puente *et al*, 2014; Abraham *et al*, 2015), and that hypoxia is a key factor for cell trafficking and dissemination of MM cells from one site to other sites in the BM (Azab *et al*, 2012a). Moreover, we have recently shown that hypoxia induces drug resistance in MM and that hypoxic MM cells downregulate the expression of CD138 (Muz *et al*, 2014a). In the present study, we developed a MRD model by treating MM-bearing mice with high-dose bortezomib until a low or undetectable tumour signal was obtained by BLI. We found that the residual MM cells in mice after bortezomib treatment were highly hypoxic. These results suggested that the MRD cells were hypoxic, and that bortezomib treatment selected for these cells. The expression of the plasma marker CD138 in the hypoxic cells representing MRD (PIM-positive) was significantly downregulated compared to normoxic MM cells, while CD38 expression was unchanged in the hypoxic-MRD cells. Simi-

© 2016 John Wiley & Sons Ltd

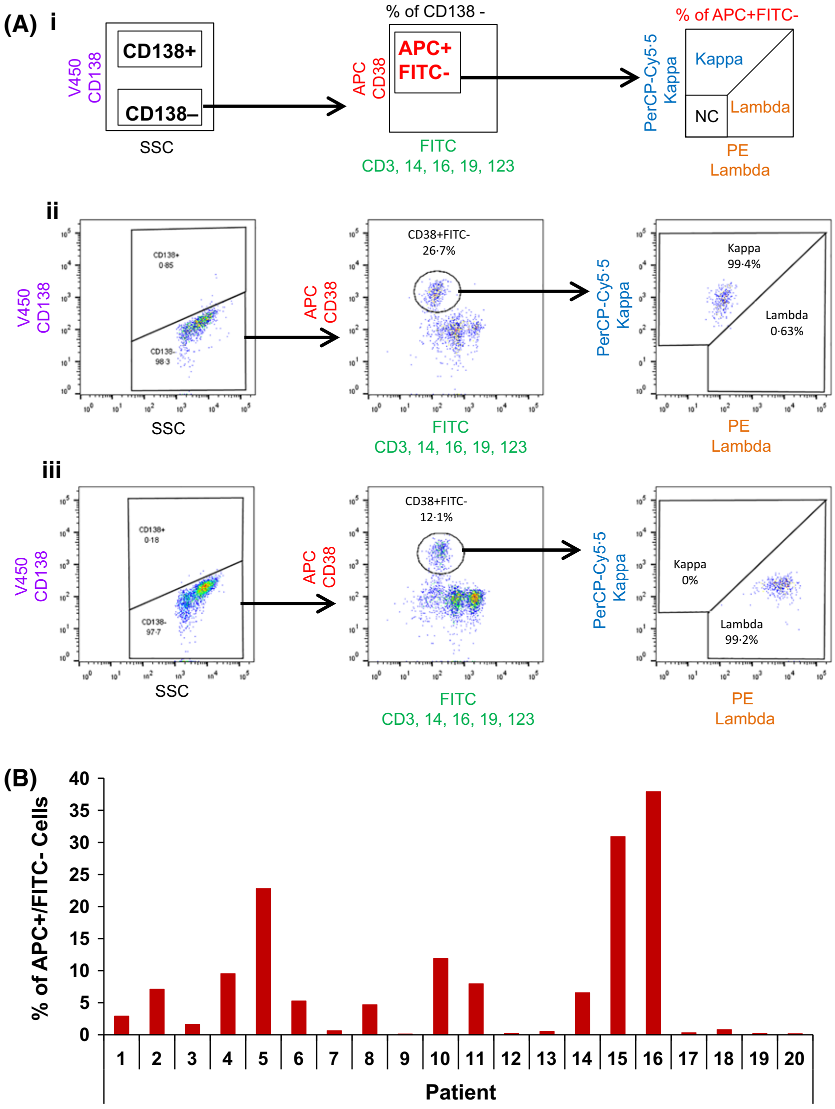

Fig 3. Detection of MM cells in patients' BM using the new strategy and confirming MM cell clonality. (Ai) The experimental approach of detecting multiple myeloma (MM) cells in the bone marrow (BM) negative fractions (CD138-depleted) from MM patients using the new method, and testing plasma cell clonality. Examples of kappa patient analysis (Aii) and lambda patient analysis (Aiii) are shown; NC - non-clonal cells. (B) Percentage of MM cells detected in MM patients by the new method. (C) Clonality assessment shown as the ratio between the percentage of kappa- and lambda-positive MM cell populations detected by the new method in the BM-negative fractions. The normal (polyclonal) ratio in healthy subjects ranges between 0·76 and 1·5, whereas values above or below this ratio indicate clonal proliferation of plasma cells. (D) The correlation study between tumour size (percentage of MM cells detected by the new method) and the clonality ratio in the lambda patients (Di) and kappa patients (Dii). (E) The level of non-clonal contamination (kappa-negative and lambda-negative) in the MM population detected by the new method.

larly, treating a panel of MM cell lines with hypoxia or bortezomib reduced the expression of CD138, but not CD38. These results suggest that stress conditions in MRD settings, such as hypoxia and cytotoxic treatment including bortezomib, down-regulate the expression of CD138; therefore, CD138 cannot be used to reliably detect the entire MM cell population.

Unlike CD138, CD38 expression did not change under these stress conditions (hypoxia and cytotoxic treatment); therefore, we sought to design a MM cell detection method based on the stable CD38 marker. However, CD38 is known to be expressed on a variety of blood cells, and about 15–20% of PBMCs from healthy donors are positive for CD38. Therefore, to be used as a valuable biomarker for MM we needed to exclude those CD38+ cells that are not MM plasma cells. We used specific markers to exclude the intended populations, such as CD3 for T cells, CD14 for monocytes, CD16 for NK cells, eosinophils and neutrophils, CD19 for B cells and CD123 for dendritic cells and basophils. These markers are suitable for excluding CD38+ non-MM cell populations, because their expression is unchanged in both normoxia and hypoxia.

Therefore, we explored the approach to detect MM cells as CD38-positive and CD3-, 14-, 16-, 19- and 123-negative by a two-colour flow cytometric method, in which case CD38 was detected with an APC-labelled antibody while all the other antibodies used for exclusion were detected with the corresponding antibodies conjugated to FITC (and in some cases, BV421).

In the *in vitro* and *in vivo* studies, we demonstrated again that CD138 is not a reliable marker for the detection of MM cells. This is due to the fact that the hypoxic sub-population of MM cells looses CD138 expression; therefore it is undetectable by the traditional CD138-based method. The use of CD38 alone as a marker is also inadequate because it detects both MM and additional non-MM cells. However, the

© 2016 John Wiley & Sons Ltd

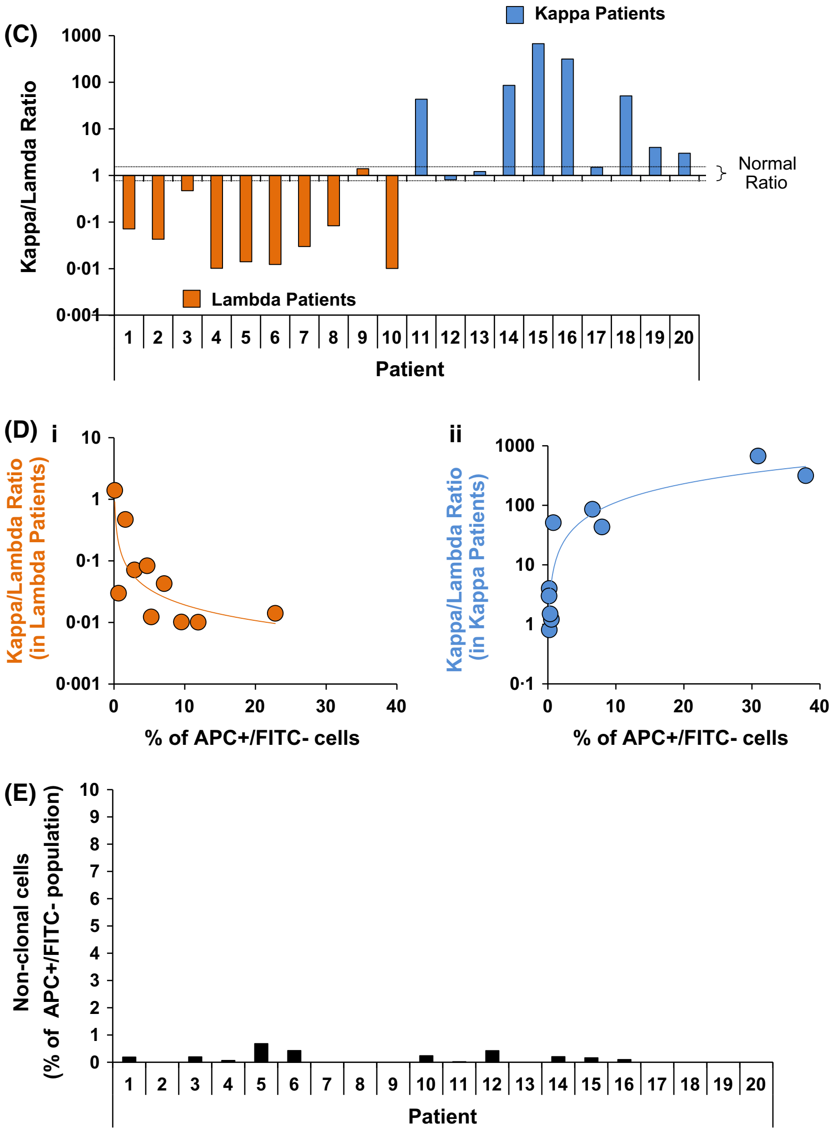

Fig 3. Continued

new method was able to detect both normoxic and hypoxic MM cells *in vitro*. Next, the new method was tested in MM-bearing mice with different tumour sizes, implying different hypoxic states. The CD138 staining showed significantly lower levels of detected MM cells, usually below 10% of total BMMCs, regardless of tumour size. On the contrary, the new method did not only detect all of the MM1s-GFP+ cells, but also it did not detect any additional contaminating cells. The new method detected all MM cells, independent of their hypoxic state, both CD138-positive and CD138-negative, with no additional contamination.

To validate the presence of MM population detected by the new markers in patients, we analysed CD138-depleted BM samples from 20 progressive MM patients (10 kappa and 10 lambda) that were stained for the new markers as well as CD138. We found that, in 16 out of 20 samples, the CD138-negative population included between 0·1% and 37·9% of MM cells detected with the new method, whereas CD138-detected residual MM cells were <0·5%. In 4 out of 20 patients, the percentage of MM cells detected by the new method was below 0·5% with a normal clonality ratio, indicating polyclonal plasma cells and suggesting that these patients were most probably successfully treated. We further confirmed the clonality of these CD138-negative cells detected by the new method by determining the kappa/lambda ratio of these cells, and showed that the clonality of each population matched the clonality of the CD138+ cells, with less than 0·69% of non-clonal contamination. Moreover, the kappa/lambda clonality ratio was in direct (for kappa patients) and indirect (for lambda patients) correlation with the tumour burden detected. These results suggest that the cell populations detected by the new method are indeed clonal MM cells.

CTCs are considered an unfavourable prognostic factor and indicate an aggressive form of the disease (Paiva *et al*, 2013; An *et al*, 2015), and therefore detecting CTCs can be a

© 2016 John Wiley & Sons Ltd

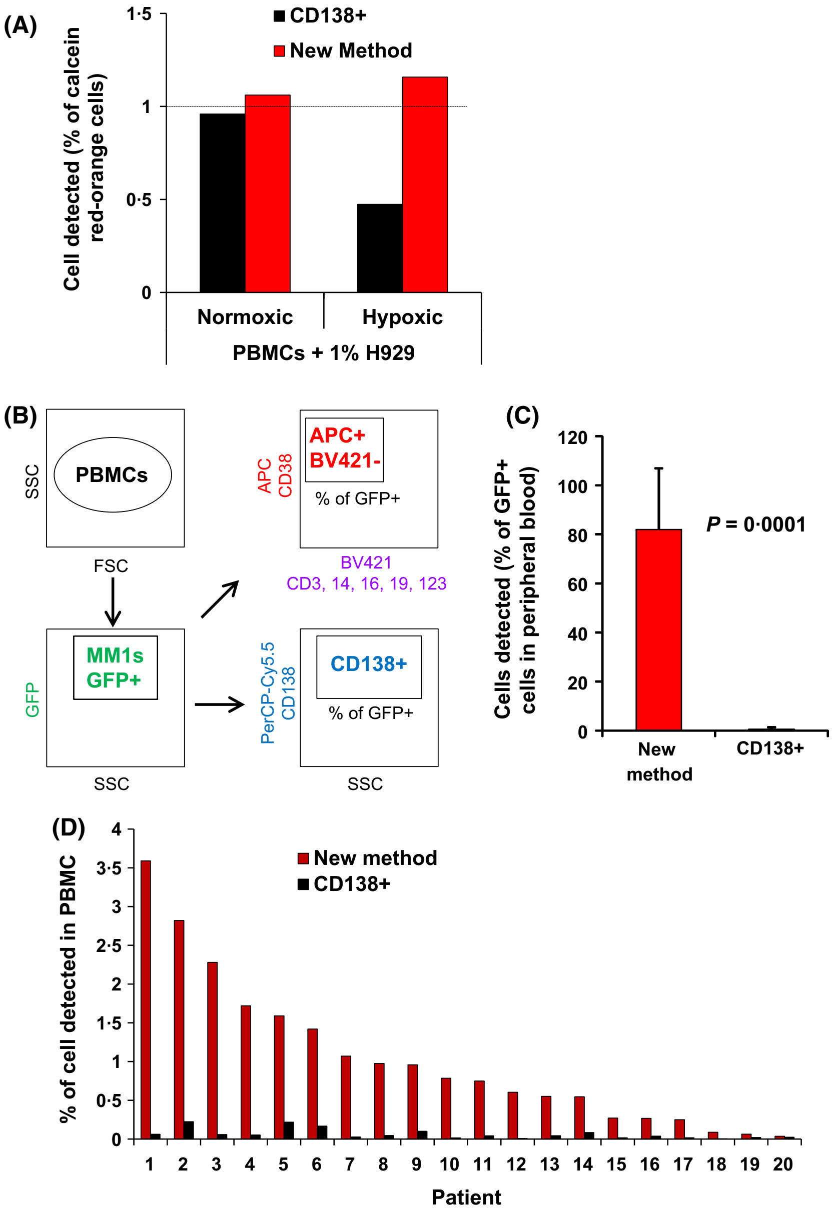

Fig 4. Detection of MM cells in peripheral blood using the new strategy. (A) Detection of normoxic and hypoxic calcein red-orange+ H929 cells spiked into peripheral blood mononuclear cells (PBMCs) detected by flow cytometry using CD138 and the new method. (B) The experimental approach of detecting multiple myeloma (MM) cells in PBMCs from six mice using three strategies: GFP+, CD138+ and the new method. (C) Percentage of MM cells identified by CD138+ or the new method and plotted against the % of GFP+ cells detected in the peripheral blood from five mice shown as a mean ± standard deviation and analysed by two-sided student *t*-test. (D) The prevalence of circulating MM cells (%) in 20 MM patients with progressive disease using CD138 or the new method. GFP, green fluorescent protein; APC, allophycocyanin

powerful prognostic tool for MM (Peceliunas *et al*, 2012). We have previously shown that CTCs derive from a hypoxic sub-population that intravasated from the BM to the circulation and have enhanced homing (thus metastasis) to new BM niches (Azab *et al*, 2012a, 2013; Muz *et al*, 2014a,b, 2015). Given that CTCs show a hypoxic phenotype, the CD138-based method is not sufficient to detect these cells. Detecting normoxic and hypoxic MM cells *in vitro* and MM cells *in vivo* as well as in MM patients' samples, demonstrated that CD138 failed to detect the majority of MM cells, compared to the new method. These results demonstrate that circulating MM cells have low expression of CD138, which is in agreement with previous studies (Paiva *et al*, 2013), and that shedding of CD138 is most prevalent in circulating MM cells (Seidel *et al*, 2000). Despite the fact that CD138 was used previously as the main marker to detect CTCs in MM (Peceliunas *et al*, 2012; Paiva *et al*, 2013; An *et al*, 2015), the current findings suggests that CD138 is not a reliable marker to detect CTCs in this disorder, in accordance with recently published studies (Thiago *et al*, 2014), and that the new method to detect MM cells could be used for detection of CTCs in MM. Future studies are necessary to determine the value of the new biomarker set as a prognostic tool for detecting CTCs.

Despite the introduction of novel therapies, more than 90% of MM patients relapse (Lobo *et al*, 2007; Nair *et al*, 2012; Ludwig *et al*, 2013). Tumour recurrence is attributed to the development of a sub-population of MM cells, i.e. MRD, which are resistant to therapy (Brennan & Matsui, 2009; Matsui, 2011; Pascutti *et al*, 2013). Several studies have demonstrated a consistent improvement in progression-free survival (PFS) with attainment of a complete remission (CR)

© 2016 John Wiley & Sons Ltd

where no residual cells were detected after treatment using the CD138 marker, but its impact on overall survival has been variable (Harousseau *et al*, 2010; Gay *et al*, 2011). Given the toxicity and negative impact on the quality of life from the intense therapies required for a CR, it is imperative that the absence of remaining population after treatment translates into improved overall survival, which is an essential step towards PFS (Paiva *et al*, 2008; Rawstron *et al*, 2013). However, the approaches to define the remaining population after treatment (MRD) are still controversial (Brennan & Matsui, 2009; Basak & Carrier, 2010). In the future, we will use the new method to detect MRD cells in the BM from patients in CR, compare the results to the CD138-based method or immunohistochemistry, and correlate the amount of MM cells detected by each method to the clinical data of tumour recurrence and survival.

In conclusion, we have shown that MRD cells after drug treatment are hypoxic, and that hypoxia and drug treatment down-regulate the expression of the main surface marker (CD138) used to identify MM cells, rendering this marker unsuitable for the detection of MM cells. Thus, we developed an alternative biomarker set that detects myeloma cells independent of their hypoxic and CD138 expression status *in vitro*, *in vivo* and in primary MM patient samples. The new markers were able to identify a clonal CD138-negative population as MRD in the BM and circulation of MM patients. Further investigation to characterize this population and its role in relapse after treatment, as well as the use of the CTCs detected by the new method as a prognostic marker in MM is warranted.

Acknowledgement

The study was supported in part by a grant from the Multiple Myeloma Research Foundation and the National Cancer Institute of the National Institutes of Health under Award Number U54CA199092. The content is solely the responsibility of the authors and does not necessarily represent the official views of the National Institutes of Health.

Author contributions

B.M. designed the study, performed research, analysed and interpreted data, and wrote the manuscript. P.P., F.A., and M.J.L performed research, analysed and interpreted data. J.K. and R.V. provided primary MM samples, analysed and interpreted data. A.K.A. designed the study, analysed and interpreted data, wrote the manuscript and supervised the study.

Conflict of interest

Dr. Azab AK receives research support from Verastem, Selexys, Karyopharm and Cell Works, and is the founder and owner of Targeted Therapeutics LLC and Cellatrix LLC. Moreover, Dr. Azab AK, Dr. Muz B and Mrs. Azab F have a PCT patent application on the method described in this manuscript. Other authors state no conflicts of interest.

References

Abraham, J., Salama, N.N. & Azab, A.K. (2015) The role of P-glycoprotein in drug resistance in multiple myeloma. *Leukaemia & Lymphoma*, **56**, 26–33.

Agarwal, J.R. & Matsui, W. (2010) Multiple myeloma: a paradigm for translation of the cancer stem cell hypothesis. *Anticancer Agents Med Chem*, **10**, 116–120.

Almeida, J., Orfao, A., Mateo, G., Ocqueteau, M., Garcia-Sanz, R., Moro, M.J., Hernandez, J., Ortega, F., Borrego, D., Barez, A., Mejido, M. & San Miguel, J.F. (1999) Immunophenotypic and DNA content characteristics of plasma cells in multiple myeloma and monoclonal gammopathy of undetermined significance. *Pathologie Biologie*, **47**, 119–127.

An, G., Qin, X., Acharya, C., Xu, Y., Deng, S., Shi, L., Zang, M., Sui, W., Yi, S., Li, Z., Hao, M., Feng, X., Jin, F., Zou, D., Qi, J., Zhao, Y., Tai, Y.T., Wang, J. & Qiu, L. (2015) Multiple myeloma patients with low proportion of circulating plasma cells had similar survival with primary plasma cell leukemia patients. *Annals of Hematology*, **94**, 257–264.

Azab, A.K., Azab, F., Blotta, S., Pitsillides, C.M., Thompson, B., Runnels, J.M., Roccaro, A.M., Ngo, H.T., Melhem, M.R., Sacco, A., Jia, X., Anderson, K.C., Lin, C.P., Rollins, B.J. & Ghobrial, I.M. (2009a) RhoA and Rac1 GTPases play major and differential roles in stromal cell-derived factor-1-induced cell adhesion and chemotaxis in multiple myeloma. *Blood*, **114**, 619–629.

Azab, A.K., Runnels, J.M., Pitsillides, C., Moreau, A.S., Azab, F., Leleu, X., Jia, X., Wright, R., Ospina, B., Carlson, A.L., Alt, C., Burwick, N., Roccaro, A.M., Ngo, H.T., Farag, M., Melhem, M.R., Sacco, A., Munshi, N.C., Hideshima, T., Rollins, B.J., Anderson, K.C., Kung, A.L., Lin, C.P. & Ghobrial, I.M. (2009b) CXCR4 inhibitor AMD3100 disrupts the interaction of multiple myeloma cells with the bone marrow microenvironment and enhances their sensitivity to therapy. *Blood*, **113**, 4341–4351.

Azab, A.K., Hu, J., Quang, P., Azab, F., Pitsillides, C., Awwad, R., Thompson, B., Maiso, P., Sun, J.D., Hart, C.P., Roccaro, A.M., Sacco, A., Ngo, H.T., Lin, C.P., Kung, A.L., Carrasco, R.D., Vanderkerken, K. & Ghobrial, I.M. (2012a) Hypoxia promotes dissemination of multiple myeloma through acquisition of epithelial to mesenchymal transition-like features. *Blood*, **119**, 5782–5794.

Azab, A.K., Quang, P., Azab, F., Pitsillides, C., Thompson, B., Chonghaile, T., Patton, J.T., Maiso, P., Monrose, V., Sacco, A., Ngo, H.T., Flores, L.M., Lin, C.P., Magnani, J.L., Kung, A.L., Letai, A., Carrasco, R., Roccaro, A.M. & Ghobrial, I.M. (2012b) P-selectin glycoprotein ligand regulates the interaction of multiple myeloma cells with the bone marrow microenvironment. *Blood*, **119**, 1468–1478.

Azab, A.K., Weisberg, E., Sahin, I., Liu, F., Awwad, R., Azab, F., Liu, Q., Griffin, J.D. & Ghobrial, I.M. (2013) The influence of hypoxia on CML trafficking through modulation of CXCR4 and E-cadherin expression. *Leukemia*, **27**, 961–964.

Azab, A.K., Sahin, I., Moschetta, M., Mishima, Y., Burwick, N., Zimmermann, J., Romagnoli, B., Patel, K., Chevalier, E., Roccaro, A.M. & Ghobria, I.M. (2014a) CXCR7-dependent angiogenic mononuclear cell trafficking regulates tumor progression in multiple myeloma. *Blood*, **124**, 1905–1914.

Azab, F., Vali, S., Abraham, J., Potter, N., Muz, B., de la Puente, P., Fiala, M., Paasch, J., Sultana, Z., Tyagi, A., Abbasi, T., Vij, R. & Azab, A.K. (2014b) PI3KCA plays a major role in multiple myeloma and its inhibition with BYL719 decreases proliferation, synergizes with other therapies and over-

© 2016 John Wiley & Sons Ltd

comes stroma-induced resistance. *British Journal of Haematology*, **165**, 89–101.

Basak, G.W. & Carrier, E. (2010) The search for multiple myeloma stem cells: the long and winding road. *Biology of Blood and Marrow Transplantation*, **16**, 587–594.

Brennan, S.K. & Matsui, W. (2009) Cancer stem cells: controversies in multiple myeloma. *Journal of Molecular Medicine (Berlin)*, **87**, 1079–1085.

Flanders, A., Stetler-Stevenson, M. & Landgren, O. (2013) Minimal residual disease testing in multiple myeloma by flow cytometry: major heterogeneity. *Blood*, **122**, 1088–1089.

Gay, F., Larocca, A., Wijermans, P., Cavallo, F., Rossi, D., Schaafsma, R., Genuardi, M., Romano, A., Liberati, A.M., Siniscalchi, A., Petrucci, M.T., Nozzoli, C., Patriarca, F., Offidani, M., Ria, R., Omede, P., Bruno, B., Passera, R., Musto, P., Boccadoro, M., Sonneveld, P. & Palumbo, A. (2011) Complete response correlates with long-term progression-free and overall survival in elderly myeloma treated with novel agents: analysis of 1175 patients. *Blood*, **117**, 3025–3031.

Ghiaur, G., Gerber, J.M., Matsui, W. & Jones, R.J. (2012) Cancer stem cells: relevance to clinical transplantation. *Current Opinion in Oncology*, **24**, 170–175.

Harousseau, J.L., Palumbo, A., Richardson, P.G., Schlag, R., Dimopoulos, M.A., Shpilberg, O., Kropff, M., Kentos, A., Cavo, M., Golenkov, A., Komarnicki, M., Mateos, M.V., Esseltine, D.L., Cakana, A., Liu, K., Deraedt, W., van de Velde, H. & San Miguel, J.F. (2010) Superior outcomes associated with complete response in newly diagnosed multiple myeloma patients treated with nonintensive therapy: analysis of the phase 3 VISTA study of bortezomib plus melphalan-prednisone versus melphalan-prednisone. *Blood*, **116**, 3743–3750.

He, X., Brenchley, P.E., Jayson, G.C., Hampson, L., Davies, J. & Hampson, I.N. (2004) Hypoxia increases heparanase-dependent tumor cell invasion, which can be inhibited by antiheparanase antibodies. *Cancer Research*, **64**, 3928–3933.

Kumar, S.K., Rajkumar, S.V., Dispenzieri, A., Lacy, M.Q., Hayman, S.R., Buadi, F.K., Zeldenrust, S.R., Dingli, D., Russell, S.J., Lust, J.A., Greipp, P.R., Kyle, R.A. & Gertz, M.A. (2008) Improved survival in multiple myeloma and the impact of novel therapies. *Blood*, **111**, 2516–2520.

Kyle, R.A., Gertz, M.A., Witzig, T.E., Lust, J.A., Lacy, M.Q., Dispenzieri, A., Fonseca, R., Rajkumar, S.V., Offord, J.R., Larson, D.R., Plevak, M.E., Therneau, T.M. & Greipp, P.R. (2003) Review of 1027 patients with newly diagnosed multiple myeloma. *Mayo Clinic Proceedings*, **78**, 21–33.

Ladetto, M., Bruggemann, M., Monitillo, L., Ferrero, S., Pepin, F., Drandi, D., Barbero, D., Palumbo, A., Passera, R., Boccadoro, M., Ritgen, M., Gokbuget, N., Zheng, J., Carlton, V., Trautmann, H., Faham, M. & Pott, C. (2014) Next-generation sequencing and real-time quantitative PCR for minimal residual disease detection in B-cell disorders. *Leukemia*, **28**, 1299–1307.

Lin, P., Owens, R., Tricot, G. & Wilson, C.S. (2004) Flow cytometric immunophenotypic analysis of 306 cases of multiple myeloma. *American Journal of Clinical Pathology*, **121**, 482–488.

Lobo, N.A., Shimono, Y., Qian, D. & Clarke, M.F. (2007) The biology of cancer stem cells. *Annual Review of Cell and Developmental Biology*, **23**, 675–699.

Ludwig, H. (2005) Advances in biology and treatment of multiple myeloma. *Annals of Oncology*, **16** Suppl. 2, ii106–112.

Ludwig, H., Milosavljevic, D., Zojer, N., Faint, J.M., Bradwell, A.R., Hubl, W. & Harding, S.J. (2013) Immunoglobulin heavy/light chain ratios improve paraprotein detection and monitoring, identify residual disease and correlate with survival in multiple myeloma patients. *Leukemia*, **27**, 213–219.

Martinez-Sanchez, P., Montejano, L., Sarasquete, M.E., Garcia-Sanz, R., Fernandez-Redondo, E., Ayala, R., Montalban, M.A., Martinez, R., Garcia Larana, J., Alegre, A., Hernandez, B., Lahuerta, J.J. & Martinez-Lopez, J. (2008) Evaluation of minimal residual disease in multiple myeloma patients by fluorescent-polymerase chain reaction: the prognostic impact of achieving molecular response. *British Journal of Haematology*, **142**, 766–774.

Matsui, W. (2011) Perspective: a model disease. *Nature*, **480**, S58.

Matsui, W., Wang, Q., Barber, J.P., Brennan, S., Smith, B.D., Borrello, I., McNiece, I., Lin, L., Ambinder, R.F., Peacock, C., Watkins, D.N., Huff, C.A. & Jones, R.J. (2008) Clonogenic multiple myeloma progenitors, stem cell properties, and drug resistance. *Cancer Research*, **68**, 190–197.

Mitsiades, C.S., Hayden, P.J., Anderson, K.C. & Richardson, P.G. (2007) From the bench to the bedside: emerging new treatments in multiple myeloma. *Best Pract Res Clin Haematol*, **20**, 797–816.

Muz, B., de la Puente, P., Azab, F., Luderer, M. & Azab, A.K. (2014a) Hypoxia promotes stem cell-like phenotype in multiple myeloma cells. *Blood Cancer J*, **4**, e262.

Muz, B., de la Puente, P., Azab, F., Luderer, M. & Azab, A.K. (2014b) The role of hypoxia and exploitation of the hypoxic environment in hematologic malignancies. *Molecular Cancer Research*, **12**, 1347–1354.

Muz, B., de la Puente, P., Azab, F., Ghobrial, I.M. & Azab, A.K. (2015) Hypoxia promotes dissemination and colonization in new bone marrow niches in Waldenstrom macroglobulinemia. *Molecular Cancer Research*, **13**, 263–272.

Nair, R.R., Gebhard, A.W., Emmons, M.F. & Hazlehurst, L.A. (2012) Emerging strategies for targeting cell adhesion in multiple myeloma. *Advances in Pharmacology*, **65**, 143–189.

Nakayama, S., Yokote, T., Hirata, Y., Iwaki, K., Akioka, T., Miyoshi, T., Nishiwaki, U., Masuda, Y., Hiraoka, N., Takayama, A., Nishimura, Y., Tsuji, M. & Hanafusa, T. (2012) Immunohistological analysis in diagnosis of plasma cell myeloma based on cytoplasmic kappa/lambda ratio of CD38-positive plasma cells. *Hematology*, **17**, 317–320.

Paiva, B., Vidriales, M.B., Cervero, J., Mateo, G., Perez, J.J., Montalban, M.A., Sureda, A., Montejano, L., Gutierrez, N.C., Garcia de Coca, A., de Las Heras, N., Mateos, M.V., Lopez-Berges, M.C., Garcia-Boyero, R., Galende, J., Hernandez, J., Palomera, L., Carrera, D., Martinez, R., de la Rubia, J., Martin, A., Blade, J., Lahuerta, J.J., Orfao, A. & San Miguel, J.F. (2008) Multiparameter flow cytometric remission is the most relevant prognostic factor for multiple myeloma patients who undergo autologous stem cell transplantation. *Blood*, **112**, 4017–4023.

Paiva, B., Paino, T., Sayagues, J.M., Garayoa, M., San-Segundo, L., Martin, M., Mota, I., Sanchez, M.L., Barcena, P., Aires-Mejia, I., Corchete, L., Jimenez, C., Garcia-Sanz, R., Gutierrez, N.C., Ocio, E.M., Mateos, M.V., Vidriales, M.B., Orfao, A. & San Miguel, J.F. (2013) Detailed characterization of multiple myeloma circulating tumor cells shows unique phenotypic, cytogenetic, functional, and circadian distribution profile. *Blood*, **122**, 3591–3598.

Pascutti, F., Cunha, L.L., Rizzatti, E.G. & Colleoni, G.W. (2013) Understanding myeloma cancer stem cells. *Immunotherapy*, **5**, 1291–1294.

Peceliunas, V., Janiulioniene, A., Matuzeviciene, R., Zvirblis, T. & Griskevicius, L. (2012) Circulating plasma cells predict the outcome of relapsed or refractory multiple myeloma. *Leukaemia & Lymphoma*, **53**, 641–647.

de la Puente, P. & Azab, A.K. (2013) Contemporary drug therapies for multiple myeloma. *Drugs Today (Barc)*, **49**, 563–573.

de la Puente, P., Muz, B., Azab, F., Luderer, M. & Azab, A.K. (2014) Molecularly targeted therapies in multiple myeloma. *Leuk Res Treatment*, **2014**, 976567.

Puig, N., Sarasquete, M.E., Balanzategui, A., Martinez, J., Paiva, B., Garcia, H., Fumero, S., Jimenez, C., Alcoceba, M., Chillon, M.C., Sebastian, E., Marin, L., Montalban, M.A., Mateos, M.V., Oriol, A., Palomera, L., de la Rubia, J., Vidriales, M.B., Blade, J., Lahuerta, J.J., Gonzalez, M., Miguel, J.F. & Garcia-Sanz, R. (2014) Critical evaluation of ASO RQ-PCR for minimal residual disease evaluation in multiple myeloma. A comparative analysis with flow cytometry. *Leukemia*, **28**, 391–397.

Rawstron, A.C., Orfao, A., Beksac, M., Bezdickova, L., Brooimans, R.A., Bumbea, H., Dalva, K., Fuhler, G., Gratama, J., Hose, D., Kovarova, L., Lioznov, M., Mateo, G., Morilla, R., Mylin, A.K., Omede, P., Pellat-Deceunynck, C., Perez Andres, M., Petrucci, M., Ruggeri, M., Rymkiewicz, G., Schmitz, A., Schreder, M., Seynaeve, C., Spacek, M., de Tute, R.M., Van Valckenborgh, E., Weston-Bell, N., Owen, R.G., San Miguel, J.F., Sonneveld, P., Johnsen, H.E. & European Myeloma, N. (2008) Report of the European Myeloma Network on multiparametric flow cytometry in multiple mye-

© 2016 John Wiley & Sons Ltd

loma and related disorders. *Haematologica*, **93**, 431–438.

Rawstron, A.C., Child, J.A., de Tute, R.M., Davies, F.E., Gregory, W.M., Bell, S.E., Szubert, A.J., Navarro-Coy, N., Drayson, M.T., Feyler, S., Ross, F.M., Cook, G., Jackson, G.H., Morgan, G.J. & Owen, R.G. (2013) Minimal residual disease assessed by multiparameter flow cytometry in multiple myeloma: impact on outcome in the Medical Research Council Myeloma IX Study. *Journal of Clinical Oncology*, **31**, 2540–2547.

Reghunathan, R., Bi, C., Liu, S.C., Loong, K.T., Chung, T.H., Huang, G. & Chng, W.J. (2013) Clonogenic multiple myeloma cells have shared stemness signature associated with patient survival. *Oncotarget*, **4**, 1230–1240.

San Miguel, J.F., Gutierrez, N.C., Mateo, G. & Orfao, A. (2006) Conventional diagnostics in multiple myeloma. *European Journal of Cancer*, **42**, 1510–1519.

Seidel, C., Sundan, A., Hjorth, M., Turesson, I., Dahl, I.M., Abildgaard, N., Waage, A. & Borset, M. (2000) Serum syndecan-1: a new independent prognostic marker in multiple myeloma. *Blood*, **95**, 388–392.

Thiago, L.S., Perez-Andres, M., Balanzategui, A., Sarasquete, M.E., Paiva, B., Jara-Acevedo, M., Barcena, P., Sanchez, M.L., Almeida, J., Gonzalez, M., San Miguel, J.F., Garcia-Sanz, R. & Orfao, A. (2014) Circulating clonotypic B cells in multiple myeloma and monoclonal gammopathy of undetermined significance. *Haematologica*, **99**, 155–162.

Wijdenes, J., Vooijs, W.C., Clement, C., Post, J., Morard, F., Vita, N., Laurent, P., Sun, R.X., Klein, B. & Dore, J.M. (1996) A plasmocyte selective monoclonal antibody (B-B4) recognizes syndecan-1. *British Journal of Haematology*, **94**, 318–323.

Wu, W., Pan, C., Meng, K., Zhao, L., Du, L., Liu, Q. & Lin, R. (2010) Hypoxia activates heparanase expression in an NF-kappaB dependent manner. *Oncology Reports*, **23**, 255–261.

Yang, Y., Shi, J., Tolomelli, G., Xu, H., Xia, J., Wang, H., Zhou, W., Zhou, Y., Das, S., Gu, Z., Levasseur, D., Zhan, F. & Tricot, G. (2013) RARalpha2 expression confers myeloma stem cell features. *Blood*, **122**, 1437–1447.

© 2016 John Wiley & Sons Ltd
