# Supplementary material for: Expression of Concern: Prognostic value of circulating plasma cells in patients with multiple myeloma: A meta-analysis
Source: PLoS One. 2023 Feb 21;18(2):e0282230. doi: 10.1371/journal.pone.0282230 (PMC9942954; doi:10.1371/journal.pone.0282230)
Supplement: S1 File — (ZIP) [file pone.0282230.s001.zip › primary data/stata20170109/11-20170109.docx]

**PFS**

Heterogeneity chi-squared = 9.19 (d.f. = 8) p = 0.327

I-squared (variation in ES attributable to heterogeneity) = 12.9%

Test of ES=1 : z= 8.82 p = 0.000

Test(s) of heterogeneity:

Heterogeneity degrees of

statistic freedom P I-squared**

IF 0.00 0 . .%

FCM 4.31 4 0.366 7.2%

PCR 0.74 1 0.390 0.0%

CM 0.00 0 . .%

Overall 9.19 8 0.327 12.9%

Overall Test for heterogeneity between sub-groups:

4.14 3 0.247

** I-squared: the variation in ES attributable to heterogeneity)

Some heterogeneity observed (up to 7.2%) in one or more sub-groups,

Test for heterogeneity between sub-groups may be invalid

Significance test(s) of ES=

IF z= 0.81 p = 0.420

FCM z= 7.82 p = 0.000

PCR z= 2.69 p = 0.007

CM z= 3.58 p = 0.000

Overall z= 8.82 p = 0.000

Test(s) of heterogeneity:

Heterogeneity degrees of

statistic freedom P I-squared**

Before SCT 3.04 3 0.386 1.3%

RA 0.00 0 . .%

After SCT 0.00 0 . .%

First diagnosis 2.34 2 0.311 14.4%

Overall 9.19 8 0.327 12.9%

Overall Test for heterogeneity between sub-groups:

3.81 3 0.283

** I-squared: the variation in ES attributable to heterogeneity)

Some heterogeneity observed (up to 14.4%) in one or more sub-groups,

Test for heterogeneity between sub-groups may be invalid

Significance test(s) of ES=1

Before SCT z= 6.80 p = 0.000

RA z= 2.02 p = 0.043

After SCT z= 2.79 p = 0.005

First diagnosis z= 4.84 p = 0.000

Overall z= 8.82 p = 0.000

Begg's Test

adj. Kendall's Score (P-Q) = 4

Std. Dev. of Score = 9.59

Number of Studies = 9

z = 0.42

Pr > |z| = 0.677

z = 0.31 (continuity corrected)

Pr > |z| = 0.754 (continuity corrected)

Egger's test

------------------------------------------------------------------------------

Std_Eff | Coef. Std. Err. t P>|t| [95% Conf. Interval]

-------------+----------------------------------------------------------------

slope | .4705129 .1228554 3.83 0.006 .1800059 .7610198

bias | .6707243 .6231508 1.08 0.317 -.8027931 2.144242

os

Heterogeneity chi-squared = 12.51 (d.f. = 9) p = 0.186

I-squared (variation in ES attributable to heterogeneity) = 28.1%

Test of ES=1 : z= 8.66 p = 0.000


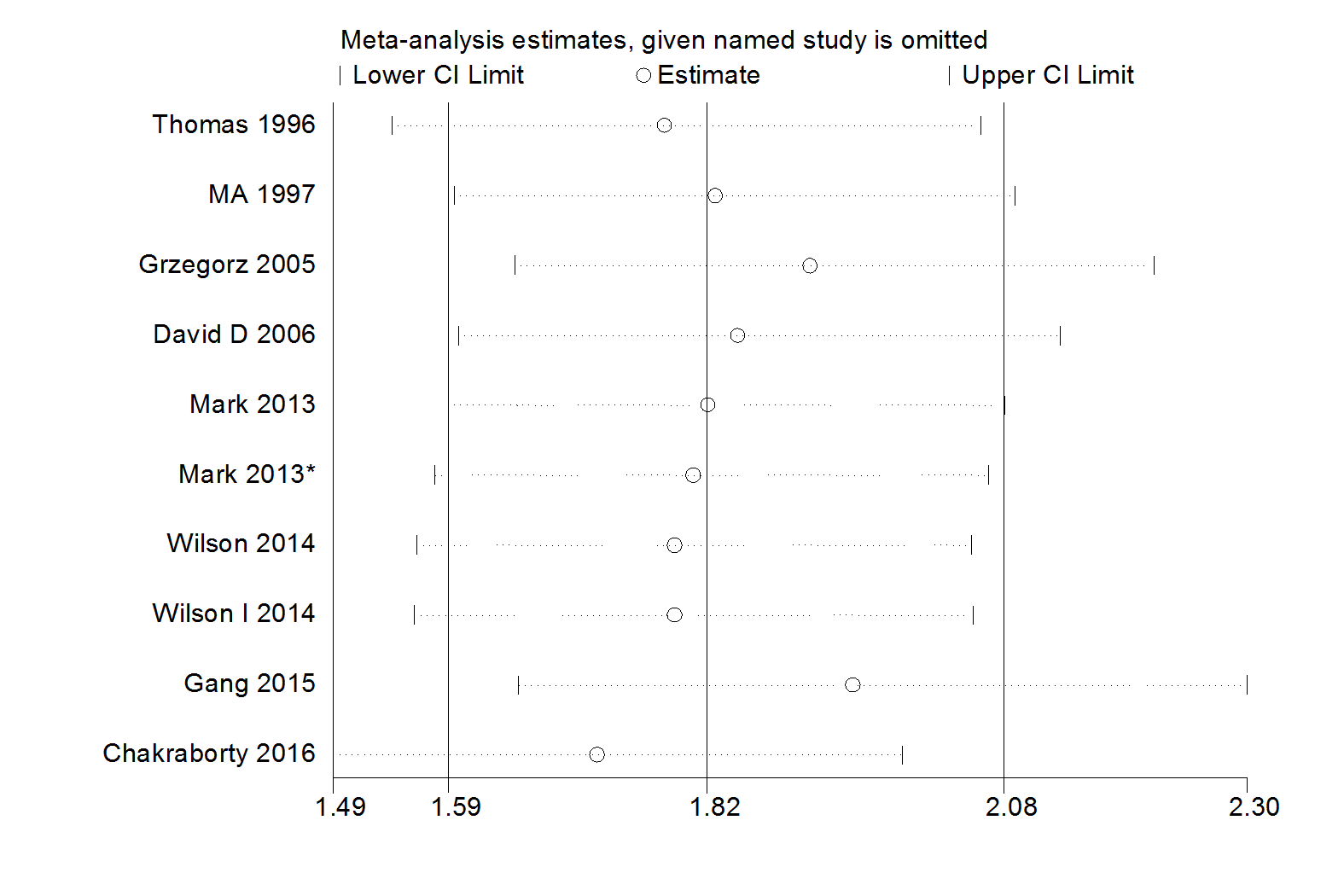

Test(s) of heterogeneity:

Heterogeneity degrees of

statistic freedom P I-squared**

IF 0.50 1 0.480 0.0%

FCM 8.43 4 0.077 52.6%

PCR 0.54 1 0.463 0.0%

CM 0.00 0 . .%

Overall 12.51 9 0.186 28.1%

Overall Test for heterogeneity between sub-groups:

3.04 3 0.385

** I-squared: the variation in ES attributable to heterogeneity)

Moderate heterogeneity observed (up to 52.6%) in one or more sub-groups,

Test for heterogeneity between sub-groups likely to be invalid

Significance test(s) of ES=1

IF z= 3.95 p = 0.000

FCM z= 6.64 p = 0.000

PCR z= 1.89 p = 0.058

CM z= 3.83 p = 0.000

Overall z= 8.66 p = 0.000

Test(s) of heterogeneity:

Heterogeneity degrees of

statistic freedom P I-squared**

First diagnosis 4.85 3 0.183 38.1%

Before SCT 3.09 3 0.377 3.0%

After SCT 0.00 0 . .%

RA 0.00 0 . .%

Overall 12.51 9 0.186 28.1%

Overall Test for heterogeneity between sub-groups:

4.57 3 0.206

** I-squared: the variation in ES attributable to heterogeneity)

Some heterogeneity observed (up to 38.1%) in one or more sub-groups,

Test for heterogeneity between sub-groups may be invalid

Significance test(s) of ES=1

First diagnosis z= 6.16 p = 0.000

Before SCT z= 5.42 p = 0.000

After SCT z= 1.99 p = 0.047

RA z= 2.88 p = 0.004

Overall z= 8.66 p = 0.000

Tests for Publication Bias

Begg's Test

adj. Kendall's Score (P-Q) = 12

Std. Dev. of Score = 11.14 (corrected for ties)

Number of Studies = 10

z = 1.08

Pr > |z| = 0.281

z = 0.99 (continuity corrected)

Pr > |z| = 0.323 (continuity corrected)

Egger's test

------------------------------------------------------------------------------

Std_Eff | Coef. Std. Err. t P>|t| [95% Conf. Interval]

-------------+----------------------------------------------------------------

slope | .4252378 .1495131 2.84 0.022 .0804599 .7700157

bias | .9281768 .6841711 1.36 0.212 -.6495247 2.505878

------------------------------------------------------------------------------

metan var2 var3 var4 var5, label(namevar=var1) random or

Study | OR [95% Conf. Interval] % Weight

---------------------+---------------------------------------------------

Mark 2013 | 1.375 0.074 25.433 2.68

Mark 2013* | 6.600 0.862 50.541 5.11

Wilson 2014 | 3.967 1.782 8.829 18.69

Gang 2015 | 4.404 2.630 7.372 26.11

Davide V 2015 | 1.971 0.868 4.479 18.23

Chakraborty 2016 | 1.663 1.106 2.500 29.18

---------------------+---------------------------------------------------

D+L pooled OR | 2.778 1.691 4.564 100.00

---------------------+---------------------------------------------------

Heterogeneity chi-squared = 11.11 (d.f. = 5) p = 0.049

I-squared (variation in OR attributable to heterogeneity) = 55.0%

Estimate of between-study variance Tau-squared = 0.1766

Test of OR=1 : z= 4.03 p = 0.000

metan var2 var3 var4 var5, label(namevar=var1) random or

Study | OR [95% Conf. Interval] % Weight

---------------------+---------------------------------------------------

David D 2006 | 1.069 0.607 1.883 35.13

Mark 2013 | 0.727 0.039 13.452 6.02

Mark 2013* | 2.767 0.129 59.482 5.52

Gang 2015 | 4.652 1.854 11.673 26.81

Davide V 2015 | 1.000 0.393 2.542 26.52

---------------------+---------------------------------------------------

D+L pooled OR | 1.604 0.742 3.470 100.00

---------------------+---------------------------------------------------

Heterogeneity chi-squared = 8.78 (d.f. = 4) p = 0.067

I-squared (variation in OR attributable to heterogeneity) = 54.4%

Estimate of between-study variance Tau-squared = 0.3579

Test of OR=1 : z= 1.20 p = 0.230
