# Supplementary figures and images for: Expression of Concern: Prognostic value of circulating plasma cells in patients with multiple myeloma: A meta-analysis
Source: PLoS One. 2023 Feb 21;18(2):e0282230. doi: 10.1371/journal.pone.0282230 (PMC9942954; doi:10.1371/journal.pone.0282230)

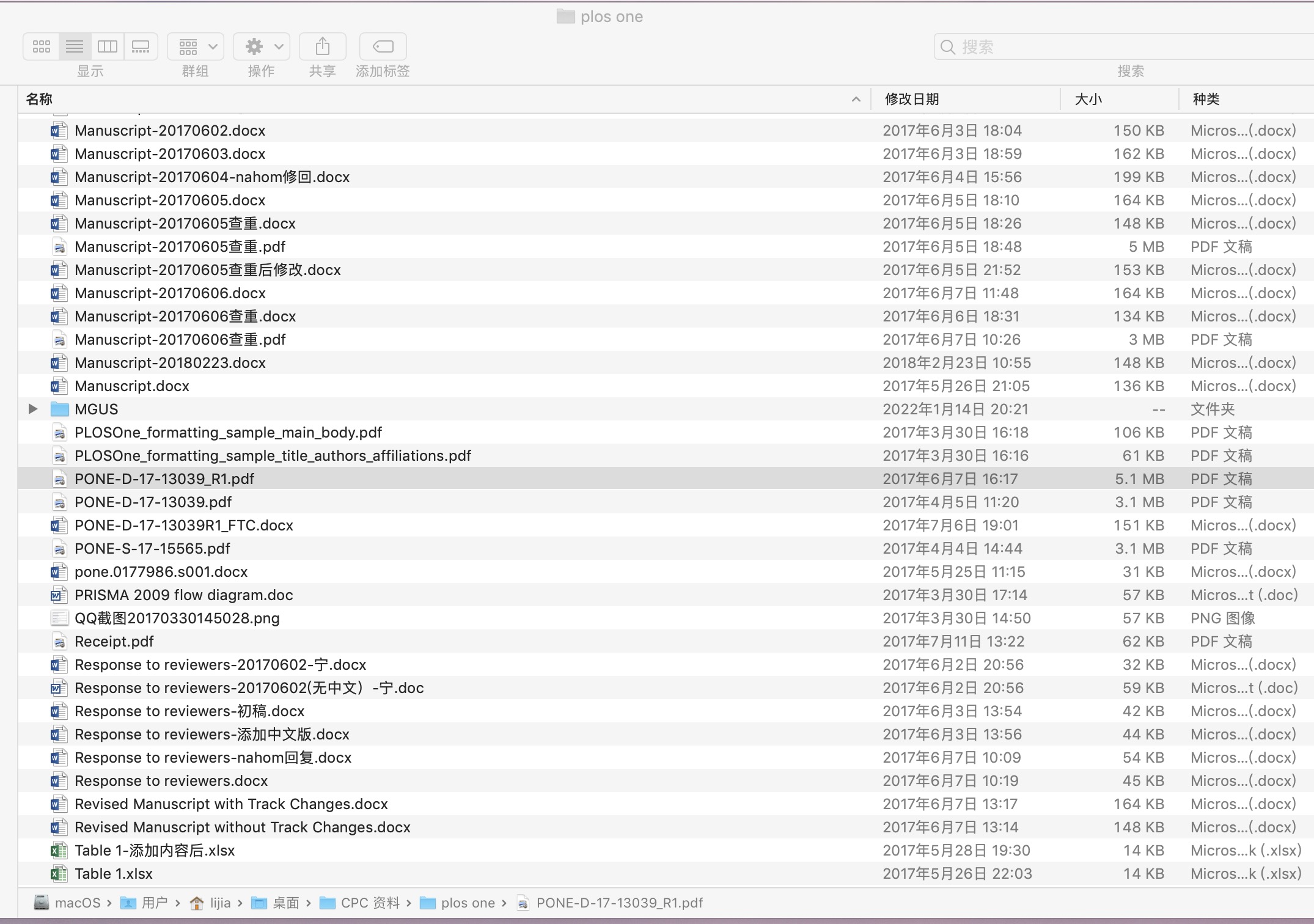

Supplement: S1 File — (ZIP) [file pone.0282230.s001.zip › primary data/all data.jpg]

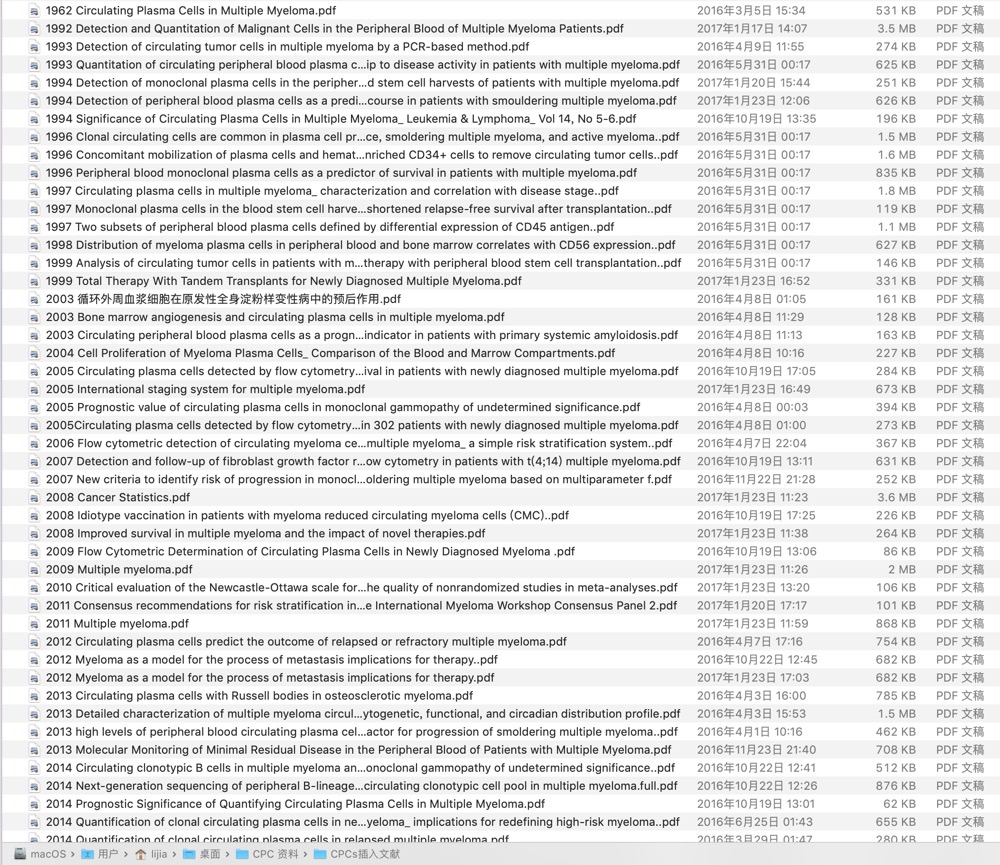

Supplement: S1 File — (ZIP) [file pone.0282230.s001.zip › primary data/primary screen.jpg]

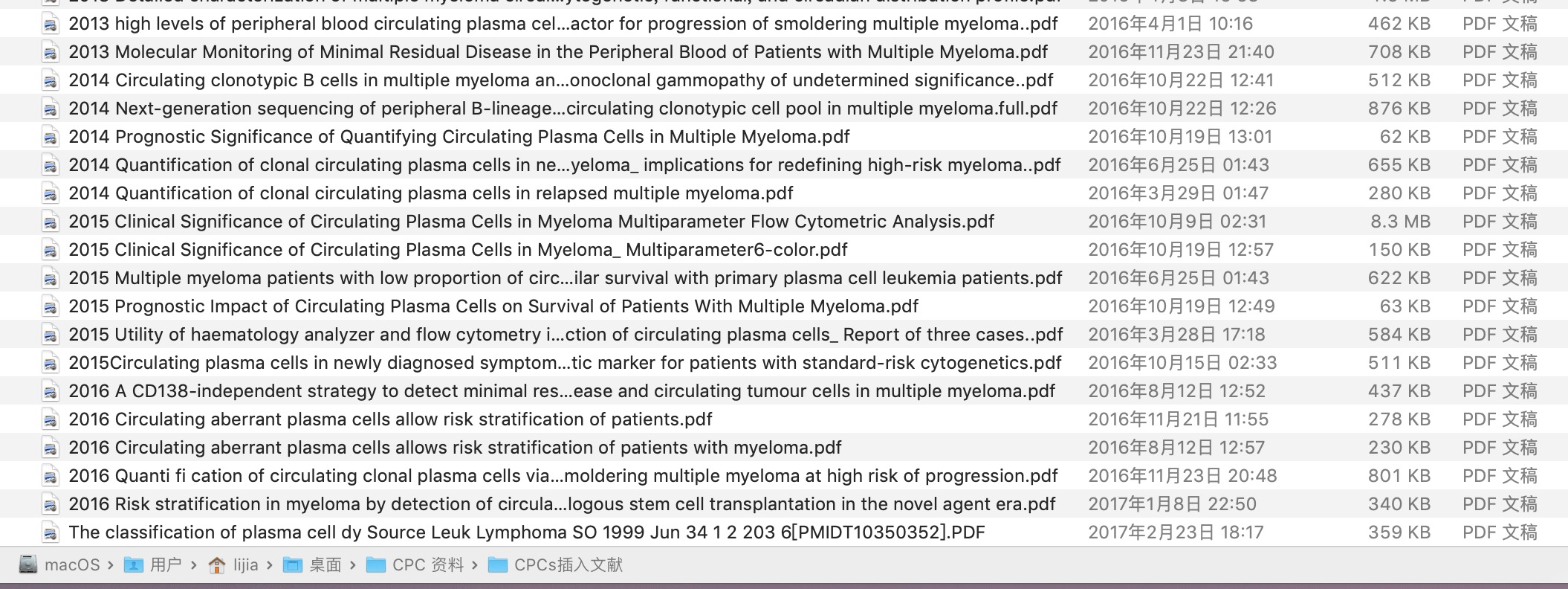

Supplement: S1 File — (ZIP) [file pone.0282230.s001.zip › primary data/primary screen2.jpg]

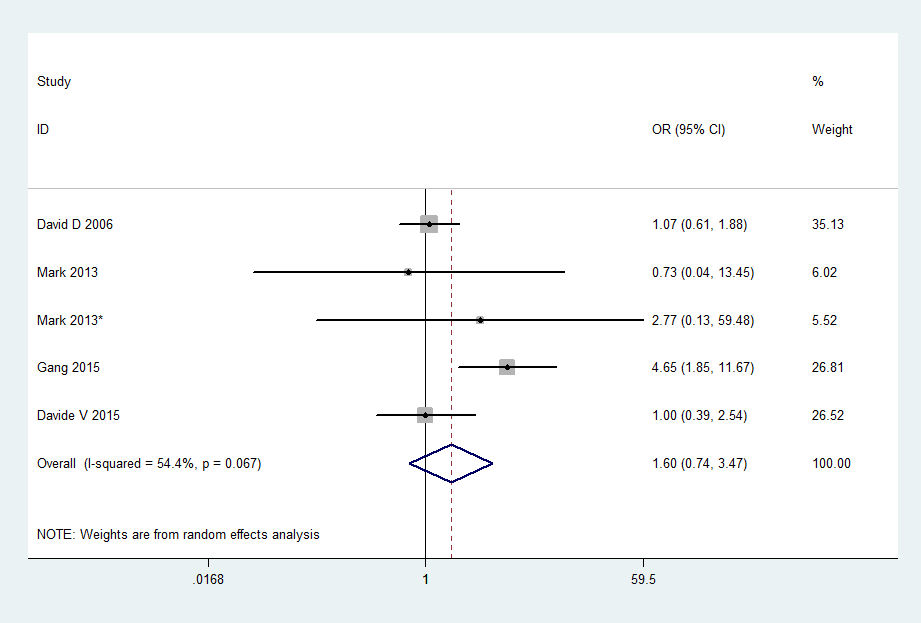

Supplement: S1 File — (ZIP) [file pone.0282230.s001.zip › primary data/stata20170109/D-S.png]

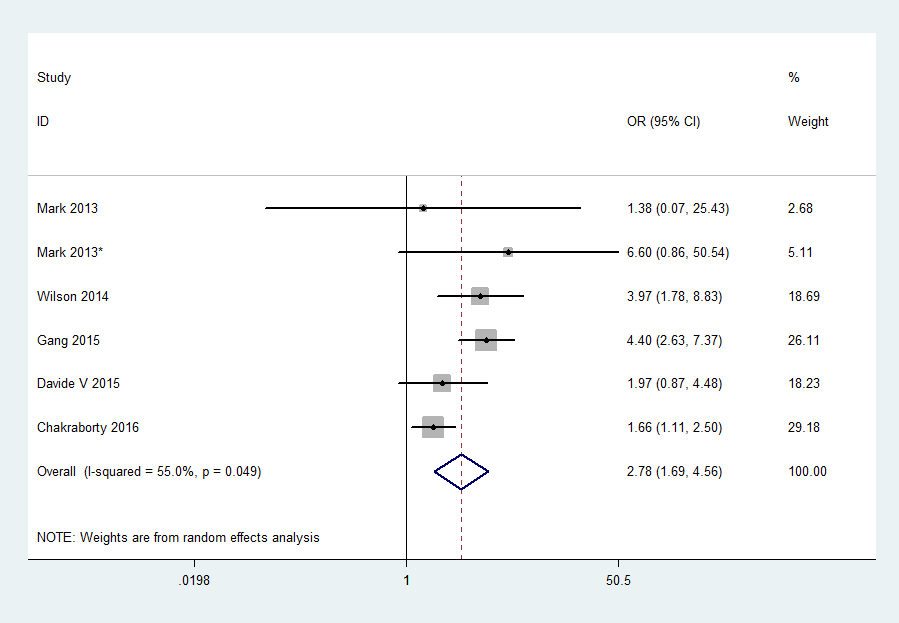

Supplement: S1 File — (ZIP) [file pone.0282230.s001.zip › primary data/stata20170109/ISS.png]

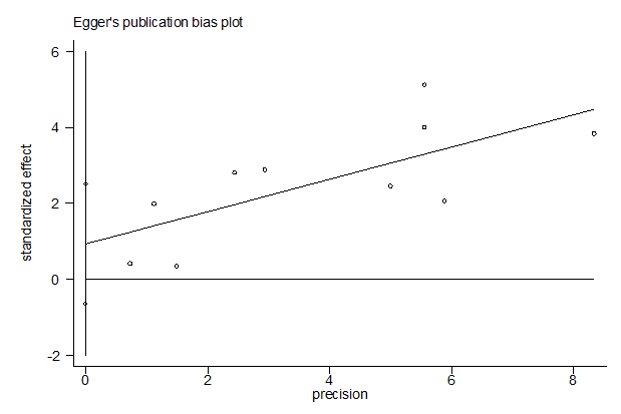

Supplement: S1 File — (ZIP) [file pone.0282230.s001.zip › primary data/stata20170109/os bias eggar.png]

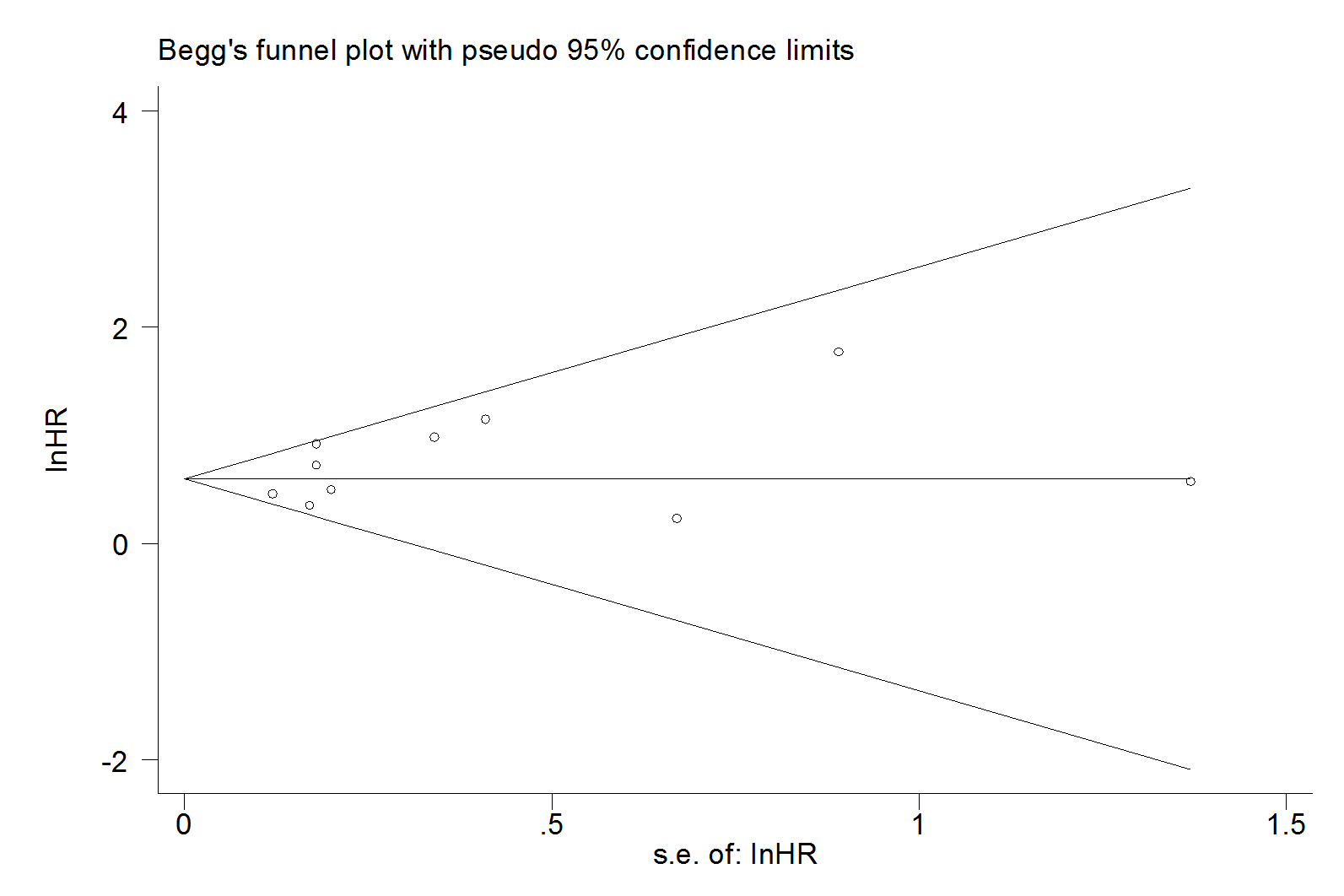

Supplement: S1 File — (ZIP) [file pone.0282230.s001.zip › primary data/stata20170109/OS BIAS.png]

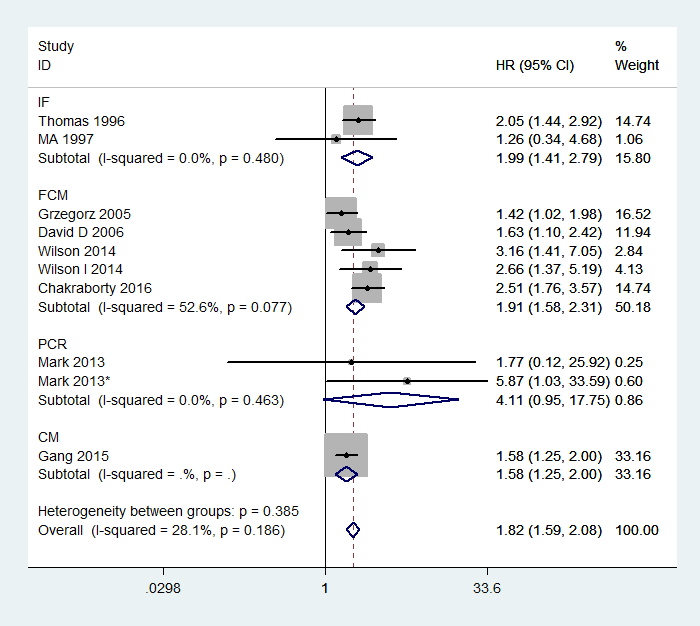

Supplement: S1 File — (ZIP) [file pone.0282230.s001.zip › primary data/stata20170109/OS METHOD.png]

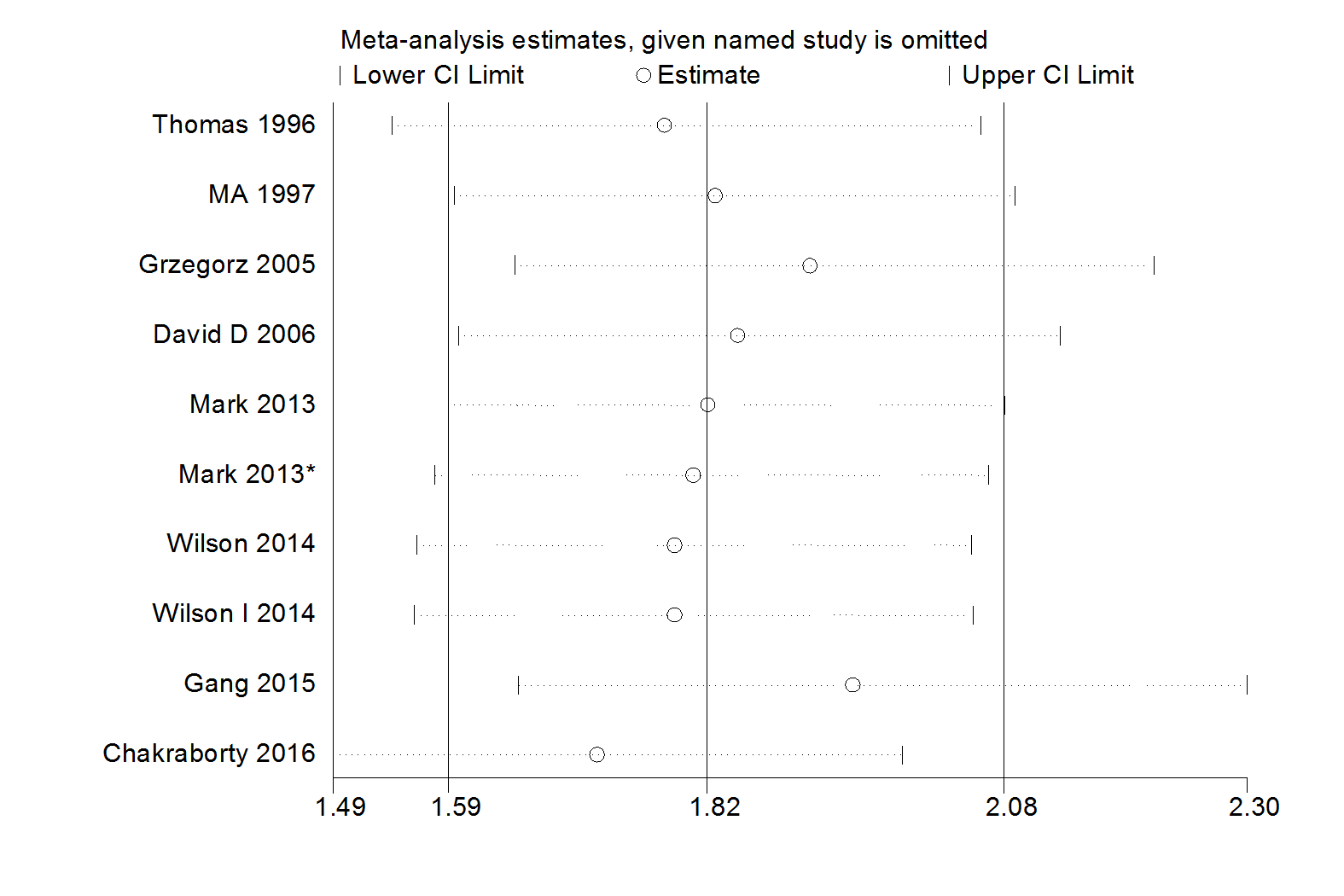

Supplement: S1 File — (ZIP) [file pone.0282230.s001.zip › primary data/stata20170109/OS SEN.png]

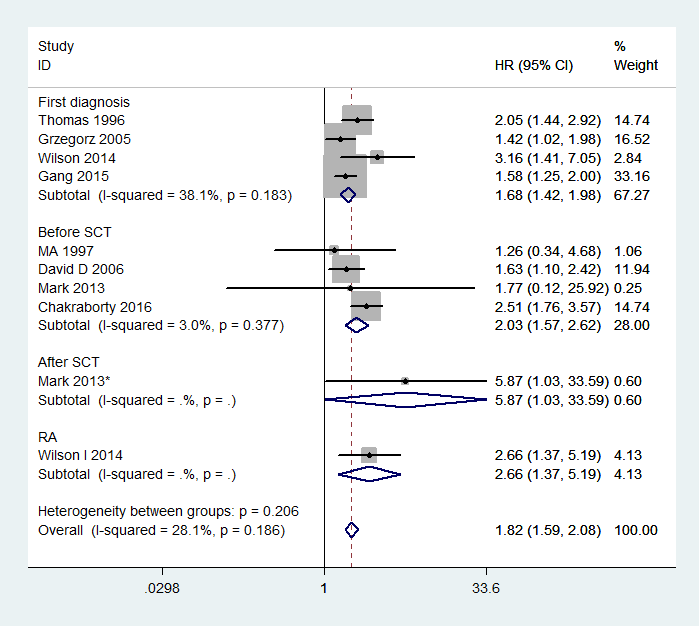

Supplement: S1 File — (ZIP) [file pone.0282230.s001.zip › primary data/stata20170109/OS TIME.png]

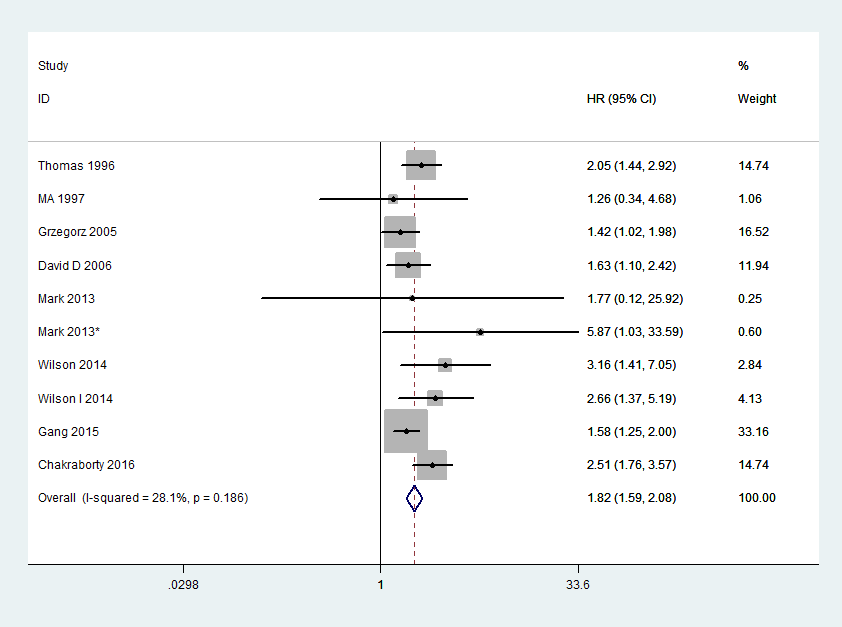

Supplement: S1 File — (ZIP) [file pone.0282230.s001.zip › primary data/stata20170109/OS.png]

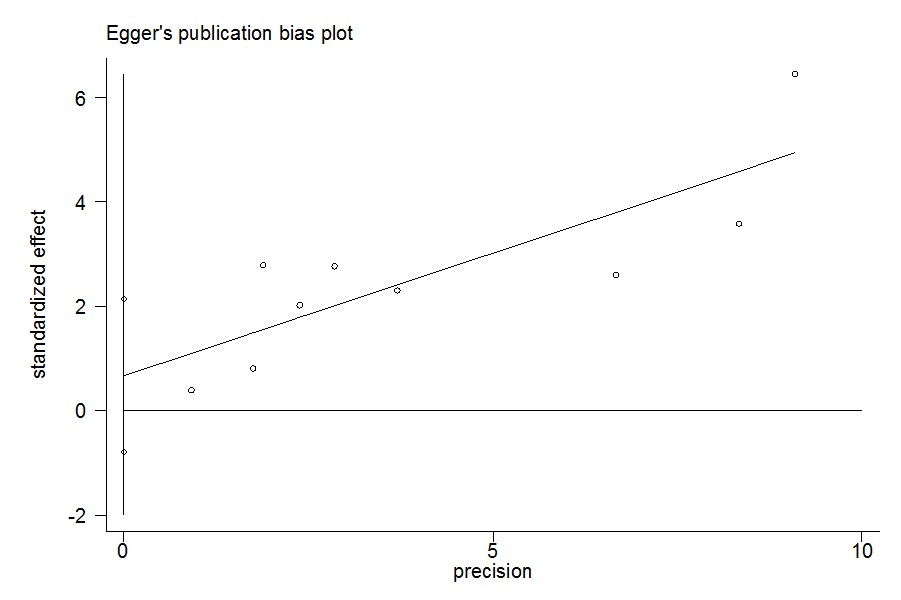

Supplement: S1 File — (ZIP) [file pone.0282230.s001.zip › primary data/stata20170109/PFS bias eggar.png]

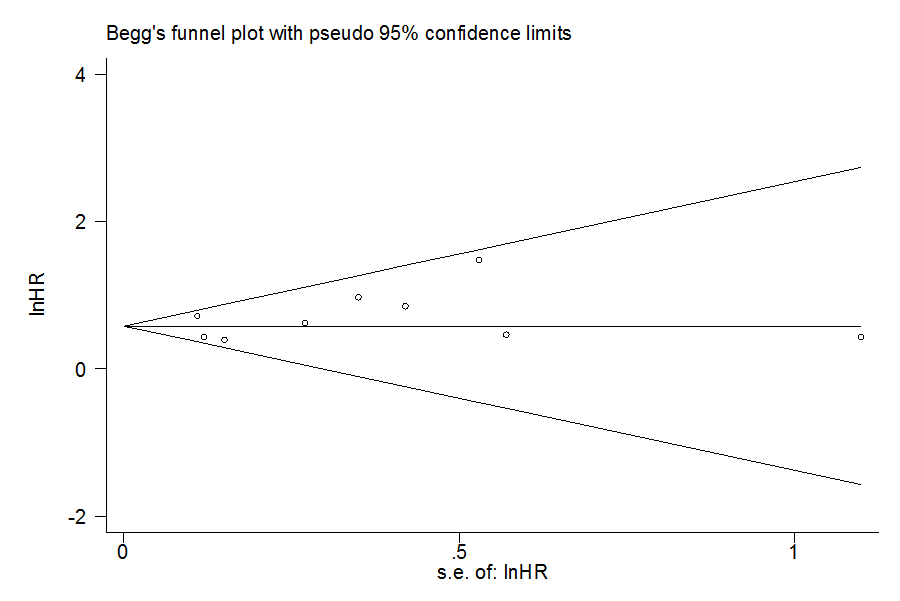

Supplement: S1 File — (ZIP) [file pone.0282230.s001.zip › primary data/stata20170109/PFS bias.png]

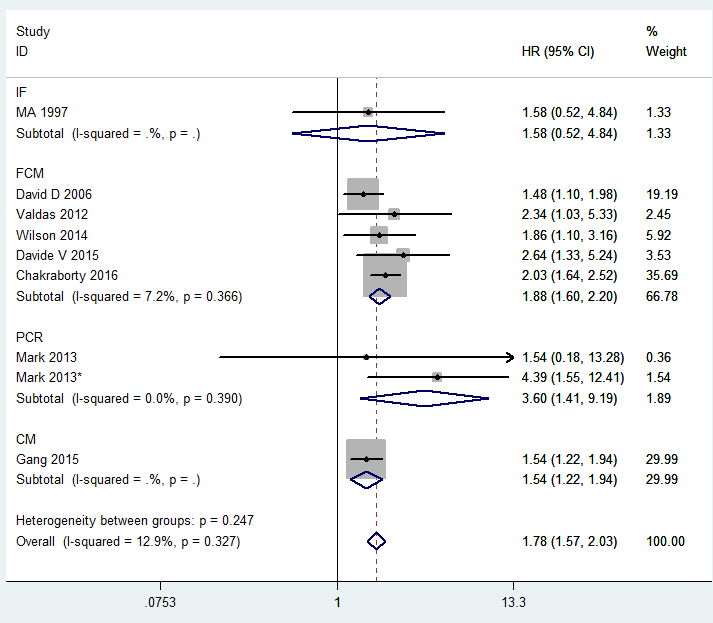

Supplement: S1 File — (ZIP) [file pone.0282230.s001.zip › primary data/stata20170109/PFS METHOD.png]

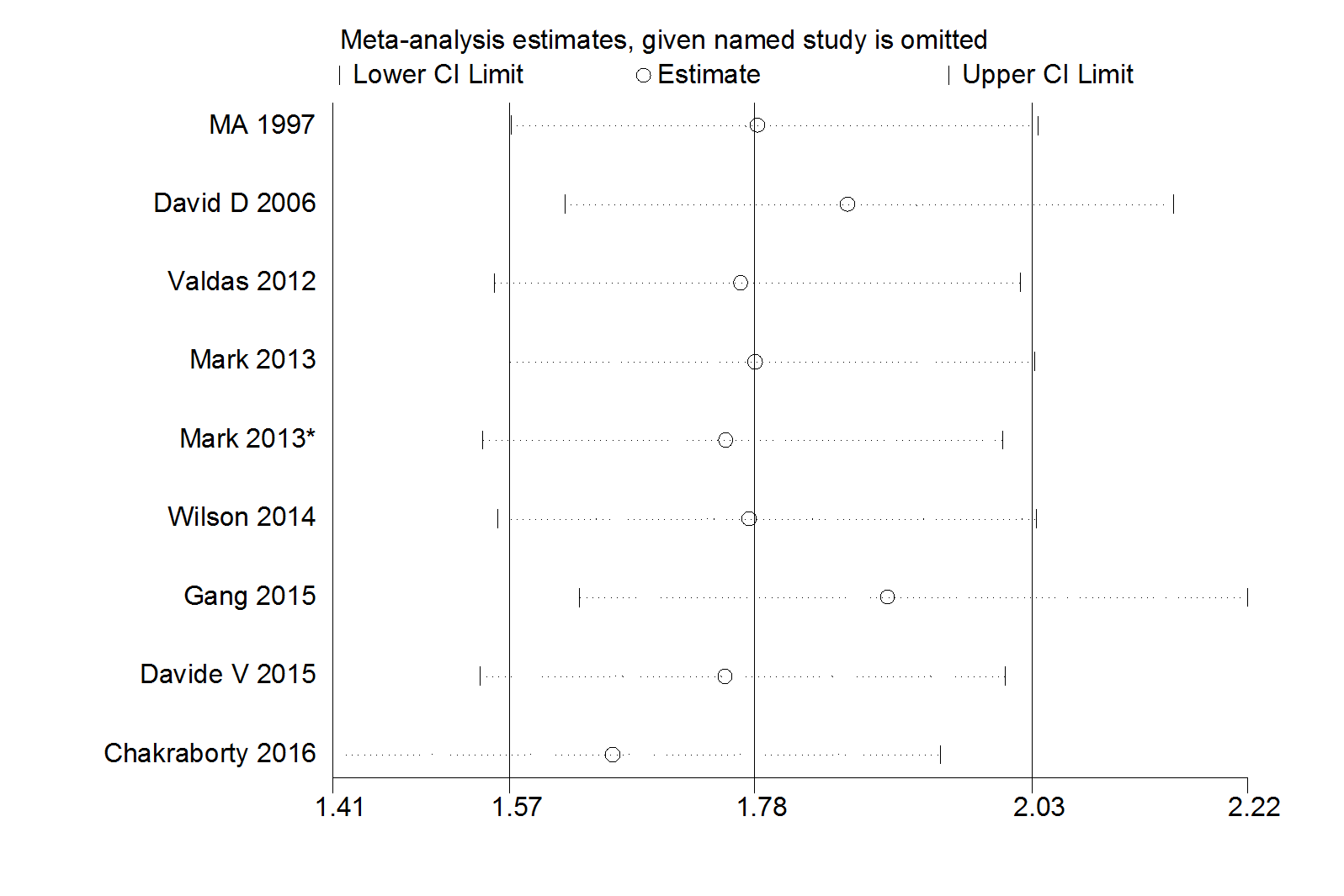

Supplement: S1 File — (ZIP) [file pone.0282230.s001.zip › primary data/stata20170109/PFS SEN.png]

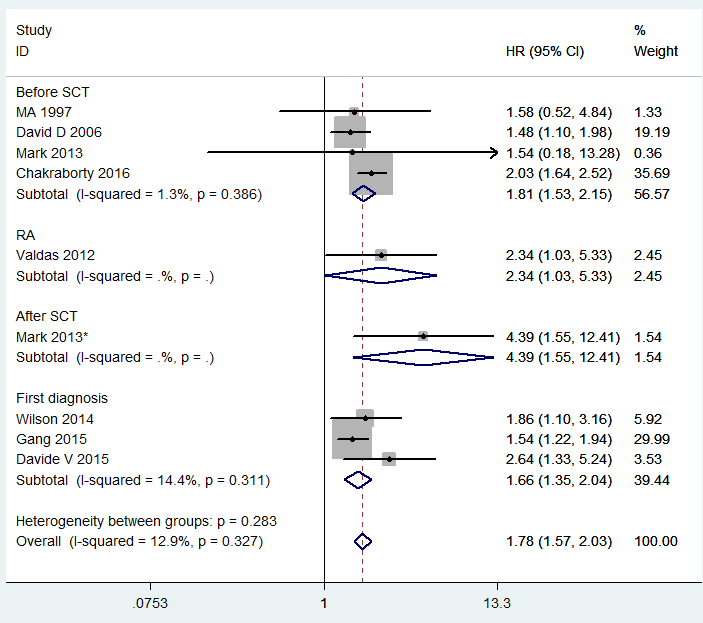

Supplement: S1 File — (ZIP) [file pone.0282230.s001.zip › primary data/stata20170109/PFS TIME 1.png]

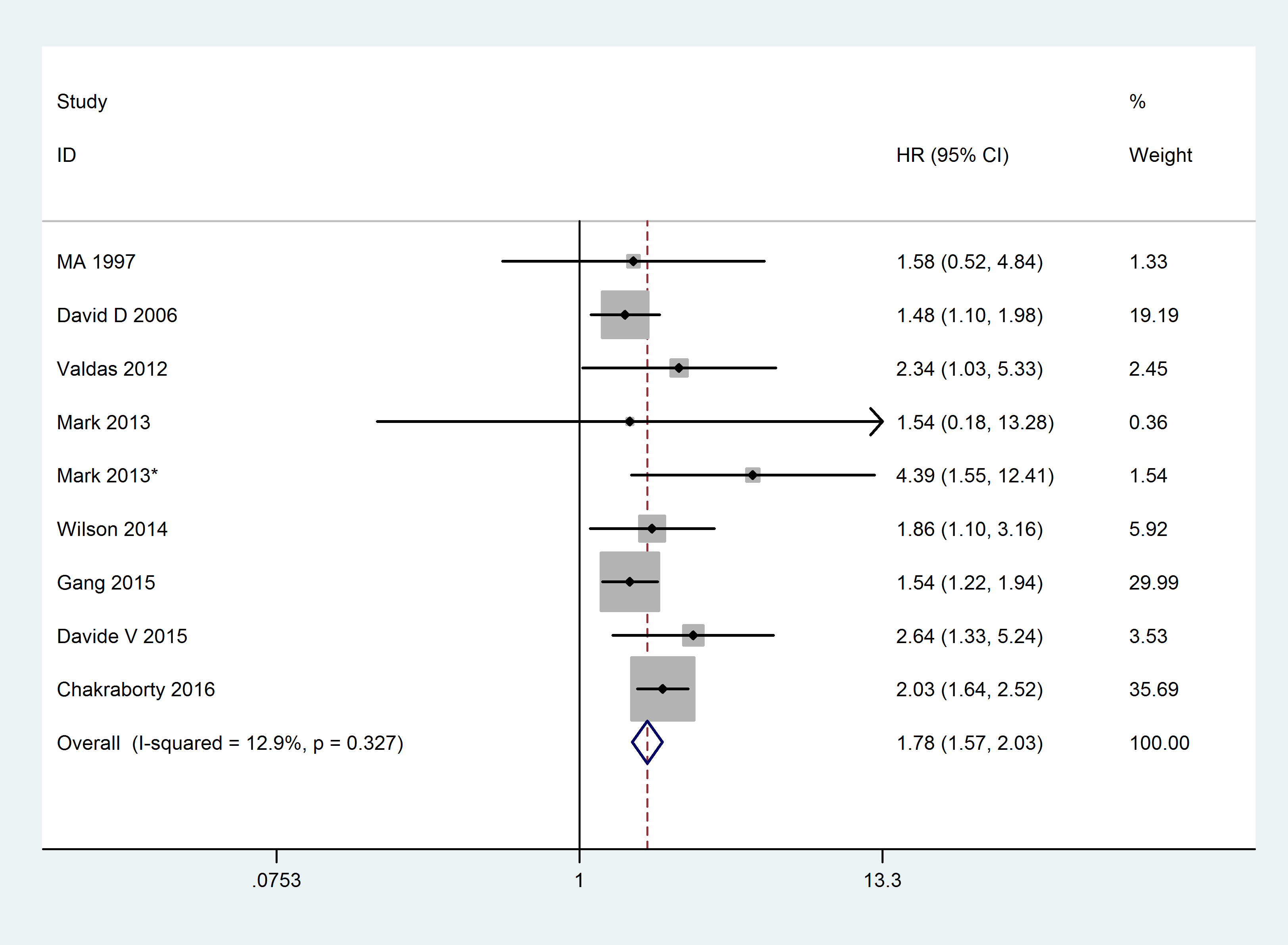

Supplement: S1 File — (ZIP) [file pone.0282230.s001.zip › primary data/stata20170109/PFS.png]
